# Irradiation of Bifunctional Masked Ketone Pro-Aromatics Unveils Autoinductive Autocatalysis via Electron Donor–Acceptor (EDA) Complexes

**DOI:** 10.1021/acs.orglett.5c02448

**Published:** 2025-08-25

**Authors:** Cheng-Lin Chan, Yong-Ting Tsao, Aira Shayne Paculba, Pei-Shan Lin, Zong-Nan Tsai, Hung-Hsuan Chiu, Risa Kunitake, Ming-Jia Chiu, Chun-Chi Yeh, Cheng-Chau Chiu, Hsuan-Hung Liao

**Affiliations:** † Department of Chemistry, 34874 National Sun Yat-sen University, Kaohsiung 804201, Taiwan (R.O.C.); ∥ Department of Chemistry, University of Rochester, New York 14627, United States; § Department of Applied and Medicinal Chemistry, Kaohsiung Medical University, Kaohsiung 807378, Taiwan (R.O.C.)

## Abstract

We disclose an autocatalytic electron donor–acceptor (EDA) strategy by reutilizing redox auxiliary byproducts as in situ acceptors, enabling an external initiator-free activation of pro-aromatic dihydroquinazolinones (DHQZs). Spectroscopic and DFT data support the Lewis acid-enhanced aggregate formation where DHQZ serves as both donor and latent acceptor through its quinazolinone byproduct. Kinetic studies reveal a kinetic profile specifically representing autoinductive autocatalysis. This platform enables Giese-type acylation/alkylation, desulfonylation, and Minisci reactions, forging C–C, C–N, C–S, and C–Se bonds under mild conditions.

## Graphical abstract

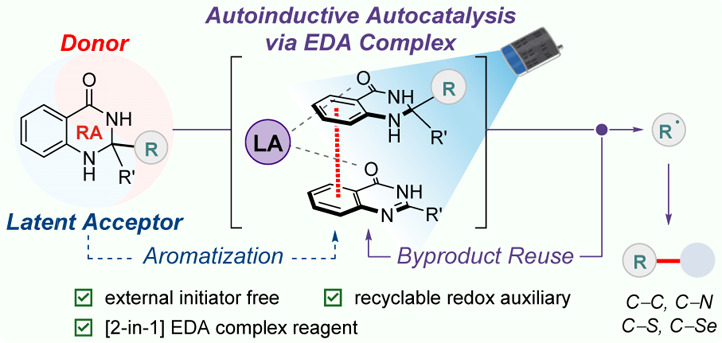

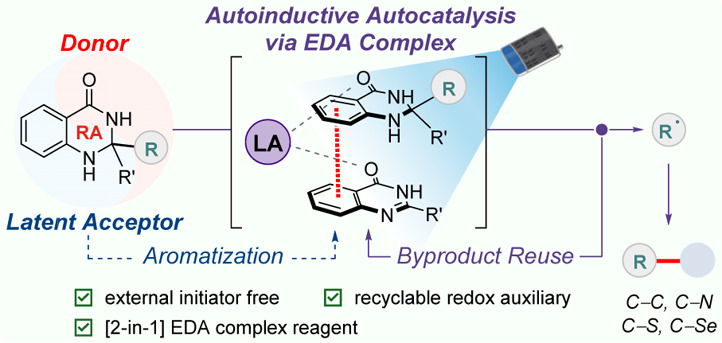

Harnessing visible light in organic reactions has enhanced synthetic capabilities by providing a mild and sustainable method for radical generation.^1^ In this context, photoredox catalysis remains a leading strategy for activating nonphotoactive organic compounds.^2^ However, an alternative approach utilizing electron donor–acceptor (EDA) complexes has gained attraction for their photocatalyst-free activation of colorless substrates under visible light.^3^ EDA complexes, formed through a reversible aggregation of electron-rich donor and electron-poor acceptor molecules, undergo photoinduced single-electron transfer (SET), which requires a leaving group for irreversible fragmentation into radicals.^4^ The challenges (e.g., limited scope) of relying on electronically biased substrates in the classical approach were addressed by introducing a redox auxiliary (RA) that activates neutral substrates, improving the EDA photochemistry (Scheme 1a). These RA-activated substrates can associate with apt partners to enable direct coupling,^5^ or undergo further diversification by suitable radical traps (RT).^6^ Despite the advancements, one challenge remains: *the RAs often accumulate as waste after fragmenting.*

**Scheme 1:**
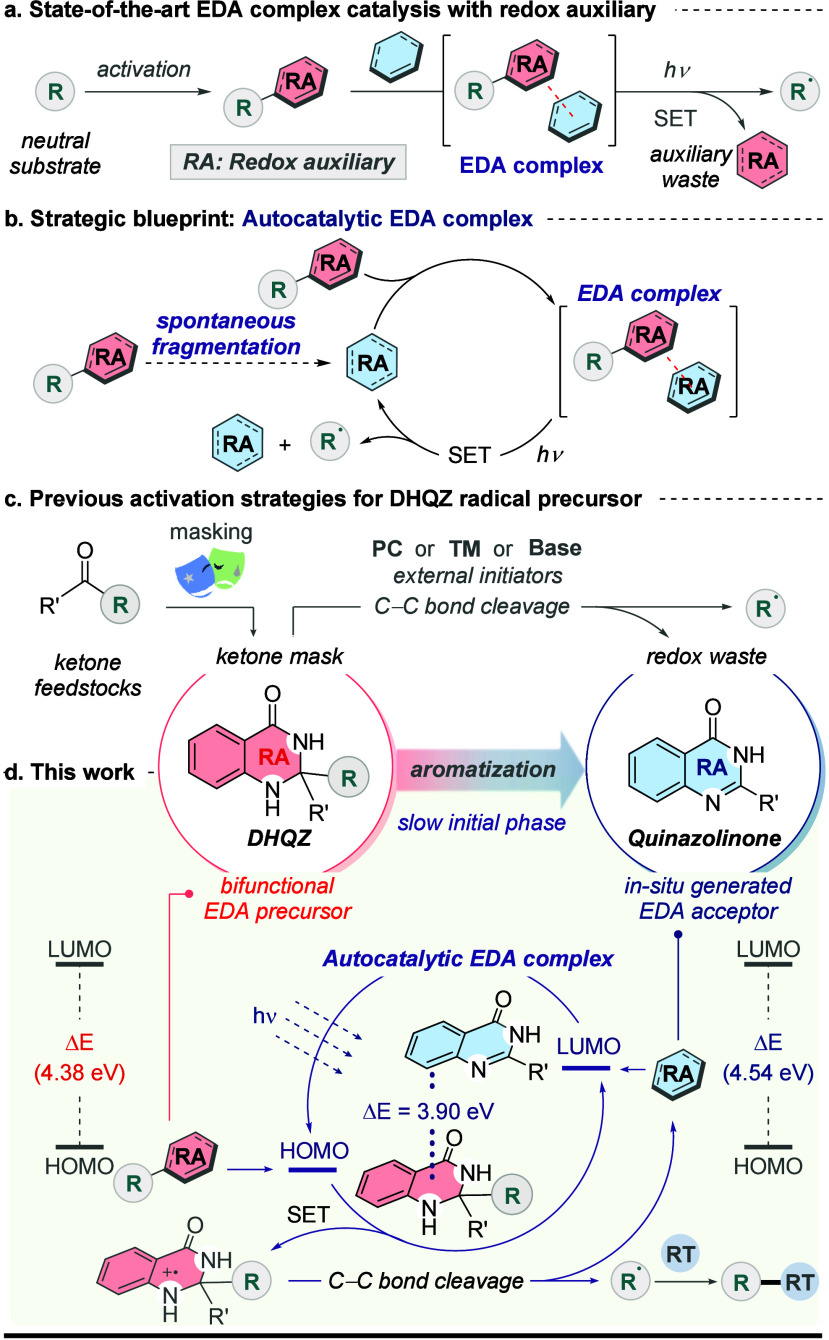
Background and Our Work^a^

a (a) General activation strategy of electronically neutral substrates with RA for EDA complex catalysis. (b) Autocatalytic EDA complex strategic blueprint. (c) Ketone masking with pro-aromatic dihydroquinazolinones (DHQZs) and their previous C–C bond scission strategies for radical generation. (d) External-initiator-free activation of DHQZs via the autocatalytic EDA complex approach.

Autocatalysis is generally referred to as reactions catalyzed by at least one of its products, which contrasts with stoichiometric and catalytic processes that rely on external promoters.^7^ Although autocatalysis is common in biochemical reactions,^8^ its relevance in abiotic contexts, like in organic synthesis, remains scarce.^9^ An incipient strategy known as photoredox autocatalysis has recently emerged, where the products act as photocatalysts, boosting their own formation or driving simultaneous reactions without external catalysts.^10^ Nevertheless, the photocatalyst-free alternative, EDA complexes, has not yet been reported to form and operate via an autocatalytic process.^11,12^ We herein present a blueprint for an autocatalytic EDA complex that repurposes the RA byproducts, addressing the RA waste issue. This system may specifically represent an autoinductive autocatalysis subtype,^7c^ wherein the RA byproduct indirectly promotes its own formation and subsequent reactions via EDA activation (Scheme 1b).

Pro-aromatic dihydroquinazolinones (DHQZs), derived from ketone feedstocks,^13^ are masked ketones that can facilitate group transfer.^14^ Driven by the aromatic stabilization energy (ASE), pro-aromatic DHQZs undergo C–C bond homolytic cleavage, typically facilitated by external initiators such as photocatalysts (PC),^15^ transition metals (TM),^16^ or a stoichiometric base,^17^ promptly releasing reactive radicals and aromatic quinazolinone byproducts (Scheme 1c). Building on our previous studies using PCs,^15b,15i^ we envisioned an external initiator-free C–C bond activation of DHQZ via an autocatalytic EDA complex strategy (Scheme 1d). We first considered the pro-aromatic DHQZ as the donor and the quinazolinone as the acceptor, which was supported by preliminary Density Functional Theory (DFT) calculations. Results revealed that their aggregation lowers the energy gap compared to the individual components, favoring intracomplex electron transfer.^18^ We further propose the autocatalytic initiation via aromatization-driven fragmentation of pro-aromatic precursor into its aromatic byproduct. This work highlights (i) the external initiator-free photochemical activation of DHQZ pro-aromatics, (ii) the first autocatalytic EDA mode repurposing the redox auxiliary byproduct, (iii) DHQZ as a bifunctional EDA reagent, and (iv) radical acylation/alkylation primarily via Giese-type conjugate addition.

To actualize our EDA strategic blueprint, 2-benzoyl-2-phenyl-2,3-dihydroquinazolin-4­(1*H*)-one **1a** and diethyl 2-ethylidenemalonate **2a** were chosen as model substrates in a Giese-type addition. We explored various Lewis acids, solvents, concentrations, and reaction times, achieving the highest yield of **3a** at 82% NMR yield (see (SI), Section 2.3). The reaction was successfully scaled to 1.0 mmol, affording **3a** in 85% isolated yield, thereby demonstrating the protocol’s scalability without compromising efficiency.

We then examined the generality of our protocol under the optimized conditions (Scheme 2). The scope of DHQZ radical precursors (**1a**–**m**) was first explored partnered with electron-deficient olefins, resulting in Giese adducts **3a**–**m** with fair to excellent yields. Besides aroyl radicals (**3a**–**c**), the less reactive propanoyl also efficiently provided **3d** in 75% yield. Similarly, the *p*-methoxybenzyl radical underwent smooth addition (**3e**, 73%). The protocol successfully accommodated a broader range of alkyl radicals compared to our previous system,^15b^ including secondary isopropyl (**3f**, 63%), cyclopentyl (**3g**, 52%), cyclohexyl (**3h**, 47%), indanyl (**3i**, 48%), as well as the bulky *tert*-butyl (**3j**, 42%) and adamantyl (**3k**, 69%) groups. A tetrahydropyran-derived radical afforded **3l** in 38%, while the acetal-derived radical, a masked formyl equivalent and a valuable synthetic handle, provided **3m** in 56% yield. These results significantly expand the synthetic utility of DHQZs, facilitating efficient radical transfer from various ketone feedstocks.

**Scheme 2:**
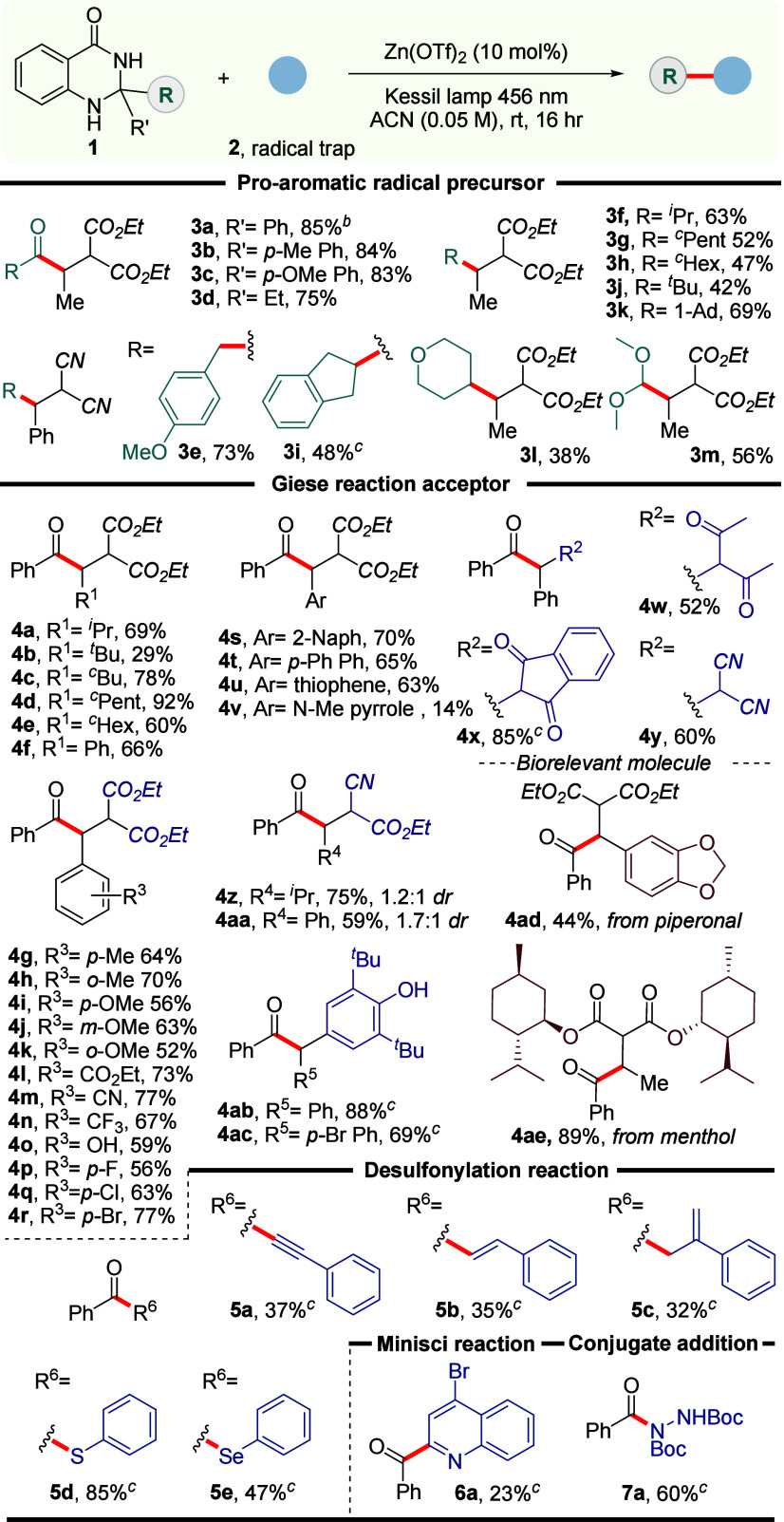
Substrate Scope^a^

a Reactions performed under the optimized conditions: **1** (0.15 mmol, 1.5 equiv), **2** (0.10 mmol, 1.0 equiv), Zn­(OTf)_2_ (0.01 mmol) in anhydrous acetonitrile (ACN) (2.0 mL, 0.05 M).
b Upscale condition: **1a** (1.5 mmol), **2** (1.0 mmol).
c
**1** (0.30 mmol, 3 equiv), **2** (0.10 mmol, 1 equiv). *dr* = diastereomeric ratio.

Next, the electron-deficient olefins were evaluated using DHQZ **1a**. The aliphatic (**4a**, 69%) and cyclic (**4c**–**e**, 60–92%) alkyl substituents at R^1^ delivered moderate to excellent yields of Giese adducts except for the *tert*-butyl analogue (**4b**, 29%), hampered by steric effects. The investigation considered the impact of aryl substitutions on the alkene, revealing that all neutral (**4f**–**h**), electron-donating (**4i**–**k**), and electron-withdrawing (**4l**–**n**) substitutions performed comparably, suggesting a limited impact on the reaction efficiency. Meanwhile, versatile synthetic handles, phenol (**4o**, 59%) and halogens (**4p**–**r**, 56–77%), remained intact. Moderate to good yields were achieved with substrates containing conjugated π-systems like naphthyl (**4s**, 70%) and biphenyl (**4t**, 65%). Heteroaromatic alkenes, including thiophene (**4u**, 63%) and *N*-methylpyrrole (**4v**, 14%), underwent successful acylation, albeit with varied efficiency. Changing the R^2^ with diacetyl (**4w**), dione (**4x**), and dinitrile (**4y**) afforded the corresponding products in fair to good yields (52–85%). Olefins with unsymmetrical electron-withdrawing groups produced diastereomeric adducts, achieving diastereomeric ratios of 1.2:1 for isopropyl (**4z**, 75%) and 1.7:1 for phenyl (**4aa**, 59%) substitutions at the R^4^ position. Acylation of *p*-quinone methides yielded aromatized products **4ab** (88%) and **4ac** (69%). Additionally, the potential of our protocol to modify biomolecules into complex chemical entities through late-stage functionalization (LSF) was also demonstrated with piperonal (**4ad**, 44%) and menthol (**4ae**, 89%) derivatives.

Encouraged by the feat of the Giese-type addition, we extended our studies to other radical acceptors. The sulfones underwent desulfonylative acylation. Notably, alkynyl sulfone afforded ynone **5a** (37%), a motif typically synthesized via Sonogashira coupling. Vinyl and allyl sulfones gave α,β-unsaturated ketone **5b** (35%) and acylated styrene derivative **5c** (32%), respectively, with retention of olefinic character. Beyond C–C bond formation, the protocol enabled C–heteroatom bond construction. C–S and C–Se bonds were formed efficiently, furnishing phenyl thiobenzoate **5d** (85%) and selenoester **5e** (47%). We also accomplished a Minisci-type acylation using brominated quinoline as a radical trap, generating product **6a** (23%). Finally, conjugate addition to di-*tert*-butyl azodicarboxylate furnished hydrazine derivative **7a** in 60% yield. These results highlight the broad utility of our protocol beyond Giese-type additions, offering a complementary platform for constructing diverse C–C and C–heteroatom bonds from DHQZ-derived radicals.

A preliminary photoflow application was tested to assess industrial-scale use potential. The model substrates DHQZ **1a** and olefin **2a** gave 73% yield of **3a** (see , Section 2.7).

We then conducted a series of mechanistic studies to shed light on the possible mechanism of this autocatalytic EDA complex (see , Section 4). The EDA complex formation was investigated using UV–visible absorption measurements with ACN solvent. We first ruled out the possibility of electron-deficient olefin **2a** as an acceptor via Lewis acid coordination (see , Section 4.4). Then we examined the in situ-generated quinazolinone **1a′** (Figure 1a). Isolated for further absorption measurements, **1a′** revealed a minimal absorbance in the visible region. Interestingly, mixing **1a** and **1a′** (purple) elicited a substantial bathochromic shift and an even more pronounced charge-transfer band around the irradiation wavelength after adding the Lewis acid (violet). Furthermore, adding Zn­(OTf)_2_ to **1a** (orange) and **1a′** (dark blue) led to a noticeable red shift in each solution, suggesting an interaction of the Lewis acid with these likely EDA complex constituents. From this, we postulated the EDA complex intermediate, showing the interaction of Zn­(OTf)_2_ toward both **1a** and **1a′**. The evidence of the EDA complex was likewise observed in the color change from pale to an intense yellow when mixing solutions of **1a**, **1a′**, and Zn­(OTf)_2_.

**Figure 1:**
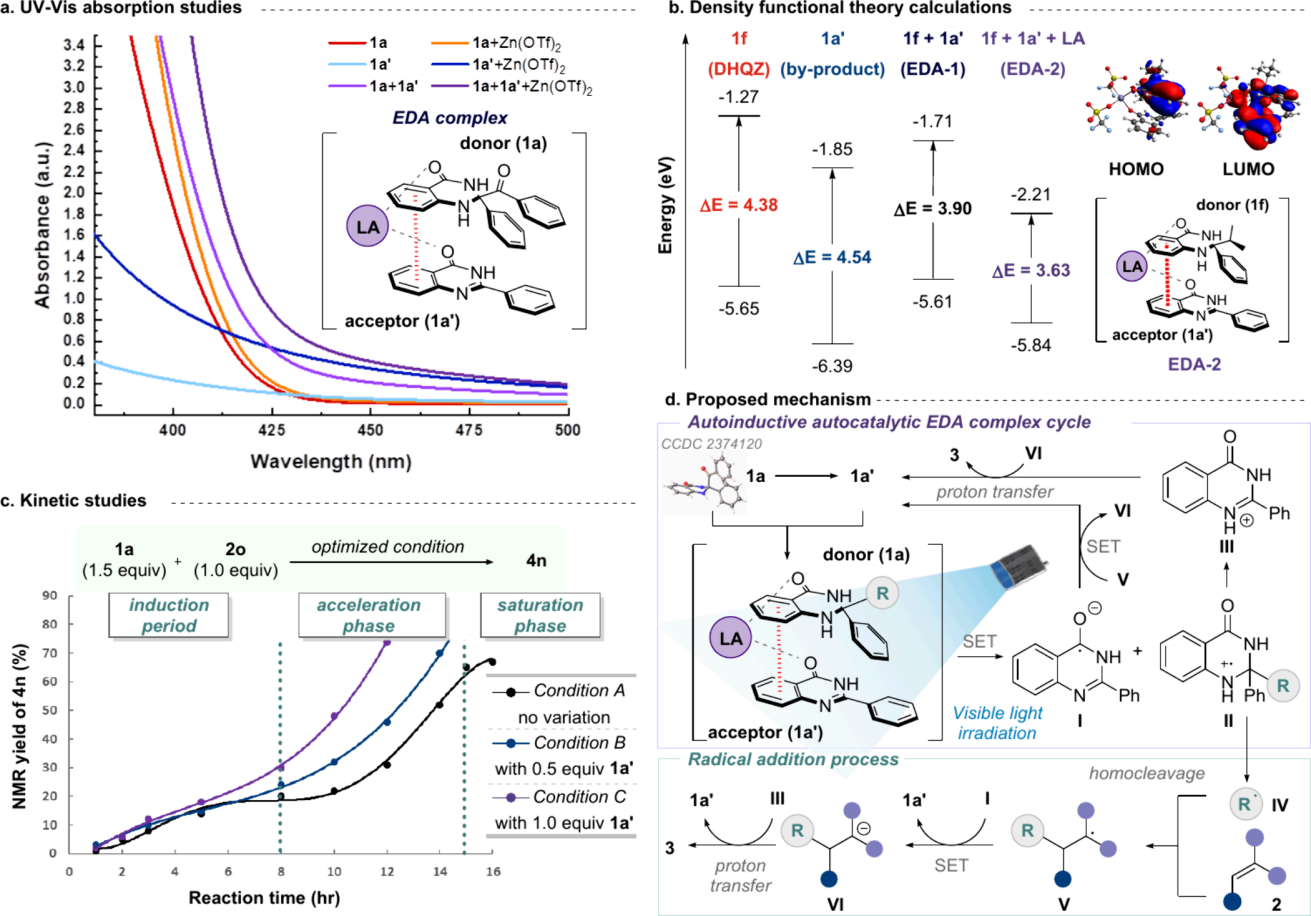
Mechanistic studies. (a) UV–vis absorption experiments. (b) DFT calculations of the HOMO/LUMO for the energy gap value comparison. (c) Reaction monitoring to evaluate the effect of adding **1a′** on the reaction rate. (d) Proposed mechanism for the autoinductive autocatalytic EDA complex catalysis.

DFT calculations employed DHQZ (**1f**) and **1a′** as EDA components (Figure 1b). The energy gap of the hypothesized aggregate (EDA-1, **1f**+**1a′**) was found to be 3.90 eV, lower than those of the individual donor (**1f**, 4.38 eV) and acceptor (**1a′**, 4.54 eV). However, the calculated Gibbs energy for EDA-1 formation (+4.0 kJ/mol) suggests a low propensity for EDA complex formation. Given the observed influence of the Lewis acid (**LA**) on both yield and EDA complex formation, as indicated by the UV–vis experiments, we further calculated the HOMO/LUMO gap for the three-component EDA complex model (EDA-2, **1f**+**1a′**+**LA**) by integrating Zn­(OTf)_2_ into the EDA-1. In this case, EDA-2 exhibited a reduced energy gap of 3.63 eV, and its calculated Gibbs energy was −5.7 kJ/mol, suggesting a favorable complex formation. These DFT results highlight the significant role of the Lewis acid in facilitating the interaction between DHQZ and quinazolinone for efficient intracomplex charge transfer. Moreover, the HOMO is primarily localized on DHQZ, while the LUMO is on quinazolinone, supporting their roles as donor and acceptor, respectively.

Finally, we performed kinetic studies to evaluate the effect of adding **1a′** on initial reaction rates to investigate the autocatalytic nature of this EDA complex, while considering the specific subtype, autoinductive autocatalysis.^7d^ The Giese reaction of **1a** and olefin **2o** was monitored under three conditions: condition A (black, optimized condition), condition B (blue, with 0.5 equiv **1a′** additive), and condition C (violet, with 1.0 equiv **1a′** additive) (Figure 1c). Under condition A, the conversion to **4n** is slow within 8 h with minimal increase until 12 h, then accelerates but plateaus by 15–16 h (see ). Notably, increasing the amount of **1a′** (0.5 to 1.0 equiv) shortened the induction period and enhanced the conversion to **4n** within 8 h, with further acceleration observed beyond that point. Nonetheless, the induction period persists, which aligns with the indirect promotion of **1a′** through EDA formation. These results suggest a kinetic profile supporting the *autoinductive autocatalysis*.^19^

A plausible mechanism is depicted in Figure 1d. Without external initiators,^20^ the C–C bond cleavage of DHQZ **1** generates quinazolinone **1a′** in situ. Coordination with Zn­(OTf)_2_ enhances the aggregation of **1** and **1a′**, forming the putative EDA complex. Upon visible-light irradiation, SET generates radical ion pairs **I** and **II**. The oxidized DHQZ **II** undergoes homolytic cleavage, yielding protonated species **III** and free radical **IV**, which is captured by **2** to form open-shell intermediate **V**. Intermediate **V** oxidizes the reduced quinazolinone **I** via SET, restoring its aromaticity to generate **1a′** and forming **VI**. The anionic adduct **VI** undergoes proton transfer with **III**, affording the final product **3** while liberating **1a′**. The regenerated quinazolinone **1a′** sustains the autocatalytic cycle by acting as an in situ acceptor for subsequent EDA complex catalysis.

In summary, we have developed an autoinductive autocatalytic EDA complex platform that enables mild, initiator-free photochemical radical generation from ketone-derived DHQZs. This strategy offers a versatile and efficient route for various chemical modifications, including acylation, alkylation, C–heteroatom bond formation, and biomolecule modification, beyond Giese additions. Central to this reactivity is the dual role of DHQZ as both donor and latent acceptor, with the quinazolinone byproduct indirectly promoting the transformation via EDA complex formation with DHQZ and a Lewis acid. This work expands the scope of EDA photochemistry and introduces a unique concept of photoautoinductive autocatalysis, with demonstrated scalability and potential for photoflow adaptation.

## Supplementary Material





## Data Availability

The data underlying this study are available in the published article and its

## Notes

FAIR data, including the primary NMR FID files, for compounds **1a**–**1m**, **1a′**, **2b**–**2f**, **2h**–**2z**, **2aa**–**2ab**, **2ad**–**2ak**, **3a**–**3m**, **4a**–**4z**, **4aa**–**4ae**, **5a**–**5e**, **6a**, **7a** (ZIP)
Experimental procedure; mechanistic studies; computational details; single crystal X-ray diffraction analysis; reference and NMR spectra (PDF)
