# Supplementary figures and images for: Irradiation of Bifunctional Masked Ketone Pro-Aromatics Unveils Autoinductive Autocatalysis via Electron Donor–Acceptor (EDA) Complexes
Source: Org Lett. 2025 Aug 25;27(35):9593–8. doi: 10.1021/acs.orglett.5c02448 (PMC12418491; doi:10.1021/acs.orglett.5c02448)

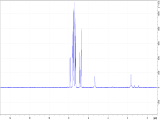

Supplement: Supplementary file 1 [file ol5c02448_si_001.zip › NMR raw data/1a/1H/pdata/1/thumb.png]

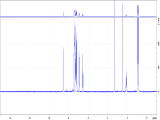

Supplement: Supplementary file 1 [file ol5c02448_si_001.zip › NMR raw data/1f/1H/pdata/1/thumb.png]

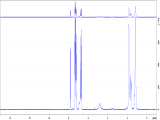

Supplement: Supplementary file 1 [file ol5c02448_si_001.zip › NMR raw data/1h/1H/pdata/1/thumb.png]

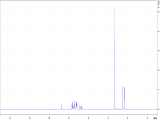

Supplement: Supplementary file 1 [file ol5c02448_si_001.zip › NMR raw data/1b/1H/pdata/1/thumb.png]

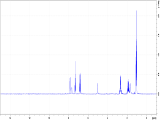

Supplement: Supplementary file 1 [file ol5c02448_si_001.zip › NMR raw data/1d/1H/pdata/1/thumb.png]

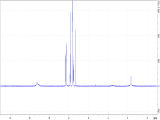

Supplement: Supplementary file 1 [file ol5c02448_si_001.zip › NMR raw data/1a'/1H/pdata/1/thumb.png]

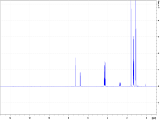

Supplement: Supplementary file 1 [file ol5c02448_si_001.zip › NMR raw data/2b/1H/pdata/1/thumb.png]

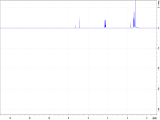

Supplement: Supplementary file 1 [file ol5c02448_si_001.zip › NMR raw data/2c/1H/pdata/1/thumb.png]

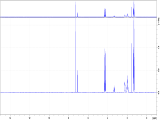

Supplement: Supplementary file 1 [file ol5c02448_si_001.zip › NMR raw data/2d/1H/pdata/1/thumb.png]
