# Supplementary material for: Irradiation of Bifunctional Masked Ketone Pro-Aromatics Unveils Autoinductive Autocatalysis via Electron Donor–Acceptor (EDA) Complexes
Source: Org Lett. 2025 Aug 25;27(35):9593–8. doi: 10.1021/acs.orglett.5c02448 (PMC12418491; doi:10.1021/acs.orglett.5c02448)

Supplementary Information

Irradiation of Bifunctional Masked Ketone Pro-Aromatics Unveils Autoinductive Autocatalysis via EDA Complexes

Cheng-Lin Chan,[a], ‡ Yong-Ting Tsao,[a], ‡ Aira Shayne Paculba,[a] Pei-Shan Lin,[a] Zong-Nan Tsai,[a] Hung-Hsuan Chiu,[a] Risa Kunitake,[a, b] Ming-Jia Chiu,[a] Chun-Chi Yeh,[a] Cheng-Chau Chiu,[a] Hsuan-Hung Liao*[a, c]

[a] Department of Chemistry, National Sun Yat-sen University, Kaohsiung 804201, Taiwan (R.O.C.)
[b] Department of Chemistry, University of Rochester, New York 14627, United States
[c] Department of Applied and Medicinal Chemistry, Kaohsiung Medical University, Kaohsiung 807378, Taiwan (R.O.C.)
[‡] These authors contributed equally.

**Corresponding author. Email: hsuan-hung.liao@mail.nsysu.edu.tw*

Contents

1. General information 2
1.1 Pre-treatment of experiments, solvents, and reagents 2
1.2 Chromatography, data analysis, and collection 2
1.3 Instrument catalogue 2
1.4 Purchasing reagent 4
2. Experimental procedure 8
2.1. Preparation of dihydroquinazolinones (DHQZs) 8
2.2. Preparation of Radical traps 12
2.3. Condition optimization of Autocatalytic EDA promoted Giese reaction 26
2.4. Procedure of Autocatalytic EDA Complex Acylation/Alkylation 28
2.5. Substrate Scope of Autocatalytic EDA Complex Acylation/Alkylation 29
2.6. Scope limitation 82
2.7. Flow synthesis 83
3. Background and comparison of reaction types 84
3.1. EDA concept and comparison of different strategies 84
3.2. Autoinductive autocatalysis reaction concept and comparison 85
4. Mechanistic studies 86
4.1. Background reaction 86
4.2. Radical trapping experiments 86
4.3. Light on-off experiments 87
4.4. UV-vis spectra 88
4.5. NMR investigation of EDA complex formation 89
4.6. Investigation of 1a’ formation 90
4.7. Investigation of autoinductive autocatalytic reaction 91
4.8. Quantum yield calculation 93
5. Computational details 94
5.1 Characterization of structures 94
5.2 HOMO-LUMO calculation 99
6. Single crystal X-ray diffraction analysis 100
7. References 111
8. NMR spectra 114

1. General information

1.1 Pre-treatment of experiments, solvents, and reagents

Glassware All glassware has been oven-dried after cleaning with a cleaning machine, Steelco LAB 500CL.

Solvents All solvents were purchased from suppliers. Unless otherwise stated, the following ACS grade solvents (Acetonitrile, 1,4-Dioxane, Diethyl Ether, DCM, DMF, THF, Toluene) were stored over microwave-activated 3Å molecular sieves for at least one night and transferred into anhydrous engineering alumina column drying system (Vigor Gas Purification Technologies Co., Ltf, VSPS-7) before use.

Chemicals To maintain the activity, sensitive compounds such as NHC catalysts and aldehyde were stored in the glovebox. Other chemicals were directly used as received and were always filled with nitrogen before storage and twined with parafilm carefully. See section **S1.4** for the complete supplier list of each chemical.

1.2 Chromatography, data analysis, and collection

Thin-layer Chromatography (TLC) Merck aluminium-backed sheets coated with $60F_{254}$ silica gel. The silica plate was visualized using a UV lamp (λ_{max} = 254 nm).

Column chromatography Column chromatography was carried out using KM3 scientific silica gel (45 – 75 μm) purchased from KM3 scientific.

1.3 Instrument catalogue

Nuclear Magnetic Resonance (NMR) ^{1}H-, ^{13}C- and ^{19}F- Nuclear Magnetic Resonance (NMR) spectra were used to identify the structure of starting materials and products by using Bruker Avance 300 MHz, Jeol ECZS 400 MHz, Bruker Avance 500 MHz and Jeol ECZR 600 MHz. Coupling constants are abridged as follows: s = singlet, d = doublet, t = triplet, q = quartet, quin = quintet, sext = sextet, sept = septet, m = multiplet, dd = doublet of doublet.

Gas Chromatography–Mass Spectrometry (GC-MS) The operation method was set as follows: 1.0 mL sample was injected by auto-sampler in a split mode (100:1) with 0.5 mL air gap into the GC-MS system consisting of an Agilent 8860 gas chromatograph, an Agilent 5977B mass selective detector, and Agilent 7693A autoinjector. Gas chromatography was performed on a 30 m HP-5MS with 0.25 mm inner diameter (I.D.) and 0.25 mm film thickness with an initial injection temperature of 50 ºC to 300 ºC, MSD transfer line of 280 ºC, and the ion source adjusted to 230 ºC. The helium carrier gas was set at a constant flow rate of 1.197 ml min-1. The mass spectrometer was operated in positive electron impact mode (EI), with ionization energy in the m/z 50 – 550 scan range. The spectra of all chromatogram peaks were evaluated using the MSD Chemstation.

High-Resolution Mass Spectra (HRMS) Jeol AccuTOF GCx-plus / Shimadzu QP2020

UV-Vis & UV-Vis-NIR Instruments Agilent Cary5000, a high-performance UV-Vis and NIR spectrophotometer with superb photometric performance in the 175–3300 nm range.

Table S1 Supplier of equipment and instruments

Equipment	Supplier
Electronic balance	*Shimadzu* UW2200H/ ATX224
Hot plate stirrer	*Corning* PC-420D
Immersion cooler	*Panchum* IC-9090
Pump of rotary evaporator	*KNF Laboport* N820.3FT.18
Rotary evaporator	*Heidolph* Hei-Vap Core HL G3
Refrigerated circulator bath	*Panchum* CC-300
Ultra-low temp. reaction bath	*Panchum* UR-8500
Vacuum pump	*Edwards* RV5
Visible light source	*Kessil lamp* PR160L
**Instruments**	**Supplier**
Glovebox	*Vigor* SG1200/750TS-F
GC-MS<br>(Gas Chromatograph Mass Spectrometer)	*Agilent* 5977B

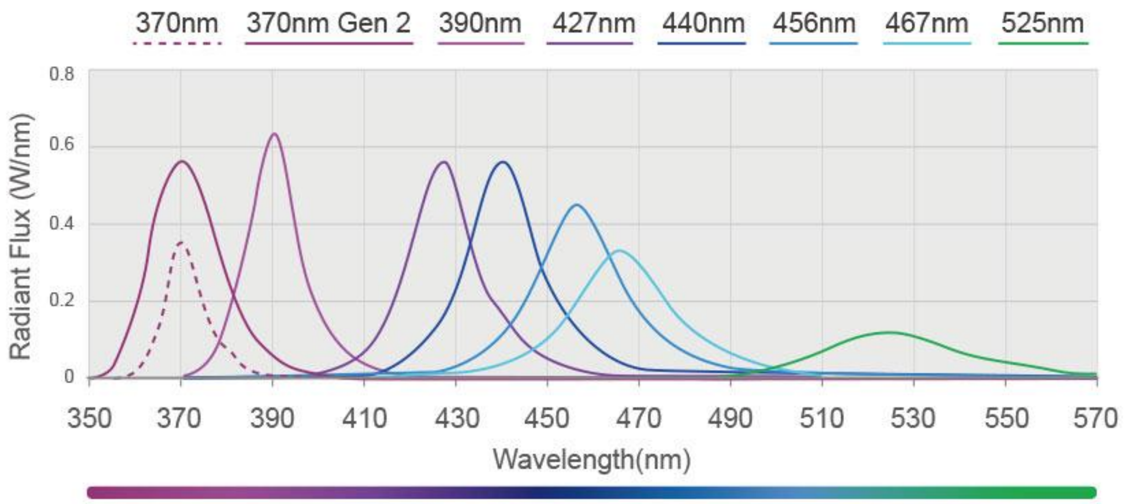

Figure S1 Emission spectra of the kessil lamp used within this study

1.4 Purchasing reagent

Table S2 Supplier of solvents

Solvent	Supplier	Solvent	Supplier
ACS Acetone	Duksan	HPLC Acetonitrile	J.T. Baker
ACS Acetonitrile	J.T. Baker	HPLC DMF	Macron
ACS Benzene	Echo	HPLC Ethyl acetate	Merck
ACS Chloroform	Acros	HPLC Hexane	Echo
ACS Dimethyl sulfoxide	UR	HPLC Isopropanol	Echo
ACS Diethyl ether	Duksan	ACS Hexane	Duksan
ACS Dichloromethane	Macron /Duksan	ACS Methanol	Macron
ACS Ethanol	J.T. Baker	ACS THF	Macron
ACS Ethyl acetate	Macron	ACS Toluene	Echo

Table S3 Supplier of solvents in solvent purification systems

Solvent	Supplier	Solvent	Supplier
Acetonitrile	J.T. Baker	Dichloromethane	Macron
DMF	Macron	THF	Macron
1,4-Dioxane	J.T. Baker	Toluene	J.T. Baker
Diethyl ether	Echo		

Table S4 Supplier of deuterated solvents

Solvent	Supplier	Solvent	Supplier
Acetonitrile-d_3	Sigma-Aldrich	DMSO-d_6	Sigma-Aldrich
Benzene-d_6	Sigma-Aldrich	Dichloromethane-d_2	Sigma-Aldrich
Chloroform-d_1	Merck	Methanol-d_4	Sigma-Aldrich

Back to contents

Table S5 Catalogue of commercial aldehyde reagents

Name	CAS Number	Supplier
Acetaldehyde	75-07-0	Riedel-de Haën
Isobutyraldehyde	78-84-2	Alfa Aesar
Pivalaldehyde	630-19-3	Alfa Aesar
Cyclobutanecarbaldehyde	2987-17-9	BLD
Cyclopentanecarbaldehyde	872-53-7	BLD
Cyclohexanecarbaldehyde	2043-61-0	Nova-Malts
Benzaldehyde	100-52-7	Alfa Aesar
4-Fluorobenzaldehyde	459-57-4	Sigma-Aldrich
4-Chlorobenzaldehyde	104-88-1	Acros
4-Bromobenzaldehyde	1122-91-4	Sigma-Aldrich
4-Cyanobenzaldehyde	105-07-7	Merck
Piperonal	120-57-0	Merck
4-Trifluoromethylbenzaldehyde	455-19-6	Combi-Blocks
[1,1'-Biphenyl]-4-carbaldehyde	3218-36-8	BLD
4-Methyl-benzaldehyde	104-87-0	BLD
4-Hydroxybenzaldehyde	123-08-0	Acros
4-Methoxybenzaldehyde	123-11-5	Alfa Aesar
o-Tolualdehyde	529-20-4	Alfa Aesar
3-Methoxybenzaldehyde	591-31-1	Sigma-Aldrich
Thiophene-2-carbaldehyde	98-03-3	Alfa Aesar
N-Methylpyrrole aldehyde	1192-58-1	Alfa Aesar
4-Nitrobenzaldehyde	555-16-8	BLD
4-(Dimethylamino)benzaldehyde	100-10-7	Acros

Back to contents

Table S6 Catalogue of commercial ketone reagents

Name	CAS Number	Supplier
1-Acetyladamantane	1660-04-4	Bidepharm
Benzil	134-81-6	Acros
4-Benzylidene-2,6-di-*tert*-butylcyclohexa-2,5-dien-1-one	7078-98-0	Nova
1,2-Bis(4-methoxyphenyl)ethane-1,2-dione	1226-42-2	BLD
Cyclopentyl methyl ketone	6004-60-0	BLD
1-(2,3-Dihydro-1*H*-inden-2-yl)ethan-1-one	33982-85-3	BLD
1,1-Dimethoxypropan-2-one	6342-56-9	Merck
3,4-Hexanedione	4437-51-8	Bidepharm
1,3-Indanedione	606-23-5	Nova
Isopropyl phenyl ketone	611-70-1	BLD
Phenyl cyclohexyl ketone	712-50-5	TCI
1-(Tetrahydro-2*H*-pyran-4-yl)ethenone	137052-08-5	BLD

Table S7 Catalogue of other commercial reagents

Name	CAS Number	Supplier
Acetic acid	64-19-7	Fluka
Acetic anhydride	108-24-7	Nihon Shiyaku
Acetylacetone	123-54-6	Alfa Aesar
2-Aminobenzamide	88-68-6	Nova-Malts
4-Bromchinolin	3964-04-3	Nova-Matls
tert-Butylhydroperoxid	75-91-2	TCI
Diethyl malonate	105-53-3	Janssen
Diphenyl diselenide	1666-13-3	Alfa Aesar
2,6-Di-*tert*-butylphenol	128-39-2	Nova
Di-*tert*-butyl azodicarboxylate	870-50-8	Nova-Matls
Ethyl cyanoacetate	105-56-6	Alfa Aesar
Iodine	7553-56-2	TCI
Lithium bromide	7550-35-8	Alfa Aesar

Malononitrile	109-77-3	Sigma-Aldrich
L-Menthol	2216-51-5	Acros
Phenylpropiolic acid	637-44-5	Nova
Piperidine	110-89-4	Alfa Aesar
L-Proline	147-85-3	Merck
Styrene	100-42-5	Alfa Aesar
Sodium benzenesulphinate	873-55-2	Nova
Sodium acetate	127-09-3	Sigma Aldrich

2. Experimental procedure

2.1. Preparation of dihydroquinazolinones (DHQZs)

Table S8 Scope of radical precursor 1

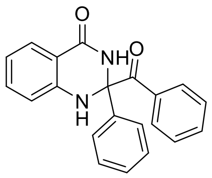 **1a**	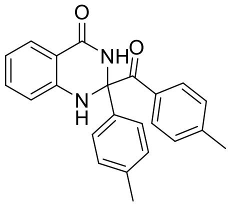 **1b**	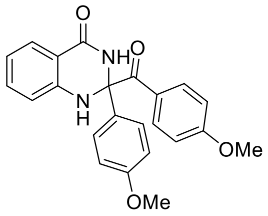 **1c**	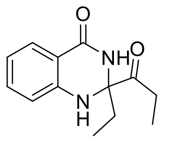 **1d**
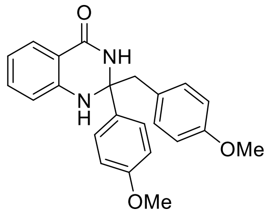 **1e**	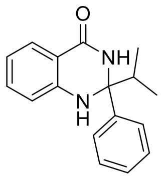 **1f**	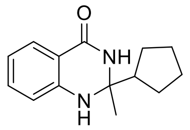 **1g**	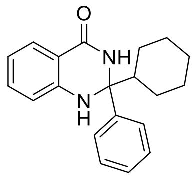 **1h**
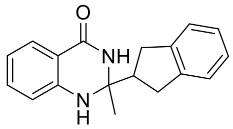 **1i**	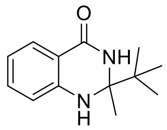 **1j**	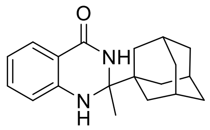 **1k**	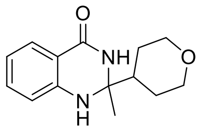 **1l**
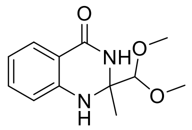 **1m**			

General procedure A

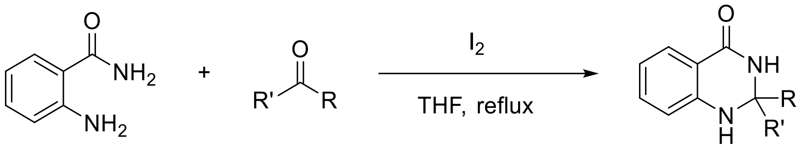

S1 **1**

According to the literature procedure,[1] an oven-dried 50 mL round bottom flask was added with **S1** (2-aminobenzamide, 1.4 g, 10.0 mmol, 1.0 equiv), ketone (10.5 mmol, 1.05 equiv) and iodine (0.1 g, 0.05 mmol, 0.5 mol%) in anhydrous THF (15 mL). The reaction mixture was stirred at 75 °C for 48 hr. Upon completion (monitored by TLC), the reaction was cooled to room temperature (monitored by TLC). Water (50 mL) was added to the mixture and then extracted by ethyl acetate. The organic layer was dried with $MgSO_4$, then concentrated under reduced pressure and purified by recrystallization in hexane/ethyl acetate to afford the corresponding compound **1**.

2-Benzoyl-2-phenyl-2,3-dihydroquinazolin-4(1*H*)-one, 1a

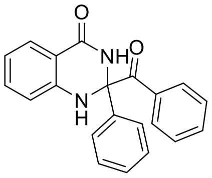

^{1}H NMR (300 MHz, $CDCl_3$) δ 7.85 (dd, J = 7.8, 1.6 Hz, 1H), 7.59 – 7.56 (m, 2H), 7.52 – 7.43 (m, 5H), 7.35 – 7.29 (m, 3H), 6.88 – 6.83 (m, 1H), 6.73 – 6.70 (m, 2H), 5.33 (br, 1H) ppm.

See NMR spectra

The spectra data are consistent with the reported literature.[1]

2-(4-Methylbenzoyl)-2-(*p*-tolyl)-2,3-dihydroquinazolin-4(1*H*)-one, 1b

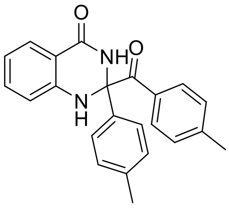

^{1}H NMR (300 MHz, DMSO-d_6) δ 8.76 (s, 1H), 7.72 – 7.53 (m, 4H), 7.39 (d, J = 8.2 Hz, 2H), 7.21 (dd, J = 18.4, 8.0 Hz, 5H), 6.85 (d, J = 8.0 Hz, 1H), 6.68 (t, J = 7.8 Hz, 1H), 2.31 (d, J = 5.4 Hz, 6H) ppm.

See NMR spectra

The spectra data are consistent with the reported literature.[1]

2-(4-Methoxybenzoyl)-2-(4-methoxyphenyl)-2,3-dihydroquinazolin-4(1*H*)-one, 1c

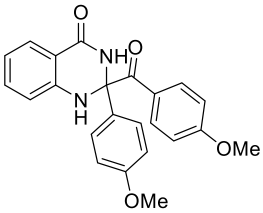

The compound **1c** was afforded as white solid (3.1 g, 80%).**^{1}H NMR** (400 MHz, $CDCl_3$) δ 7.85 (d, J = 7.8 Hz, 1H), 7.59 (d, J = 8.9 Hz, 2H), 7.47 (d, J = 8.8 Hz, 2H), 7.31 (t, J = 7.7 Hz, 1H), 6.92 (d, J = 8.8 Hz, 2H), 6.86 – 6.80 (m, 3H), 6.72 (d, J = 8.1 Hz, 1H), 6.65 – 6.58 (m, 1H), 5.29 (s, 1H), 3.81 (s, 3H), 3.78 (m,3H) ppm.

^{13}C NMR (101 MHz, $CDCl_3$) δ 196.2, 163.5, 160.4, 134.4, 131.9, 129.5, 128.4, 127.1, 126.0, 119.7, 115.3, 114.8, 114.2, 55.4 ppm.

See NMR spectra

Melting point: 161-165 ℃

HRMS (m/z): (ESI) calc'd for $C_{23}H_{20}O_4N_2{}^{23}Na$ $[M+Na]^+$: 411.1315, found: 411.1312

2-Ethyl-2-propionyl-2,3-dihydroquinazolin-4(*1H*)-one, 1d

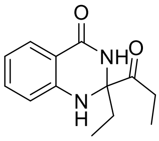

^{1}H NMR (300 MHz, $CDCl_3$) δ 7.83 (dd, J = 7.8, 1.6 Hz, 1H), 7.67 – 7.64 (m, 1H), 7.34 – 7.28 (m, 1H), 6.85 – 6.80 (m, 1H), 6.73 (dd, J = 8.1, 1.1 Hz, 1H), 5.00 (br, 1H), 2.77 – 2.54 (m, 2H), 1.96 – 1.73 (m, 2H), 1.07 – 0.98 (m, 6H) ppm.

See NMR spectra

The spectra data are consistent with the reported literature.[1]

Back to contents/Back to table

2-(4-Methoxybenzyl)-2-(4-methoxyphenyl)-2,3-dihydroquinazolin-4(1H)-one, 1e

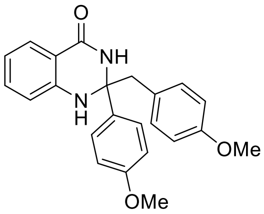

^{1}H NMR (300 MHz, DMSO-d_6) δ 8.60 (s, 1H), 7.52 (d, J = 8.5 Hz, 2H), 7.41 – 7.35 (m, 4H), 7.15 (t, J = 8.4 Hz, 1H), 6.90 – 6.83 (m, 4H), 6.75 (d, J = 8.4 Hz, 1H), 6.50 (t, J = 7.2 Hz, 1H), 3.71 (s, 6H), 2.95 (s, 2H) ppm. See NMR spectra

The spectra data are consistent with the reported literature.[1]

2-Isopropyl-2-phenyl-2,3-dihydroquinazolin-4(1*H*)-one, 1f

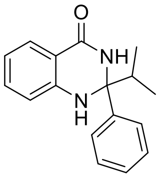

^{1}H NMR (300 MHz, DMSO-d_6) δ 8.54 (s, 1H), 7.45 – 7.41 (m, 3H), 7.30 – 7.14 (m, 5H), 6.92 (d, J = 8.1 Hz, 1H), 6.54 (t, J = 7.5 Hz, 1H), 2.11 (p, J = 6.8 Hz, 1H), 0.93 (d, J = 6.8 Hz, 3H), 0.85 (d, J = 6.9 Hz, 3H) ppm.

See NMR spectra

The spectra data are consistent with the reported literature.[1]

2-Cyclopentyl-2-methyl-2,3-dihydroquinazolin-4(1*H*)-one, 1g

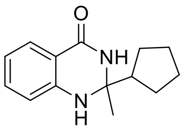

^{1}H NMR (400 MHz, $CDCl_3$) δ 7.84 (d, J = 7.8 Hz, 1H), 7.29 – 7.26 (m, 1H), 6.77 (t, J = 7.5 Hz, 1H), 6.59 (d, J = 8.1 Hz, 1H), 6.09 (s, 1H), 2.29 (p, J = 8.7 Hz, 1H), 1.81 – 1.40 (m, 11H) ppm.

See NMR spectra

The spectra data are consistent with the reported literature.[2]

2-Cyclohexyl-2-phenyl-2,3-dihydroquinazolin-4(1*H*)-one, 1h

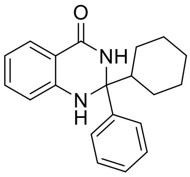

^{1}H NMR (300 MHz, $CDCl_3$) δ 7.80 – 7.77 (m, 1H), 7.36 – 7.33 (m, 2H), 7.30 – 7.17 (m, 4H), 6.75 – 6.67 (dd, J = 17.3, 8.1 Hz, 2H), 1.81 – 1.56 (m, 7H), 1.24 – 1.06 (m, 5H) ppm.

See NMR spectra

The spectra data are consistent with the reported literature.[1]

2-Isopropyl-2-phenyl-2,3-dihydroquinazolin-4(1*H*)-one, 1i

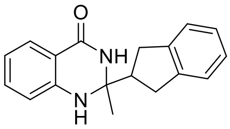

^{1}H NMR (300 MHz, DMSO-d_6) δ 8.03 (s, 2H), 7.56 (d, J = 6.2 Hz, 1H), 7.23 – 7.15 (m, 3H), 7.12 – 7.07 (m, 2H), 6.67 – 6.66 (m, 2H), 6.61 – 6.56 (m, 1H), 2.98 (dd, J = 18.4, 8.9 Hz, 2H), 2.88 – 2.79 (m, 3H), 1.37 (s, 3H) ppm.

See NMR spectra

The spectra data are consistent with the reported literature.[2]

Back to contents/Back to table

2-(*tert*-Butyl)-2-methyl-2,3-dihydroquinazolin-4(*1H*)-one, 1j

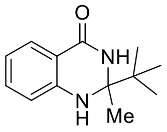

The compound **1j** was afforded as white solid (0.904 g, 41%). **^{1}H NMR** (400 MHz, $CDCl_3$) δ 7.84 (dd, J = 7.8, 1.6 Hz, 1H), 7.30 – 7.23 (m, 1H), 6.75 (td, J = 7.5, 1.0 Hz, 1H), 6.57 (dd, J = 8.1, 1.0 Hz, 1H), 5.89 (br, 1H), 4.14 (br, 1H), 1.49 (s, 3H), 1.06 (s, 9H) ppm.

See NMR spectra

Melting point:178-184℃

HRMS (m/z): (ESI) calc'd for $C_{13}H_{18}ON_2{}^{23}Na$ $[M+Na]^+$: 241.1311, found: 241.1310

2-((1*R*,3*S*,5*r*,7*r*)-Adamantan-2-yl)-2-methyl-2,3-dihydroquinazolin-4(1*H*)-one, 1k

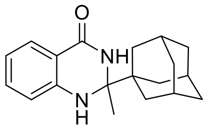

^{1}H NMR (400 MHz, $CDCl_3$) δ 7.83 (dd, J = 7.8, 1.6 Hz, 1H), 7.28 – 7.24 (m, 1H), 6.78 – 6.68 (m, 1H), 6.55 (dd, J = 8.1, 1.1 Hz, 1H), 5.75 (s, 1H), 4.15 – 4.10 (m, 1H), 2.06 (d, J = 8.9 Hz, 3H), 1.77 – 1.58 (m, 12H), 1.45 (s, 3H) ppm. See NMR spectra

The spectra data are consistent with the reported literature.[1]

2-Methyl-2-(tetrahydro-2*H*-pyran-4-yl)-2,3-dihydroquinazolin-4(1*H*)-one, 1l

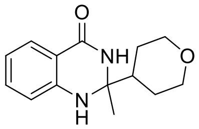

^{1}H NMR (300 MHz, DMSO-d_6) δ 7.93 (s, 1H), 7.53 (d, J = 7.6 Hz, 1H), 7.21 – 7.15 (m, 2H), 6.67 – 6.63 (m, 3H), 6.56 (t, J = 7.4 Hz, 1H), 3.91 – 3.84 (m, 3H), 3.15 (q, J = 11.0 Hz, 3H), 1.82 – 1.74 (t, J = 11.9 Hz, 1H), 1.55 (t, J = 12.4 Hz, 2H), 1.42 – 1.34 (m, 3H), 1.31 (s, 3H) ppm.

See NMR spectra

The spectra data are consistent with the reported literature.[2]

2-(Dimethoxymethyl)-2-methyl-2,3-dihydroquinazolin-4(1*H*)-one, 1m

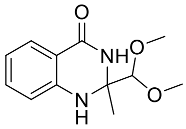

^{1}H NMR (400 MHz, $CDCl_3$) δ 7.86 (d, J = 7.8 Hz, 1H), 7.31 – 7.27 (m, 1H), 6.81 (t, J = 7.5 Hz, 1H), 6.61 (d, J = 8.1 Hz, 1H), 5.96 (s, 1H), 4.45 (s, 1H), 4.34 (s, 1H), 3.57 (s, 3H), 3.54 (s, 3H), 1.41 (s, 4H) ppm.

^{13}C NMR (101 MHz, $CDCl_3$) δ 163.6, 145.5, 134.2, 128.3, 118.7, 114.2, 114.1, 108.2, 71.3, 59.1, 58.6, 20.9 ppm.

See NMR spectra

The spectra data are consistent with the reported literature.[23]

2.2. Preparation of Radical traps

Table S9 Scopes of Compound 2

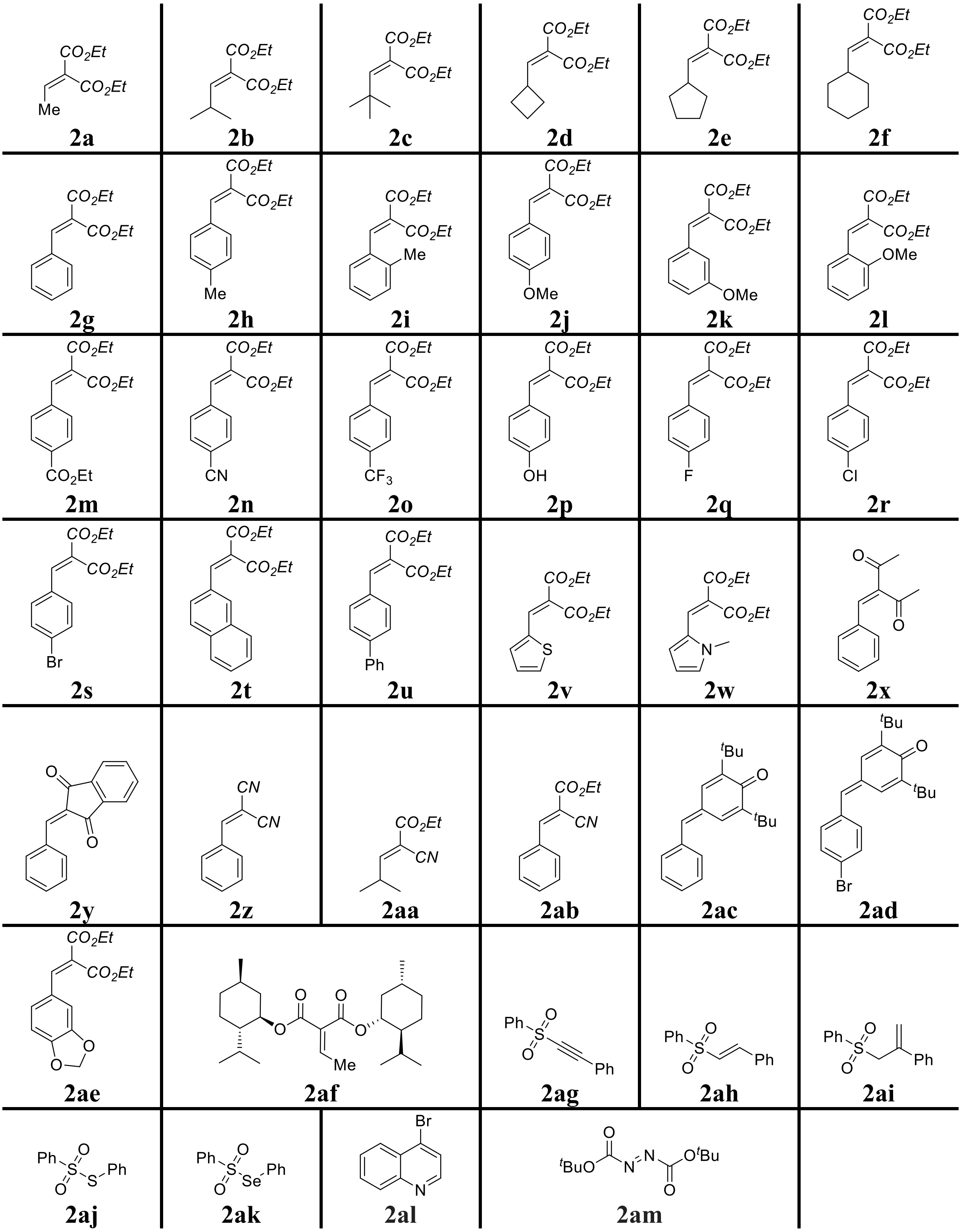

General procedure B

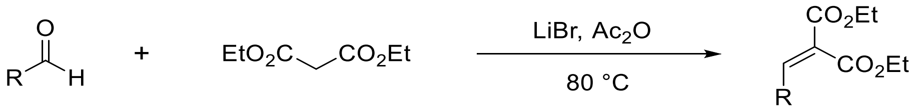

According to the literature procedure,[3] an oven-dried sealed tube containing a stirring bar was added Ac_2O (1.88 mL, 20.0 mmol, 2.0 equiv), anhydrous LiBr (170 mg, 2.0 mmol, 0.2 equiv) and malonate (1.53 mL, 10.0 mmol, 1.0 equiv). After the resulting mixture was stirred at 80 °C for 3 hr under nitrogen, aldehyde was added in one portion into the tube. The solution was stirred at 80 °C further for 4 hr. After monitoring by TLC, the solution was then cooled to room temperature and quenched with a saturated aqueous solution of Na_2CO_3 (25 mL). The reaction mixture was extracted with Et_2O (2 × 30 mL), and then the combined organic phases were washed with brine and dried over $MgSO_4$. After removing Et_2O by rotary evaporation, the residue was purified by flash chromatography using hexane/ethyl acetate (25:1, v/v) as the eluent to obtain the desired compound.

General procedure C

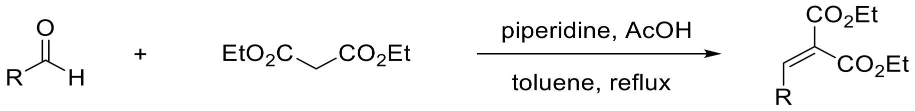

According to the literature procedure,[4] an oven-dried flask containing a stirring bar was added aldehyde, diethyl malonate, piperidine, and toluene (0.1 M). Acetic acid was added dropwise to the mixture. The resulting mixture was refluxed for several hr in an apparatus with a Dean-Stark trap to remove water. After the reaction was completed, the solution was then concentrated under reduced pressure. The residue was taken into ethyl acetate (6 mL), which was washed successively with sat. $NaHCO_3$(3 × 5 mL), 3.0 M HCl (3 × 5 mL), and water (3 × 5 mL), and then dried over $MgSO_4$. After removing ethyl acetate by rotary evaporation, the residue was purified by flash chromatography using hexane/ethyl acetate as the eluent to obtain the desired compound.

General procedure D

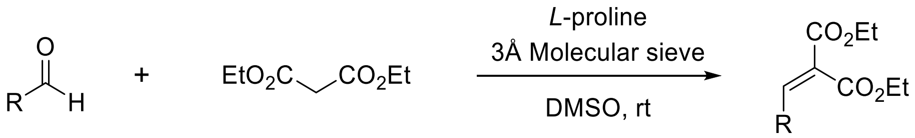

According to the literature procedure,[5] an oven-dried flask containing a stirring bar was taken into the glovebox where the 3Å molecular sieve (2.0 g) was added. After being taken out of the glove box, the flask with molecular sieve was charged with a solution of *L*-proline (0.16 equiv) in DMSO (2.0 M) and further added with the aldehyde. The reaction mixture was stirred overnight, then extracted with ethyl acetate and washed twice with water. The organic layer was dried over $MgSO_4$, and the solvent was removed in vacuo. After removing ethyl acetate by rotary evaporation, the residue was purified by flash chromatography using hexane/ethyl acetate as the eluent to obtain the desired compound.

Diethyl 2-(2-methylpropylidene)malonate (2b)

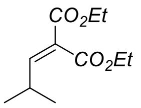

According to **General procedure D**, isobutyraldehyde (0.91 mL, 10.0 mmol, 1.0 equiv), diethyl malonate (1.68 mL, 11.0 mmol, 1.1 equiv) and *L*-proline (0.18 g, 1.6 mmol, 0.16 equiv) were used to obtain a crude residue, which was purified by silica gel column chromatography (hexane/ethyl acetate 25:1, v/v) to afford the corresponding product.

^{1}H NMR (300 MHz, $CDCl_3$) δ 6.78 (d, J = 10.5 Hz, 1H), 4.30 (q, J = 7.1 Hz, 2H), 4.23 (q, J = 7.1 Hz, 2H), 2.75 – 2.63 (m, 1H), 1.32 (t, J = 7.1 Hz, 3H), 1.29 (t, J = 7.1 Hz, 3H), 1.07 (d, J = 6.6 Hz, 6H) ppm.

See NMR spectra

All other data matches what was reported in the literature.[6]

Diethyl 2-(2,2-dimethylpropylidene)malonate (2c)

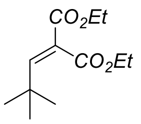

According to **General procedure D**, pivalaldehyde (861.3 mg, 10.0 mmol, 1.0 equiv), diethyl malonate (1.68 mL, 11.0 mmol, 1.1 equiv) and *L*-proline (0.18 g, 1.6 mmol, 0.16 equiv) were used to obtain a crude residue, which was purified by silica gel column chromatography (hexane/ethyl acetate 25:1, v/v) to afford **2c** as colorless liquid (958.4 mg, 4.2 mmol, 42%).

^{1}H NMR (300 MHz, $CDCl_3$) δ 6.87 (s, 1H), 4.31 – 4.17 (m, 4H), 1.35 – 1.24 (m, 6H), 1.13 (s, 9H) ppm.

See NMR spectra

HRMS (m/z): (ESI) calc'd for $C_{12}H_{20}O_4{}^{23}Na$ $[M+Na]^+$: 251.1254, found: 251.1252

Diethyl 2-(cyclobutylmethylene)malonate (2d)

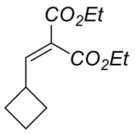

According to **General procedure D**, cyclobutanecarbaldehyde (0.54 mL, 6.0 mmol, 1.2 equiv), diethyl malonate (0.76 mL, 5.0 mmol, 1.0 equiv) and *L*-proline (0.09 g, 0.8 mmol, 0.16 equiv) were used to obtain a crude residue, which was purified by silica gel column chromatography (hexane/ethyl acetate 25:1, v/v) to afford **2d** as colorless liquid (680.0 mg, 3.0 mmol, 60%).

^{1}H NMR (300 MHz, $CDCl_3$) δ 7.08 (d, J = 9.3 Hz, 1H), 4.32 – 4.19 (m, 4H), 3.37 – 3.25 (m, 1H), 2.28 – 2.20 (m, 2H), 2.06 – 1.86 (m, 4H), 1.32 (t, J = 7.1 Hz, 3H), 1.29 (t, J = 7.1 Hz, 3H) ppm.

^{13}C{^{1}H} NMR (101 MHz, $CDCl_3$) δ 165.5, 164.1, 153.0, 126.3, 61.13, 61.12, 35.3, 28.4, 18.9, 14.1, 14.0 ppm.

See NMR spectrum

HRMS (m/z): (ESI) calc'd for $C_{12}H_{18}O_4{}^{23}Na$ $[M+Na]^+$: 249.1097, found: 249.1097

Diethyl 2-(cyclopentylmethylene)malonate (2e)

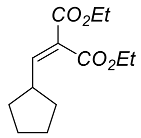

According to **General procedure C**, cyclopentanecarbaldehyde (1,078 mg, 11.0 mmol, 1.1 equiv), diethyl malonate (1.53 mL, 10.0 mmol, 1.0 equiv), acetic acid (0.2 mL, 3 mmol, 0.3 equiv) and piperidine (0.2 mL, 2.0 mmol, 0.2 equiv) were used to obtain crude residue, which was purified by silica gel column chromatography (hexane/ethyl acetate 25:1, v/v) to afford **2e** as colorless liquid (700.0 mg, 2.9 mmol, 29%).

^{1}H NMR (300 MHz, $CDCl_3$) δ 6.89 (d, J = 10.5 Hz, 1H), 4.33 – 4.19 (m, 4H), 2.85 – 2.71 (m, 1H), 1.94 – 1.84 (m, 2H), 1.72 – 1.59 (m, 4H), 1.45 – 1.40 (m, 2H), 1.34 – 1.26 (m, 6H) ppm.

See NMR spectrum

HRMS (m/z): (ESI) calc'd for $C_{13}H_{20}O_4{}^{23}Na$ $[M+Na]^+$: 263.1254, found: 263.1252

Diethyl 2-(cyclohexylmethylene)malonate (2f)

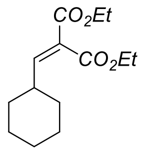

According to **General procedure C**, cyclohexanecarbaldehyde (0.99 mL, 11.0 mmol, 1.1 equiv), diethyl malonate (1.53 mL, 10.0 mmol, 1.0 equiv), acetic acid (0.2 mL, 3 mmol, 0.3 equiv) and piperidine (0.2 mL, 2.0 mmol, 0.2 equiv) were used to obtain crude residue, which was purified by silica gel column chromatography (hexane/ethyl acetate 30:1, v/v) to afford the corresponding product.

^{1}H NMR (300 MHz, $CDCl_3$) δ 6.80 (d, J = 10.4 Hz, 1H), 4.30 (q, J = 7.1 Hz, 2H), 4.23 (q, J = 7.1 Hz, 2H), 2.44 – 2.34 (m, 1H), 1.75 – 1.65 (m, 5H), 1.33 (t, J = 7.1 Hz, 3H), 1.29 (t, J = 7.1 Hz, 3H), 1.26 – 1.11 (m, 5H) ppm.

See NMR spectrum

All other data matches what was reported in the literature.[6]

Diethyl 2-(4-methylbenzylidene)malonate (2h)

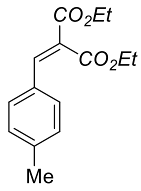

According to **General procedure C**, 4-Methylbenzaldehyde (1.04 mL, 8.8 mmol, 1.1 equiv), diethyl malonate (1.22 mL, 8.0 mmol, 1.0 equiv), acetic acid (0.05 mL, 0.8 mmol, 0.1 equiv) and piperidine (0.08 mL, 0.8 mmol, 0.1 equiv) were used to obtain crude residue, which was purified by silica gel column chromatography (hexane/ethyl acetate 30:1, v/v) to afford the corresponding product.

^{1}H NMR (300 MHz, $CDCl_3$) δ 7.70 (s, 1H), 7.35 (d, J = 8.1 Hz, 2H), 7.18 (d, J = 8.0 Hz, 2H), 4.38 – 4.26 (m, 4H), 2.37 (s, 3H), 1.35 – 1.28 (m, 6H) ppm.

See NMR spectrum

All other data matches what was reported in the literature.[6]

Diethyl 2-(2-methylbenzylidene)malonate (2i)

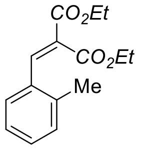

According to **General procedure C**, 2-methylbenzaldehyde (1.27 mL, 11.0 mmol, 1.1 equiv), diethyl malonate (1.53 mL, 10.0 mmol, 1.0 equiv), acetic acid (0.06 mL, 1.0 mmol, 0.1 equiv) and piperidine (0.10 mL, 1.0 mmol, 0.1 equiv) were used to obtain crude residue, which was purified by silica gel column chromatography (hexane/ethyl acetate 25:1, v/v) to afford the corresponding product.

^{1}H NMR (300 MHz, $CDCl_3$) δ 7.97 (s, 1H), 7.34 – 7.25 (m, 2H), 7.21 – 7.12 (m, 2H), 4.32 (q, J = 7.1 Hz, 2H), 4.22 (q, J = 7.1 Hz, 2H), 2.38 (s, 3H), 1.34 (t, J = 7.1 Hz, 3H), 1.16 (t, J = 7.1 Hz, 3H) ppm.

See NMR spectrum

All other data matches what was reported in the literature.[6]

Diethyl 2-(4-methoxybenzylidene)malonate (2j)

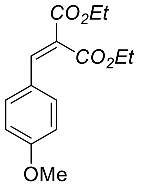

According to **General procedure C**, 4-methoxybenzaldehyde (1.46 mL, 12.0 mmol, 1.0 equiv), diethyl malonate (2.01 mL, 13.2 mmol, 1.1 equiv), acetic acid (0.07 mL, 1.2 mmol, 0.1 equiv) and piperidine (0.12 mL, 1.2 mmol, 0.1 equiv) were used to obtain crude residue for 12 h heating, which was purified by silica gel column chromatography (hexane/ethyl acetate 15:1, v/v) to afford the corresponding product.

^{1}H NMR (300 MHz, $CDCl_3$) δ 7.67 (s, 1H), 7.42 (d, J = 8.6 Hz, 2H), 6.89 (d, J = 8.9 Hz, 2H), 4.35 (q, J = 7.1 Hz, 2H), 4.29 (q, J = 7.1 Hz, 2H), 3.83 (s, 3H), 1.32 (t, J = 7.1 Hz, 3H), 1.32 (t, J = 7.1 Hz, 3H) ppm.

See NMR spectrum

All other data matches what was reported in the literature.[6]

Diethyl 2-(3-methoxybenzylidene)malonate (2k)

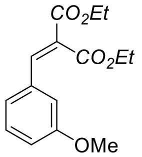

According to **General procedure D**, 3-methoxybenzaldehyde (1.17 mL, 9.6 mmol, 1.2 equiv), diethyl malonate (1.22 mL, 8.0 mmol, 1.0 equiv) and *L*-proline (0.15 g, 1.28 mmol, 0.16 equiv) were used to obtain crude residue, which was purified by silica gel column chromatography (hexane/ethyl acetate 25:1, v/v) to afford the corresponding product.

^{1}H NMR (300 MHz, $CDCl_3$) δ 7.70 (s, 1H), 7.31 – 7.28 (m, 1H), 7.06 – 7.03 (m, 1H), 7.00 – 6.92 (m, 2H), 4.37 – 4.27 (m, 4H), 3.80 (s, 3H), 1.33 (t, J = 7.1 Hz, 3H), 1.29 (t, J = 7.1 Hz, 3H) ppm.

See NMR spectrum

All other data matches what was reported in the literature.[8]

Diethyl 2-(2-methoxybenzylidene)malonate (2l)

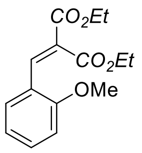

According to **General procedure D**, 2-methoxybenzaldehyde (1.31 g, 9.6 mmol, 1.2 equiv), diethyl malonate (1.22 mL, 8.0 mmol, 1.0 equiv) and *L*-proline (0.15 g, 1.28 mmol, 0.16 equiv) were used to obtain crude residue, which was purified by silica gel column chromatography (hexane/ethyl acetate 25:1, v/v) to afford the corresponding product.

^{1}H NMR (300 MHz, $CDCl_3$) δ 8.08 (s, 1H), 7.39 – 7.33 (m, 2H), 6.93 – 6.88 (m, 2H), 4.33 – 4.23 (m, 4H), 3.85 (s, 3H), 1.33 (t, J = 7.2 Hz, 3H), 1.23 (t, J = 7.2 Hz, 3H) ppm.

See NMR spectrum

All other data matches what was reported in the literature.[8]

Diethyl 2-(4-(ethoxycarbonyl)benzylidene)malonate (2m)

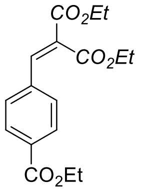

According to **General procedure C**, ethyl 4-formylbenzoate (1.70 g, 9.6 mmol, 1.2 equiv), diethyl malonate (1.22 mL, 8.0 mmol, 1.0 equiv) and piperidine (0.08 mL, 0.8 mmol, 0.1 equiv) were used to obtain crude residue, which was purified by silica gel column chromatography (hexane/ethyl acetate 15:1, v/v) to afford **2m** as colorless liquid (1024.9 mg, 3.2 mmol, 40%).

^{1}H NMR (300 MHz, $CDCl_3$) δ 8.04 (d, J = 8.3, 2H), 7.75 (s, 1H), 7.51 (J = 8.2 Hz, 2H), 4.42 – 4.28 (m, 6H), 1.42 – 1.25 (m, 9H) ppm.

See NMR spectrum

HRMS (m/z): (ESI) calc'd for $C_{17}H_{20}O_6{}^{23}Na$ $[M+Na]^+$: 343.1152, found: 343.1150

Diethyl 2-(4-cyanobenzylidene)malonate (2n)

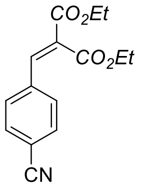

According to **General procedure C**, 4-formylbenzonitrile (1.26 g, 9.6 mmol, 1.2 equiv), diethyl malonate (1.22 mL, 8.0 mmol, 1.0 equiv) and piperidine (0.08 mL, 0.8 mmol, 0.1 equiv) were used to obtain crude residue for, which was purified by silica gel column chromatography (hexane/ethyl acetate 15:1, v/v) to afford the corresponding product.

^{1}H NMR (300 MHz, $CDCl_3$) δ 7.71 (s, 1H), 7.68 – 7.66 (m, 2H), 7.55 – 7.53 (m, 2H), 4.33 (q, J = 7.1 Hz, 2H), 4.32 (q, J = 7.1 Hz, 2H), 1.34 (t, J = 7.1 Hz, 3H), 1.27 (t, J = 7.1 Hz, 3H) ppm.

See NMR spectrum

All other data matches what was reported in the literature.[6]

Diethyl 2-(4-(trifluoromethyl)benzylidene)malonate (2o)

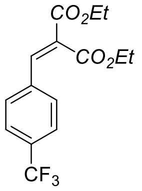

According to **General procedure C**, 4-(trifluoromethyl)benzaldehyde (0.82 mL, 6.0 mmol, 1.0 equiv), diethyl malonate (0.92 mL, 6.0 mmol, 1.0 equiv), acetic acid (0.03 mL, 0.6 mmol, 0.1 equiv) and piperidine (0.06 mL, 0.6 mmol, 0.1 equiv) were used to obtain crude residue, which was purified by silica gel column chromatography (hexane/ethyl acetate 25:1, v/v) to afford the corresponding product.

^{1}H NMR (300 MHz, $CDCl_3$) δ 7.74 (s, 1H), 7.65 – 7.63 (m, 2H), 7.57 – 7.54 (m, 2H), 4.33 (q, J = 7.1 Hz, 4H), 1.34 (t, J = 7.1 Hz, 3H), 1.28 (t, J = 7.1 Hz, 3H) ppm.

See NMR spectrum

All other data matches what was reported in the literature.[9]

Diethyl 2-(4-hydroxybenzylidene)malonate (2p)

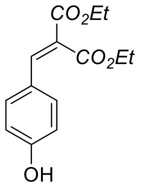

According to **General procedure C**, 4-hydroxybenzaldehyde (1.17 g, 9.6 mmol, 1.2 equiv), diethyl malonate (1.22 mL, 8.0 mmol, 1.0 equiv), acetic acid (0.46 mL, 8.0 mmol, 1.0 equiv) and piperidine (0.79 mL, 8.0 mmol, 1.0 equiv) were used to obtain crude residue, which was purified by silica gel column chromatography (hexane/ethyl acetate 10:1, v/v) to afford the corresponding product.

^{1}H NMR (300 MHz, $CDCl_3$) δ 7.65 (s, 1H), 7.35 (d, J = 8.6 Hz, 2H), 6.81 (d, J = 8.7 Hz, 2H), 4.35 (q, J = 7.1 Hz, 2H), 4.29 (q, J = 7.1 Hz, 2H), 1.32 (t, J = 7.1 Hz, 3H), 1.32 (t, J = 7.1 Hz, 3H) ppm.

See NMR spectrum

All other data matches what was reported in the literature.[10]

Diethyl 2-(4-fluorobenzylidene)malonate (2q)

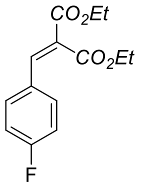

According to **General procedure D**, 4-fluorobenzaldehyde (1.18 mL, 11.0 mmol, 1.1 equiv), diethyl malonate (1.53 mL, 10.0 mmol, 1.0 equiv) and *L*-proline (0.18 g, 1.6 mmol, 0.16 equiv) were used to obtain crude residue, which was purified by silica gel column chromatography (hexane/ethyl acetate 25:1, v/v) to afford the corresponding product.

^{1}H NMR (300 MHz, $CDCl_3$) δ 7.68 (s, 1H), 7.48 – 7.43 (m, 2H), 7.10 – 7.04 (m, 2H), 4.34 (q, J = 7.1 Hz, 2H), 4.30 (q, J = 7.1 Hz, 2H), 1.33 (t, J = 7.1 Hz, 3H), 1.30 (t, J = 7.1 Hz, 3H) ppm.

See NMR spectrum

All other data matches what was reported in the literature.[6]

Diethyl 2-(4-chlorobenzylidene)malonate (2r)

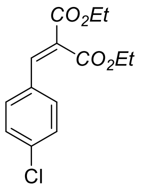

According to **General procedure C**, 4-chlorobenzaldehyde (1.46 g, 10.4 mmol, 1.3 equiv), diethyl malonate (1.22 mL, 8.0 mmol, 1.0 equiv), acetic acid (0.05 mL, 0.8 mmol, 0.1 equiv) and piperidine (0.08 mL, 0.8 mmol, 0.1 equiv) was used to obtain crude residue, which were purified by silica gel column chromatography (hexane/ethyl acetate 30:1, v/v) to afford the corresponding product.

^{1}H NMR (300 MHz, $CDCl_3$) δ 7.67 (s, 1H), 7.40 – 7.34 (m, 4H), 4.37 – 4.27 (m, 4H), 1.36 – 1.27 (m, 6H) ppm.

See NMR spectrum

All other data matches what was reported in the literature.[6]

Diethyl 2-(4-bromobenzylidene)malonate (2s)

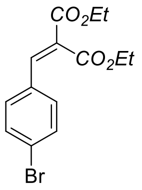

According to **General procedure D**, 4-bromobenzaldehyde (2.04 g, 11.0 mmol, 1.1 equiv), diethyl malonate (1.53 mL, 10.0 mmol, 1.0 equiv) and *L*-proline (0.18 g, 1.6 mmol, 0.16 equiv) were used to obtain crude residue, which was purified by silica gel column chromatography (hexane/ethyl acetate 25:1, v/v) to afford the corresponding product.

^{1}H NMR (300 MHz, $CDCl_3$) δ 7.65 (s, 1H), 7.53 – 7.50 (m, 2H), 7.33 – 7.30 (m, 2H), 4.36 – 4.27 (m, 4H), 1.33 (t, J = 7.1 Hz, 3H), 1.29 (t, J = 7.1 Hz, 3H) ppm.

See NMR spectrum

All other data matches what was reported in the literature.[7]

Diethyl 2-(naphthalen-2-ylmethylene)malonate (2t)

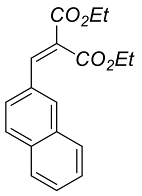

According to **General procedure D**, 2-naphthaldehyde (1.72 g, 11.0 mmol, 1.1 equiv), diethyl malonate (1.53 mL, 10.0 mmol, 1.0 equiv) and *L*-proline (0.18 g, 1.6 mmol, 0.16 equiv) were used to obtain crude residue, which was purified by silica gel column chromatography (hexane/ethyl acetate 25:1, v/v) to afford the corresponding product.

^{1}H NMR (300 MHz, $CDCl_3$) δ 7.97 (s, 1H), 7.90 (s, 1H), 7.85 – 7.80 (m, 3H), 7.57 – 7.48 (m, 3H), 4.41 – 4.30 (m, 4H), 1.36 (t, J = 7.1 Hz, 3H), 1.30 (t, J = 7.1 Hz, 3H) ppm.

See NMR spectrum

All other data matches what was reported in the literature.[9]

Diethyl 2-([1,1'-biphenyl]-4-ylmethylene)malonate (2u)

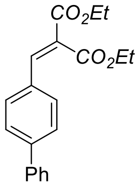

According to **General procedure C**, [1,1'-biphenyl]-4-carbaldehyde (1.46 g, 8.0 mmol, 1.0 equiv), diethyl malonate (1.34 mL, 8.8 mmol, 1.1 equiv), acetic acid (0.05 mL, 0.8 mmol, 0.1 equiv) and piperidine (0.08 mL, 0.8 mmol, 0.1 equiv) were used to obtain crude residue, which was purified by silica gel column chromatography (hexane/ethyl acetate 15:1, v/v) to afford the corresponding product.

^{1}H NMR (300 MHz, $CDCl_3$) δ 7.77 (s, 1H), 7.63 – 7.59 (m, 4H), 7.55 – 7.52 (m, 2H), 7.48 – 7.35 (m, 3H), 4.38 (q, J = 7.1 Hz, 2H), 4.32 (q, J = 7.1 Hz, 2H), 1.37 – 1.30 (m, 6H) ppm.

See NMR spectrum

All other data matches what was reported in the literature.[11]

Diethyl 2-(thiophen-2-ylmethylene)malonate (2v)

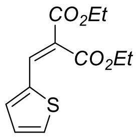

According to **General procedure C**, thiophene-2-carbaldehyde (0.84 mL, 9.0 mmol, 1.0 equiv), diethyl malonate (1.78 mL, 11.7 mmol, 1.3 equiv), acetic acid (0.05 mL, 0.9 mmol, 0.1 equiv) and piperidine (0.09 mL, 0.9 mmol, 0.1 equiv) were used to obtain crude residue, which was purified by silica gel column chromatography (hexane/ethyl acetate 15:1, v/v) to afford the corresponding product.

^{1}H NMR (300 MHz, $CDCl_3$) δ 7.84 (s, 1H), 7.52 (d, J = 5.1 Hz, 1H), 7.37 (d, J = 3.4 Hz, 1H), 7.10 – 7.07 (m, 1H), 4.41 (q, J = 7.1 Hz, 2H), 4.29 (q, J = 7.1 Hz, 2H), 1.38 (t, J = 7.1 Hz, 3H), 1.32 (t, J = 7.1 Hz, 3H) ppm.

See NMR spectrum

All other data matches what was reported in the literature.[12]

Diethyl 2-((1-methyl-1*H*-pyrrol-2-yl)methylene)malonate (2w)

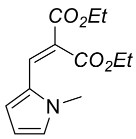

According to **General procedure C**, 1-methyl-1*H*-pyrrole-2-carbaldehyde (0.97 mL, 9.0 mmol, 1.0 equiv), diethyl malonate (1.78 mL, 11.7 mmol, 1.3 equiv), acetic acid (0.05 mL, 0.9 mmol, 0.1 equiv) and piperidine (0.09 mL, 0.9 mmol, 0.1 equiv) were used to obtain crude residue, which was purified by silica gel column chromatography (hexane/ethyl acetate 20:1, v/v) to afford **2w** as beige solid (1024.9 mg, 3.2 mmol, 40%). **^{1}H NMR** (300 MHz, $CDCl_3$) δ 7.61 (s, 1H), 6.80 (s, 1H), 6.65 – 6.63 (m, 1H), 6.20 – 6.18 (m, 1H), 4.37 (q, J = 7.1 Hz, 2H), 4.27 (q, J = 7.1 Hz, 2H), 3.72 (s, 3H), 1.38 – 1.28 (m, 6H) ppm.

See NMR spectrum

Melting point: 72-74℃

HRMS (m/z): (ESI) calc'd for $C_{13}H_{17}O_4N^{23}Na$ $[M+Na]^+$: 274.1050, found: 274.1050

3-Benzylidenepentane-2,4-dione (2x)

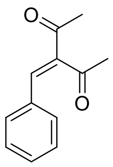

According to **General procedure B**, benzaldehyde (2.81 mL, 50.0 mmol, 5.0 equiv) was used to obtain a crude residue, which was purified by silica gel column chromatography (hexane/ethyl acetate 25:1, v/v) to afford the corresponding product.

^{1}H NMR (300 MHz, $CDCl_3$) δ 7.49 (s, 1H), 7.40 (s, 5H), 2.42 (s, 3H), 2.28 (s, 3H) ppm.

See NMR spectrum

All other data matches what was reported in the literature.[13]

2-Benzylidene-1*H*-indene-1,3(2*H*)-dione (2y)

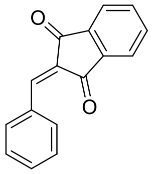

According to the literature procedure,[17] benzaldehyde (127 mg, 1.2 mmol) and 1,3-indanedione (146 mg, 1.0 mmol) were added into EtOH. The reaction mixture was refluxed for 3 hr in an oil bath. The crude was extracted, dried, and purified on a silica gel column chromatography (hexane/ethyl acetate 8:1, v/v) to provide **2y**.

^{1}H NMR (300 MHz, $CDCl_3$) δ 8.47 (dd, J = 7.8, 1.8 Hz, 2H), 8.04 – 7.99 (m, 2H), 7.92 (s, 1H), 7.83 (dd, J = 5.6, 3.0 Hz, 2H), 7.57 – 7.50 (m, 3H) ppm.

See NMR spectrum

All other data matches what was reported in the literature.[14]

2-Benzylidenemalononitrile (2z)

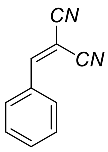

Benzaldehyde (2.81 mL, 50.0 mmol, 5.0 equiv), malononitrile (660 mg, 10.0 mmol, 1.0 equiv), and potassium carbonate (138 mg, 1.0 mmol, 0.1 equiv) were added in a mortar and ground rapidly with a pestle at room temperature for several minutes. Then, the mixture was washed with water, filtered, and dried under a high vacuum to afford the corresponding product.

^{1}H NMR (300 MHz, $CDCl_3$) δ 7.91 (d, J = 7.7 Hz, 2H), 7.78 (s, 1H), 7.66 – 7.61 (m, 1H), 7.57 – 7.52 (m, 2H) ppm.

See NMR spectrum

All other data matches what was reported in the literature.[6]

Ethyl (*E*)-2-cyano-4-methylpent-2-enoate (2aa)

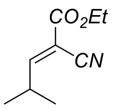

According to **General procedure D**, isobutyraldehyde (0.88 mL, 9.6 mmol, 1.2 equiv), ethyl 2-cyanoacetate (0.85 mL, 8.0 mmol, 1.0 equiv) and *L*-proline (0.15 g, 1.28 mmol, 0.16 equiv) were used to obtain crude residue, which was purified by silica gel column chromatography (hexane/ethyl acetate 25:1, v/v) to afford the corresponding product.

^{1}H NMR (300 MHz, $CDCl_3$) δ 7.46 (d, J = 10.6 Hz, 1H), 4.31 (q, J = 7.1 Hz, 2H), 3.06 – 2.94 (m, 1H), 1.35 (t, J = 7.1 Hz, 3H), 1.15 (d, J = 6.6 Hz, 6H) ppm.

See NMR spectrum

All other data matches what was reported in the literature.[15]

Ethyl (*E*)-2-cyano-3-phenylacrylate (2ab)

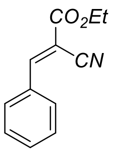

According to **General procedure C**, benzaldehyde (0.98 mL, 9.6 mmol, 1.2 equiv), ethyl 2-cyanoacetate (0.85 mL, 8.0 mmol, 1.0 equiv), acetic acid (0.05 mL, 0.8 mmol, 0.1 equiv) and piperidine (0.08 mL, 0.8 mmol, 0.1 equiv) were used to obtain crude residue, which was purified by silica gel column chromatography (hexane/ethyl acetate 15:1, v/v) to afford the corresponding product.

^{1}H NMR (300 MHz, $CDCl_3$) δ 8.25 (s, 1H), 7.99 (d, J = 7.6 Hz, 2H), 7.59 – 7.47 (m, 3H), 4.39 (q, J = 7.1 Hz, 2H), 1.40 (t, J = 7.1 Hz, 3H) ppm.

See NMR spectrum

All other data matches what was reported in the literature.[16]

4-(4-Bromobenzylidene)-2,6-di-*tert*-butylcyclohexa-2,5-dien-1-one (2ad)

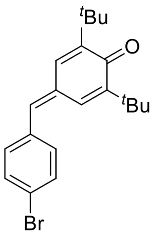

A flask was added 2,6-di tert-butylphenol (10 mmol, 1.0 equiv) and aldehyde (10 mmol, 1.0 equiv), and toluene (40 mL). The mixture was refluxed and stirred for 1 hour. Piperidine (20 mmol, 2.0 equiv) was then added dropwise added into the solution. The mixture was stirred further for 14 hr. After cooling to 100 °C, acetic anhydride (20 mmol, 2.0 equiv) was added. Stirring for 15 minutes further. Then, the crude mixture was extracted, dried, and purified through column chromatography using silica gel to obtain the desired products.

^{1}H NMR (400 MHz, $CDCl_3$) δ 7.60 – 7.57 (m, 2H), 7.43 (d, J = 2.1 Hz, 1H), 7.32 – 7.29 (m, 2H), 7.08 (s, 1H), 6.99 – 6.99 (m, 1H), 1.33 (s, 9H), 1.29 (s, 9H) ppm.

See NMR spectrum

All other data matches what was reported in the literature.[17]

Diethyl 2-(benzo[*d*][1,3]dioxol-5-ylmethylene)malonate (2ae)

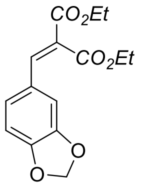

According to **General procedure C**, benzo[*d*][1,3]dioxole-5-carbaldehyde (1.32 g, 8.8 mmol, 1.1 equiv), diethyl malonate (1.22 mL, 8.0 mmol, 1.0 equiv), acetic acid (0.05 mL, 0.8 mmol, 0.1 equiv) and piperidine (0.08 mL, 0.8 mmol, 0.1 equiv) were used to obtain crude residue, which was purified by silica gel column chromatography (hexane/ethyl acetate 20:1, v/v) to afford the corresponding product.

^{1}H NMR (300 MHz, $CDCl_3$) δ 7.61 (s, 1H), 7.01 – 6.98 (m, 1H), 6.96 – 6.95 (m, 1H), 6.82 – 6.79 (m, 1H), 6.01 (s, 2H), 4.36 (q, J = 7.1 Hz, 2H), 4.29 (q, J = 7.1 Hz, 2H), 1.33 (t, J = 7.1 Hz, 3H), 1.32 (t, J = 7.1 Hz, 3H) ppm.

See NMR spectrum

All other data matches what was reported in the literature.[18]

1-((1*R*,5*R*)-2-Isopropyl-5-methylcyclohexyl) 3-((2*S*)-2-isopropyl-5-methylcyclohexyl) (*E*)-2-ethylidenemalonate (2af)

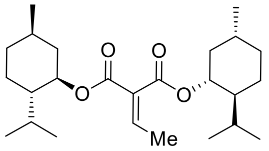

According to **General procedure B**, acetaldehyde (2.2 g, 50.0 mmol, 5.0 equiv) and di-(-)-menthyl malonate (10.0 mmol, 1.0 equiv) were used to obtain a crude residue, which was purified by silica gel column chromatography (hexane/ethyl acetate 30:1, v/v) to afford the corresponding product.

^{1}H NMR (300 MHz, $CDCl_3$) δ 7.01 (q, J = 7.2 Hz, 1H), 4.87 (td, J = 11.2, 4.7 Hz, 1H), 4.78 (td, J = 11.2, 4.7 Hz, 1H), 2.11 – 1.97 (m, 2H), 1.93 – 1.91 (m, 2H), 1.72 – 1.65 (m, 4H), 1.58 – 1.35 (m, 7H), 1.14 – 0.97 (m, 4H), 0.94 – 0.87 (m, 14H), 0.81 – 0.74 (m, 6H) ppm.

See NMR spectrum

All other data matches what was reported in the literature.[19]

((Phenylethynyl)sulfonyl)benzene (2ag)

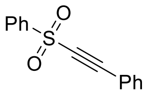

According to the literature procedure,[20] phenylpropiolic acid (4.0 mmol), $PhSO_2Na$ (8.0 mmol), I_2 (2.00 mmol), TBHP (70% in H_2O, 12.0 mmol) and THF (16 mL) were used to afford **2ag**.

^{1}H NMR (300 MHz, $CDCl_3$) δ 8.10 – 8.07 (m, 2H), 7.72 – 7.67 (m, 1H), 7.63 – 7.57 (m, 2H), 7.54 – 7.45 (m, 3H), 7.40 – 7.35 (m, 2H) ppm.

See NMR spectrum

All other data matches what was reported in the literature.[20]

(*E*)-(2-(Phenylsulfonyl)vinyl)benzene (2ah)

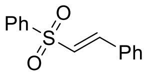

According to the literature procedure,[20] styrene (10.0 mmol), $PhSO_2Na$ (30.0 mmol), NaOAc (15.0 mmol), I_2 (15.0 mmol) and ACN (40 mL) were used to afford **2ah**.

^{1}H NMR (300 MHz, $CDCl_3$) δ 7.97 – 7.94 (m, 2H), 7.69 (d, J = 15.4 Hz, 1H), 7.62 – 7.47 (m, 4H), 7.41 – 7.36 (m, 3H), 6.86 (d, J = 15.4 Hz, 1H) ppm.

See NMR spectrum

All other data matches what was reported in the literature.[20]

((2-Phenylallyl)sulfonyl)benzene (2ai)

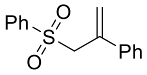

According to the literature procedure,[20] $PhSO_2Na$ (20.0 mmol) and NaOAc (10.0 mmol) in MeCN (30 mL), 2-phenylpropene (1.00 mL, 7.0 mmol) and I_2 (10.0 mmol) were used to afford **2ai**.

^{1}H NMR (300 MHz, $CDCl_3$) δ 7.80 – 7.77 (m, 2H), 7.58 – 7.52 (m, 1H), 7.46 – 7.40 (m, 2H), 7.27 – 7.22 (m, 5H), 5.60 (s, 1H), 5.23 (d, J = 0.5 Hz, 1H), 4.28 (d, J = 0.5 Hz, 2H).

See NMR spectrum

All other data matches what was reported in the literature.[20]

***S*-Phenyl benzenesulfonothioate (2aj)**

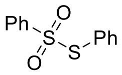

According to the literature procedure,[21] diphenyl disulfide (10.0 mmol), sodium benzenesulfinate (32.0 mmol), I_2 (20.0 mmol), and DCM (50 mL) were used to afford **2aj**.

^{1}H NMR (300 MHz, $CDCl_3$) δ 7.60 – 7.55 (m, 3H), 7.50 – 7.30 (m, 7H) ppm.

See NMR spectrum

All other data matches what was reported in the literature.[21]

Se-Phenyl benzenesulfonoselenoate (2ak)

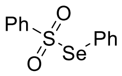

According to the literature procedure,[22] $PhSO_2Na$ (10.0 mmol), diphenyl diselenide (2.5 mmol), NBS (5.0 mmol), and MeCN (20 mL) were used to afford **2ak**.

^{1}H NMR (300 MHz, $CDCl_3$) δ 7.58 – 7.47 (m, 6H), 7.45 – 7.31 (m, 4H) ppm.

See NMR spectrum

All other data matches what was reported in the literature.[22]

2.3. Condition optimization of Autocatalytic EDA promoted Giese reaction

Table S10 Optimization of EDA promoted Giese reaction[a]

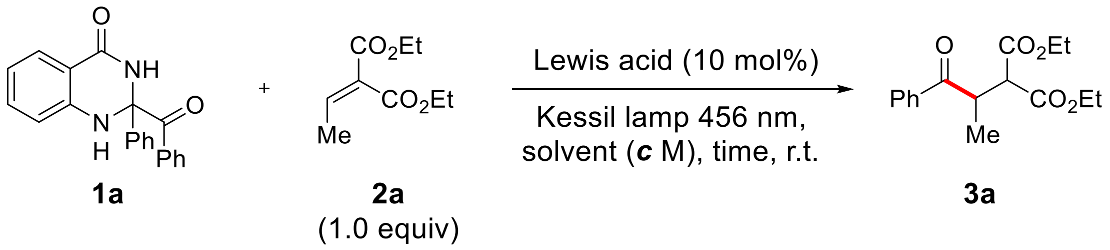

Entry	Lewis acid	Solvent (*c* M)	Time (hr)	Yield (%)[b]
1	$Zn(OTf)_2$	ACN (0.05)	16	66
2	$Cu(OTf)_2$	ACN (0.05)	16	49
3	$InCl_3$	ACN (0.05)	16	63
4	$MgCl_2$	ACN (0.05)	16	56
5	$AlCl_3$	ACN (0.05)	16	21
6	$Zn(OTf)_2$	THF (0.05)	16	51
7	$Zn(OTf)_2$	Toluene (0.05)	16	60
8	$Zn(OTf)_2$	ACN (0.03)	16	52
9	$Zn(OTf)_2$	ACN (0.1)	16	18
10	$Zn(OTf)_2$	ACN (0.05)	12	46
11	$Zn(OTf)_2$	ACN (0.05)	24	59
12[c]	$Zn(OTf)_2$	ACN (0.05)	16	82
13[d]	$Zn(OTf)_2$	ACN (0.05)	16	85[e]

[a] Reaction conditions: **1a** (0.20 mmol), **2a** (0.10 mmol), Lewis acid (0.01 mmol) in anhydrous solvent (2.0 mL). [b] Yields were determined by ^1H NMR with 1,1,2,2-tetrachloroethane as an internal standard. [c] **1a** (0.15 mmol, 1.5 equiv). [d] Upscale reaction: **1a** (1.5 mmol, 1.5 equiv), **2a** (1.0 mmol, 1.0 equiv). [e] Isolated yield.

General procedure of reaction optimization: In a nitrogen-filled glove box, a tube equipped with a stir bar was added with DHQZ **1a**, the Michael acceptor **2a**, and Lewis acid (10 mol%) in anhydrous solvent (2 mL, 0.05 M). The resulting solution was removed from the glove box and irradiated with blue light Kessil PR160L (456 nm, 50 W) at a distance of 2.0 cm from the tube for 16 hr. After the reaction was completed, the mixture was concentrated under reduced pressure and a high vacuum. The resulting residue was added with an NMR internal standard (tetrachloroethane, CAS# 79-34-5, 15 – 20 mg) and diluted with $CDCl_3$. The yield of **3a** was determined by ^{1}H NMR analysis.

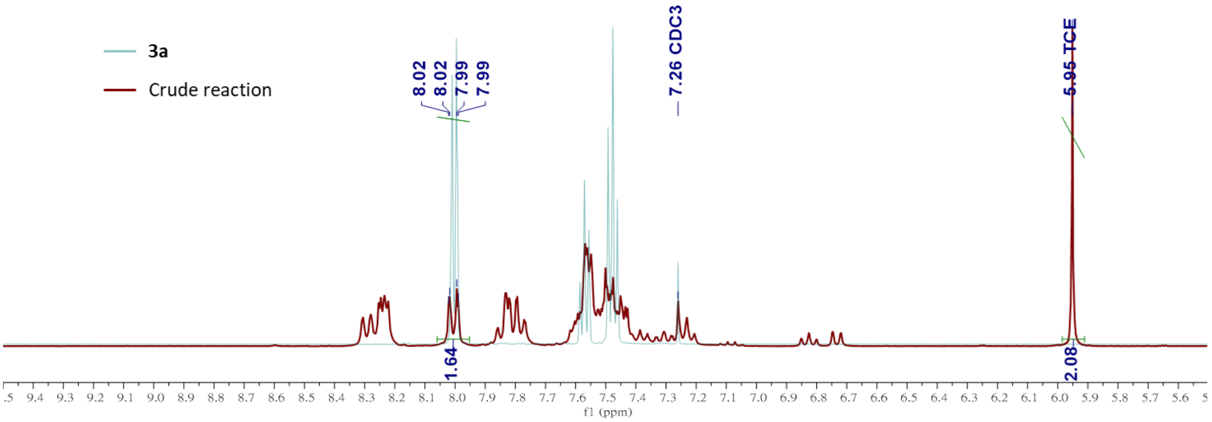

Figure S2 NMR yield determination.

The chemical shift of tetrachloroethane is at 5.95 ppm while the characterization peak of **3a** was 8.01 – 7.99 (m, 2H) ppm. (Take entry 12 as an example.)

2.4. Procedure of Autocatalytic EDA Complex Acylation/Alkylation

General procedure E

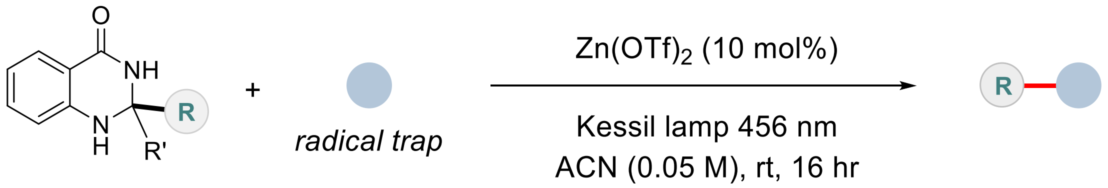

In a nitrogen-filled glove box, an oven-dried tube equipped with a stir bar was added with DHQZ **1** (0.15-0.30 mmol, 1.5-3.0 equiv), the radical trap **2** (0.10 mmol, 1.0 equiv), and $Zn(OTf)_2$ (3.6 mg, 0.01 mmol, 10 mol%) in anhydrous ACN (2 mL, 0.05 M). The resulting solution was removed from the glove box and irradiated with blue light Kessil PR160L (456 nm, 50 W) at a distance of 2.0 cm from the tube for 16 hr. After the reaction was completed, the mixture was concentrated under reduced pressure and a high vacuum. The resulting residue was purified by silica gel column chromatography or preparative TLC on silica gel to afford the product.

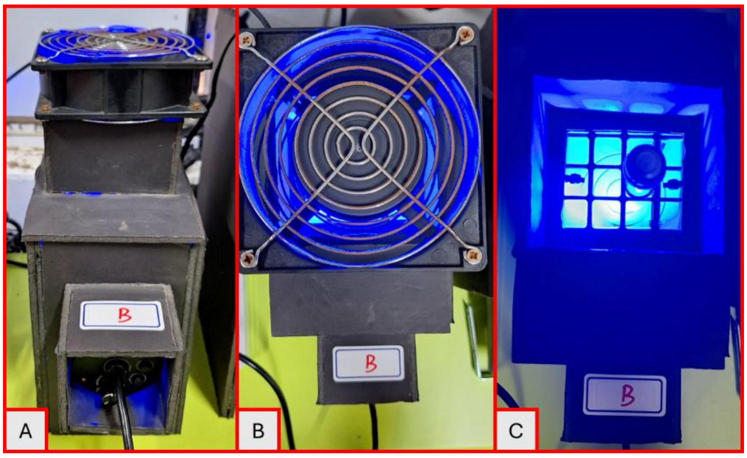

Figure S3 Standard reaction set up (A) Front view; (B) Top view; (C) Interior view.

Back to contents

2.5. Substrate Scope of Autocatalytic EDA Complex Acylation/Alkylation

Table S11 Substrate scope of compound 3

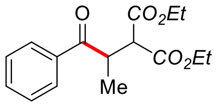 **3a**	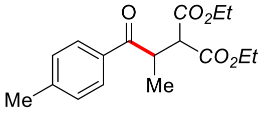 **3b**	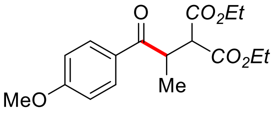 **3c**	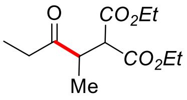 **3d**	
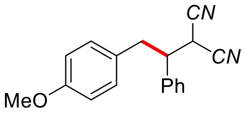 **3e**	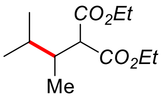 **3f**	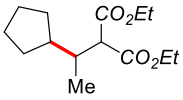 **3g**	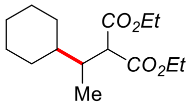 **3h**	
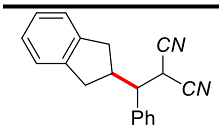 **3i**	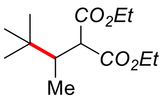 **3j**	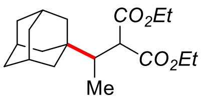 **3k**	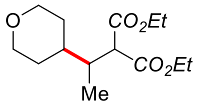 **3l**	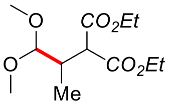 **3m**

Table S12 Substrate scope of compound 4

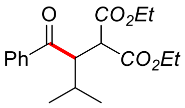 **4a**	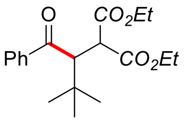 **4b**	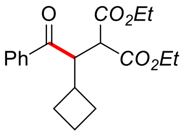 **4c**	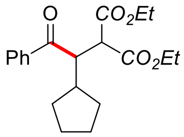 **4d**	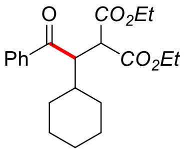 **4e**
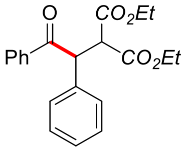 **4f**	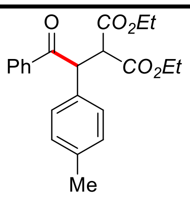 **4g**	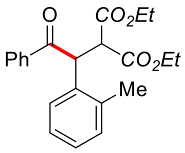 **4h**	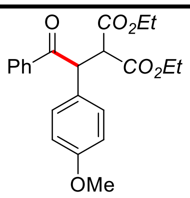 **4i**	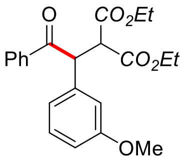 **4j**
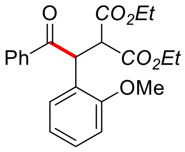 **4k**	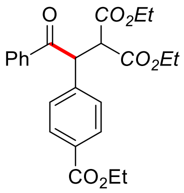 **4l**	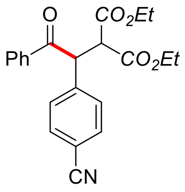 **4m**	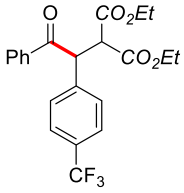 **4n**	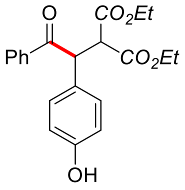 **4o**
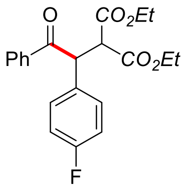 **4p**	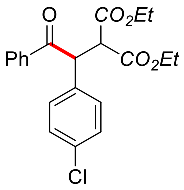 **4q**	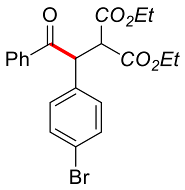 **4r**	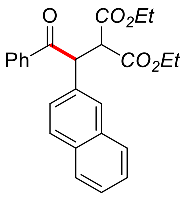 **4s**	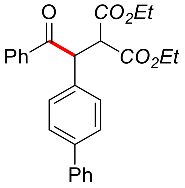 **4t**

Back to contents

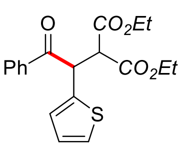 **4u**	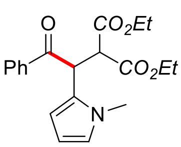 **4v**	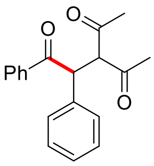 **4w**	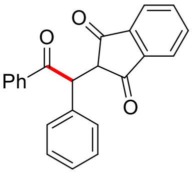 **4x**	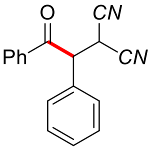 **4y**
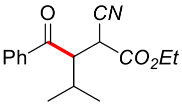 **4z**	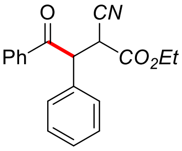 **4aa**	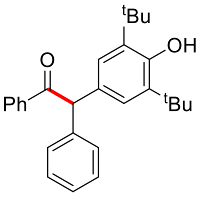 **4ab**	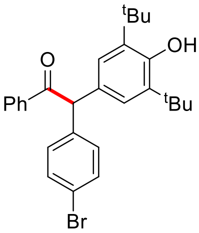 **4ac**	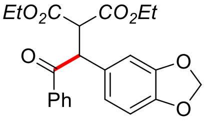 **4ad**
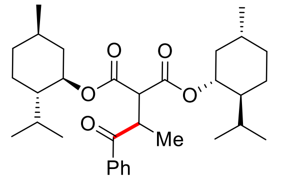 **4ae**		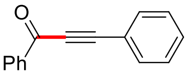 **5a**	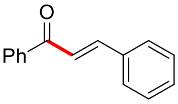 **5b**	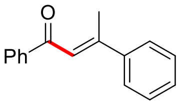 **5c**
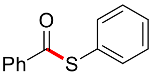 **5d**	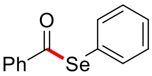 **5e**	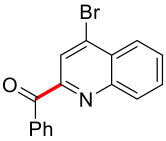 **6a**	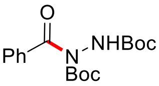 **7a**	

Diethyl 2-(1-oxo-1-phenylpropan-2-yl)malonate (3a)

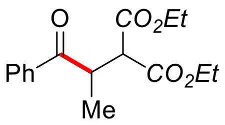

In a N_2 filled glove box, DHQZ **1a** (492.6 mg, 1.5 mmol, 1.5 equiv), **2a** (186.2 mg, 1.0 mmol, 1.0 equiv), and $Zn(OTf)_2$ (36.3 mg, 0.1 mmol, 10 mol%) was added to an oven-dried seal tube. Dissolve the mixture with 20 mL ACN. The tube was sealed and stirred under 456 nm irradiation for 16 hr. The crude residue was purified by silica gel column chromatography (hexane/ethyl acetate 25:1, v/v) to afford the corresponding product **3a** as a colorless liquid (248.0 mg, 0.849 mmol, 85%).

^{1}H NMR (500 MHz, $CDCl_3$) δ 8.01 – 7.99 (m, 2H), 7.57 (tt, J = 7.4, 1.3 Hz, 1H), 7.49 – 7.46 (m, 2H), 4.30 – 4.23 (m, 2H), 4.22 – 4.16 (m, 1H), 4.14 – 4.06 (m, 2H), 3.98 (d, J = 10.8 Hz, 1H), 1.31 (t, J = 7.2 Hz, 3H), 1.20 – 1.15 (m, 6H) ppm.

^{13}C{^{1}H} NMR (101 MHz, $CDCl_3$) δ 201.6, 168.8, 168.4, 135.6, 133.2, 128.7, 128.5, 61.7, 54.9, 40.5, 15.9, 14.1, 13.9 ppm.

See NMR spectrum

HRMS (m/z): (EI) calc'd for $C_{16}H_{20}O_5$ $[M]^+$: 292.1305, found: 292.1305

IR (ATR) ν_{max}: 2980, 2928, 1730, 1682, 1294, 1184 and 1024 cm^{-1}

TLC: R_f = 0.23 (hexane/ethyl acetate 8:1, v/v)

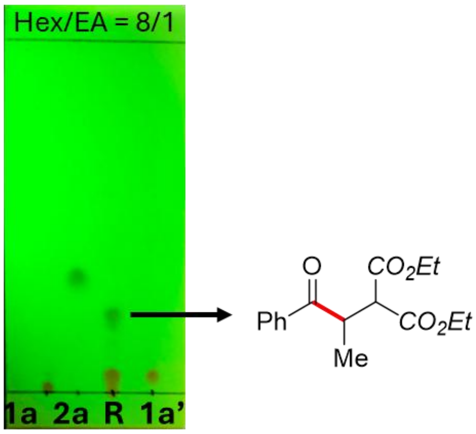

Diethyl 2-(1-oxo-1-(p-tolyl)propan-2-yl)malonate (3b)

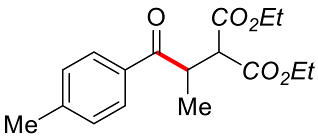

According to **General procedure E**, DHQZ **1b** (53.4 mg, 0.15 mmol, 1.5 equiv) and **2a** (18.6 mg, 0.1 mmol, 1.0 equiv) were used to obtain crude residue, which was purified by silica gel column chromatography (hexane/ethyl acetate 15:1, v/v) to afford the corresponding product **3b** as colorless oil (25.7 mg, 0.084 mmol, 84%).

^{1}H NMR (300 MHz, $CDCl_3$) δ 7.90 (d, J = 8.0 Hz, 2H), 7.27 (d, J = 8.3 Hz, 2H), 4.29 – 4.03 (m, 5H), 3.97 (d, J = 10.8 Hz, 1H), 2.40 (s, 3H), 1.31 (t, J = 7.1 Hz, 3H), 1.19 – 1.07 (m, 6H) ppm.

^{13}C{^{1}H} NMR (101 MHz, $CDCl_3$) δ 201.2, 168.8, 168.3, 144.0, 133.0, 129.4, 128.6, 61.6, 54.9, 40.4, 21.6, 15.9, 14.1, 13.8 ppm.

See NMR spectrum

HRMS (m/z): (ESI) calc'd for $C_{17}H_{22}O_5{}^{23}Na$ $[M+Na]^+$: 329.1359, found: 329.1358

IR (ATR) ν_{max}: 2980, 2936, 1747, 1730, 1679, 1454, 1368, 1294, 1182 and 1028 cm^{-1}

TLC: R_f = 0.25 (hexane/ethyl acetate 8:1, v/v)

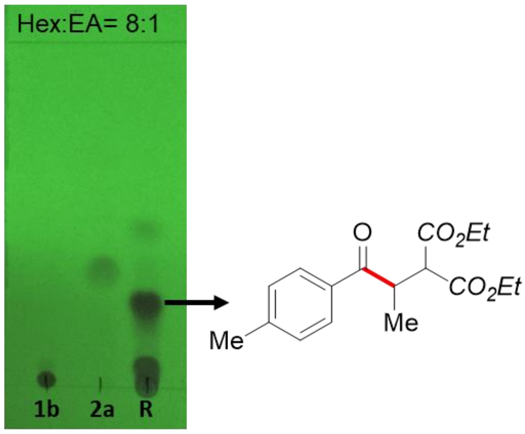

Diethyl 2-(1-(4-methoxyphenyl)-1-oxopropan-2-yl)malonate (3c)

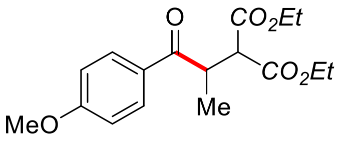

According to **General procedure E**, DHQZ **1c** (58.2 mg, 0.15 mmol, 1.5 equiv) and **2a** (18.6 mg, 0.1 mmol, 1.0 equiv) were used to obtain crude residue, which was purified by silica gel column chromatography (hexane/ethyl acetate 15:1, v/v) to afford the corresponding product **3c** as colorless oil (26.7 mg, 0.083 mmol, 83%).

^{1}H NMR (400 MHz, $CDCl_3$) δ 7.99 (d, J = 8.8 Hz, 2H), 6.95 (d, J = 8.8 Hz, 2H), 4.32 – 4.21 (m, 2H), 4.19 – 4.03 (m, 3H), 3.97 (d, J = 10.8 Hz, 1H), 3.87 (s, 3H), 1.31 (t, J = 7.1 Hz, 4H), 1.20 – 1.14 (m, 6H) ppm.

^{13}C{^{1}H} NMR (101 MHz, $CDCl_3$) δ 200.1, 168.9, 168.4, 163.6, 130.8, 128.5, 113.9, 61.6, 55.5, 54.9, 40.1, 16.1, 14.1, 13.9 ppm.

See NMR spectrum

TLC: R_f = 0.16 (hexane/ethyl acetate 8:1, v/v)

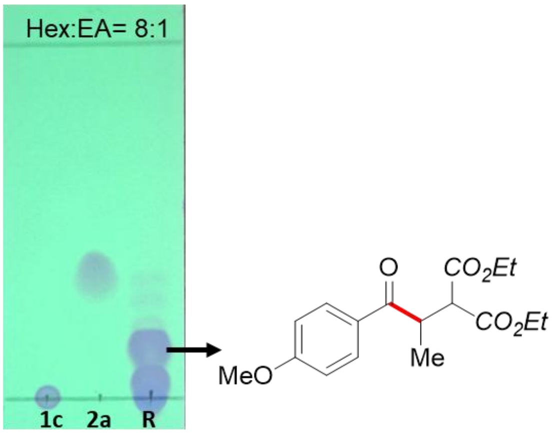

All other data matches what was reported in the literature.[24]

Diethyl 2-(3-oxopentan-2-yl)malonate (3d)

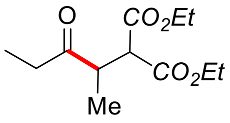

According to **General procedure E**, DHQZ **1d** (34.8 mg, 0.15 mmol, 1.5 equiv) and **2a** (18.6 mg, 0.1 mmol, 1.0 equiv) were used to obtain a crude residue, which was purified by silica gel column chromatography (hexane/ethyl acetate 20:1, v/v) to afford the corresponding product **3d** as colorless oil (18.3 mg, 0.075 mmol, 75%).

^{1}H NMR (300 MHz, $CDCl_3$) δ 4.25 – 4.08 (m, 4H), 3.76 (d, J = 10.6 Hz, 1H), 3.32 – 3.21 (m, 1H), 2.65 – 2.57 (m, 2H), 1.30 – 1.20 (m, 6H), 1.11 – 1.04 (m, 6H) ppm.

^{13}C{^{1}H} NMR (101 MHz, $CDCl_3$) δ 212.2, 168.6, 168.5, 61.6, 61.5, 54.5, 44.7, 34.6, 14.8, 14.1, 13.9, 7.6 ppm.

See NMR spectrum

TLC: R_f = 0.35 (hexane/ethyl acetate 8:1, v/v)

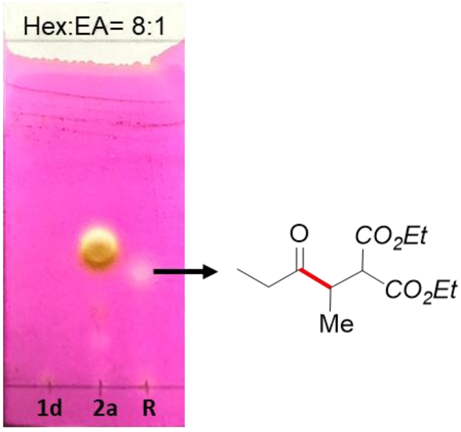

All other data matches what was reported in the literature.[25]

2-(2-(4-methoxyphenyl)-1-phenylethyl)malononitrile (3e)

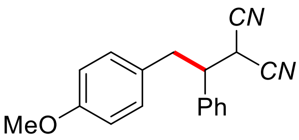

According to **General procedure E**, DHQZ **1e** (56.1 mg, 0.15 mmol, 1.5 equiv) and **2z** (15.4 mg, 0.1 mmol, 1.0 equiv) were used to obtain a crude residue, which was purified by silica gel column chromatography (hexane/ethyl acetate 10:1, v/v) to afford the corresponding product **3e** as colorless oil (20.1 mg, 0.073 mmol, 73%).

^{1}H NMR (400 MHz, $CDCl_3$) δ 7.44 – 7.38 (m, 5H), 7.10 (d, J = 8.4 Hz, 2H), 6.86 (d, J = 8.4 Hz, 2H), 3.86 (d, J = 5.1 Hz, 1H), 3.80 (s, 3H), 3.44 – 3.38 (m, 1H), 3.21 (d, J = 8.3 Hz, 2H) ppm.

^{13}C{^{1}H} NMR (101 MHz, $CDCl_3$) δ 159.0, 136.5, 129.9, 129.1, 129.0, 128.5, 128.0, 114.5, 112.1, 111.4, 55.3, 48.5, 37.6, 28.3 ppm.

See NMR spectrum

TLC: R_f = 0.33 (hexane/ethyl acetate 8:1, v/v)

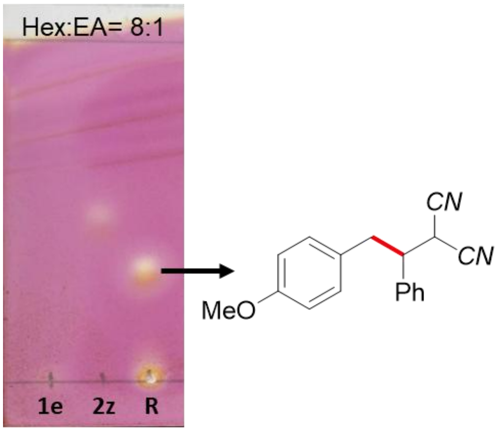

All other data matches what was reported in the literature.[26]

Back to contents/Back to table

Diethyl 2-(3-methylbutan-2-yl)malonate (3f)

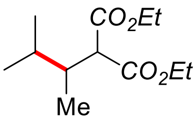

According to **General procedure E**, DHQZ **1f** (39.9 mg, 0.15 mmol, 1.5 equiv) and **2a** (18.6 mg, 0.1 mmol, 1.0 equiv) were used to obtain crude residue, which was purified by silica gel column chromatography (hexane/ethyl acetate 20:1, v/v) to afford the corresponding product **3f** as colorless oil (14.5 mg, 0.063 mmol, 63%).

^{1}H NMR (300 MHz, $CDCl_3$) δ 4.18 (q, J = 7.1 Hz, 4H), 3.33 (d, J = 9.4 Hz, 1H), 2.25 – 2.13 (m, 1H), 1.72 – 1.62 (m, 1H), 1.28 – 1.23 (m, 6H), 0.93 (d, J = 6.8 Hz, 3H), 0.87 (d, J = 6.9 Hz, 3H), 0.81 (d, J = 6.8 Hz, 3H) ppm.

See NMR spectrum

TLC: R_f = 0.30 (hexane/ethyl acetate 8:1, v/v)

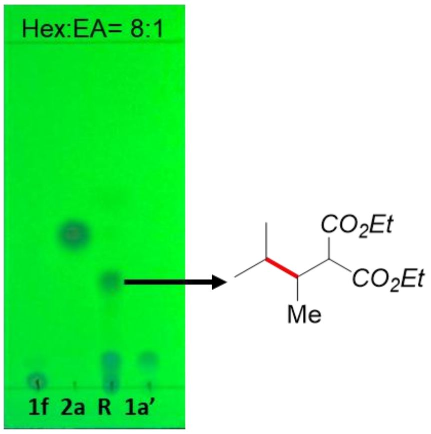

All other data matches what was reported in the literature.[27]

Diethyl 2-(1-cyclopentylethyl)malonate (3g)

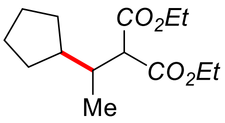

According to **General procedure E**, DHQZ **1g** (35.4 mg, 0.15 mmol, 1.5 equiv) and **2a** (18.6 mg, 0.1 mmol, 1.0 equiv) were used to obtain crude residue, which was purified by silica gel column chromatography (hexane/ethyl acetate 15:1, v/v) to afford the corresponding product **3g** as colorless oil (13.3 mg, 0.052 mmol, 52%).

^{1}H NMR (400 MHz, CDCl$_3$) δ 4.19 (q, J = 7.1 Hz, 4H), 3.43 (d, J = 6.3 Hz, 1H), 2.18 – 2.10 (m, 1H), 1,82 – 1.71 (m, 3H), 1.64 – 1.57 (m, 4H), 1.53 – 1.50 (m, 2H), 1.29 – 1.25 (m, 6H), 1.20 – 1.11 (m, 2H), 1.01 (d, J = 6.8 Hz, 3H) ppm.

^{13}C{^{1}H} NMR (101 MHz, CDCl$_3$) δ 169.5, 168.9, 56.3, 43.8, 38.4, 30.9, 29.6, 25.3, 25.3, 14.6, 14.1, 14.1 ppm.

See NMR spectrum

TLC: R$_f$= 0.55 (hexane/ethyl acetate 8:1, v/v)

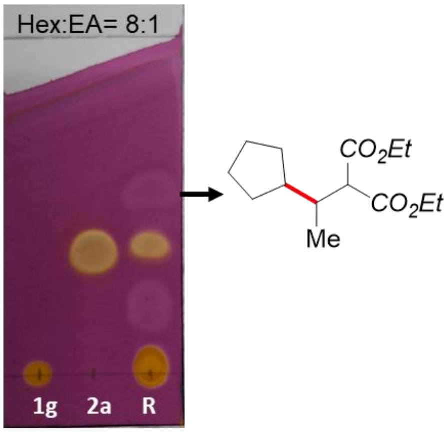

All other data matches what was reported in the literature.[27]

Diethyl 2-(1-cyclohexylethyl)malonate (3h)

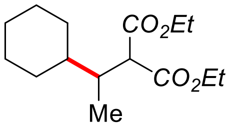

According to **General procedure E**, DHQZ **1h** (45.9 mg, 0.15 mmol, 1.5 equiv) and **2a** (18.6 mg, 0.1 mmol, 1.0 equiv) were used to obtain crude residue, which was purified by silica gel column chromatography (hexane/ethyl acetate 20:1, v/v) to afford the corresponding product **3h** as colorless oil (12.7 mg, 0.047 mmol, 47%).

^{1}H NMR (300 MHz, $CDCl_3$) δ 4.23 – 4.15 (m, 4H), 3.39 (d, J = 9.2 Hz, 1H), 2.23 – 2.12 (m, 1H), 1.75 – 1.62 (m, 3H), 1.29 – 1.24 (m, 10H), 1.17 – 1.11 (m, 1H), 0.99 – 0.83 (m, 6H) ppm.

^{13}C{^{1}H} NMR (101 MHz, $CDCl_3$) δ 169.3, 169.1, 61.1, 61.0, 55.8, 40.3, 38.5, 31.5, 29.7, 27.4, 26.7, 26.5, 26.5, 14.1, 12.9 ppm.

See NMR spectrum

TLC: R_f = 0.41 (hexane/ethyl acetate 8:1, v/v)

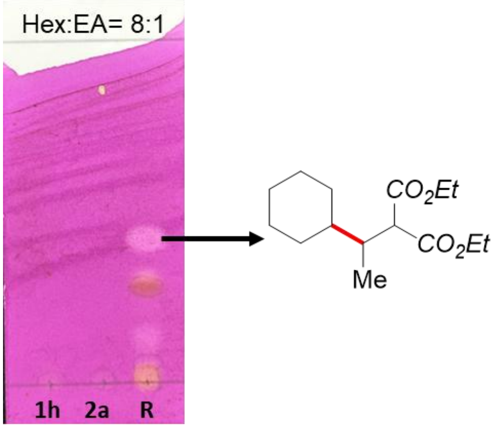

All other data matches what was reported in the literature.[27]

2-((2,3-dihydro-1H-inden-2-yl)(phenyl)methyl)malononitrile (3i)

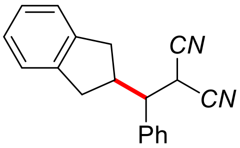

According to **General procedure E**, DHQZ **1i** (86.1 mg, 0.30 mmol, 3.0 equiv) and **2z** (15.4 mg, 0.1 mmol, 1.0 equiv) were used to obtain a crude residue, which was purified by silica gel column chromatography (hexane/ethyl acetate 15:1, v/v) to afford the corresponding product **3i** as pale-yellow oil (20.1 mg, 0.048 mmol, 48%).

^{1}H NMR (400 MHz, $CDCl_3$) δ 7.47 – 7.40 (m, 5H), 7.26 – 7.24 (m, 1H), 7.19 – 7.12 (m, 2H), 7.08 (d, J = 6.9 Hz, 1H), 4.15 (d, J = 3.4 Hz, 1H), 3.35 – 3.19 (m, 3H), 2.89 – 2.78 (m, 2H), 2.54 (dd, J = 16.1, 7.9 Hz, 1H).

^{13}C{^{1}H} NMR (101 MHz, $CDCl_3$) δ 141.7, 141.2, 136.7, 129.4, 129.1, 128.1, 126.9, 126.8, 124.5, 124.3, 111.8, 111.6, 51.6, 42.5, 38.3, 37.7, 29.0 ppm.

See NMR spectrum

TLC: R_f = 0.6 (hexane/ethyl acetate 8:1, twice, v/v)

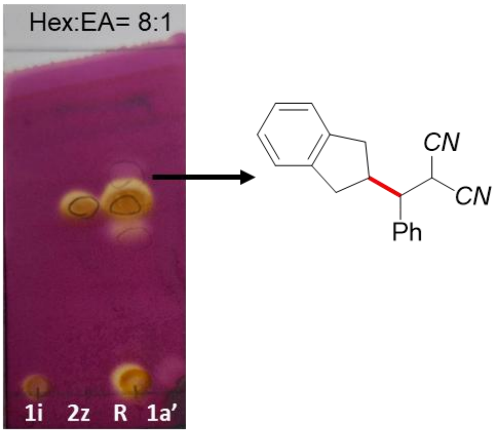

All other data matches what was reported in the literature.[28]

Diethyl 2-(3,3-dimethylbutan-2-yl)malonate (3j)

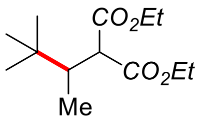

According to **General procedure E**, DHQZ **1j** (32.7 mg, 0.15 mmol, 1.5 equiv) and **2a** (18.6 mg, 0.1 mmol, 1.0 equiv) were used to obtain crude residue, which was purified by silica gel column chromatography (hexane/ethyl acetate 15:1, v/v) to afford the corresponding product **3j** as pale-yellow oil (10.2 mg, 0.042 mmol, 42%).

^{1}H NMR (400 MHz, $CDCl_3$) δ 4.21 – 4.14 (m, 4H), 3.51 (d, J = 5.4 Hz, 1H), 2.24 (qd, J = 7.2, 5.4 Hz, 1H), 1.29 – 1.24 (m, 6H), 1.01 (d, J = 7.2 Hz, 3H), 0.90 (s, 9H) ppm.

^{13}C{^{1}H} NMR (101 MHz, $CDCl_3$) δ 170.2, 169.6, 61.3, 60.9, 53.4, 42.6, 33.6, 29.8, 27.5, 19.8, 14.0, 12.1 ppm.

See NMR spectrum

TLC: R_f = 0.57 (hexane/ethyl acetate 8:1, v/v)

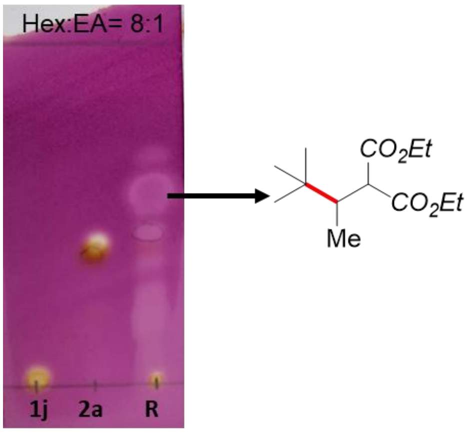

All other data matches what was reported in the literature.[27]

Diethyl 2-(1-(adamantan-1-yl)ethyl)malonate (3k)

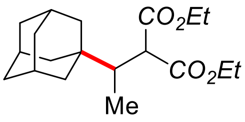

According to **General procedure E**, DHQZ **1k** (44.4 mg, 0.15 mmol, 1.5 equiv) and **2a** (18.6 mg, 0.1 mmol, 1.0 equiv) were used to obtain crude residue, which was purified by silica gel column chromatography (hexane/ethyl acetate 20:1, v/v) to afford the corresponding product **3k** as colorless oil (22.2 mg, 0.069 mmol, 69%).

^{1}H NMR (300 MHz, $CDCl_3$) δ 4.17 (qd, J = 7.1, 3.7 Hz, 4H), 3.56 (d, J = 5.2 Hz, 1H), 2.10 – 2.01 (m, 1H), 1.95 (s, 3H), 1.63 (q, J = 11.9 Hz, 7H), 1.50 – 1.46 (m, 5H), 1.29 – 1.22 (m, 6H), 0.97 (d, J = 7.3 Hz, 3H) ppm.

^{13}C{^{1}H} NMR (101 MHz, $CDCl_3$) δ 170.4, 169.8, 61.3, 60.8, 51.8, 43.1, 39.4, 37.0, 35.2, 28.6, 14.0, 14.0, 10.4 ppm.

See NMR spectrum

HRMS (m/z): (ESI) calc'd for $C_{19}H_{30}O_4{}^{23}Na$ $[M+Na]^+$: 345.2036, found: 345.2034

IR (ATR) ν_{max}: 2979, 2847, 1750, 1730, 1447, 1336, 1293, 1219 and 1147 cm^{-1}

TLC: R_f= 0.41 (hexane/ethyl acetate 8:1, v/v)

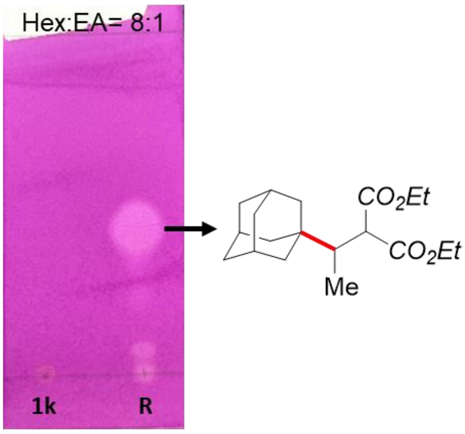

Back to contents/Back to table

Diethyl 2-(1-(tetrahydro-2H-pyran-4-yl)ethyl)malonate (3l)

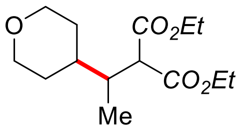

According to **General procedure E**, DHQZ **1l** (36.9 mg, 0.15 mmol, 1.5 equiv) and **2a** (18.6 mg, 0.1 mmol, 1.0 equiv) were used to obtain crude residue, which was purified by silica gel column chromatography (hexane/ethyl acetate 15:1, v/v) to afford the corresponding product **3l** as colorless oil (10.3 mg, 0.038 mmol, 38%).

^{1}H NMR (400 MHz, $CDCl_3$) δ 4.24 – 4.17 (m, 4H), 4.01 – 3.97 (m, 2H), 3.43 – 3.28 (m, 3H), 2.22 – 2.13 (m, 1H), 1.56 – 1.49 (m, 4H), 1.40 – 1.32 (m, 1H), 1.27 (t, J = 7.1 Hz, 6H), 0.96 (d, J = 6.9 Hz, 3H) ppm.

^{13}C{^{1}H} NMR (101 MHz, $CDCl_3$) δ 169.6, 168.7, 68.2, 68.1, 61.3, 61.2, 54.9, 38.0, 37.8, 31.1, 28.2, 14.1, 13.0 ppm.

See NMR spectrum

TLC: R_f= 0.30 (hexane/ethyl acetate 8:1, v/v)

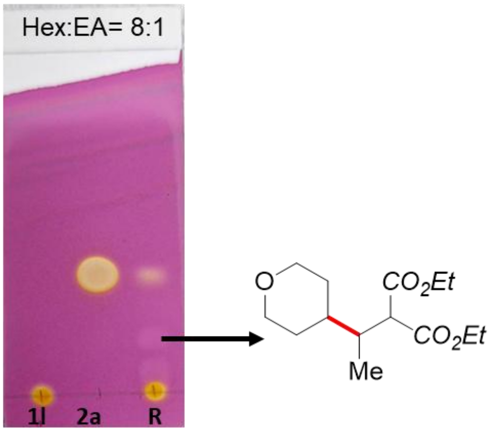

All other data matches what was reported in the literature.[27]

Diethyl 2-(1,1-dimethoxypropan-2-yl)malonate (3m)

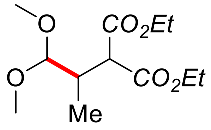

According to **General procedure E**, DHQZ **1m** (35.4 mg, 0.15 mmol, 1.5 equiv) and **2a** (18.6 mg, 0.1 mmol, 1.0 equiv) were used to obtain crude residue, which was purified by silica gel column chromatography (hexane/ethyl acetate 15:1, v/v) to afford the corresponding product **3m** as colorless oil (14.7 mg, 0.056 mmol, 56%).

^{1}H NMR (400 MHz, $CDCl_3$) δ 4.30 (d, J = 6.3 Hz, 1H), 4.19 (q, J = 7.1 Hz, 4H), 3.49 (d, J = 6.9 Hz, 1H), 3.36 (s, 3H), 3.34 (s, 3H), 2.61 – 2.52 (m, 1H), 1.27 (t, J = 7.2 Hz, 6H), 1.02 (d, J = 6.9 Hz, 3H) ppm.

^{13}C{^{1}H} NMR (101 MHz, $CDCl_3$) δ 168.9, 168.7, 106.4, 61.2, 61.1, 55.3, 53.7, 53.4, 36.2, 14.1, 14.1, 12.0 ppm.

[See NMR spectrum](#)

TLC: R_f = 0.24 (hexane/ethyl acetate 8:1, v/v)

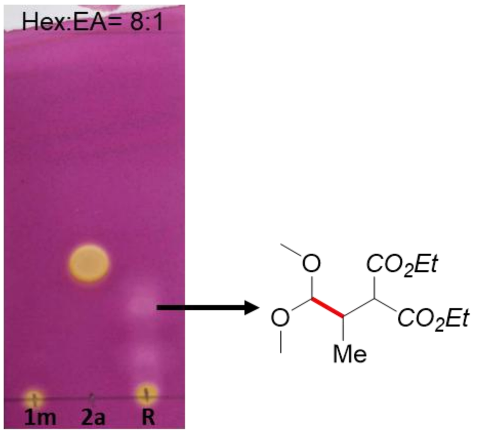

HRMS (m/z): (ESI) calc'd for $C_{12}H_{22}O_6{}^{23}Na$ $[M+Na]^+$: 285.1309, found: 285.1307

Back to contents/Back to table

Diethyl 2-(3-methyl-1-oxo-1-phenylbutan-2-yl)malonate (4a)

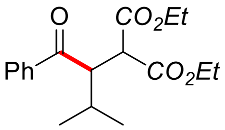

According to **General procedure E**, DHQZ **1a** (49.2 mg, 0.15 mmol, 1.5 equiv) and **2b** (21.4 mg, 0.1 mmol, 1.0 equiv) were used to obtain a crude residue, which was purified by silica gel column chromatography (hexane/ethyl acetate 25:1, v/v) to afford the corresponding product **4a** as light-yellow oil (22.1 mg, 0.069 mmol, 69%).

^{1}H NMR (300 MHz, $CDCl_3$) δ 8.01 (d, J = 7.3 Hz, 2H), 7.58 – 7.53 (m, 1H), 7.49 – 7.44 (m, 2H), 4.31 – 4.20 (m, 3H), 4.16 – 4.02 (m, 3H), 2.04 – 1.92 (m, 1H), 1.31 (t, J = 7.1 Hz, 3H), 1.13 (t, J = 7.1 Hz, 3H), 0.92 (d, J = 6.9 Hz, 3H), 0.84 (d, J = 7.1 Hz, 3H) ppm.

^{13}C{^{1}H} NMR (101 MHz, $CDCl_3$) δ 201.4, 169.0, 168.7, 138.5, 132.8, 128.5, 128.4, 61.7, 61.6, 53.0, 50.0, 29.0, 21.1, 18.3, 14.0, 13.8 ppm.

See NMR spectrum

HRMS (m/z): (EI) calc'd for $C_{18}H_{24}O_5$ $[M]^+$: 320.1618, found: 320.1619

IR (ATR) ν_{max}: 2966, 2940, 2877, 1731, 1676, 1180 and 1030 cm^{-1}

TLC: R_f = 0.31 (hexane/ethyl acetate 8:1, v/v)

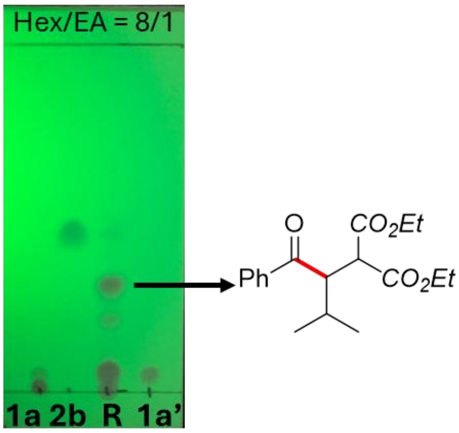

Back to contents/Back to table

Diethyl 2-(3,3-dimethyl-1-oxo-1-phenylbutan-2-yl)malonate, (4b)

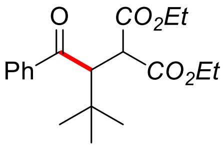

According to **General procedure E**, DHQZ **1a** (49.2 mg, 0.15 mmol, 1.5 equiv) and **2c** (22.8 mg, 0.1 mmol, 1.0 equiv) were used to obtain a crude residue, which was purified by silica gel column chromatography (hexane/ethyl acetate 25:1, v/v) to afford the corresponding product **4b** as light-yellow oil (9.7 mg, 0.029 mmol, 29%).

^{1}H NMR (300 MHz, $CDCl_3$) δ 8.05 – 8.03 (m, 2H), 7.56 – 7.43 (m, 3H), 4.32 – 4.21 (m, 3H), 4.11 (d, J = 10.6 Hz, 1H), 4.06 – 3.92 (m, 2H), 1.32 (t, J = 7.2 Hz, 3H), 1.06 (t, J = 7.1 Hz, 3H), 0.91 (s, 9H) ppm.

^{13}C{^{1}H} NMR (101 MHz, $CDCl_3$) δ 202.9, 169.6, 168.8, 139.7, 132.5, 128.5, 128.4, 61.8, 61.7, 53.5, 53.0, 34.0, 28.6, 13.9, 13.7 ppm.

See NMR spectrum

HRMS (m/z): (EI) calc'd for $C_{19}H_{26}O_5{}^{23}Na$ $[M]^+$: 357.1673, found: 357.1672

IR (ATR) ν_{max}: 2963, 2932, 2850, 1731, 1676, 1467, 1280, 1183 and 1147 cm^{-1}

TLC: R_f = 0.41 (hexane/ethyl acetate 8:1, v/v)

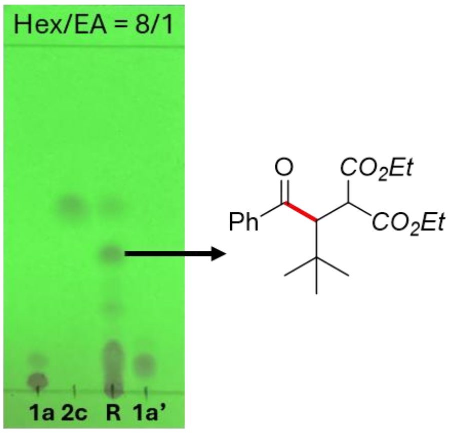

Back to contents/Back to table

Diethyl 2-(1-cyclobutyl-2-oxo-2-phenylethyl)malonate (4c)

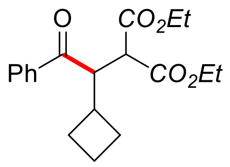

According to **General procedure E**, DHQZ **1a** (49.2 mg, 0.15 mmol, 1.5 equiv) and **2d** (22.6 mg, 0.1 mmol, 1.0 equiv) were used to obtain a crude residue, which was purified by silica gel column chromatography (hexane/ethyl acetate 25:1, v/v) to afford the corresponding product **4c** as a colorless gum (25.9 mg, 0.078 mmol, 78%).

^{1}H NMR (300 MHz, $CDCl_3$) δ 8.02 (d, J = 7.5 Hz, 2H), 7.58 – 7.53 (m, 1H), 7.49 – 7.44 (m, 2H), 4.27 – 4.18 (m, 3H), 4.11 – 3.94 (m, 3H), 2.58 – 2.44 (m, 1H), 1.90 – 1.85 (m, 1H), 1.77 – 1.63 (m, 5H), 1.31 (t, J = 7.1 Hz, 3H), 1.11 (t, J = 7.1 Hz, 3H) ppm.

^{13}C{^{1}H} NMR (101 MHz, $CDCl_3$) δ 201.5, 168.7, 168.4, 138.3, 133.0, 128.6, 128.5, 61.7, 61.6, 53.9, 49.7, 37.4, 28.1, 26.4, 18.8, 14.0, 13.8 ppm.

See NMR spectrum

HRMS (m/z): (EI) calc'd for $C_{19}H_{24}O_5$ $[M]^+$: 332.1618, found: 332.1619

IR (ATR) ν_{max}: 2980, 2871, 1730, 1676, 1291, 1178 and 1035 cm^{-1}

TLC: R_f = 0.34 (hexane/ethyl acetate 8:1, v/v)

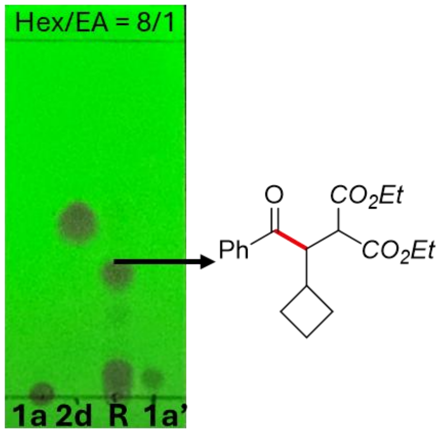

Diethyl 2-(1-cyclopentyl-2-oxo-2-phenylethyl)malonate (4d)

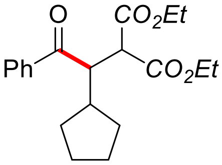

According to **General procedure E**, DHQZ **1a** (49.2 mg, 0.15 mmol, 1.5 equiv) and **2e** (24.0 mg, 0.1 mmol, 1.0 equiv) were used to obtain a crude residue, which was purified by silica gel column chromatography (hexane/ethyl acetate 25:1, v/v) to afford the corresponding product **4d** as a colorless gum (31.8 mg, 0.092 mmol, 92%).

^{1}H NMR (300 MHz, $CDCl_3$) δ 8.03 (d, J = 7.2 Hz, 2H), 7.57 – 7.53 (m, 1H), 7.48 – 7.44 (m, 2H), 4.33 – 4.19 (m, 3H), 4.14 – 3.98 (m, 3H), 2.07 – 1.93 (m, 1H), 1.72 – 1.02 (m, 14H) ppm.

^{13}C{^{1}H} NMR (101 MHz, $CDCl_3$) δ 202.0, 169.1, 168.5, 138.6, 132.8, 128.5, 128.5, 61.7, 61.6, 55.0, 47.8, 42.1, 30.7, 28.9, 24.4, 24.2, 14.0, 13.8 ppm.

See NMR spectrum

HRMS (m/z): (ESI) calc'd for $C_{20}H_{26}O_5{}^{23}Na$ $[M+Na]^+$: 369.1673 , found: 369.1670

IR (ATR) ν_{max}: 2956, 2872, 1729, 1676, 1598, 1447, 1294, 1181 and 1028 cm^{-1}

TLC: R_f= 0.28 (hexane/ethyl acetate 8:1, v/v)

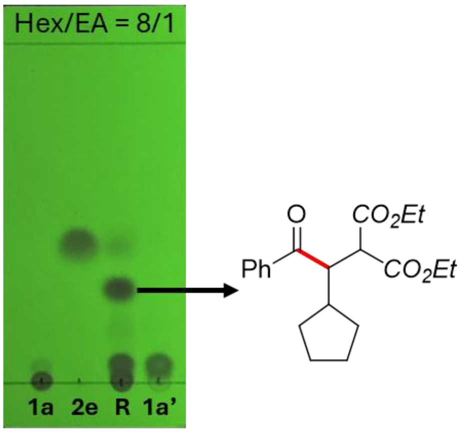

Back to contents/Back to table

Diethyl 2-(1-cyclohexyl-2-oxo-2-phenylethyl)malonate (4e)

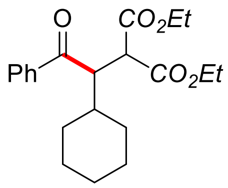

According to **General procedure E**, DHQZ **1a** (49.2 mg, 0.15 mmol, 1.5 equiv) and **2f** (25.4 mg, 0.1 mmol, 1.0 equiv) were used to obtain crude residue, which was purified by silica gel column chromatography (hexane/ethyl acetate 30:1, v/v) to afford the corresponding product **4e** as colorless liquid (21.6 mg, 0.060 mmol, 60%).

^{1}H NMR (400 MHz, $CDCl_3$) δ 8.01 (d, J = 7.4 Hz, 2H), 7.57 – 7.54 (m, 1H), 7.49 – 7.45 (m, 2H), 4.34 – 4.17 (m, 4H), 4.11 – 4.02 (m, 2H), 1.80 – 1.68 (m, 2H), 1.62 – 1.53 (m, 4H), 1.31 (t, J = 7.2 Hz, 3H), 1.12 (t, J = 7.1 Hz, 3H), 1.09 – 0.83 (m, 5H) ppm.

^{13}C{^{1}H} NMR (101 MHz, $CDCl_3$) δ 201.4, 169.1, 168.7, 138.5, 132.8, 128.5, 61.7, 61.6, 52.9, 50.0, 39.3, 32.0, 28.8, 26.9, 26.6, 26.0, 14.1, 13.8 ppm.

See NMR spectrum

HRMS (m/z): (EI) calc'd for $C_{21}H_{28}O_5$ $[M]^+$: 360.1931, found: 360.1929

IR (ATR) ν_{max}: 2982, 2929, 2854, 1729, 1675, 1235 and 1182 cm^{-1}

TLC: R_f= 0.37 (hexane/ethyl acetate 8:1, v/v)

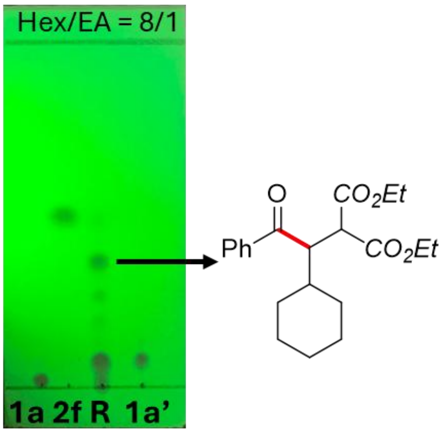

Diethyl 2-(2-oxo-1,2-diphenylethyl)malonate (4f)

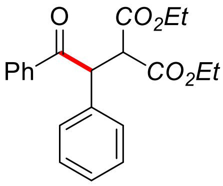

According to **General procedure E**, DHQZ **1a** (49.2 mg, 0.15 mmol, 1.5 equiv) and **2g** (24.8 mg, 0.1 mmol, 1.0 equiv) were used to obtain crude residue, which was purified by silica gel column chromatography (hexane/ethyl acetate 30:1, v/v) to afford the corresponding product **4f** as yellow oil (23.4 mg, 0.066 mmol, 66%).

^{1}H NMR (300 MHz, $CDCl_3$) δ 7.99 (d, J = 8.1 Hz, 2H), 7.50 – 7.45 (m, 1H), 7.41 – 7.36 (m, 2H), 7.33 – 7.26 (m, 3H), 7.24 – 7.16 (m, 2H), 5.32 (d, J = 11.4 Hz, 1H), 4.44 (d, J = 11.4 Hz, 1H), 4.22 – 4.12 (m, 2H) , 3.94 (q, J = 7.1 Hz, 2H), 1.21 (t, J = 7.1 Hz, 3H), 0.95 (t, J = 7.1 Hz, 3H) ppm.

^{13}C{^{1}H} NMR (101 MHz, $CDCl_3$) δ 197.3, 168.1, 168.0, 135.9, 134.5, 133.1, 129.0, 128.9, 128.5, 128.0, 61.9, 61.4, 55.9, 52.9, 13.9, 13.7 ppm.

See NMR spectrum

TLC: R_f = 0.25 (hexane/ethyl acetate 8:1, v/v)

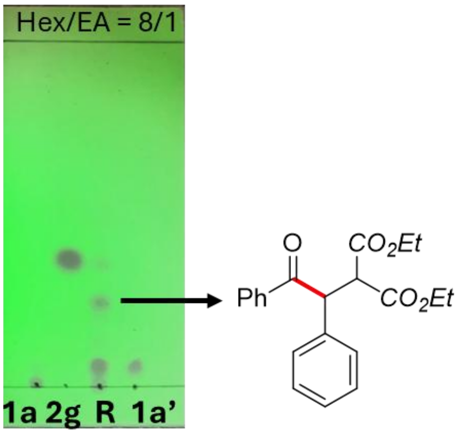

All other data matches what was reported in the literature.[29]

Back to contents/Back to table

Diethyl 2-(2-oxo-2-phenyl-1-(*p*-tolyl)ethyl)malonate (4g)

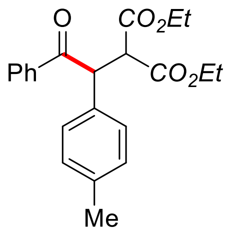

According to **General procedure E**, DHQZ **1a** (49.2 mg, 0.15 mmol, 1.5 equiv) and **2h** (26.2 mg, 0.1 mmol, 1.0 equiv) were used to obtain crude residue, which was purified by silica gel column chromatography (hexane/ethyl acetate 20:1, v/v) to afford the corresponding product **4g** as yellow gum (23.6 mg, 0.064 mmol, 64%).

^{1}H NMR (300 MHz, $CDCl_3$) δ 7.99 (d, J = 7.2 Hz, 2H), 7.50 – 7.45 (m, 1H), 7.40 – 7.35 (m, 2H), 7.19 (d, J = 8.1 Hz, 2H), 7.06 (d, J = 8.0 Hz, 2H), 5.28 (d, J = 11.4 Hz, 1H), 4.42 (d, J = 11.4 Hz, 1H), 4.21 – 4.13 (m, 2H), 3.96 (q, J = 7.1 Hz, 2H), 2.25 (s, 3H), 1.21 (t, J = 7.1 Hz, 3H), 0.99 (t, J = 7.1 Hz, 3H) ppm.

^{13}C{^{1}H} NMR (101 MHz, $CDCl_3$) δ 197.4, 168.2, 168.0, 137.7, 135.9, 133.0, 131.4, 129.7, 128.9, 128.8, 128.5, 61.8, 61.3, 56.0, 52.5, 29.7, 21.0, 13.9, 13.8 ppm.

See NMR spectrum

HRMS (m/z): (EI) calc'd for $C_{22}H_{24}O_5$ $[M]^+$: 368.1618, found: 368.1617

IR (ATR) ν_{max}: 2921, 2853, 1732, 1682, 1284, 1178 and 1034 cm^{-1}

TLC: R_f = 0.25 (hexane/ethyl acetate 8:1, v/v)

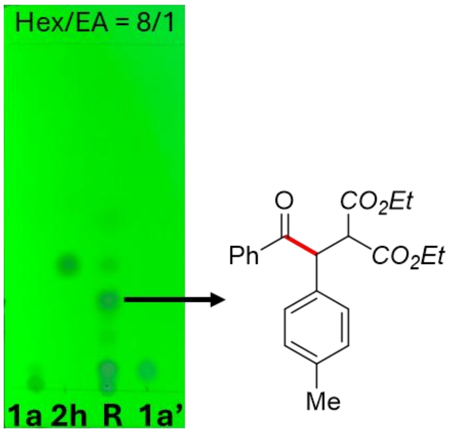

Diethyl 2-(2-oxo-2-phenyl-1-(*o*-tolyl)ethyl)malonate (4h)

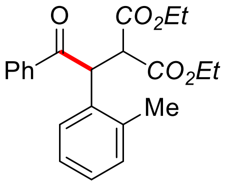

According to **General procedure E**, DHQZ **1a** (49.2 mg, 0.15 mmol, 1.5 equiv) and **2i** (26.2 mg, 0.1 mmol, 1.0 equiv) were used to obtain a crude residue, which was purified by silica gel column chromatography (hexane/ethyl acetate 25:1, v/v) to afford the corresponding product **4h** as a yellow oil (25.8 mg, 0.070 mmol, 70%).

^{1}H NMR (300 MHz, $CDCl_3$) δ 7.90 (d, J = 7.1 Hz, 2H), 7.49 – 7.45 (m, 1H), 7.40 – 7.35 (m, 2H), 7.25 – 7.23 (m, 1H), 7.10 (d, J = 3.2 Hz, 3H), 5.53 (d, J = 11.2 Hz, 1H), 4.48 (d, J = 11.2 Hz, 1H), 4.24 – 4.14 (m, 2H), 3.95 – 3.82 (m, 2H), 2.51 (s, 3H), 1.23 (t, J = 7.1 Hz, 3H), 0.89 (t, J = 7.1 Hz, 3H) ppm.

^{13}C{^{1}H} NMR (101 MHz, $CDCl_3$) δ 198.5, 168.4, 168.3, 136.7, 136.5, 132.9, 132.9, 132.6, 131.2, 128.5, 128.5, 127.9, 126.6, 61.8, 61.3, 19.9, 13.9, 13.6 ppm.

See NMR spectrum

HRMS (m/z): (EI) calc'd for $C_{22}H_{24}O_5$ $[M]^+$: 368.1618, found: 368.1620

IR (ATR) ν_{max}: 3078, 2985, 1731, 1681, 1280, 1172 and 756 cm^{-1}

TLC: R_f = 0.29 (hexane/ethyl acetate 8:1, v/v)

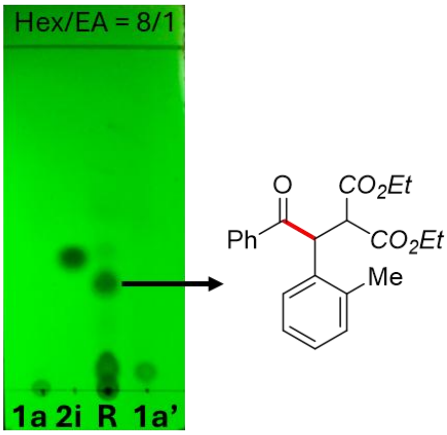

Diethyl 2-(1-(4-methoxyphenyl)-2-oxo-2-phenylethyl)malonate (4i)

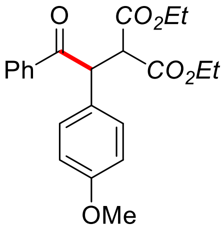

According to **General procedure E**, DHQZ **1a** (49.2 mg, 0.15 mmol, 1.5 equiv) and **2j** (27.8 mg, 0.1 mmol, 1.0 equiv) were used to obtain a crude residue, which was purified by silica gel column chromatography (hexane/ethyl acetate 18:1, v/v) to afford the corresponding product **4i** as a yellow oil (21.5 mg, 0.056 mmol, 56%).

^{1}H NMR (300 MHz, $CDCl_3$) δ 7.98 (d, J = 8.1 Hz, 2H), 7.50 – 7.45 (m, 1H), 7.41 – 7.35 (m, 2H), 7.22 (d, J = 8.7 Hz, 2H), 6.79 (d, J = 8.7 Hz, 2H), 5.27 (d, J = 11.4 Hz, 1H), 4.41 (d, J = 11.4 Hz, 1H), 4.22 – 4.11 (m, 2H), 3.96 (q, J = 7.1 Hz, 2H), 3.72 (s, 3H), 1.21 (t, J = 7.1 Hz, 3H), 1.01 (t, J = 7.1 Hz, 3H) ppm.

^{13}C{^{1}H} NMR (101 MHz, $CDCl_3$) δ 197.5, 168.2, 168.1, 159.3, 135.9, 133.0, 130.0, 128.8, 128.5, 126.3, 114.4, 61.8, 61.4, 55.9, 55.2, 52.0, 13.9, 13.8 ppm.

See NMR spectrum

TLC: R_f = 0.25 (hexane/ethyl acetate 6:1, v/v)

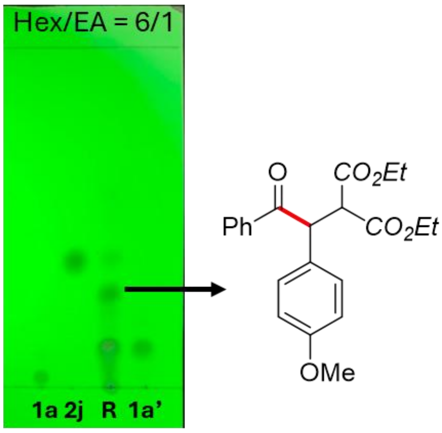

All other data matches what was reported in the literature.[30]

Diethyl 2-(1-(3-methoxyphenyl)-2-oxo-2-phenylethyl)malonate (4j)

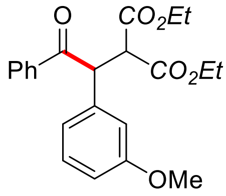

According to **General procedure E**, DHQZ **1a** (49.2 mg, 0.15 mmol, 1.5 equiv) and **2k** (27.8 mg, 0.1 mmol, 1.0 equiv) were used to obtain crude residue, which was purified by silica gel column chromatography (hexane/ethyl acetate 18:1, v/v) to afford the corresponding product **4j** as yellow oil (24.2 mg, 0.063 mmol, 63%).

^{1}H NMR (300 MHz, $CDCl_3$) δ 7.99 (d, J = 7.3 Hz, 2H), 7.50 – 7.46 (m, 1H), 7.41 – 7.36 (m, 2H), 7.18 (t, J = 7.9 Hz, 1H), 6.90 (d, J = 7.6 Hz, 1H), 6.84 (s, 1H), 6.76 – 6.73 (m, 1H), 5.28 (d, J = 11.4 Hz, 1H), 4.42 (d, J = 11.4 Hz, 1H), 4.17 (q, J = 7.1 Hz, 2H), 3.97 (q, J = 7.2 Hz, 2H), 3.74 (s, 3H), 1.21 (t, J = 7.1 Hz, 3H), 1.00 (t, J = 7.1 Hz, 3H) ppm.

^{13}C{^{1}H} NMR (101 MHz, $CDCl_3$) δ 197.2, 168.1, 168.0, 159.9, 135.9, 135.9, 133.1, 130.0, 128.9, 128.5, 121.3, 114.4, 113.4, 61.9, 61.4, 55.9, 55.2, 52.9, 13.9, 13.8 ppm.

See NMR spectrum

HRMS (m/z): (EI) calc'd for $C_{22}H_{24}O_6$ $[M]^+$: 384.1567, found: 384.1567

IR (ATR) ν_{max}: 2982, 2933, 2837, 1730, 1263, 1178 and 693 cm^{-1}

TLC: R_f = 0.28 (hexane/ethyl acetate 5:1, v/v)

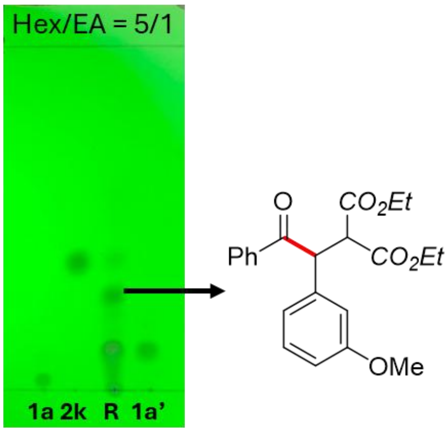

Diethyl 2-(1-(2-methoxyphenyl)-2-oxo-2-phenylethyl)malonate (4k)

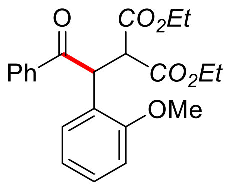

According to **General procedure E**, DHQZ **1a** (49.2 mg, 0.15 mmol, 1.5 equiv) and **2l** (27.8 mg, 0.1 mmol, 1.0 equiv) were used to obtain a crude residue, which was purified by silica gel column chromatography (hexane/ethyl acetate 18:1, v/v) to afford the corresponding product **4k** as yellow oil (19.9 mg, 0.052 mmol, 52%).

^{1}H NMR (400 MHz, $CDCl_3$) δ 8.03 – 8.00 (m, 2H), 7.47 – 7.43 (m, 1H), 7.36 – 7.33 (m, 2H), 7.23 (dd, J = 7.7, 1.7 Hz, 1H), 7.17 (ddd, J = 8.2, 7.4, 1.7 Hz, 1H), 6.88 – 6.80 (m, 2H), 5.78 (d, J = 11.3 Hz, 1H), 4.43 (d, J = 11.3 Hz, 1H), 4.25 – 4.13 (m, 2H), 3.95 – 3.88 (m, 2H), 3.87 (s, 3H), 1.22 (t, J = 7.1 Hz, 3H), 0.95 (t, J = 7.1 Hz, 3H) ppm.

^{13}C{^{1}H} NMR (101 MHz, $CDCl_3$) δ 197.8, 168.5, 168.2, 156.7, 135.9, 132.9, 129.7, 129.2, 128.7, 128.3, 123.2, 121.0, 111.2, 61.6, 61.1, 55.5, 54.8, 45.7, 13.9, 13.6 ppm.

See NMR spectrum

HRMS (m/z): (ESI) calc'd for $C_{22}H_{24}O_6{}^{23}Na$ $[M+Na]^+$: 407.1465, found: 407.1463

TLC: R_f = 0.20 (hexane/ethyl acetate 5:1, v/v)

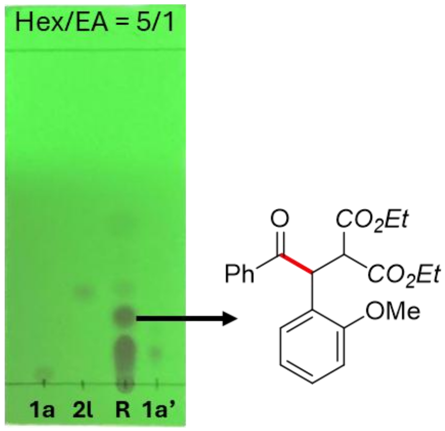

Diethyl 2-(1-(4-(ethoxycarbonyl)phenyl)-2-oxo-2-phenylethyl)malonate (4l)

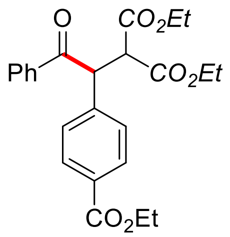

According to **General procedure E**, DHQZ **1a** (49.2 mg, 0.15 mmol, 1.5 equiv) and **2m** (32.0 mg, 0.1 mmol, 1.0 equiv) were used to obtain a crude residue, which was purified by silica gel column chromatography (hexane/ethyl acetate 18:1, v/v) to afford the corresponding product **4l** as colorless oil (31.1 mg, 0.073 mmol, 73%).

^{1}H NMR (300 MHz, $CDCl_3$) δ 7.97 – 7.93 (m, 4H), 7.48 (t, J = 7.3 Hz, 1H), 7.41 – 7.35 (m, 4H), 5.38 (d, J = 11.4 Hz, 1H), 4.45 (d, J = 11.4 Hz, 1H), 4.31 (q, J = 7.1 Hz, 2H), 4.17 (qd, J = 7.1, 1.9 Hz, 2H), 3.94 (qd, J = 7.1, 1.6 Hz, 2H), 1.33 (t, J = 7.1 Hz, 3H), 1.21 (t, J = 7.1 Hz, 3H), 0.98 (t, J = 7.1 Hz, 3H) ppm.

^{13}C{^{1}H} NMR (101 MHz, $CDCl_3$) δ 196.8, 167.9, 167.7, 166.0, 139.6, 135.7, 133.3, 130.2, 130.1, 129.0, 128.8, 128.6, 62.0, 61.5, 61.0, 55.7, 52.7, 14.2, 13.9, 13.8 ppm.

See NMR spectrum

HRMS (m/z): (EI) calc'd for $C_{24}H_{26}O_7{}^{23}Na$ $[M+Na]^+$: 449.1571, found: 449.1567

IR (ATR) ν_{max}: 2983, 2939, 1718, 1682, 1580, 1447, 1277, 1178, 1106 and 693 cm^{-1}

TLC: R_f = 0.12 (hexane/ethyl acetate 8:1, v/v)

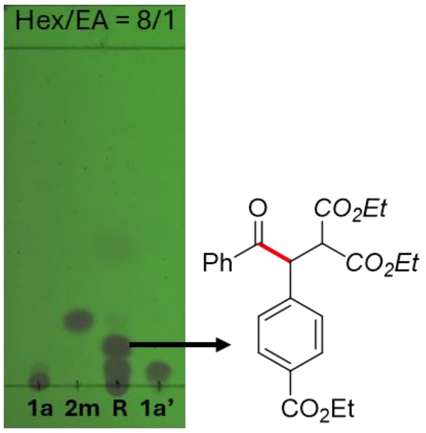

Back to contents/Back to table

Diethyl 2-(1-(4-cyanophenyl)-2-oxo-2-phenylethyl)malonate (4m)

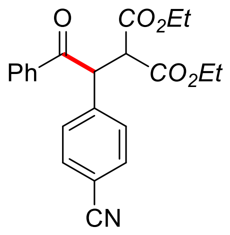

According to **General procedure E**, DHQZ **1a** (49.2 mg, 0.15 mmol, 1.5 equiv) and **2n** (27.3 mg, 0.1 mmol, 1.0 equiv) were used to obtain a crude residue, which was purified by silica gel column chromatography (hexane/ethyl acetate 15:1, v/v) to afford the corresponding product **4m** as a colorless liquid (29.2 mg, 0.077 mmol, 77%).

^{1}H NMR (300 MHz, $CDCl_3$) δ 7.96 (d, J = 7.1 Hz, 2H), 7.59 – 7.39 (m, 7H), 5.39 (d, J = 11.4 Hz, 1H), 4.44 (d, J = 11.4 Hz, 1H), 4.23 – 4.13 (m, 2H), 4.02 – 3.92 (m, 2H), 1.21 (t, J = 7.1 Hz, 3H), 1.01 (t, J = 7.1 Hz, 3H) ppm.

^{13}C{^{1}H} NMR (101 MHz, $CDCl_3$) δ 196.5, 167.6, 167.5, 140.0, 135.5, 133.7, 132.7, 129.8, 128.8, 118.2, 112.1, 62.2, 61.7, 55.6, 52.4, 13.8 ppm.

See NMR spectrum

HRMS (m/z): (EI) calc'd for $C_{22}H_{21}NO_5$ $[M]^+$: 379.1414, found: 379.1416

IR (ATR) ν_{max}: 2980, 2928, 2230, 1728, 1682, 1284 and 1181 cm^{-1}

TLC: R_f = 0.26 (hexane/ethyl acetate 5:1, v/v)

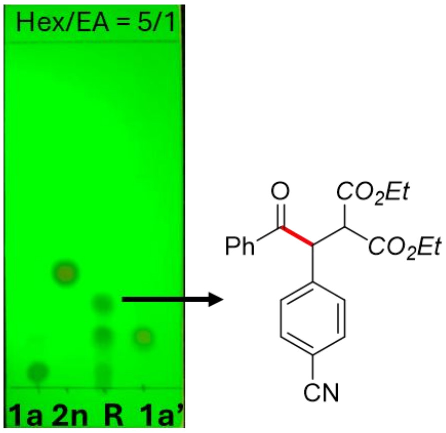

Diethyl 2-(2-oxo-2-phenyl-1-(4-(trifluoromethyl)phenyl)ethyl)malonate (4n)

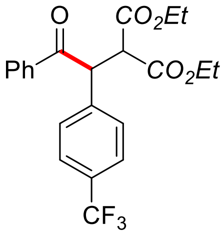

According to **General procedure E**, DHQZ **1a** (49.2 mg, 0.15 mmol, 1.5 equiv) and **2o** (31.6 mg, 0.1 mmol, 1.0 equiv) were used to obtain a crude residue, which was purified by silica gel column chromatography (hexane/ethyl acetate 25:1, v/v) to afford the corresponding product **4n** as a yellow oil (28.3 mg, 0.067 mmol, 67%).

^{1}**H NMR** (300 MHz, $CDCl_3$) δ 7.98 (d, J = 7.9 Hz, 2H), 7.55 – 7.39 (m, 7H), 5.40 (d, J = 11.4 Hz, 1H), 4.45 (d, J = 11.4 Hz, 1H), 4.18 (q, J = 7.2 Hz, 2H), 3.96 (q, J = 7.2 Hz, 2H), 1.22 (t, J = 7.1 Hz, 3H), 0.96 (t, J = 6.9 Hz, 3H) ppm.

^{13}**C{^{1}H} NMR** (101 MHz, $CDCl_3$) δ 196.8, 167.8, 167.7, 138.7, 135.6, 133.5, 129.4, 128.9, 128.7, 125.9, 123.8 (q, J = 282.8 Hz), 62.1, 61.6, 55.8, 52.4, 13.9, 13.7 ppm.

^{19}**F NMR** (282 MHz, $CDCl_3$) δ -62.8 ppm.

See NMR spectrum

HRMS (m/z): (EI) calc'd for $C_{22}H_{21}O_5F_3$ $[M]^+$: 422.1336, found: 422.1339

IR (ATR) ν_{max}: 2983, 2936, 1731, 1682, 1323, 1125, 1068 and 700 cm^{-1}

TLC: R_f = 0.26 (hexane/ethyl acetate 8:1, v/v)

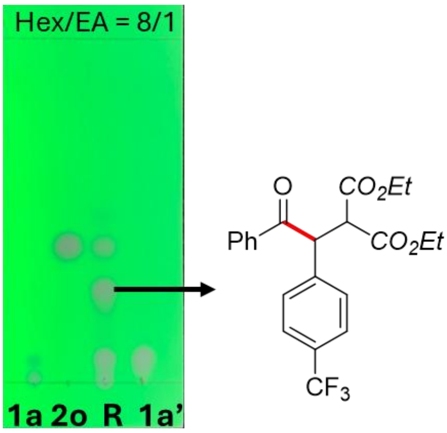

Diethyl 2-(1-(4-hydroxyphenyl)-2-oxo-2-phenylethyl)malonate (4o)

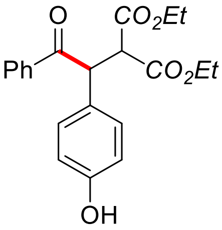

According to **General procedure E**, DHQZ **1a** (49.2 mg, 0.15 mmol, 1.5 equiv) and **2p** (26.4 mg, 0.1 mmol, 1.0 equiv) were used to obtain crude residue, which was purified by silica gel column chromatography (hexane/ethyl acetate 10:1, v/v) to afford the corresponding product **4o** as yellow solid (21.9 mg, 0.059 mmol, 59%).

^{1}H NMR (300 MHz, $CDCl_3$) δ 7.97 (d, J = 8.0 Hz, 2H), 7.51 – 7.46 (m, 1H), 7.40 – 7.36 (m, 2H), 7.16 (d, J = 8.5 Hz, 2H), 6.70 (d, J = 8.5 Hz, 2H), 5.25 (d, J = 11.4 Hz, 1H), 4.40 (d, J = 11.4 Hz, 1H), 4.21 – 4.11 (m, 2H), 3.97 (q, J = 7.1 Hz, 2H), 1.20 (t, J = 7.1 Hz, 3H), 1.02 (t, J = 7.1 Hz, 3H) ppm.

^{13}C{^{1}H} NMR (101 MHz, $CDCl_3$) δ 197.5, 168.2, 168.2, 155.4, 135.9, 133.1, 130.2, 128.9, 128.5, 126.4, 115.9, 61.9, 61.5, 56.0, 52.0, 13.9, 13.8 ppm.

See NMR spectrum

HRMS (m/z): (EI) calc'd for $C_{21}H_{22}O_6$ $[M]^+$: 370.1411, found: 370.1412

IR (ATR) ν_{max}: 3424, 2982, 1732, 1513, 1277, 1175 and 1031 cm^{-1}

Melting point: 102 – 105 ˚C

TLC: R_f = 0.18 (hexane/ethyl acetate 3:1, v/v)

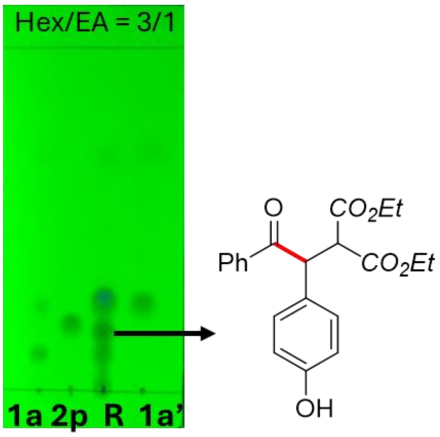

Diethyl 2-(1-(4-fluorophenyl)-2-oxo-2-phenylethyl)malonate (4p)

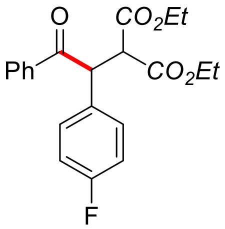

According to **General procedure E**, DHQZ **1a** (49.2 mg, 0.15 mmol, 1.5 equiv) and **2q** (26.6 mg, 0.1 mmol, 1.0 equiv) were used to obtain a crude residue, which was purified by silica gel column chromatography (hexane/ethyl acetate 20:1, v/v) to afford the corresponding product **4p** as a yellow oil (20.9 mg, 0.056 mmol, 56%).

^{1}H NMR (300 MHz, $CDCl_3$) δ 7.98 – 7.95 (m, 2H), 7.50 (tt, J = 7.3, 1.2 Hz, 1H), 7.42 – 7.37 (m, 2H), 7.32 – 7.27 (m, 2H), 7.00 – 6.92 (m, 2H), 5.31 (d, J = 11.4 Hz, 1H), 4.41 (d, J = 11.4 Hz, 1H), 4.22 – 4.12 (m, 2H) , 3.97 (q, J = 7.1 Hz, 2H), 1.21 (t, J = 7.1 Hz, 3H), 1.00 (t, J = 7.1 Hz, 3H) ppm.

^{13}C{^{1}H} NMR (101 MHz, $CDCl_3$) δ 197.3, 168.0, 167.9, 162.4 (d, J = 248.7 Hz), 135.7, 133.3, 130.6 (d, J = 8.3 Hz), 130.3 (d, J = 3.3 Hz), 128.8, 128.6, 116.0 (d, J = 21.7 Hz), 62.0, 61.5, 55.9, 51.9, 13.9, 13.8 ppm.

^{19}F NMR (282 MHz, $CDCl_3$) δ -113.9

See NMR spectrum

HRMS (m/z): (EI) calc'd for $C_{21}H_{21}O_5F$ $[M]^+$: 372.1368, found: 372.1366

IR (ATR) ν_{max}: 2982, 2938, 1730, 1681, 1284, 1032 and 691 cm^{-1}

TLC: R_f= 0.24 (hexane/ethyl acetate 8:1, v/v)

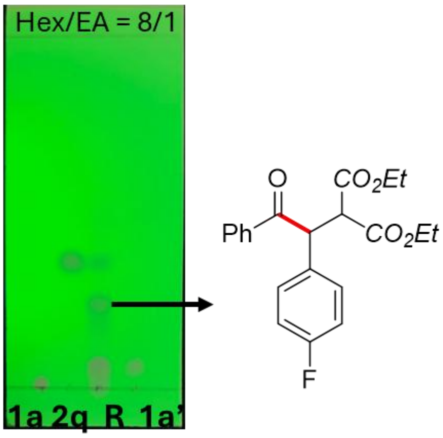

Diethyl 2-(1-(4-chlorophenyl)-2-oxo-2-phenylethyl)malonate (4q)

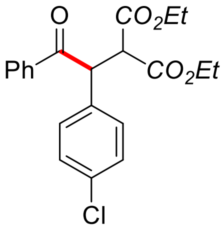

According to **General procedure E**, DHQZ **1a** (49.2 mg, 0.15 mmol, 1.5 equiv) and **2r** (28.3 mg, 0.1 mmol, 1.0 equiv) were used to obtain a crude residue, which was purified by preparative TLC (hexane/ethyl acetate 20:1, v/v) to afford the corresponding product **4q** as a yellow oil (24.4 mg, 0.063 mmol, 63%).

^{1}H NMR (300 MHz, $CDCl_3$) δ 7.97 (d, J = 7.2 Hz, 2H), 7.53 – 7.48 (m, 1H), 7.43 – 7.38 (m, 2H), 7.29 – 7.23 (m, 4H), 5.31 (d, J = 11.4 Hz, 1H), 4.41 (d, J = 11.4 Hz, 1H), 4.22 – 4.14 (m, 2H), 3.98 (q, J = 7.1 Hz, 2H), 1.21 (t, J = 7.1 Hz, 3H), 1.02 (t, J = 7.1 Hz, 3H) ppm.

^{13}C{^{1}H} NMR (101 MHz, $CDCl_3$) δ 197.0, 167.9, 167.8, 135.6, 134.1, 133.3, 133.0, 130.2, 129.2, 128.8, 128.6, 62.0, 61.5, 55.8, 52.0, 13.9, 13.8 ppm.

See NMR spectrum

HRMS (m/z): (ESI) calc'd for $C_{21}H_{21}O_5{}^{35}Cl^{23}Na$ $[M+Na]^+$: 411.0970, found: 411.0968

IR (ATR) ν_{max}: 3060, 2982, 2851, 1731, 1682, 1284, 1178 and 722 cm^{-1}

TLC: R_f = 0.32 (hexane/ethyl acetate 8:1, v/v)

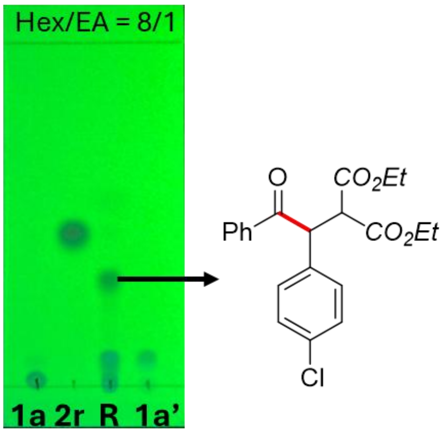

Back to contents/Back to table

Diethyl 2-(1-(4-bromophenyl)-2-oxo-2-phenylethyl)malonate (4r)

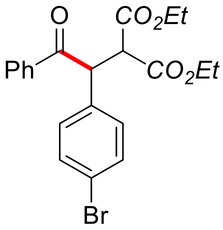

According to **General procedure E**, DHQZ **1a** (49.2 mg, 0.15 mmol, 1.5 equiv) and **2s** (32.7 mg, 0.1 mmol, 1.0 equiv) were used to obtain crude residue, which was purified by silica gel column chromatography (hexane/ethyl acetate 18:1, v/v) to afford the corresponding product **4r** as a yellow oil (33.4 mg, 0.077 mmol, 77%).

^{1}H NMR (300 MHz, $CDCl_3$) δ 7.96 (d, J = 7.6 Hz, 2H), 7.52 – 7.48 (m, 1H), 7.42 – 7.37 (m, 4H), 7.21 – 7.18 (m, 2H), 5.29 (d, J = 11.4 Hz, 1H), 4.40 (d, J = 11.4 Hz, 1H), 4.22 – 4.11 (m, 2H) , 3.97 (q, J = 7.1 Hz, 2H), 1.21 (t, J = 7.1 Hz, 3H), 1.01 (t, J = 7.1 Hz, 3H) ppm.

^{13}C{^{1}H} NMR (101 MHz, $CDCl_3$) δ 197.0, 167.9, 167.8, 135.6, 133.6, 133.3, 132.2, 130.6, 128.8, 128.6, 122.2, 62.0, 61.5, 55.7, 52.1, 13.9, 13.8 ppm.

See NMR spectrum

HRMS (m/z): (EI) calc'd for $C_{21}H_{21}O_5Br$ $[M]^+$: 432.0567, found: 432.0567

IR (ATR) ν_{max}: 2981, 2937, 1728, 1680, 1284, 1178 and 1101 cm^{-1}

TLC: R_f = 0.39 (hexane/ethyl acetate 6:1, v/v)

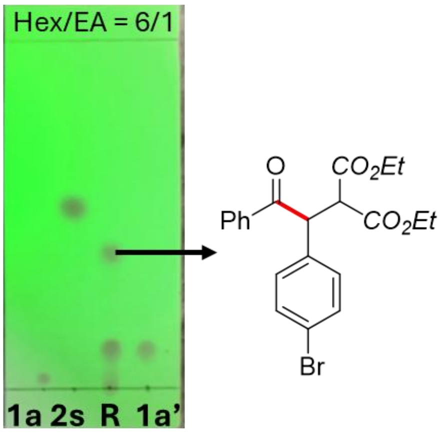

Diethyl 2-(1-(naphthalen-2-yl)-2-oxo-2-phenylethyl)malonate (4s)

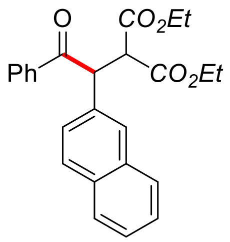

According to **General procedure E**, DHQZ **1a** (49.2 mg, 0.15 mmol, 1.5 equiv) and **2t** (29.8 mg, 0.1 mmol, 1.0 equiv) were used to obtain a crude residue, which was purified by silica gel column chromatography (hexane/ethyl acetate 20:1, v/v) to afford the corresponding product **4s** as yellow oil (28.3 mg, 0.070 mmol, 70%).

^{1}H NMR (300 MHz, $CDCl_3$) δ 8.03 (d, J = 7.5 Hz, 2H), 7.79 – 7.74 (m, 4H), 7.45 – 7.43 (m, 4H), 7.37 (t, J = 7.4 Hz, 2H), 5.51 (d, J = 11.4 Hz, 1H), 4.56 (d, J = 11.4 Hz, 1H), 4.21 (q, J = 7.1, 6.5 Hz, 2H), 3.88 (qq, J = 7.1, 3.7 Hz, 2H), 1.24 (t, J = 7.2 Hz, 3H), 0.84 (t, J = 7.1 Hz, 3H) ppm.

^{13}C{^{1}H} NMR (101 MHz, $CDCl_3$) δ 197.3, 168.2, 168.0, 135.9, 133.4, 133.1, 132.8, 132.0, 128.9, 128.8, 128.5, 128.4, 127.9, 127.6, 126.3, 61.9, 61.4, 56.0, 53.0, 13.9, 13.6 ppm.

See NMR spectrum

HRMS (m/z): (ESI) calc'd for $C_{25}H_{24}O_5{}^{23}Na$ $[M+Na]^+$: 427.1516, found: 427.1513

IR (ATR) ν_{max}: 3057, 2980, 2936, 2847, 1730, 1679, 1447, 1284, 1181 and 688 cm^{-1}

TLC: R_f = 0.18 (hexane/ethyl acetate 8:1, v/v)

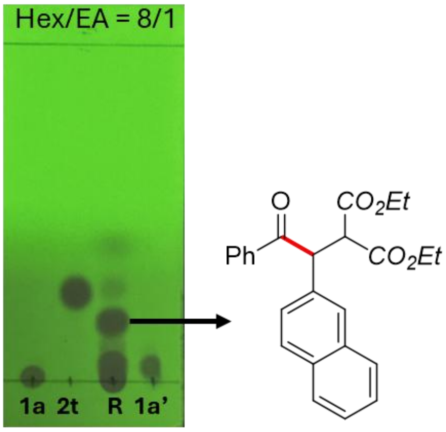

Diethyl 2-(1-([1,1'-biphenyl]-4-yl)-2-oxo-2-phenylethyl)malonate (4t)

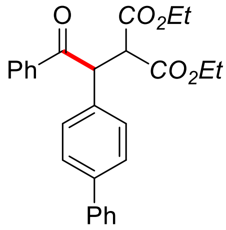

According to **General procedure E**, DHQZ **1a** (49.2 mg, 0.15 mmol, 1.5 equiv) and **2u** (32.4 mg, 0.1 mmol, 1.0 equiv) were used to obtain a crude residue, which was purified by preparative TLC (hexane/ethyl acetate 13:1, v/v) to afford the corresponding product **4t** as a colorless gum (28.0 mg, 0.065 mmol, 65%).

^{1}H NMR (400 MHz, $CDCl_3$) δ 8.04 (d, J = 8.2 Hz, 2H), 7.52 – 7.48 (m, 5H), 7.43 – 7.39 (m, 6H), 7.34 – 7.30 (m, 1H), 5.38 (d, J = 11.4 Hz, 1H), 4.49 (d, J = 11.4 Hz, 1H), 4.23 – 4.15 (m, 2H), 4.01 – 3.93 (m, 2H), 1.23 (t, J = 7.1 Hz, 3H), 0.97 (t, J = 7.1 Hz, 3H) ppm.

^{13}C{^{1}H} NMR (101 MHz, $CDCl_3$) δ 197.3, 168.1, 168.1, 140.8, 140.2, 135.9, 133.4, 133.2, 129.3, 128.9, 128.7, 128.6, 127.6, 127.4, 126.9, 61.9, 61.4, 55.9, 52.5, 13.9, 13.7 ppm.

See NMR spectrum

HRMS (m/z): (ESI) calc'd for $C_{27}H_{26}O_5{}^{23}Na$ $[M+Na]^+$: 453.1673, found: 453.1670

IR (ATR) ν_{max}: 2981, 1731, 1680, 1280, 1176 and 690 cm^{-1}

TLC: R_f = 0.28 (hexane/ethyl acetate 5:1, v/v)

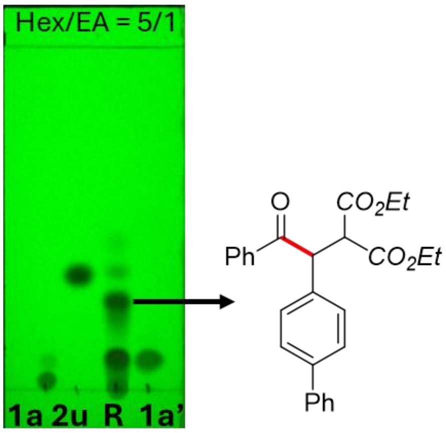

Back to contents/Back to table

Diethyl 2-(2-oxo-2-phenyl-1-(thiophen-2-yl)ethyl)malonate (4u)

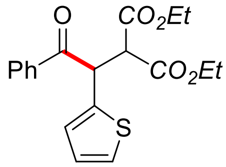

According to **General procedure E**, DHQZ **1a** (49.2 mg, 0.15 mmol, 1.5 equiv) and **2v** (25.4 mg, 0.1 mmol, 1.0 equiv) were used to obtain a crude residue, which was purified by silica gel column chromatography (hexane/ethyl acetate 15:1, v/v) to afford the corresponding product **4u** as brown gum (22.7 mg, 0.063 mmol, 63%).

^{1}H NMR (300 MHz, $CDCl_3$) δ 8.03 (d, J = 7.3 Hz, 2H), 7.55 – 7.50 (m, 1H), 7.42 (t, J = 7.4 Hz, 2H), 7.18 (d, J = 5.4 Hz, 1H), 6.96 – 6.95 (m, 1H), 6.90 – 6.87 (m, 1H), 5.61 (d, J = 11.3 Hz, 1H), 4.46 (d, J = 11.3 Hz, 1H), 4.20 – 4.12 (m, 2H), 4.10 – 4.02 (m, 2H), 1.20 (t, J = 7.1 Hz, 3H), 1.09 (t, J = 7.1 Hz, 3H) ppm.

^{13}C{^{1}H} NMR (101 MHz, $CDCl_3$) δ 196.2, 167.8, 136.2, 135.5, 133.3, 128.9, 128.6, 127.6, 127.2, 126.2, 62.0, 61.6, 56.5, 47.3, 13.9, 13.8 ppm.

See NMR spectrum

TLC: R_f= 0.21 (hexane/ethyl acetate 6:1, v/v)

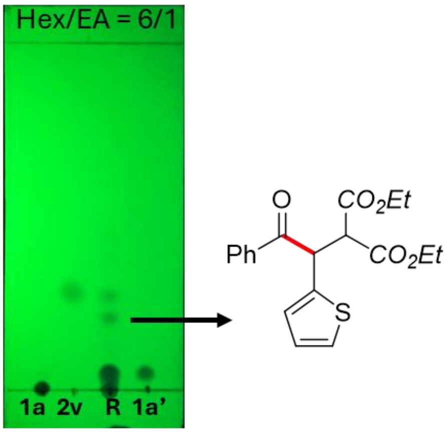

All other data matches what was reported in the literature.[30]

Diethyl 2-(1-(1-methyl-1*H*-pyrrol-2-yl)-2-oxo-2-phenylethyl)malonate (4v)

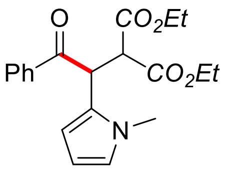

According to **General procedure E**, DHQZ **1a** (49.2 mg, 0.15 mmol, 1.5 equiv) and **2w** (25.1 mg, 0.1 mmol, 1.0 equiv) were used to obtain a crude residue, which was purified by silica gel column chromatography (hexane/ethyl acetate 25:1, v/v) to afford the corresponding product **4v** as brown gum (5.0 mg, 0.014 mmol, 14%).

^{1}H NMR (300 MHz, $CDCl_3$) δ 7.88 (d, J = 7.0 Hz, 2H), 7.49 (t, J = 7.3 Hz, 1H), 7.39 (t, J = 7.4 Hz, 2H), 6.47 (t, J = 2.2 Hz, 1H), 6.05 – 5.97 (m, 2H), 5.36 (d, J = 11.2 Hz, 1H), 4.48 (d, J = 11.2 Hz, 1H), 4.18 (qd, J = 7.1, 1.5 Hz, 2H), 4.01 (qd, J = 7.1, 2.4 Hz, 2H), 3.65 (s, 3H), 1.25 – 1.20 (m, 3H), 1.05 (t, J = 7.1 Hz, 3H) ppm.

^{13}C{^{1}H} NMR (101 MHz, $CDCl_3$) δ 196.0, 168.4, 168.2, 136.5, 133.5, 132.9, 128.5, 124.6, 123.6, 110.2, 107.9, 61.9, 61.5, 54.9, 44.9, 34.1, 29.7, 13.9, 13.8 ppm.

See NMR spectrum

HRMS (m/z): (ESI) calc'd for $C_{20}H_{23}NO_5{}^{23}Na$ $[M+Na]^+$: 380.1468, found: 380.1466

TLC: R_f = 0.20 (hexane/ethyl acetate 8:1, v/v)

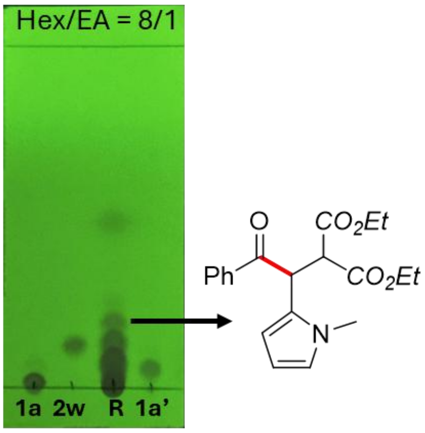

3-Acetyl-1,2-diphenylpentane-1,4-dione (4w)

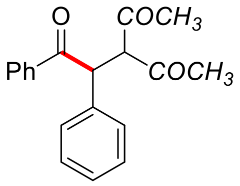

According to **General procedure E**, DHQZ **1a** (49.2 mg, 0.15 mmol, 1.5 equiv) and **2x** (18.8 mg, 0.1 mmol, 1.0 equiv) were used to obtain a crude residue, which was purified by silica gel column chromatography (hexane/ethyl acetate 8:1, v/v) to afford the corresponding product **4w** as colorless oil (15.2 mg, 0.052 mmol, 52%).

^{1}H NMR (300 MHz, $CDCl_3$) δ 7.94 (d, J = 7.3 Hz, 2H), 7.47 (t, J = 7.3 Hz, 1H), 7.36 (t, J = 7.5 Hz, 2H), 7.28 – 7.18 (m, 5H), 5.37 (d, J = 11.1 Hz, 1H), 4.86 (d, J = 11.1 Hz, 1H), 2.28 (s, 3H), 1.92 (s, 3H) ppm.

^{13}C{^{1}H} NMR (101 MHz, $CDCl_3$) δ 203.1, 201.2, 197.8, 135.7, 135.1, 133.2, 129.4, 128.9, 128.7, 128.5, 128.0, 71.3, 59.1, 53.8, 31.7, 30.2 ppm.

See NMR spectrum

TLC: R_f = 0.12 (hexane/ethyl acetate 8:1, v/v)

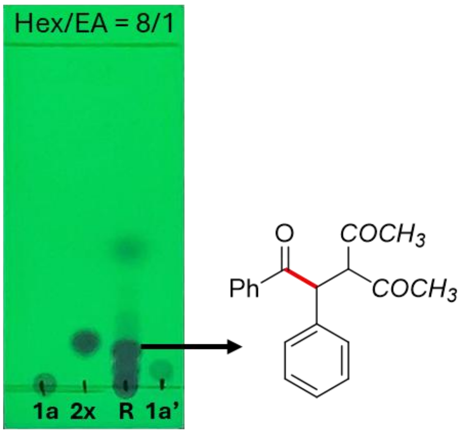

All other data matches what was reported in the literature.[31]

2-(2-Oxo-1,2-diphenylethyl)-1*H*-indene-1,3(2*H*)-dione (4x)

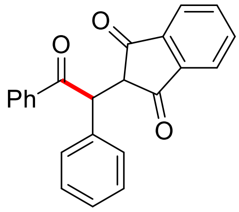

According to **General procedure E**, DHQZ **1a** (98.4 mg, 0.30 mmol, 3.0 equiv) and **2y** (23.4 mg, 0.1 mmol, 1.0 equiv) was used to obtain a crude residue, which was purified by silica gel column chromatography (hexane/ethyl acetate 15:1, v/v) to afford the corresponding product **4x** as a yellow solid (28.9 mg, 0.085 mmol, 85%).

^{1}H NMR (400 MHz, $CDCl_3$) δ 8.02 (d, J = 7.6 Hz, 1H), 7.94 (d, J = 7.0 Hz, 1H) 7.87 – 7.76 (m, 4H), 7.45 – 7.41 (m, 1H), 7.37 – 7.24 (m, 7H), 5.57 (d, J = 3.5 Hz, 1H), 3.41 (d, J = 3.5 Hz, 1H) ppm.

^{13}C{^{1}H} NMR (101 MHz, $CDCl_3$) δ 199.1, 198.2, 197.5, 143.0, 141.0, 136.6, 135.6, 134.8, 134.8, 133.3, 129.7, 129.4, 128.8, 128.4, 127.7, 123.1, 123.0, 55.7, 55.5, 29.7 ppm.

See NMR spectrum

TLC: R_f = 0.29 (hexane/ethyl acetate 8:2, v/v)

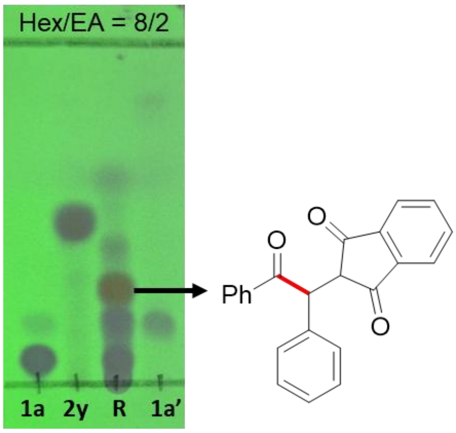

All other data matches what was reported in the literature.[29]

2-(2-Oxo-1,2-diphenylethyl)malononitrile (4y)

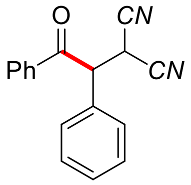

According to **General procedure E**, DHQZ **1a** (49.2 mg, 0.15 mmol, 1.5 equiv) and **2z** (15.4 mg, 0.1 mmol, 1.0 equiv) were used to obtain a crude residue, which was purified by preparative TLC (hexane/ethyl acetate 7:1, v/v) to afford the corresponding product **4y** as a yellow oil (15.6 mg, 0.060 mmol, 60%).

^{1}H NMR (300 MHz, $CDCl_3$) δ 7.90 (d, J = 7.4 Hz, 2H), 7.57 – 7.52 (m, 1H), 7.43 – 7.34 (m, 7H), 5.12 (d, J = 8.3 Hz, 1H), 4.54 (d, J = 8.4 Hz, 1H) ppm.

^{13}C{^{1}H} NMR (101 MHz, $CDCl_3$) δ 193.0, 134.4, 133.8, 132.0, 130.0, 129.8, 129.2, 128.9, 128.6, 112.1, 111.5, 54.8, 26.8 ppm.

See NMR spectrum

TLC: R_f = 0.25 (hexane/ethyl acetate 7:2, v/v)

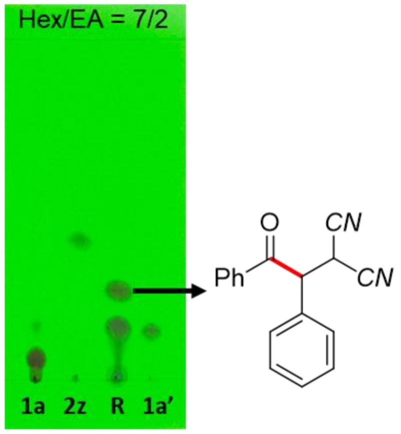

All other data matches what was reported in the literature.[29]

Ethyl 3-benzoyl-2-cyano-4-methylpentanoate (4z)

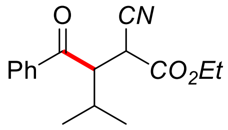

According to **General procedure E**, DHQZ **1a** (49.2 mg, 0.15 mmol, 1.5 equiv) and **2aa** (16.7 mg, 0.1 mmol, 1.0 equiv) were used to obtain a crude residue, which was purified by preparative TLC (hexane/ethyl acetate 9:1, v/v) to afford the corresponding product **4z** as white solid (20.5 mg, 0.075 mmol, 75%, 1.2:1 *d.r.*).

^{1}H NMR (300 MHz, $CDCl_3$) δ 7.95 (d, J = 7.2 Hz, 2H), 7.62 – 7.57 (m, 1H), 7.51 – 7.45 (m, 2H), 4.31 – 4.09 (m, 3H), 3.97 – 3.89 (m, 1H), 2.42 – 2.23 (m, 1H), 1.25 – 1.17 (m, 3H), 1.07 (minor, d, J = 6.8 Hz, 3H), 1.02 (major, d, J = 6.9 Hz, 3H), 0.92 (t, J = 7.1 Hz, 3H) ppm.

^{13}C{^{1}H} NMR (101 MHz, $CDCl_3$) δ 200.4, 199.4, 165.7, 165.6, 137.5, 133.5, 128.8, 128.4, 116.0, 116.0, 115.3, 63.2, 50.6, 49.5, 38.1, 37.7, 30.1, 29.3, 20.9, 20.7, 20.4, 18.1, 13.8 ppm. (4 diastereomers)

See NMR spectrum

HRMS (m/z): (ESI) calc'd for $C_{16}H_{19}O_3N^{23}Na$ $[M+Na]^+$: 296.1257, found: 296.1257

IR (ATR) ν_{max}: 3061, 2968, 2938, 2248, 1744, 1677 and 1218 cm^{-1}

Melting point: 78 – 80 °C

TLC: R_f = 0.28 (hexane/ethyl acetate 5:1, v/v)

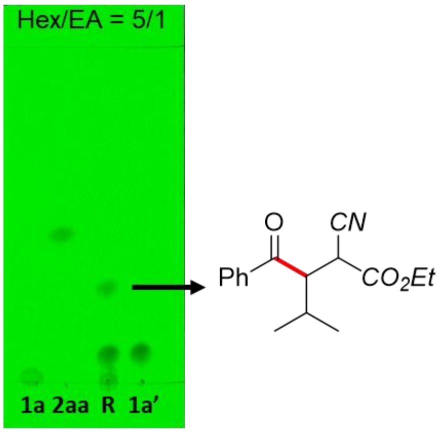

Back to contents/Back to table

Ethyl 2-cyano-4-oxo-3,4-diphenylbutanoate (4aa)

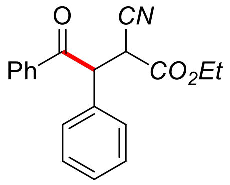

According to **General procedure E**, DHQZ **1a** (49.2 mg, 0.15 mmol, 1.5 equiv) and **2ab** (20.1 mg, 0.1 mmol, 1.0 equiv) was used to obtain a crude residue, which was purified by preparative TLC (hexane/ethyl acetate 12:1, v/v) to afford the corresponding product **4aa** as yellow oil (18.1 mg, 0.059 mmol, 59%, 1.7:1 *d.r.*).

^{1}H NMR (300 MHz, $CDCl_3$) δ 7.94 – 7.89 (m, 2H), 7.53 – 7.47 (m, 1H), 7.41 – 7.29 (m, 7H), 5.29 (minor, d, J = 7.6 Hz, 1H), 5.20 (major, d, J = 10.6 Hz, 1H), 4.54 (major, d, J = 10.3 Hz, 1H), 4.26 (major, q, J = 7.1 Hz, 1H), 4.16 (minor, q, J = 7.1 Hz, 1H), 4.07 (minor, d, J = 7.5 Hz, 1H), 1.29 (major, t, J = 7.1 Hz, 3H), 1.18 (minor, t, J = 7.1 Hz, 3H) ppm.

^{13}C{^{1}H} NMR (101 MHz, $CDCl_3$) δ 195.6 (major), 195.0 (minor), 165.1 (major), 165.1 (minor), 134.9 (major), 134.7 (minor), 133.8 (major), 133.7, 133.4 (minor), 129.6 (major), 129.5 (minor), 129.1 (major), 128.9 (minor), 128.9, 128.8, 128.7, 128.7, 128.6, 115.4 (minor), 115.3 (major), 63.3 (major), 63.0 (minor), 54.0 (minor), 52.9 (major), 41.5 (minor), 41.1 (major), 13.8 (major), 13.8 (minor) ppm.

See NMR spectrum

HRMS (m/z): (ESI) calc'd for $C_{19}H_{17}O_3N^{23}Na$ $[M+Na]^+$: 330.1101, found: 330.1102

IR (ATR) ν_{max}: 2254, 1749, 1683, 1264, 904, 730 and 650 cm^{-1}

TLC: R_f = 0.26 (hexane/ethyl acetate 6:1, v/v)

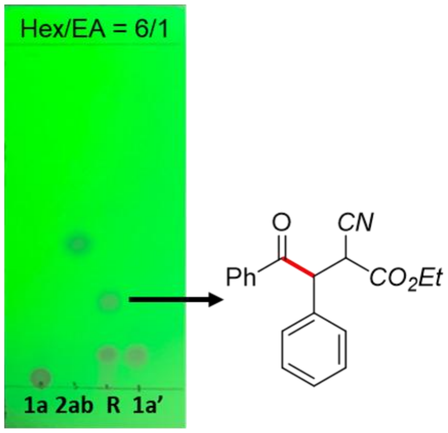

2-(3,5-Di-*tert*-butyl-4-hydroxyphenyl)-1,2-diphenylethan-1-one, (4ab)

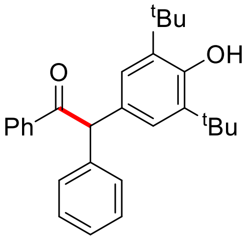

According to **General procedure E**, DHQZ **1a** (98.4 mg, 0.30 mmol, 3.0 equiv) and **2ac** (29.4 mg, 0.1 mmol, 1.0 equiv) was used to obtain a crude residue, which was purified by silica gel column chromatography (hexane/ethyl acetate 15:1, v/v) to afford the corresponding product **4ab** as a yellow solid (35.2 mg, 0.088 mmol, 88%).

^{1}H NMR (300 MHz, $CDCl_3$) δ 8.02 (d, J = 7.2 Hz, 2H), 7.54 – 7.49 (m, 1H), 7.44 – 7.39 (m, 2H), 7.36 – 7.28 (m, 4H), 7.27 – 7.21 (m, 1H), 7.08 (s, 2H), 5.96 (s, 1H), 5.14 (s, 1H), 1.40 (s, 18H) ppm.

^{13}C{^{1}H} NMR (101 MHz, $CDCl_3$) δ 198.8, 152.9, 139.7, 137.3, 135.9, 132.7, 129.4, 129.1, 128.9, 128.6, 128.5, 126.9, 125.8, 59.3, 34.3, 30.3 ppm.

See NMR spectrum

TLC: R_f = 0.58 (hexane/ethyl acetate 6:1, v/v)

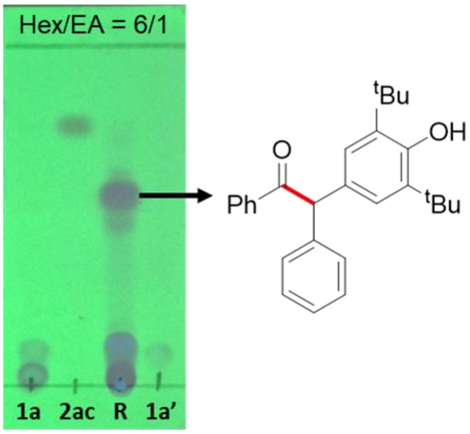

All other data matches what was reported in the literature.[29]

2-(3,5-Di-*tert*-butyl-4-hydroxyphenyl)-1,2-diphenylethan-1-one, (4ac)

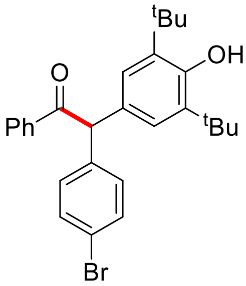

According to **General procedure E**, DHQZ **1a** (98.4 mg, 0.30 mmol, 3.0 equiv) and **2ad** (37.3 mg, 0.1 mmol, 1.0 equiv) was used to obtain a crude residue, which was purified by silica gel column chromatography (hexane/ethyl acetate 20:1, v/v) to afford the corresponding product **4ac** as a yellow solid (33.1 mg, 0.069 mmol, 69%).

^{1}H NMR (400 MHz, $CDCl_3$) δ 7.99 (dd, J = 8.4, 1.4 Hz, 2H), 7.54 – 7.50 (m, 1H), 7.45 – 7.40 (m, 4H), 7.17 (d, J = 8.5 Hz, 2H), 7.05 (s, 2H), 5.89 (s, 1H), 5.15 (s, 1H), 1.39 (s, 18H) ppm.

^{13}C{^{1}H} NMR (101 MHz, $CDCl_3$) δ 198.4, 153.0, 138.9, 137.0, 136.1, 133.0, 131.6, 130.8, 128.9, 128.6, 125.6, 121.0, 58.6, 34.4, 30.2 ppm.

See NMR spectrum

TLC: R_f= 0.55 (hexane/ethyl acetate 6:1, v/v)

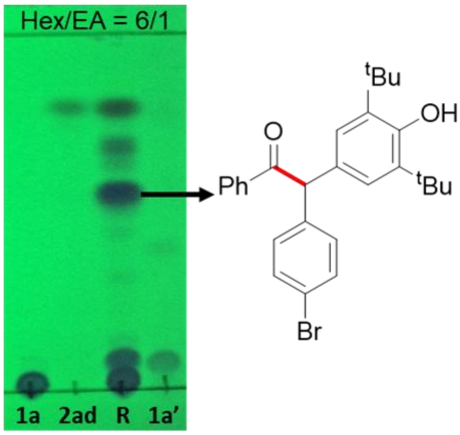

All other data matches what was reported in the literature.[29]

Diethyl 2-(1-(benzo[*d*][1,3]dioxol-5-yl)-2-oxo-2-phenylethyl)malonate (4ad)

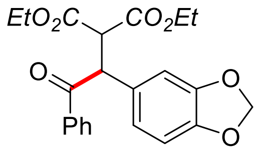

According to **General procedure E**, DHQZ **1a** (49.2 mg, 0.15 mmol, 1.5 equiv) and **2ae** (29.2 mg, 0.1 mmol, 1.0 equiv) were used to obtain crude residue, which was purified by silica gel column chromatography (hexane/ethyl acetate 18:1, v/v) to afford the corresponding product **4ad** as yellow oil (17.5 mg, 0.044 mmol, 44%).

^{1}H NMR (300 MHz, $CDCl_3$) δ 7.98 (d, J = 7.1 Hz, 2H), 7.52 – 7.47 (m, 1H), 7.42 – 7.37 (m, 2H), 6.79 – 6.77 (m, 2H), 6.71 – 6.68 (m, 1H), 5.89 (d, J = 4.0 Hz, 2H), 5.23 (d, J = 11.4 Hz, 1H), 4.37 (d, J = 11.4 Hz, 1H), 4.20 – 4.12 (m, 2H), 4.01 (q, J = 7.3 Hz, 2H), 1.21 (t, J = 7.1 Hz, 3H), 1.06 (t, J = 7.1 Hz, 3H) ppm.

^{13}C{^{1}H} NMR (101 MHz, $CDCl_3$) δ 197.3, 168.1, 168.0, 148.1, 147.4, 135.8, 133.1, 128.9, 128.5, 128.0, 122.7, 109.0, 108.7, 101.2, 61.9, 61.4, 56.0, 52.4, 13.9, 13.9 ppm.

See NMR spectrum

TLC: R_f = 0.28 (hexane/ethyl acetate 5:1, v/v)

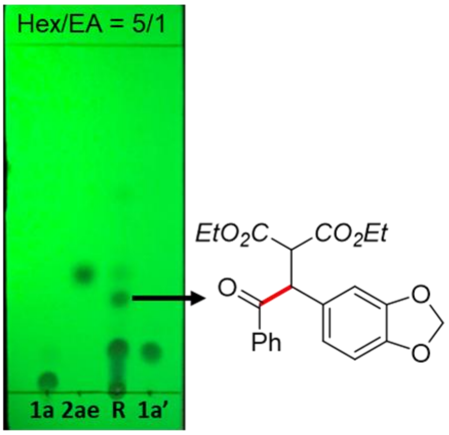

All other data matches what was reported in the literature.[30]

Bis((1*R*,2*S*,5*R*)-2-isopropyl-5-methylcyclohexyl) 2-(1-oxo-1-phenylpropan-2-yl)malonate (4ae)

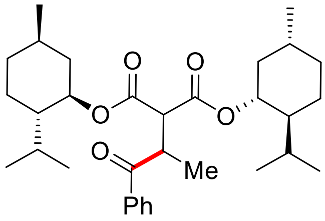

According to **General procedure E**, DHQZ **1a** (49.2 mg, 0.15 mmol, 1.5 equiv) and **2af** (29.2 mg, 0.1 mmol, 1.0 equiv) were used to obtain crude residue, which was purified by silica gel column chromatography (hexane/ethyl acetate 20:1, v/v) to afford the corresponding product **4ae** as colorless oil (51.1 mg, 0.089 mmol, 89%).

^{1}H NMR (300 MHz, $CDCl_3$) δ 8.03 – 7.98 (m, 2H), 7.57 (t, J = 7.2 Hz, 1H), 7.47 (t, J = 7.5 Hz, 2H), 4.78 (dtd, J = 14.9, 10.9, 4.4 Hz, 1H), 4.64 – 4.54 (m, 1H), 4.28 – 4.13 (m, 1H), 4.00 – 3.93 (m, 1H), 2.06 – 1.90 (m, 4H), 1.72 – 1.60 (m, 5H), 1.51 – 1.22 (m, 2H), 1.17 (d, J = 7.1 Hz, 3H), 1.09 – 0.69 (m, 24H), 0.63 (d, J = 6.9 Hz, 1H) ppm.

^{13}C{^{1}H} NMR (101 MHz, $CDCl_3$) δ 201.7, 201.6, 168.6, 168.5, 168.0, 167.9, 135.8, 135.6, 133.1, 133.0, 128.6, 128.6, 128.5, 75.9, 75.7, 75.6, 55.5, 55.5, 46.9, 46.8, 46.7, 46.6, 40.8, 40.6, 40.4, 40.4, 40.4, 40.2, 34.2, 34.1, 34.1, 31.4, 31.3, 31.3, 31.2, 26.2, 26.0, 25.2, 23.4, 23.2, 23.0, 22.7, 22.0, 22.0, 21.9, 20.8, 20.7, 20.6, 16.3, 16.3, 15.9, 15.8, 15.7, 15.4 ppm.

See NMR spectrum

HRMS (m/z): (ESI) calc'd for $C_{32}H_{48}O_5{}^{23}Na$ [M+Na]$^+$: 535.3394, found: 535.3392.

TLC: R_f = 0.63 (hexane/ethyl acetate 8:1, v/v)

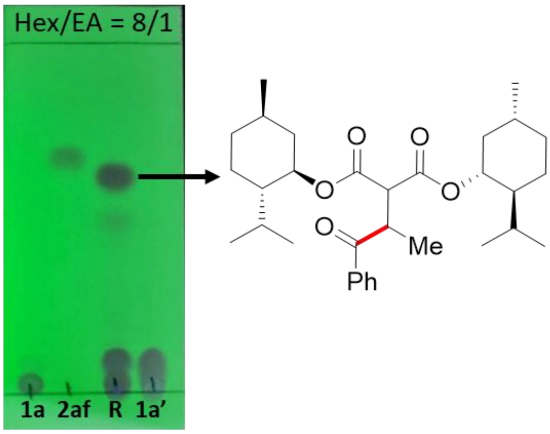

1,3-Diphenylprop-2-yn-1-one (5a)

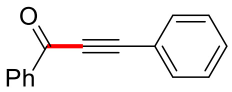

According to **General procedure E**, DHQZ **1a** (98.4 mg, 0.30 mmol, 3.0 equiv) and **2ag** (24.4 mg, 0.1 mmol, 1.0 equiv) was used to obtain a crude residue, which was purified by silica gel column chromatography (hexane/ethyl acetate 20:1, v/v) to afford the corresponding product **5a** as a white solid (7.6 mg, 0.037 mmol, 37%).

^{1}H NMR (300 MHz, $CDCl_3$) δ 8.23 (d, J = 7.4 Hz, 2H), 7.71 – 7.62 (m, 3H), 7.55 – 7.41 (m, 5H) ppm.

^{13}C{^{1}H} NMR (101 MHz, $CDCl_3$) δ 178.0, 136.9, 134.1, 133.0, 130.8, 129.6, 128.7, 128.6, 120.1, 93.1, 86.9 ppm.

See NMR spectrum

TLC: R_f = 0.44 (hexane/ethyl acetate 6:1, v/v)

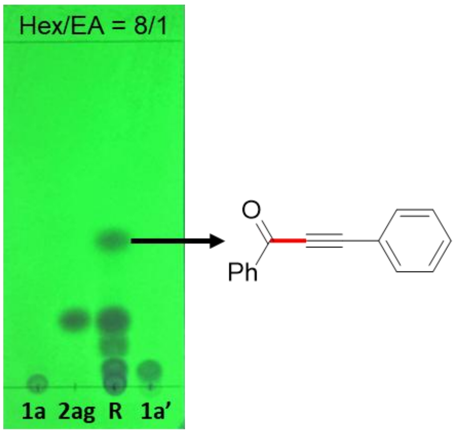

All other data matches what was reported in the literature.[32]

(*E*)-Chalcone (5b)

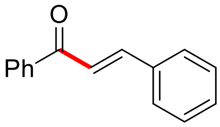

According to **General procedure E**, DHQZ **1a** (98.4 mg, 0.30 mmol, 3.0 equiv) and **2ah** (24.4 mg, 0.1 mmol, 1.0 equiv) was used to obtain a crude residue, which was purified by silica gel column chromatography (hexane/ethyl acetate 20:1, v/v) to afford the corresponding product **5b** as a pale-yellow solid (7.3 mg, 0.035 mmol, 35%).

^{1}H NMR (300 MHz, $CDCl_3$) δ 8.04 – 8.01 (m, 2H), 7.82 (d, J = 15.7 Hz, 1H), 7.65 (dd, J = 6.7, 2.8 Hz, 2H), 7.61 – 7.58 (m, 1H), 7.54 (d, J = 15.9 Hz, 1H), 7.53 – 7.49 (m, 2H), 7.43 – 7.40 (m, 3H) ppm.

^{13}C{^{1}H} NMR (101 MHz, $CDCl_3$) δ 190.7, 144.9, 134.9, 132.8, 130.5, 129.0, 128.6, 128.5, 128.4, 122.1 ppm.

See NMR spectrum

TLC: R_f= 0.49 (hexane/ethyl acetate 8:1, v/v)

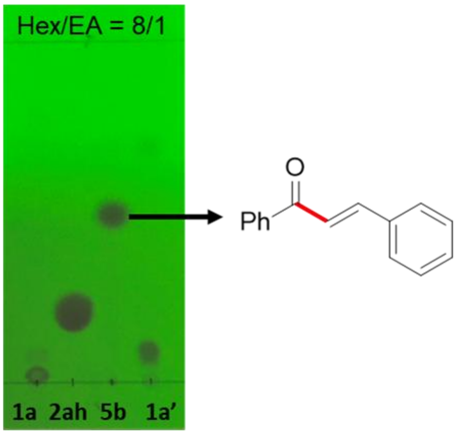

All other data matches what was reported in the literature.[33]

1,3-Diphenylbut-3-en-1-one (5c)/(*E*)-1,3-Diphenylbut-2-en-1-one (5c')

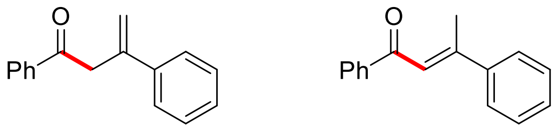

5c **5c'**

According to **General procedure E**, DHQZ **1a** (98.4 mg, 0.30 mmol, 3.0 equiv) and **2ai** (25.8 mg, 0.1 mmol, 1.0 equiv) was used to obtain a crude residue, which was purified by silica gel column chromatography (hexane/ethyl acetate 20:1, v/v) to afford the corresponding product **5c** (yellow oil, 7.1 mg, 0.032 mmol, 32%) and **5c'** (colorless oil, 5.9 mg, 0.027 mmol, 27%).

5c:

^{1}H NMR (300 MHz, $CDCl_3$) δ 8.00 – 7.97 (m, 2H), 7.59 – 7.54 (m, 1H), 7.50 – 7.40 (m, 4H), 7.34 – 7.24 (m, 3H), 5.61 (s, 1H), 5.18 (s, 1H), 4.17 (s, 2H) ppm.

See NMR spectrum

All other data matches what was reported in the literature.[34]

5c':

^{1}H NMR (300 MHz, $CDCl_3$) δ 8.02 – 7.98 (m, 2H), 7.59 – 7.41 (m, 8H), 7.17 (s, 1H), 2.60 (s, 3H) ppm.

See NMR spectrum

All other data matches what was reported in the literature.[35]

TLC:

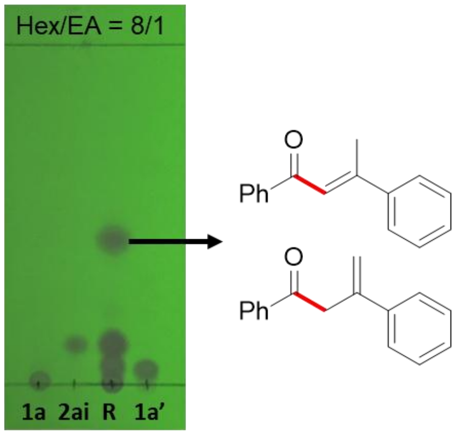

Back to contents/Back to table

S-Phenyl benzothioate (5d)

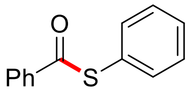

According to **General procedure E**, DHQZ **1a** (98.4 mg, 0.30 mmol, 3.0 equiv) and **2aj** (25.8 mg, 0.1 mmol, 1.0 equiv) was used to obtain a crude residue, which was purified by silica gel column chromatography (hexane/ethyl acetate 20:1, v/v) to afford the corresponding product **5d** as a white solid (18.2 mg, 0.085 mmol, 85%).

^{1}H NMR (300 MHz, $CDCl_3$) δ 8.04 (d, J = 7.5 Hz, 2H), 7.62 (t, J = 7.4 Hz, 1H), 7.54 – 7.46 (m, 7H) ppm.

^{13}C{^{1}H} NMR (101 MHz, $CDCl_3$) δ 190.1, 136.7, 135.1, 133.6, 129.5, 129.4, 129.2, 128.7, 127.5, 127.4 ppm.

See NMR spectrum

TLC: R_f = 0.44 (hexane/ethyl acetate 8:1, v/v)

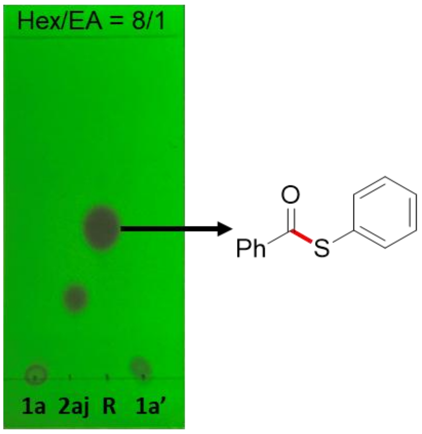

All other data matches what was reported in the literature.[36]

Se-Phenyl benzoselenoate (5e)

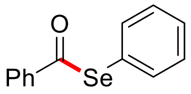

According to **General procedure E**, DHQZ **1a** (98.4 mg, 0.30 mmol, 3.0 equiv) and **2ak** (25.8 mg, 0.1 mmol, 1.0 equiv) was used to obtain a crude residue, which was purified by silica gel column chromatography (hexane/ethyl acetate 20:1, v/v) to afford the corresponding product **5e** as a white solid (18.2 mg, 0.047 mmol, 47%).

^{1}H NMR (400 MHz, $CDCl_3$) δ 7.94 (d, J = 7.5 Hz, 2H), 7.64 – 7.59 (m, 3H), 7.51 – 7.41 (m, 5H) ppm.

^{13}C{^{1}H} NMR (101 MHz, $CDCl_3$) δ 193.4, 138.6, 136.3, 133.9, 129.4, 129.1, 128.9, 127.3, 125.8 ppm.

See NMR spectrum

TLC: R_f = 0.65 (hexane/ethyl acetate 8:1, v/v)

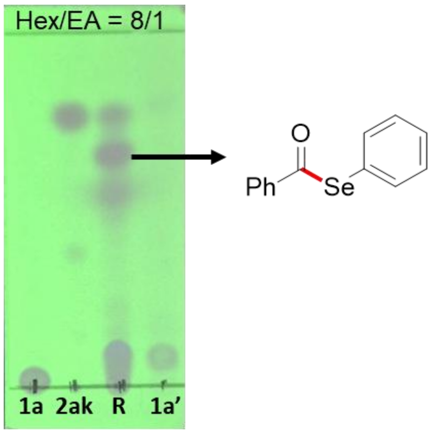

All other data matches what was reported in the literature.[37]

(4-Bromoquinolin-2-yl)(phenyl)methanone (6a)

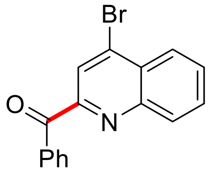

According to **General procedure E**, DHQZ **1a** (98.4 mg, 0.30 mmol, 3.0 equiv) and **2am** (20.8 mg, 0.1 mmol, 1.0 equiv) was used to obtain a crude residue, which was purified by silica gel column chromatography (hexane/ethyl acetate 20:1, v/v) to afford the corresponding product **6a** as a white solid (7.2 mg, 0.023 mmol, 23%).

^{1}H NMR (400 MHz, $CDCl_3$) δ 8.41 (s, 1H), 8.30 – 8.19 (m, 4H), 7.87 – 7.74 (m, 2H), 7.65 (t, J = 7.4 Hz, 1H), 7.52 (t, J = 7.5 Hz, 2H) ppm.

^{13}C{^{1}H} NMR (101 MHz, $CDCl_3$) δ 192.4, 156.3, 147.3, 135.2, 133.3, 131.4, 131.00, 130.96, 129.7, 128.2, 126.8, 124.8 ppm.

See NMR spectrum

TLC: R_f = 0.46 (hexane/ethyl acetate 8:1, v/v)

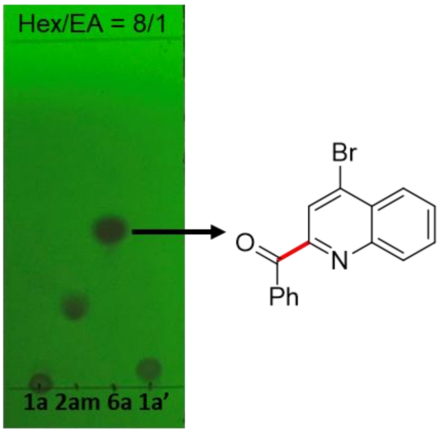

All other data matches what was reported in the literature.[38]

Di-*tert*-butyl 1-benzoylhydrazine-1,2-dicarboxylate (7a)

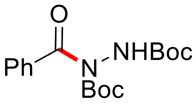

According to **General procedure E**, DHQZ **1a** (98.4 mg, 0.30 mmol, 3.0 equiv) and **2an** (23.2 mg, 0.1 mmol, 1.0 equiv) was used to obtain a crude residue, which was purified by silica gel column chromatography (hexane/ethyl acetate 25:1, v/v) to afford the corresponding product **7a** as a white solid (20.2 mg, 0.060 mmol, 60%).

^{1}H NMR (400 MHz, $CDCl_3$) δ 7.70 (br, 2H), 7.51 (t, J = 7.4 Hz, 1H), 7.41 (t, J = 7.5 Hz, 2H), 6.78 (br, 1H), 1.50 (s, 9H), 1.23 (s, 9H) ppm.

^{13}C{^{1}H} NMR (101 MHz, $CDCl_3$) δ 172.7, 154.5, 151.7, 135.8, 131.7, 128.2, 128.1, 84.4, 28.1, 27.4 ppm.

See NMR spectrum

TLC: R_f = 0.18 (hexane/ethyl acetate 8:1, v/v)

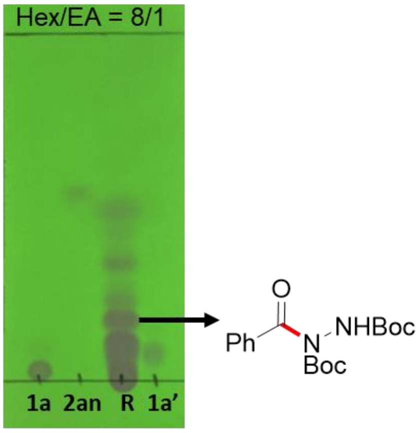

All other data matches what was reported in the literature.[39]

2.6. Scope limitation

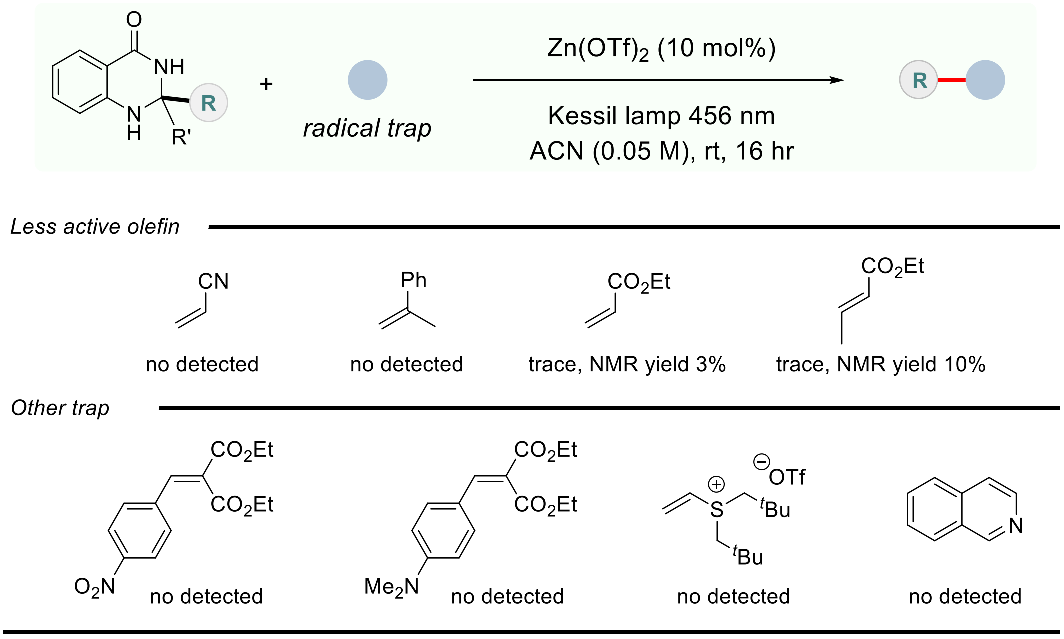

Scheme S1 Scope limitation

Discussion: Here, we presented representative failed olefin substrates. For the details, the conversion is fair; however, we don't receive the desired product in an acceptable amount to report. We proposed that Zn(OTf)2 might be involved in the addition process, so that the olefin, which cannot coordinate with Zn, shows less reactivity in the system. On the other hand, N-containing functional groups might be harmful in this methodology.

Back to contents

2.7. Flow synthesis

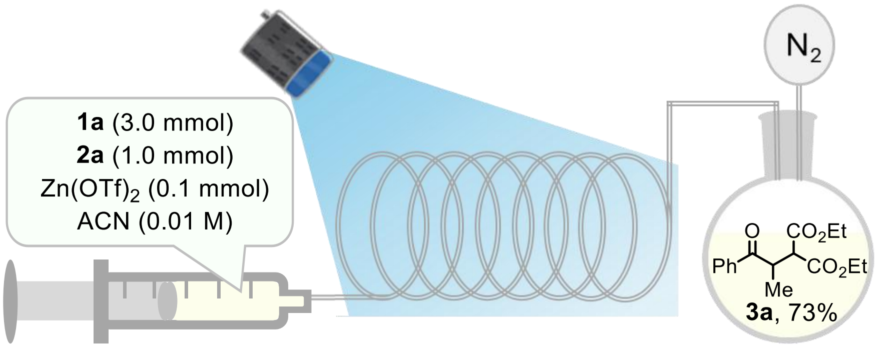

Figure S4 Flow reaction set-up diagram

Experimental detail: In a nitrogen-filled glove box, an oven-dried 250 mL flask equipped with a stir bar was added with DHQZ **1a** (3.0 mmol, 3.0 equiv), **2a** (1.0 mmol, 1.0 equiv), and $Zn(OTf)_2$ (0.1 mmol, 10 mol%) in anhydrous ACN (100 mL, 0.01 M). The resulting solution was removed from the glove box and injected into the flow system by a syringe pump (10 mL/h). After irradiated under blue light Kessil PR160L (456 nm) for 21 hr, the mixture was concentrated under reduced pressure and a high vacuum. The resulting residue was added with an NMR internal standard (trimethoxyl benzene, CAS# 621-23-8) and diluted with $CDCl_3$. The yield of **3a** was determined by ^{1}H NMR analysis.

Result:

$$Productivity = Yield \times Flow\ rate\ \times Concentration = 0.73\ \times 10\ \frac{mL}{h} \times 0.01\ \frac{mmol}{mL}$$
$$= 0.073\ \frac{mmol}{h}$$

$$Residence\ time\ (t_r) = \frac{Reaction\ scale\ in\ mmol}{Productivity} = \frac{1.0\ mmol}{0.073\ \frac{mmol}{h}} = 13.7\ h$$

3. Background and comparison of reaction types

3.1. EDA concept and comparison of different strategies

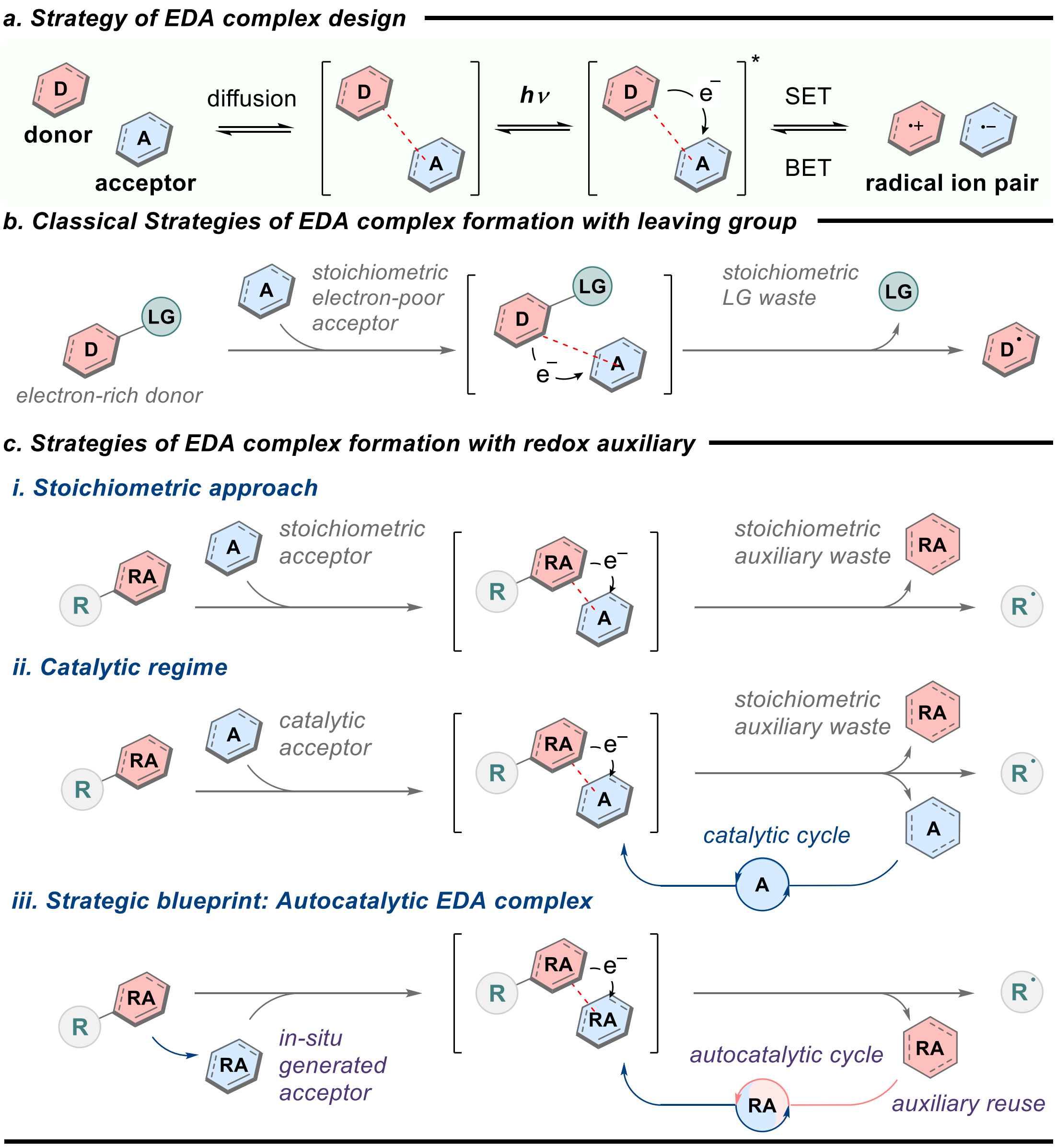

Scheme S2 Various EDA complex strategies

Electron donor-acceptor (EDA) complexes are reversible ground-state aggregates of electron-rich donor (D) and electron-poor acceptor (A) molecules (Scheme S1(a)). Upon irradiation, intracomplex single electron transfer (SET) ensues to form reactive radical ion pairs. To eliminate the competitive back electron transfer (BET)[40], the classical approach which introduced leaving groups (LG) into one of the EDA complex components to facilitate irreversible fragmentation following SET, was developed. (Scheme S1(b)). Furthermore, to overcome the limitation of EDA complex formation that specifically requires electron-rich donors and electron-deficient acceptors as substrates, an auxiliary strategy is introduced by installing a redox auxiliary (RA) on an inert substrate, enabling aggregation with another partner and thereby broadening the applicability of EDA complexes beyond the need for highly biased donors and acceptors.

3.2. Autoinductive autocatalysis reaction concept and comparison

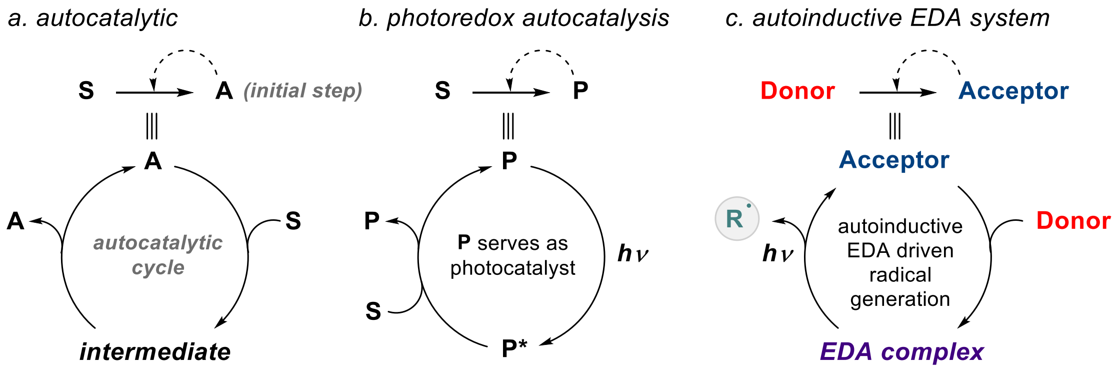

Figure S5 An autoinductive autocatalysis design in EDA complex [41] [42]

Generally, autocatalytic reactions are characterized by products that amplify their own production either through direct autocatalysis or an indirect autocatalytic network (Figure S5(a)). In photoredox autocatalysis, the product would generate slightly during the initial step through lower reaction rate. The obtained product could serve as photocatalyst under irradiation to facilitate the product formation faster. (Figure S5(b)). Herein, we present an autocatalytic EDA mode in which the acceptor (quinazolinone) is generated through the aromatization of the donor. This process leads to the formation of an EDA complex, which further promotes both the formation of the acceptor and the generation of synthetically useful radicals (Figure S5(c)).

Back to contents

4. Mechanistic studies

4.1. Background reaction

Table S13 Background reaction conditions

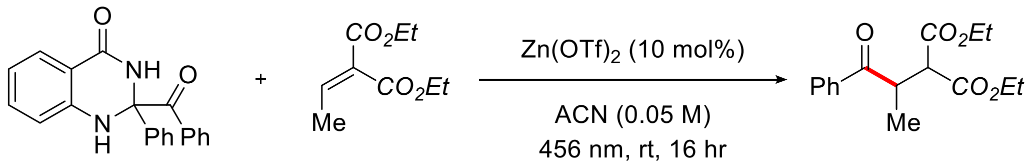

1a (2.0 equiv) **2a** (1.0 equiv) **3a**

entry[a]	variation	yield of **3a** (%)[b]
1	none	66
2	w/o $Zn(OTf)_2$	12
3	w/o irradiation	Not detected
4	w/o irradiation, 50 °C	Not detected

Reaction conditions: [a] **1a** (0.20 mmol), **2a** (0.10 mmol), $Zn(OTf)_2$ (0.01 mmol), ACN (2.0 mL), Kessil lamp (456 nm). [b] Yields were determined by ^{1}H NMR with 1,1,2,2-tetrachloroethane as an internal standard.

4.2. Radical trapping experiments

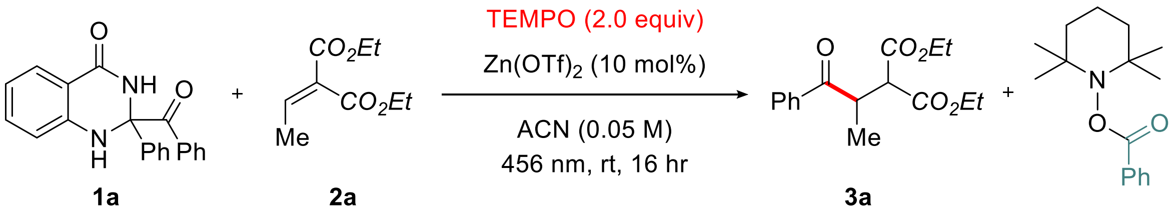

1a (1.5 equiv) **2a** (1.0 equiv) **3a** (not observed) 54% (isolated)

Reaction conditions: [a] **1a** (0.15 mmol), **2a** (0.10 mmol), $Zn(OTf)_2$ (0.01 mmol), ACN (2.0 mL), TEMPO (0.20 mmol), Kessil lamp (456 nm). [b] Isolated yield.

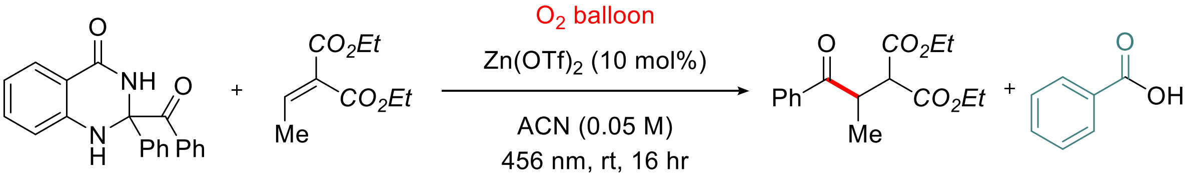

1a (1.5 equiv) **2a** (1.0 equiv) **3a** (trace) 77% (isolated)

Reaction conditions: [a] **1a** (0.15 mmol), **2a** (0.10 mmol), $Zn(OTf)_2$ (0.01 mmol), ACN (2.0 mL), Kessil lamp (456 nm) in O_2 gas. [b] Isolated yield.

4.3. Light on-off experiments

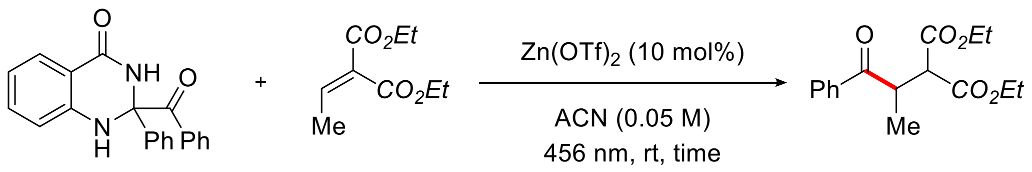

Reaction conditions: [a] **1a** (0.15 mmol), **2a** (0.10 mmol), $Zn(OTf)_2$ (0.01 mmol), anhydrous ACN (2.0 mL), Kessil lamp (456 nm) in N_2 gas. [b] Yields were determined by ^{1}H NMR with 1,1,2,2-tetrachloroethane as an internal standard.

Discussion: The following results show that the reaction would not proceed when the light was switched off, suggesting that the irradiation is highly crucial for facilitating this reaction.

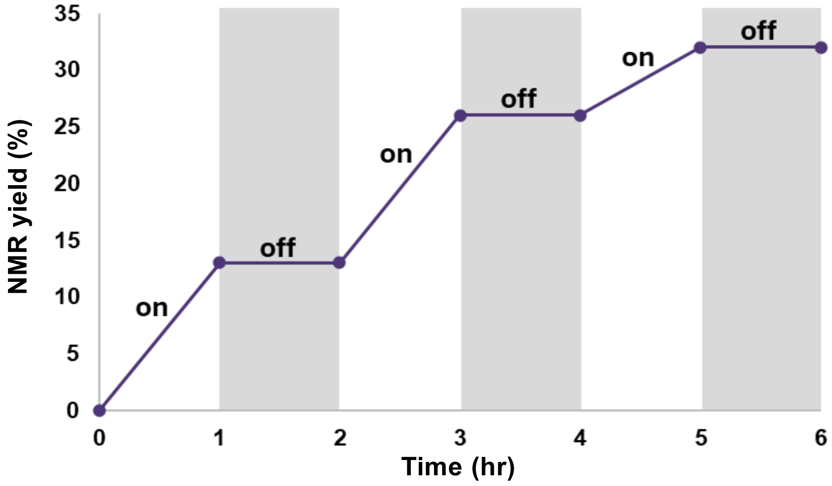

Figure S6 Light on-off experiments

4.4. UV-vis spectra

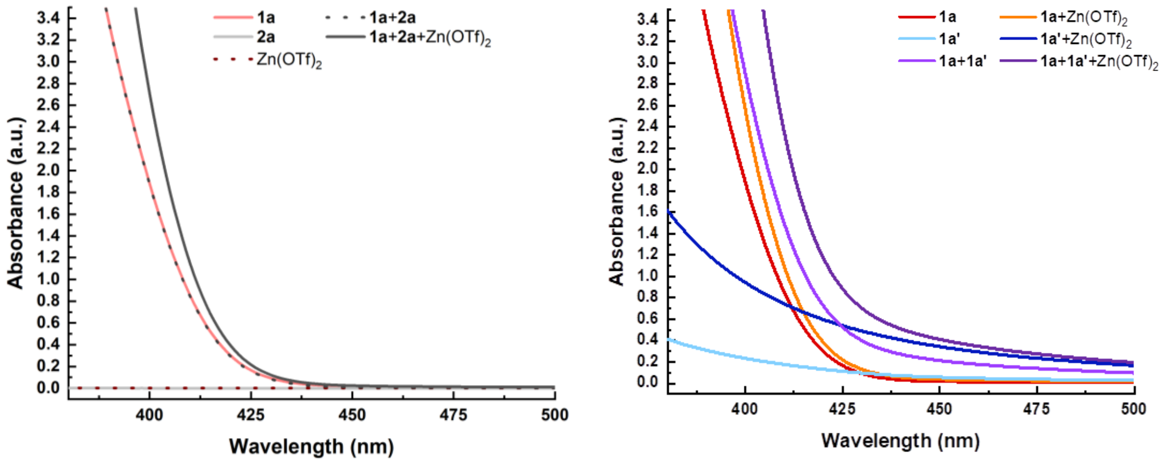

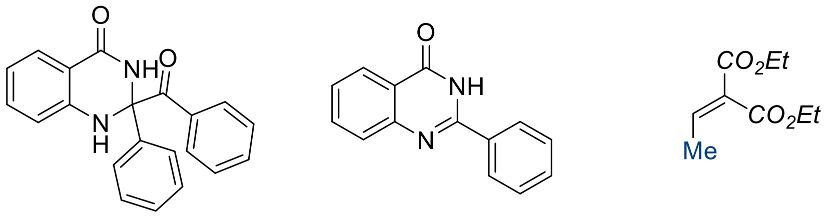

1a **1a'** **2a**

Figure S7 UV-vis spectra of 0.05 M in ACN

Sample preparation: In an N_2 gas-filled glove box, a 20 mL vial was added with 0.10 mmol compound and 2.0 mL degassed anhydrous ACN. Cover the vial with aluminum paper and stir it at room temperature for 30 minutes.

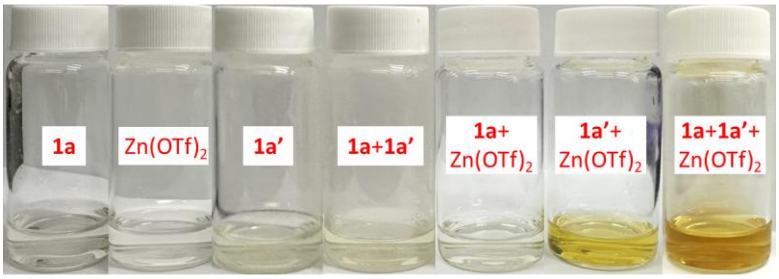

Figure S8 Solution of 0.05 M of chemical in ACN

Sample preparation: In an N_2 gas-filled glove box, a 20 mL vial was added with 0.10 mmol compound and 2.0 mL degassed anhydrous ACN.

Discussion: The results showed that the mixture of **1a** and **2a** had no significant change in absorption. Instead, mixture of **1a** and **1a'** does, and this phenomenon is enhanced by Lewis acid.

4.5. NMR investigation of EDA complex formation

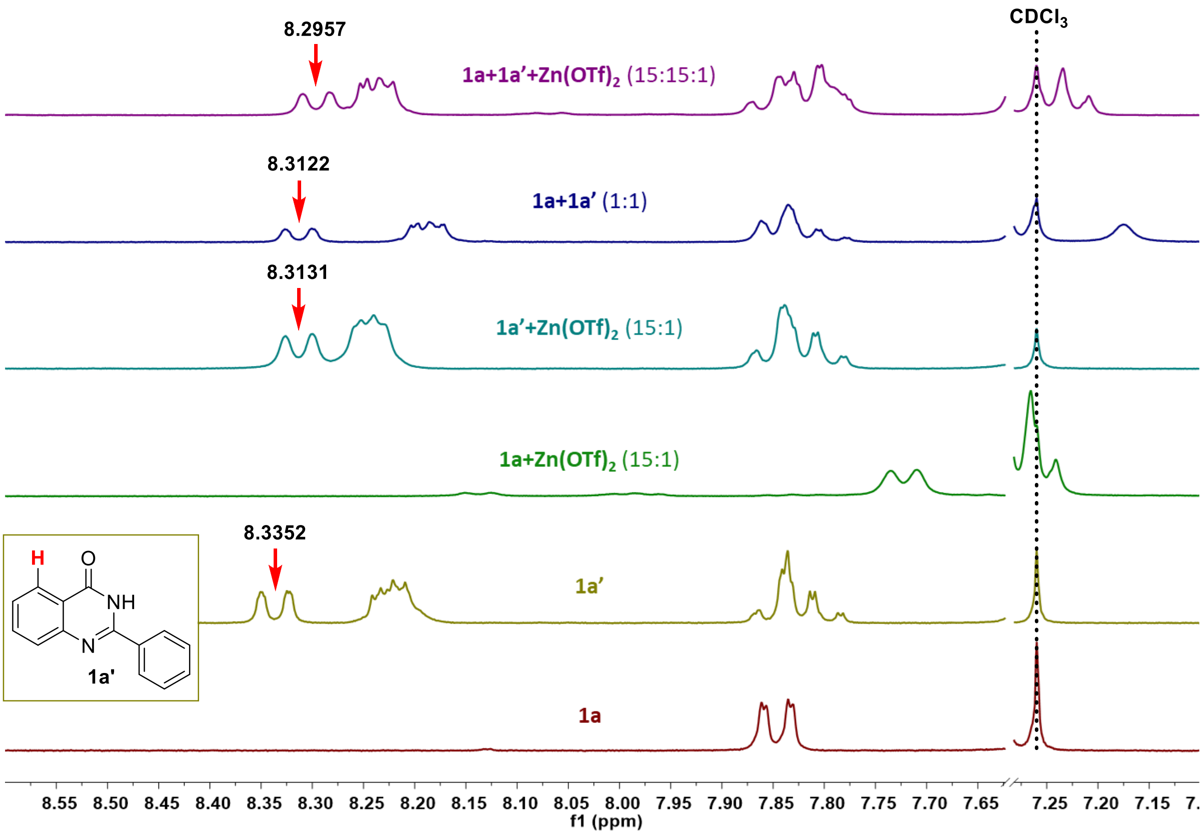

Figure S9 EDA complex formation investigation in ^{1}H NMR spectrum

Sample preparation: [a] (**1a** and **1a'**) In an N_2 gas-filled glove box, the chemical (0.025 mmol) was respectively added to an NMR tube and dissolved by $CDCl_3$. [b] (**1a**+**1a'**) In an N_2 gas-filled glove box, **1a** and **1a'** (1:1, mol/mol) were mixed in a 7 mL vial and dissolved by $CDCl_3$, covered the vial with aluminum paper and stirred it at room temperature for 15 minutes. [c] (**1a**+**1a'**+**Zn(OTf)$_2$**) In an N_2 gas-filled glove box, **1a**, **1a'**, and $Zn(OTf)_2$ (15:15:1, mol/mol/mol) were mixed in a 7 mL vial and dissolved by $CDCl_3$. The vial was covered with aluminum paper and stirred at room temperature for 15 minutes.

Discussion: The above result shows that the by-product **1a'** would interact with pro-aromatic DHQZ **1a**. The stronger upfield shift supports the hypothesis that Lewis acid could enhance the interaction.

4.6. Investigation of 1a' formation

Table S14 1a' formation experiment

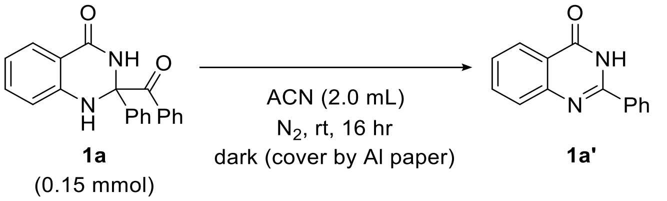

entry[a]	variation	yield of **1a'** (%)[b]
1	none	4
2[c]	Purge with N_2 for 30 min	<1
3	with $Zn(OTf)_2$ (0.01 mmol)	7
4	under 456 nm	74
5[c]	under 456 nm	72

Reaction conditions: [a] **1a** (0.15 mmol), anhydrous ACN (2.0 mL). The reaction was covered by aluminum paper stirring for 16 hr. [b]. Yields were determined by 1H NMR with trimethoxyl benzene as an internal standard. [c] Dry ACN was **purged by N_2 balloon for 30 min** before using.

Discussion: The result shows that the initiation might be assisted by a trace amount of oxygen in the solvent. Further, stirring with the Zn additive increased the initiation (entry3). While we irradiated it under a 456-nm Kessil lamp and indeed generated a significant amount of the byproduct **1a'** compared to just stirring in the dark (entry 4 and 5).

Back to contents

4.7. Investigation of autoinductive autocatalytic reaction

Table S15 Kinetic plot of compound 4n formation

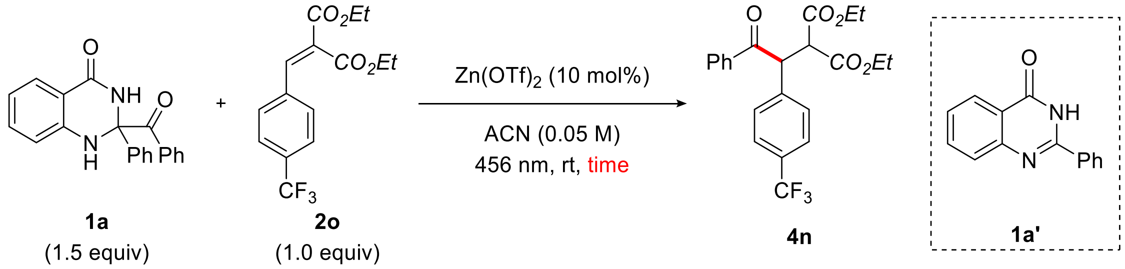

Yield of **4n**(%) \ Reaction time (hr)	1	2	3	5	8	10	12	14	15	16
Condition A (no variation)	1	5	8	14	20	22	31	52	65	67
Condition B (with 0.5 equiv **1a'**)	3	6	10	15	24	32	46	70	-	-
Condition C (with 1.0 equiv **1a'**)	2	6	12	18	30	48	74	-	-	-

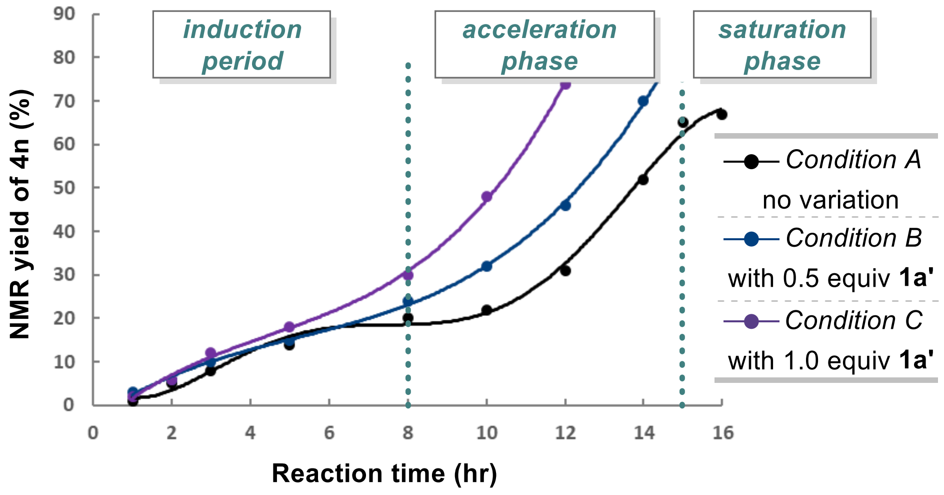

Figure S10 Kinetic studies for formation of compound 4n

Reaction set up: In a nitrogen-filled glove box, oven-dried tubes equipped with a stir bar were added with **1a** (0.15 mmol), the radical trap **2o** (0.10 mmol, 1.0 equiv), and $Zn(OTf)_2$ (0.01 mmol, 10 mol%) in anhydrous ACN (2 mL, 0.05 M) (***Condition A***). For ***Condition B*** and ***C*** additional **1a'** (0.05, 0.10 mmol) was added into the reaction tube. The resulting solution was irradiated with blue light Kessil PR160L (456 nm, 50 W) at a distance of 2.0 cm from the tube. The mixture was concentrated under reduced pressure and a high vacuum. The resulting residue was added with an NMR internal standard (tetrachloroethane, CAS# 79-34-5, 15 – 20 mg) and diluted with $CDCl_3$. The yield of **4n** was determined by ^{1}H NMR analysis.

Discussion: Comparing ***Condition B*** (0.5 equiv **1a'**) and ***C*** (1.0 equiv **1a'**) with ***Condition A***, we observed that the **1a'** additive indeed helps shorten the induction period. Also, the induction period cannot be eliminated, consistent with the reported characteristics of *autoinductive autocatalysis*.

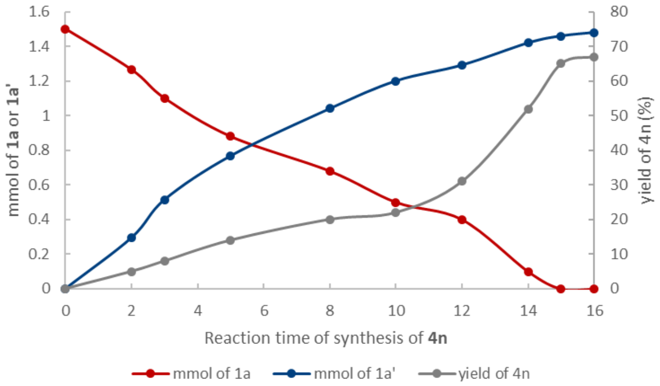

Figure S11 Kinetic studies for the formation of compound 1a' during the synthesis of compound 4n

Discussion: On the other hand, we further determined the amount of **1a'** based on the ^{1}H NMR of the crude reaction during data collection for ***Condition A*** (standard condition) in the kinetic investigation to produce the figure/curve. We proposed that the **1a'** could undergo further SET to keep the structure. Since the increase in **1a'** concentration is inversely proportional to the decrease in DHQZ concentration. Furthermore, the seemingly indirect relationship between **1a'** concentration and product formation supports its indirect role in assisting the reaction following *autoinductive autocatalysis*.

4.8. Quantum yield calculation

All the following calculations were done according to the previously reported paper [43, 44]

Equation S1 $$\Phi = \frac{\text{mol of product formed}}{\text{photon flux} * t * f}$$

Quantum yield formular is shown by Equation S1, where the photon flux is the number of photons incident on a system per unit time, t is the irradiation time, f is the fraction of the light absorbed of the sample (the UV-Vis spectrum revealed that).

Equation S2 $$photon\ flux = \frac{\text{mol of } Fe^{2+}}{\Phi * t * f} = 1.72 \times 10^{-9}\ (\text{einstein} \cdot s^{-1})$$

Photon flux measurement and calculation were done and documented in our previous work by using Fe^{2+} ion [44] (Equation S2), where Φ is the quantum yield for potassium ferrioxalate at λ_{max} = 456 nm (0.99), t is the irradiation time and f is the fraction of light absorbed; f value can be calculated by equation S3(>0.99).

Since the light source (Kessil PR160L), and all the other reaction set up in this work are identical to our previous work, the value was used to calculate the quantum yield of this work directly.

Equation S3 $$f = 1 - 10^{-A\,(456\ \text{nm})}$$

f is the fraction of the light absorbed. Based on the UV Vis spectrum (**Figure S7**), the value A of 3-component EDA complex is 0.37, which gives the value f is calculated to be 0.57.

Based on the result in condition A (standard condition) in the kinetic investigation (**Table S7**), we made the following calculation:

a) **Data of irradiation for 16 hr:**
mol of product: $6.7*10^{-5}$ mol; irradiation time: 16*60*60 sec

$$\Phi = \frac{\text{mol of product formed}}{\text{photon } flux * t * f} = \frac{6.7 * 10^{-5}}{1.72 * 10^{-9} * 16 * 60 * 60 * 0.57} = 1.186$$

b) **Data of irradiation for 8 hr:**
mol of product: $2.0*10^{-5}$ mol; irradiation time: 8*60*60 sec

$$\Phi = \frac{\text{mol of product formed}}{\text{photon } flux * t * f} = \frac{2.0 * 10^{-5}}{1.72 * 10^{-9} * 8 * 60 * 60 * 0.57} = 0.708$$

Discussion: Under otherwise identical conditions, the quantum yield above (0.71 at 8 hours and 1.19 at 16 hours) indicates that the reaction is a non-chain-propagation or inefficient chain propagation step in nature.

5. Computational details

5.1 Characterization of structures

The theoretical data reported in this study were all obtained with the program Gaussian16 (Revision C.01).[45] The DFT calculations have all been performed at B3LYP-D3/6-311++G(d,p) level of theory.[46] To account for the solvent effects, we have used a continuum solvation model of type SMD (solvation model based on density") in combination with the pre-defined parameters for acetonitrile. [47] The vibrational frequencies for all optimized geometries have been calculated analytically within the harmonic approximation. The presented minima geometries are checked to have no vibrational modes with imaginary frequencies. The vibrational frequencies are also used to approximate the Gibbs Free energies (ΔG) at a temperature of T = 298 K.

1f

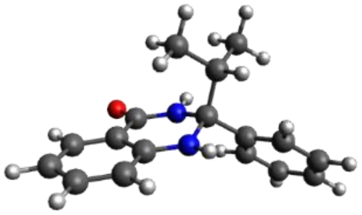

N 0.0435792376 0.9310556162 0.9564829620

H -0.5120778798 1.6349542585 1.4256534011

C 1.3479530416 0.8873774088 1.3530518333

C 2.2144482446 -0.0448255277 0.6050659931

C 1.6500395290 -0.8294094270 -0.4219491019

C 2.4787787373 -1.7268400386 -1.1251434649

H 2.0516873003 -2.3330651309 -1.9170627188

C 3.8252162153 -1.8243965822 -0.8066524099

H 4.4490884256 -2.5171411109 -1.3617720810

C 4.3889774984 -1.0417768028 0.2124316237

H 5.4427519498 -1.1255487723 0.4499525512

N 0.3093936336 -0.7422969947 -0.6982650747

C 3.5750015035 -0.1589885051 0.9095591732

H 3.9754049940 0.4605378432 1.7035751066

O 1.7463368531 1.5740337113 2.2992868871

C -0.5455242966 0.3677916924 -0.2609934530

C -1.9294549959 -0.2411202305 0.0545075618

C -2.4056604173 -0.3615102261 1.3622952838

H -1.8111296968 -0.0063009506 2.1945208015

C -3.6468825648 -0.9481927872 1.6189762395

H -3.9982113833 -1.0274172194 2.6421258194

C -4.4271876013 -1.4326228607 0.5716478703

H -5.3903366827 -1.8895001799 0.7708840048

C -3.9541599261 -1.3282868586 -0.7373336617

H -4.5474278181 -1.7060506928 -1.5630160725

C -2.7192927418 -0.7359600765 -0.9922467951

H -2.3751086411 -0.6674993944 -2.0177589290

C -0.6574464351 1.4730088980 -1.3696773112

C -1.6110828576 2.6038490455 -0.9606693782

H -2.6026960530 2.2316125624 -0.6934320675

H -1.7286501421 3.3056593428 -1.7908558384

H -1.2163885596 3.1691043916 -0.1101265322

C 0.7090056522 2.0420374576 -1.7670037208

H -1.0764139488 0.9723906269 -2.2481697129

H 1.1839866740 2.5629552257 -0.9306383282

H 0.5802532760 2.7667680444 -2.5759170827

H 1.3915613870 1.2678151490 -2.1228034994

H -0.0141696564 -1.2136614448 -1.5321768208

1a'

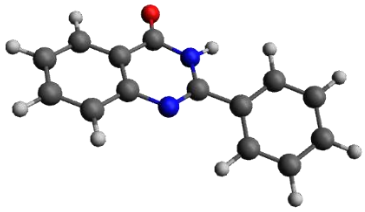

C -4.3846247778 -0.5134670006 0.0591384693

C -3.5858749680 0.6120981074 -0.0471289781

C -3.7917612988 -1.7832120113 0.1783185785

C -2.4148458859 -1.9233881115 0.1908734798

C -1.5848208247 -0.7895620763 0.0830062590

C -2.1885067660 0.4848755820 -0.0352825216

H -5.4642051643 -0.4186250340 0.0511672755

H -4.0207957617 1.5999726763 -0.1409501066

H -4.4207120598 -2.6627270003 0.2612775122

H -1.9517102102 -2.8990405444 0.2833280682

C -1.3272266097 1.6601766434 -0.1554941543

N 0.0292152841 1.3536994298 -0.1319496294

C 0.5447681765 0.0836039732 -0.0031319874

N -0.2126539755 -0.9665671111 0.1034222174

C 2.0210290695 -0.0628029384 0.0065827865

C 2.5881382822 -1.2791314766 -0.4033203459

C 3.9687297674 -1.4480210444 -0.3994846372

C 4.8029871933 -0.4094449059 0.0194606080

C 4.2469049647 0.8001873461 0.4340703498

C 2.8647046854 0.9760607460 0.4261160776

O -1.7036646116 2.8216482189 -0.2781743701

H 0.6553020132 2.1406650241 -0.2636910365

H 1.9412325332 -2.0826719752 -0.7317197795

H 4.3949100724 -2.3896597549 -0.7269716841

H 5.8789514526 -0.5436083523 0.0237282777

H 4.8868834030 1.6078899347 0.7706259144

H 2.4559253093 1.9168560433 0.7759484899

1a’+Zn(OTf)$_2$

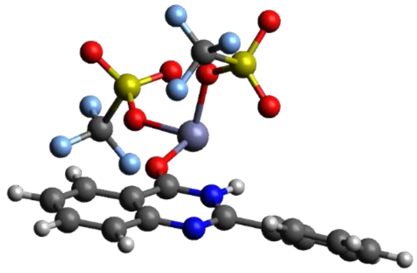

Zn 1.3011383215 -0.6052582115 -0.7658059726

S 4.4173412169 -0.2694345053 -0.1021996217

S -0.2631958743 -2.4453155391 1.2907612464

F -1.1255687699 -0.1418655572 2.3136616262

F -2.0155971025 -1.9108940789 3.2109551596

F 0.0277461903 -1.3518568877 3.7058273335

F 6.6675316988 0.3824602603 -1.3545781762

F 4.8903860269 0.5268865954 -2.6006942391

F 5.2913090741 2.0466878775 -1.0972084170

O 0.2314067320 1.0467784535 -0.3412388141

O 3.0222173084 0.2986721318 -0.2992705080

O 1.0034428460 -1.6978430855 0.9068621951

O -1.3131510939 -2.3327413856 0.2691343015

O 0.0028326810 -3.7602114626 1.8652063845

O 4.5435738523 -1.6593823326 -0.5463589625

O 4.9940164401 0.1004442827 1.1886027714

N -1.9355501648 0.4965516384 -0.8145539940

N -3.7711869842 1.8536546683 -0.2777447782

C -0.9941025984 1.3335218023 -0.3030697003

C -1.4923921458 2.5419115097 0.2996638994

C -2.8959647230 2.7415111148 0.3107763858

C -3.2921170101 0.7784652726 -0.8216320038

C -0.6268294854 3.4755063721 0.8974941340

C -1.1523722199 4.6007663459 1.5011266443

C -2.5461810966 4.8072089214 1.5164200243

C -3.4078169711 3.8981909377 0.9312610110

C -4.1788453249 -0.1913607969 -1.4973967916

C -3.7206483912 -0.9752918409 -2.5665997855

C -5.5142603539 -0.3030717561 -1.0831825963

C -6.3719834233 -1.1906013150 -1.7237266827

C -4.5860213099 -1.8579350187 -3.2079776171

C -5.9111464352 -1.9693795747 -2.7876397672

C 5.3770694164 0.7398320343 -1.3709052140

C -0.8810581545 -1.3919076118 2.7270153900

H -1.6204062774 -0.4105944206 -1.1479718310

H -2.9482306457 5.6934054130 1.9944866626

H -4.4800634589 4.0538560654 0.9379810006

H -5.8678235438 0.3003936617 -0.2565672879

H -2.7014870083 -0.8807074370 -2.9249776466

H -4.2262546920 -2.4507233704 -4.0411385083

H -6.5830373390 -2.6579863822 -3.2876346781

H -7.4004567376 -1.2770522388 -1.3921205739

H 0.4404576166 3.2933987408 0.8800489765

H -0.4949662823 5.3256178339 1.9662460995

Back to contents

Aggregation (1f+1a’)

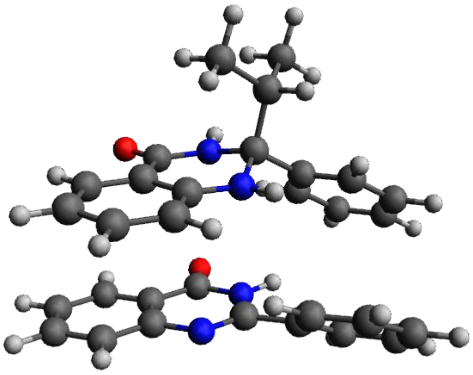

O 2.4057103717 2.4584233062 1.5452664106

O 0.6772113043 -0.8977061647 3.3218523242

N -0.3167689446 1.0446080385 -1.0878282589

N 0.2535680173 2.1395840950 0.9214923362

N -0.2610429502 -1.6343000420 1.3850392197

N 0.9447573106 -2.3263335999 -0.5045600486

C -0.0739932044 4.0419674591 -1.4439493909

C -1.7886072957 4.3143271946 0.3781033316

C -1.2196419622 3.3639104238 -0.6848837865

C -3.1719788081 1.0408668403 -0.2129203702

C -4.3076264724 0.4506323529 0.3353392810

C -4.3481485875 0.1297424582 1.6924544317

C -3.2381953026 0.3989522558 2.4892262882

C -2.0986920003 0.9894146757 1.9371331508

C -2.0513269624 1.3198790265 0.5813901180

C -0.8150168442 1.9751399597 -0.0685581792

C 3.3492559194 1.3909204000 -0.9555990826

C 3.7254392124 0.8347964351 -2.1706154169

C 2.7315651127 0.3531897332 -3.0344714808

C 1.3885766053 0.4288849884 -2.6954354330

C 1.0031839090 0.9958539845 -1.4660634947

C 2.0014322880 1.4768402150 -0.5952562966

C 1.5992029775 2.0680032095 0.6961323582

C 0.8336373677 -1.3283022576 2.1825748588

C 2.1142531705 -1.5679761563 1.5192335961

C 2.1113325303 -2.0610892870 0.1923819777

C -0.1851222951 -2.1054907685 0.0954640759

C 3.3248813398 -1.3017807797 2.1756815849

C 4.5280192068 -1.5298266972 1.5300748866

C 4.5323622012 -2.0284453240 0.2157038704

C 3.3456617742 -2.2905211566 -0.4457875942

C -1.4656419257 -2.3412468826 -0.6121943382

C -2.6108823927 -2.7768901884 0.0668495555

C -1.5307219539 -2.1142154349 -1.9941201862

C -2.7245749411 -2.3081774188 -2.6807764200

C -3.8013432693 -2.9822099503 -0.6272272573

C -3.8635241525 -2.7437475870 -1.9992816431

H -0.9911936690 0.8015425690 -1.7997976897

H 0.3062038809 3.4250487326 -2.2599880233

H -0.4329325707 4.9797377996 -1.8779450611

H 0.7590425217 4.2861572238 -0.7786076901

H -2.0136202658 3.1445028744 -1.4060291977

H -1.0229741418 4.5962187981 1.1080600424

H -2.1387013268 5.2338875843 -0.0987767215

H -2.6303925901 3.8748119257 0.9178535762

H -3.1682952109 1.2753581944 -1.2715012651

H -5.1578163815 0.2337081332 -0.3015175791

H -5.2309733525 -0.3328539378 2.1196141484

H -3.2488075493 0.1469573808 3.5441235848

H -1.2439249888 1.1640572200 2.5773570215

H 4.0905724545 1.7643574062 -0.2591697285

H 4.7713929855 0.7648699904 -2.4442391821

H 3.0111756079 -0.0860793202 -3.9863710592

H 0.6254767073 0.0579999107 -3.3708826457

H 0.0044525146 2.6457517998 1.7613453612

H -0.6470511563 -1.7710462114 -2.5161289748

H -2.5790816100 -2.9734746899 1.1325474319

H -4.6805015506 -3.3237093657 -0.0928107755

H -4.7939126244 -2.8945544552 -2.5355162285

H -2.7688090597 -2.1153788362 -3.7469393119

H 3.2967751401 -0.9146665006 3.1871773755

H 5.4650356020 -1.3231374087 2.0341239511

H -1.1685423620 -1.4069506857 1.7769647674

H 5.4764290519 -2.2016731606 -0.2890792566

H 3.3414151853 -2.6624998457 -1.4634374899

Back to contents

EDA complex II (1f+1a'+Zn(OTf)$_2$)

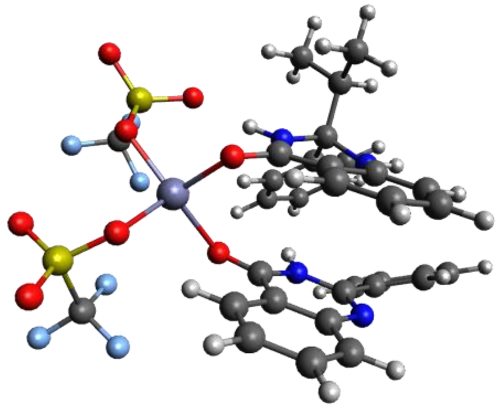

Zn 1.9266614218 0.0966555676 0.5036422045

S 4.6005162568 1.8320531943 -0.1627050076

S 2.8002745186 -2.8485205043 1.1627683772

F 4.5965811522 -3.0860461870 -0.7948546898

F 2.6396658015 -2.3912144617 -1.4447447336

F 2.9380933831 -4.4871089561 -0.9349963935

F 5.0713928902 2.1570939655 -2.7630876645

F 3.1274195842 2.8935972159 -2.1240714080

F 3.4681520313 0.7532444223 -2.3163817399

O 3.6456626684 -3.6875321587 2.0025527414

O 1.3453046261 -3.0808384535 1.1961084199

O 5.5434018782 0.7151881724 -0.1291340632

O 5.0987520133 3.1711389722 0.1461634461

O 3.2894434799 1.5524549814 0.5482093365

O 3.1445633276 -1.3768564044 1.2510035701

O 0.6315498889 0.7596404073 -0.9360696125

O 0.6437796134 0.2935776116 2.0185786192

N -2.8861720342 2.8350433451 -0.8610096833

N -1.5592262747 0.9847713891 -1.4089479857

N -0.9118298356 -1.2243903487 1.4009928859

N -3.1929395438 -0.6179830501 1.2222057517

C 4.0288279573 1.9181112675 -1.9583753241

C 3.2803871835 -3.2342056927 -0.6196089841

C -6.2059121105 -0.3515918138 -2.9361758672

C -4.9638227898 -0.5527633720 -3.5366926456

C -6.3182299200 0.5037634903 -1.8375407990

C -5.1950796064 1.1590212151 -1.3448400191

C -3.8327180484 0.0897084292 -3.0394564889

C -3.9427206547 0.9525574355 -1.9411493942

C -1.8871832198 4.7172470815 0.2368348905

C -0.7827236939 5.3399888052 0.7850720924

C 0.4752065910 4.7069108324 0.7897369906

C 0.6203800535 3.4494695676 0.2391982525

C -2.7676433511 1.6538068844 -1.3843051768

C -1.7650740179 3.4339526101 -0.3313774863

C -0.4924768734 2.8041206950 -0.3293302013

C -0.4021072934 1.4813604097 -0.8904504996

C -0.5821655320 -0.0382281039 1.9190067677

C -1.6457566174 0.8641963249 2.3134865796

C -2.9636338777 0.5241081881 1.9368448657

C -4.0058607087 1.4207970837 2.2436023421

C -3.7241361982 2.6011183006 2.9103672280

C -2.4119282727 2.9372592517 3.2872506436

C -1.3795261298 2.0710227899 2.9769258460

C -2.2656098009 -1.7555394565 1.1778648451

C -2.3467274016 -2.3666466372 -0.2363279435

C -1.2570038946 -2.3764204979 -1.1114331502

C -1.3739505600 -2.9220183131 -2.3917287055

C -2.5828202735 -3.4668880757 -2.8154701597

C -3.6765258284 -3.4585889207 -1.9492963048

C -3.5592512477 -2.9157036785 -0.6725947465

C -2.6164666569 -2.8214388499 2.2703955578

C -1.7242082286 -4.0663551147 2.1811471440

C -2.5815068345 -2.2365207181 3.6864044450

H -2.8608100999 5.1922325325 0.2383094767

H -0.8863505774 6.3258919827 1.2240987614

H -1.5278068595 0.0248178448 -1.7444006308

H 1.3303555393 5.2070726776 1.2285939948

H 1.5822578872 2.9573840673 0.2430671221

H -7.2810873243 0.6573219461 -1.3633029272

H -7.0832515674 -0.8596717698 -3.3207114710

H -4.8718048318 -1.2132266464 -4.3909067198

H -2.8762372719 -0.0713291289 -3.5236112157

H -5.2737829155 1.8177135027 -0.4893050069

H -0.1393850470 -1.8752198038 1.2578978230

H -5.0198962483 1.1752690668 1.9499377364

H -4.5375514928 3.2806870100 3.1410258997

Back to contents

H -2.2122166355 3.8700821398 3.7995243285

H -0.3538672599 2.3075199002 3.2303842607

H -0.3024982773 -1.9584609397 -0.8199071083

H -0.5140367864 -2.9151359454 -3.0524885836

H -2.6765070024 -3.8857815734 -3.8111060226

H -4.6275795223 -3.8689576224 -2.2695366609

H -4.4262087505 -2.9221017114 -0.0217258141

H -1.7412252765 -4.5155352179 1.1856663512

H -2.0760236045 -4.8164462176 2.8944131807

H -0.6861181452 -3.8339403260 2.4306647072

H -3.6468716320 -3.1214779825 2.0533566850

H -1.5738551564 -1.9060287178 3.9549579147

H -2.8781713564 -3.0067664293 4.4034538712

H -3.2653844487 -1.3939616978 3.8049280163

H -4.1590030687 -0.8541778785 1.0397777354

Back to contents

5.2 HOMO-LUMO calculation

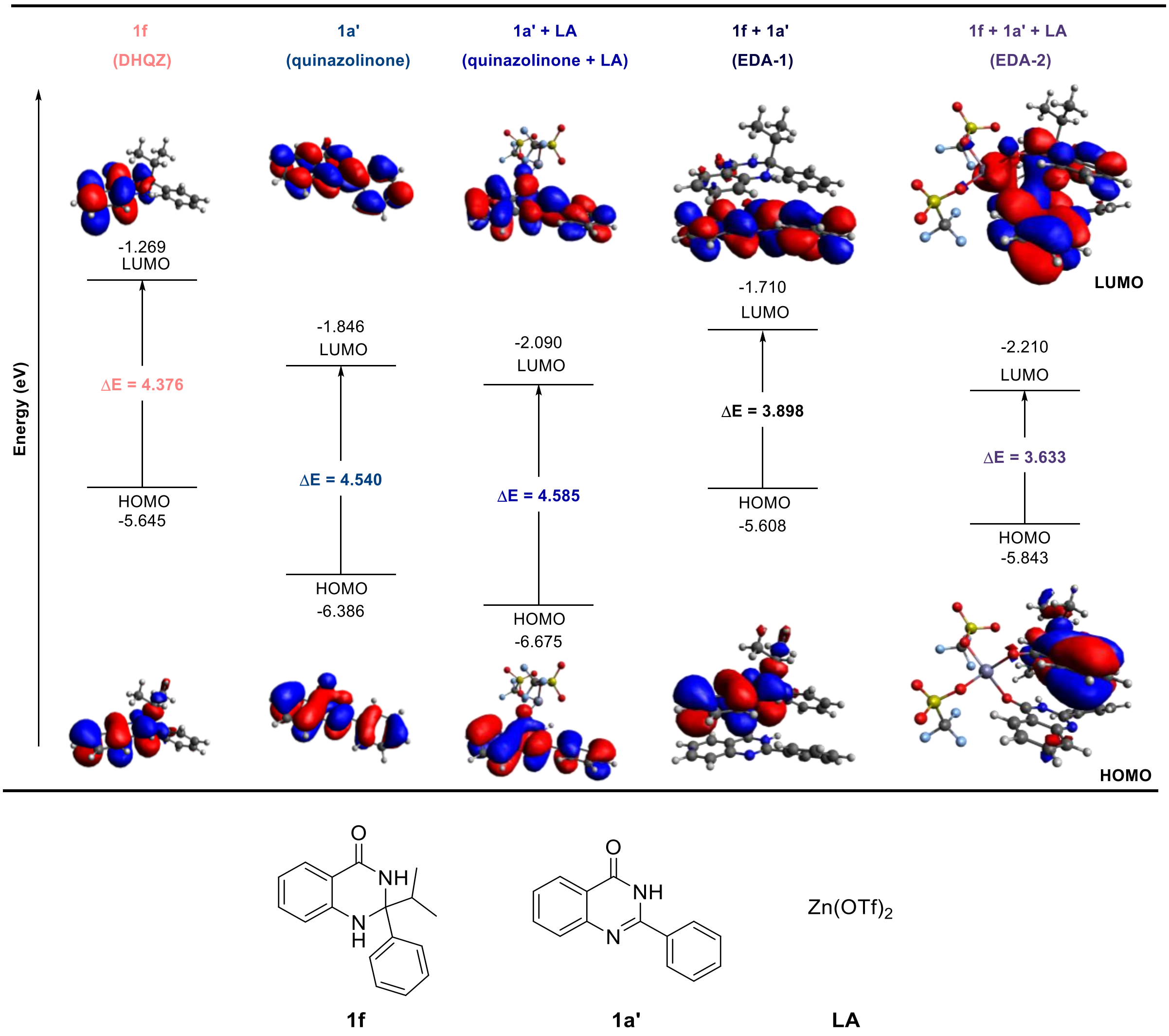

Figure S12 Computational result of HOMO-LUMO of molecules in ACN

Discussion: The results showed that the aggregation of **1f** and **1a’** had a significant decrease in HOMO-LUMO energy gap. This phenomenon is further enhanced by Lewis acid which explain the experimental observations in Table S15. The Lewis acid might facilitate the interaction between DHQZ and quinazolinone for efficient intracomplex charge transfer. Also, the HOMO/LUMO orbital locations imply the roles of **1f** and **1a’** as the donor and acceptor, respectively.

It should be noted here that the DFT calculations here are time-independent calculations, which only try to obtain a rough estimate for the energy required for the excitation of the complexes. The calculated HOMO-LUMO gaps, in fact, are a bit larger than what one would expect from the fact that the experiment is conducted under 456 nm irradiation (corresponding to 2.72 eV). In light of this, the DFT results for the HOMO-LUMO gaps should only be interpreted qualitatively instead of quantitatively.

Back to contents

6. Single crystal X-ray diffraction analysis

All single X-ray diffraction data were accumulated using Rigaku Oxford Diffraction single crystal X-ray diffractometers with Mo Kα radiation (λ = 0.71073 Å). The data collection was executed using the CrysAlisPro 1.171.41.56a program. Cell refinement and data reduction were made with CrysAlisPro 1.171.41.56a program. The structure was determined using the Olex2/ ShelXL program refined using full-matrix least squares. All non-hydrogen atoms were refined anisotropically, whereas hydrogen atoms were placed at calculated positions and included in the final stage of refinement with fixed parameters.

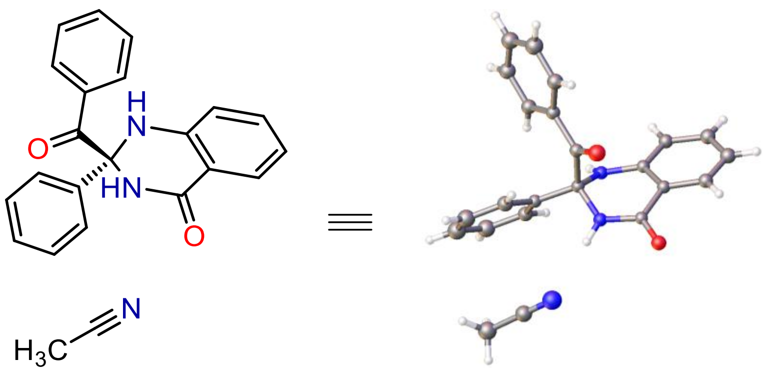

Figure S13 Single Crystallography data of compound 1a

(Co-crystal with ACN, CCDC number: 2374120)

Back to contents

K11307-HHL-A_auto

Table S16 Crystal data and structure refinement for K11307-HHL-A_auto.

Identification code	K11307-HHL-A_auto
Empirical formula	$C_{23}H_{19}N_3O_2$
Formula weight	369.41
Temperature/K	130(2)
Crystal system	monoclinic
Space group	$P2_1/n$
a/Å	8.8632(2)
b/Å	13.3019(3)
c/Å	16.2393(3)
α/°	90
β/°	91.061(2)
γ/°	90
Volume/Å^3	1914.24(7)
Z	4
ρ_{calc}g/cm^3	1.282
μ/mm^{-1}	0.084
F(000)	776.0
Crystal size/mm^3	0.5 × 0.4 × 0.3
Radiation	Mo Kα (λ = 0.71073)
2Θ range for data collection/°	3.958 to 50
Index ranges	$-10 \le h \le 10$, $-15 \le k \le 15$, $-19 \le l \le 19$
Reflections collected	38841
Independent reflections	3363 [R_{int} = 0.0827, R_{sigma} = 0.0299]
Data/restraints/parameters	3363/0/255
Goodness-of-fit on F^2	1.063
Final R indexes [I>=2σ (I)]	R_1 = 0.0389, wR_2 = 0.1039
Final R indexes [all data]	R_1 = 0.0456, wR_2 = 0.1078
Largest diff. peak/hole / e Å^{-3}	0.51/-0.51

Back to contents

Table S17 Fractional Atomic Coordinates (×10^4) and Equivalent Isotropic Displacement Parameters (Å^2×10^3) for K11307-HHL-A_auto. U_{eq} is defined as 1/3 of the trace of the orthogonalised U_{IJ} tensor.

Atom	*x*	*y*	*z*	U(eq)
O1	3401.4(11)	3843.7(7)	2690.5(6)	26.2(3)
O2	5996.7(12)	5920.2(8)	3019.9(7)	34.4(3)
N2	2605.3(13)	6824.3(8)	2713.2(7)	19.7(3)
N1	3242.9(13)	5314.6(8)	3378.6(7)	21.5(3)
C8	3319.8(15)	4771.6(10)	2676.9(8)	20.0(3)
C14	2740.1(15)	6368.9(10)	1948.3(8)	19.4(3)
N3	2236.8(17)	4276.0(12)	5013.3(9)	42.8(4)
C9	3176.8(15)	5355.8(10)	1908.8(8)	20.0(3)
C1	3487.0(15)	6399.7(10)	3392.1(8)	19.4(3)
C2	3011.8(16)	6788.5(10)	4231.4(8)	20.5(3)
C13	2391.8(16)	6880.8(10)	1216.6(9)	23.4(3)
C16	5811.5(16)	7661.2(11)	3254.5(8)	23.0(3)
C10	3327.5(16)	4890.2(11)	1146.3(9)	24.6(3)
C7	1596.7(17)	7200.5(11)	4346.6(9)	25.6(3)
C15	5197.7(16)	6614.3(11)	3224.3(8)	22.0(3)
C17	4981.3(17)	8507.7(11)	3478.6(9)	25.6(3)
C12	2505.6(17)	6391.8(11)	470.0(9)	27.2(3)
C3	4000.0(17)	6689.5(12)	4901.0(9)	28.5(4)
C11	2997.8(18)	5398.5(12)	427.5(9)	28.5(4)
C18	5654.8(18)	9449.8(12)	3504.5(9)	31.3(4)
C21	7330.7(17)	7790.2(13)	3058.0(9)	30.6(4)
C23	1612.4(18)	4572.7(13)	5569.3(10)	33.2(4)
C19	7154.1(19)	9559.2(13)	3303.8(9)	35.4(4)
C20	7988.7(19)	8729.8(14)	3077.6(10)	37.2(4)
C6	1183(2)	7524.8(12)	5125.7(10)	36.0(4)
C4	3581(2)	7004.1(13)	5676.5(10)	36.4(4)
C5	2177(2)	7431.9(13)	5784.6(10)	38.8(4)
C22	814.0(19)	4961.3(14)	6278.0(10)	38.2(4)

Back to contents

Table S18 Anisotropic Displacement Parameters (Å^2×10^3) for K11307-HHL-A_auto. The Anisotropic displacement factor exponent takes the form: -2π^2[h^{2}a*^{2}U$_{11}$+2hka*b*U$_{12}$+…].

Atom	U11	U22	U33	U23	U13	U12
O1	33.7(6)	15.6(5)	29.2(6)	0.4(4)	-0.3(5)	0.8(4)
O2	24.4(6)	35.4(6)	43.4(7)	-13.2(5)	1.7(5)	6.8(5)
N2	21.7(6)	16.5(6)	20.9(6)	-2.1(5)	-1.3(5)	4.4(5)
N1	28.3(7)	17.1(6)	19.2(6)	1.6(5)	2.2(5)	1.1(5)
C8	17.8(7)	17.8(7)	24.4(7)	-0.8(6)	-0.4(6)	-0.2(5)
C14	16.8(7)	19.1(7)	22.2(7)	-0.3(5)	0.2(6)	-2.1(5)
N3	42.6(9)	53.9(10)	32.1(8)	6.0(7)	0.4(7)	1.2(7)
C9	19.1(7)	18.9(7)	21.8(7)	-0.6(5)	-1.4(6)	-1.7(5)
C1	21.1(7)	16.4(7)	20.6(7)	0.6(5)	-0.3(6)	0.5(5)
C2	23.7(7)	15.6(7)	22.3(7)	-0.5(5)	3.8(6)	-2.9(5)
C13	25.1(8)	19.2(7)	25.7(8)	1.9(6)	-1.6(6)	0.6(6)
C16	22.5(7)	31.0(8)	15.3(7)	1.5(6)	-0.7(6)	-4.2(6)
C10	27.4(8)	19.8(7)	26.6(8)	-3.1(6)	1.5(6)	0.8(6)
C7	27.7(8)	22.3(7)	27.1(8)	2.7(6)	5.5(6)	0.9(6)
C15	21.4(7)	29.1(8)	15.5(7)	-2.0(6)	-1.8(6)	2.6(6)
C17	24.4(8)	26.9(8)	25.6(8)	1.5(6)	-0.9(6)	-4.9(6)
C12	31.5(8)	29.4(8)	20.7(8)	4.3(6)	-2.0(6)	-0.1(6)
C3	25.5(8)	35.6(9)	24.4(8)	-0.7(6)	1.2(6)	-3.6(6)
C11	35.2(9)	29.5(8)	20.9(8)	-4.3(6)	0.3(6)	0.1(7)
C18	37.6(9)	27.4(8)	28.7(8)	3.3(7)	-3.2(7)	-7.5(7)
C21	24.8(8)	46.1(10)	20.9(8)	0.2(7)	1.3(6)	-3.0(7)
C23	30.9(9)	37.8(9)	30.8(9)	8.9(7)	-7.0(7)	-3.9(7)
C19	40.9(10)	39.5(9)	25.7(8)	8.3(7)	-5.6(7)	-20.4(8)
C20	27.7(9)	56.8(11)	27.2(9)	6.8(8)	0.7(7)	-17.4(8)
C6	39.4(10)	32.1(9)	37.2(9)	2.5(7)	17.0(8)	7.0(7)
C4	40.9(10)	45.5(10)	22.8(8)	-3.0(7)	0.6(7)	-10.5(8)
C5	53.6(11)	37.6(9)	25.7(9)	-6.3(7)	15.0(8)	-5.7(8)
C22	35.4(9)	45.5(10)	33.6(9)	0.2(8)	-2.1(7)	-1.1(8)

Table S19 Bond Lengths for K11307-HHL-A_auto.

Atom	Atom	Length/Å	Atom	Atom	Length/Å
O1	C8	1.2366(16)	C13	C12	1.381(2)
O2	C15	1.2136(17)	C16	C15	1.496(2)
N2	C14	1.3890(18)	C16	C17	1.397(2)
N2	C1	1.4537(18)	C16	C21	1.400(2)
N1	C8	1.3521(17)	C10	C11	1.376(2)
N1	C1	1.4596(17)	C7	C6	1.392(2)
C8	C9	1.4730(19)	C17	C18	1.388(2)
C14	C9	1.4039(19)	C12	C11	1.394(2)
C14	C13	1.399(2)	C3	C4	1.384(2)
N3	C23	1.138(2)	C18	C19	1.382(2)
C9	C10	1.3930(19)	C21	C20	1.379(2)
C1	C2	1.5243(19)	C23	C22	1.457(2)
C1	C15	1.5716(19)	C19	C20	1.382(3)
C2	C7	1.385(2)	C6	C5	1.379(3)
C2	C3	1.390(2)	C4	C5	1.383(3)

Back to contents

Table S20 Bond Angles for K11307-HHL-A_auto.

Atom	Atom	Atom	Angle/°	Atom	Atom	Atom	Angle/°
C14	N2	C1	117.06(11)	C17	C16	C15	124.47(13)
C8	N1	C1	122.08(11)	C17	C16	C21	118.29(14)
O1	C8	N1	121.47(12)	C21	C16	C15	117.22(13)
O1	C8	C9	123.08(12)	C11	C10	C9	120.88(13)
N1	C8	C9	115.28(12)	C2	C7	C6	119.99(15)
N2	C14	C9	119.20(12)	O2	C15	C1	118.62(13)
N2	C14	C13	121.73(12)	O2	C15	C16	120.24(13)
C13	C14	C9	119.01(12)	C16	C15	C1	120.99(12)
C14	C9	C8	119.15(12)	C18	C17	C16	120.47(14)
C10	C9	C8	120.66(12)	C13	C12	C11	121.20(14)
C10	C9	C14	119.90(13)	C4	C3	C2	120.50(15)
N2	C1	N1	107.18(11)	C10	C11	C12	119.06(13)
N2	C1	C2	113.13(11)	C19	C18	C17	120.18(16)
N2	C1	C15	107.74(11)	C20	C21	C16	120.92(16)
N1	C1	C2	107.79(10)	N3	C23	C22	179.51(19)
N1	C1	C15	108.68(11)	C20	C19	C18	120.03(15)
C2	C1	C15	112.14(11)	C21	C20	C19	120.09(15)
C7	C2	C1	121.41(13)	C5	C6	C7	120.15(15)
C7	C2	C3	119.41(13)	C5	C4	C3	119.80(16)
C3	C2	C1	119.14(12)	C6	C5	C4	120.13(15)
C12	C13	C14	119.85(13)				

Back to contents

Table S21 Torsion Angles for K11307-HHL-A_auto.

A	B	C	D	Angle/°	A	B	C	D	Angle/°
O1	C8	C9	C14	165.21(13)	C1	N1	C8	C9	-18.77(18)
O1	C8	C9	C10	-8.6(2)	C1	C2	C7	C6	178.32(13)
N2	C14	C9	C8	6.61(19)	C1	C2	C3	C4	-177.80(13)
N2	C14	C9	C10	-179.54(12)	C2	C1	C15	O2	127.45(14)
N2	C14	C13	C12	-178.39(12)	C2	C1	C15	C16	-56.97(16)
N2	C1	C2	C7	21.26(18)	C2	C7	C6	C5	-0.3(2)
N2	C1	C2	C3	-161.35(12)	C2	C3	C4	C5	-0.8(2)
N2	C1	C15	O2	-107.43(14)	C13	C14	C9	C8	-170.77(12)
N2	C1	C15	C16	68.16(15)	C13	C14	C9	C10	3.1(2)
N1	C8	C9	C14	-10.03(18)	C13	C12	C11	C10	2.1(2)
N1	C8	C9	C10	176.16(12)	C16	C17	C18	C19	0.3(2)
N1	C1	C2	C7	-97.06(15)	C16	C21	C20	C19	0.9(2)
N1	C1	C2	C3	80.33(15)	C7	C2	C3	C4	-0.4(2)
N1	C1	C15	O2	8.39(17)	C7	C6	C5	C4	-0.9(2)
N1	C1	C15	C16	-176.02(11)	C15	C1	C2	C7	143.36(13)
C8	N1	C1	N2	46.83(16)	C15	C1	C2	C3	-39.24(17)
C8	N1	C1	C2	168.90(12)	C15	C16	C17	C18	178.75(13)
C8	N1	C1	C15	-69.36(15)	C15	C16	C21	C20	-179.44(13)
C8	C9	C10	C11	171.24(13)	C17	C16	C15	O2	179.77(14)
C14	N2	C1	N1	-48.50(15)	C17	C16	C15	C1	4.3(2)
C14	N2	C1	C2	-167.17(11)	C17	C16	C21	C20	-0.6(2)
C14	N2	C1	C15	68.30(14)	C17	C18	C19	C20	-0.1(2)
C14	C9	C10	C11	-2.5(2)	C3	C2	C7	C6	0.9(2)
C14	C13	C12	C11	-1.5(2)	C3	C4	C5	C6	1.4(3)
C9	C14	C13	C12	-1.1(2)	C18	C19	C20	C21	-0.5(2)
C9	C10	C11	C12	-0.1(2)	C21	C16	C15	O2	-1.5(2)
C1	N2	C14	C9	24.94(18)	C21	C16	C15	C1	-176.97(12)
C1	N2	C14	C13	-157.75(13)	C21	C16	C17	C18	0.0(2)
C1	N1	C8	O1	165.90(13)					

Table S22 Hydrogen Atom Coordinates (Å×10^4) and Isotropic Displacement Parameters (Å^2×10^3) for K11307-HHL-A_auto.

Atom	*x*	*y*	*z*	U(eq)
H2	2012.45	7346.69	2783.63	24
H1	3039.31	5004.04	3841.96	26
H13	2077.75	7563.19	1233.18	28
H10	3662.45	4212.56	1122.41	30
H7	908.14	7261.97	3894.08	31
H17	3948.54	8438.15	3614.38	31
H12	2243.5	6738.88	-23.47	33
H3	4970.12	6403.76	4825.98	34
H11	3104.21	5076.91	-90.52	34
H18	5083.24	10020.74	3660.54	38
H21	7916.51	7222.74	2909.15	37
H19	7611.37	10204.97	3321.26	43
H20	9017.18	8806.75	2935.48	45
H6	213.84	7810.74	5203.49	43
H4	4256.77	6926.33	6133.07	44
H5	1896.01	7662.25	6313.86	47
H22A	133.29	5503.09	6100.12	57
H22B	1544.86	5222.7	6684.53	57
H22C	226.85	4419.52	6526.21	57

Experimental

Single crystals of $C_{23}H_{19}N_3O_2$ **[K11307-HHL-A_auto]** were **[]**. A suitable crystal was selected and **[]** on a **XtaLAB Pro II AFC12 (RINC): Kappa single** diffractometer. The crystal was kept at 130(2) K during data collection. Using Olex2 [1], the structure was solved with the SHELXT [2] structure solution program using Intrinsic Phasing and refined with the SHELXL [3] refinement package using Least Squares minimisation.

1. Dolomanov, O.V., Bourhis, L.J., Gildea, R.J, Howard, J.A.K. & Puschmann, H. (2009), J. Appl. Cryst. 42, 339-341.
2. Sheldrick, G.M. (2015). Acta Cryst. A71, 3-8.
3. Sheldrick, G.M. (2015). Acta Cryst. C71, 3-8.

Crystal structure determination of [K11307-HHL-A_auto]

Crystal Data for $C_{23}H_{19}N_3O_2$ (M =369.41 g/mol): monoclinic, space group P2$_1$/n (no. 14), a = 8.8632(2) Å, b = 13.3019(3) Å, c = 16.2393(3) Å, β = 91.061(2)°, V = 1914.24(7) Å^3, Z = 4, T = 130(2) K, μ(Mo Kα) = 0.084 mm^{-1}, $Dcalc$ = 1.282 g/cm^3, 38841 reflections measured (3.958° ≤ 2Θ ≤ 50°), 3363 unique (R_{int} = 0.0827, R_{sigma} = 0.0299) which were used in all calculations. The final R_1 was 0.0389 (I > 2σ(I)) and wR_2 was 0.1078 (all data).

[Back to contents]

Refinement model description

Number of restraints - 0, number of constraints - unknown.
Details:
1. Fixed Uiso
 At 1.2 times of:
  All C(H) groups, All N(H) groups
 At 1.5 times of:
  All C(H,H,H) groups
2.a Aromatic/amide H refined with riding coordinates:
 N2(H2), N1(H1), C13(H13), C10(H10), C7(H7), C17(H17), C12(H12), C3(H3), C11(H11), C18(H18), C21(H21), C19(H19), C20(H20), C6(H6), C4(H4), C5(H5)
2.b Idealised Me refined as rotating group:
 C22(H22A,H22B,H22C)

This report has been created with Olex2, compiled on 2024.02.16 svn.r378c4104 for OlexSys. Please let us know if there are any errors or if you would like to have additional features.

Datablock k11307-hhl-a_auto - ellipsoid plot

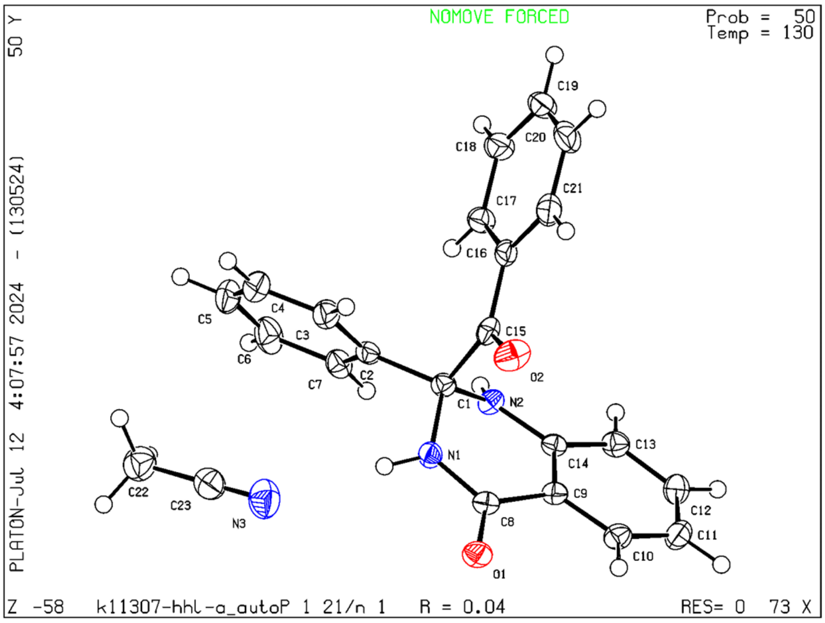

Back to contents

checkCIF/PLATON report

Structure factors have been supplied for datablock(s) k11307-hhl-a_auto

THIS REPORT IS FOR GUIDANCE ONLY. IF USED AS PART OF A REVIEW PROCEDURE FOR PUBLICATION, IT SHOULD NOT REPLACE THE EXPERTISE OF AN EXPERIENCED CRYSTALLOGRAPHIC REFEREE.

No syntax errors found. CIF dictionary Interpreting this report

Table S23 Datablock: k11307-hhl-a_auto

Bond precision:	C-C = 0.0020 A		Wavelength=0.71073
Cell:	a=8.8632(2)	b=13.3019(3)	c=16.2393(3)
	alpha=90	beta=91.061(2)	gamma=90
Temperature: 130 K			

	Calculated	Reported
Volume	1914.24(7)	1914.24(7)
Space group	P 21/n	P 1 21/n 1
Hall group	-P 2yn	-P 2yn
Moiety formula	C21 H16 N2 O2, C2 H3 N	C21 H16 N2 O2, C2 H3 N
Sum formula	C23 H19 N3 O2	C23 H19 N3 O2
Mr	369.41	369.41
Dx,g cm-3	1.282	1.282
Z	4	4
Mu (mm-1)	0.084	0.084
F000	776.0	776.0
F000'	776.31	
h,k,lmax	10,15,19	10,15,19
Nref	3366	3363
Tmin,Tmax	0.960,0.975	0.696,1.000
Tmin'	0.959	

Correction method= # Reported T Limits: Tmin=0.696 Tmax=1.000
AbsCorr = MULTI-SCAN

Data completeness= 0.999	Theta(max)= 25.000
R(reflections)= 0.0389(2921)	wR2(reflections)= 0.1078(3363)
S = 1.063	Npar= 255

The following ALERTS were generated. Each ALERT has the format
test-name_ALERT_alert-type_alert-level.
Click on the hyperlinks for more details of the test.

Alert level C

PLAT911_ALERT_3_C Missing FCF Refl Between Thmin & STh/L= 0.595 3 Report
0 8 1, 6 0 2, -3 2 7,
PLAT975_ALERT_2_C Check Calcd Resid. Dens. 0.90Ang From N2 . 0.49 eA-3
PLAT976_ALERT_2_C Check Calcd Resid. Dens. 1.04Ang From N2 . -0.53 eA-3
PLAT977_ALERT_2_C Check Negative Difference Density on H2 . -0.53 eA-3

Alert level G

Back to contents

PLAT007_ALERT_5_G Number of Unrefined Donor-H Atoms .... 2 Report
H1 H2
PLAT793_ALERT_4_G Model has Chirality at C1 (Centro SpGr) S Verify
PLAT909_ALERT_3_G Percentage of I>2sig(I) Data at Theta(Max) Still 75% Note
PLAT933_ALERT_2_G Number of HKL-OMIT Records in Embedded .res File 3 Note
-3 2 7, 0 8 1, 6 0 2,
PLAT967_ALERT_5_G Note: Two-Theta Cutoff Value in Embedded .res .. 50.0 Degree
PLAT969_ALERT_5_G The 'Henn et al.' R-Factor-gap value .... 3.286 Note
Predicted wR2: Based on SigI**2 3.28 or SHELX Weight 10.14
PLAT978_ALERT_2_G Number C-C Bonds with Positive Residual Density. 7 Info

---

0 **ALERT level A** = Most likely a serious problem - resolve or explain
0 **ALERT level B** = A potentially serious problem, consider carefully
4 **ALERT level C** = Check. Ensure it is not caused by an omission or oversight
7 **ALERT level G** = General information/check it is not something unexpected

0 ALERT type 1 CIF construction/syntax error, inconsistent or missing data
5 ALERT type 2 Indicator that the structure model may be wrong or deficient
2 ALERT type 3 Indicator that the structure quality may be low
1 ALERT type 4 Improvement, methodology, query or suggestion
3 ALERT type 5 Informative message, check

It is advisable to attempt to resolve as many as possible of the alerts in all categories. Often the minor alerts point to easily fixed oversights, errors and omissions in your CIF or refinement strategy, so attention to these fine details can be worthwhile. In order to resolve some of the more serious problems it may be necessary to carry out additional measurements or structure refinements. However, the purpose of your study may justify the reported deviations and the more serious of these should normally be commented upon in the discussion or experimental section of a paper or in the "special_details" fields of the CIF. checkCIF was carefully designed to identify outliers and unusual parameters, but every test has its limitations and alerts that are not important in a particular case may appear. Conversely, the absence of alerts does not guarantee there are no aspects of the results needing attention. It is up to the individual to critically assess their own results and, if necessary, seek expert advice.

Publication of your CIF in IUCr journals

A basic structural check has been run on your CIF. These basic checks will be run on all CIFs submitted for publication in IUCr journals (*Acta Crystallographica*, *Journal of Applied Crystallography*, *Journal of Synchrotron Radiation*); however, if you intend to submit to *Acta Crystallographica Section C* or *E* or *IUCrData*, you should make sure that full publication checks are run on the final version of your CIF prior to submission.

Publication of your CIF in other journals

Please refer to the *Notes for Authors* of the relevant journal for any special instructions relating to CIF submission.

Back to contents

7. References

[1] Z. N. Tsai, L. Y. Li, A. S. Paculba, S. Miñoza, Y. T. Tsao, P. S. Lin, H. H. Liao, *Chem.: Asian J.* **2024**, *19*, e202301004.

[2] T. Wang, Z. Zhang, F. Gao, X. Yan, *Org. Lett.* **2024**, *26*, 6915–6920

[3] X.-J. Tang, Z. Zhang, W. R. Dolbier, Jr., *Chem. Eur. J.* **2015**, *21*, 18961 – 18965.

[4] Z. Niu, W. Gunatilleke, Q. Sun, P. Lan, J. Perman, J.-G. Ma, Y. Cheng, B. Aguila, S. Ma, *Chem* **2018**, *4*, 2587 – 2599.

[5] Q. An, Z. Wang, Y. Chen, X.Wang, K. Zhang, H. Pan, W. Liu, Z. Zuo, *J. Am. Chem. Soc.* **2020**, *142*, 6216 – 6226.

[6] J. Wang, Y. Zhou, L. Zhang, Z. Li, X. Chen, H. Liu, *Org. Lett.* **2013**, *15*, 1508 – 1511.

[7] W. Nicholson, J. Howard, G. Magri, A.Seastram, A. Khan, R. R. A. Bolt, L. Morrill, E. Richards,D. L. Browne, *Angew. Chem. Int. Ed.* **2021**, *60*, 23128 – 23133.

[8] S. Ghosh, C. K. Jana, *Org. Biomol. Chem.* **2019**, *17*, 10153 – 10157.

[9] W. Nicholson, J. Howard, G. Magri, A.Seastram, A. Khan, R. R. A. Bolt, L. Morrill, E. Richards, D. L. Browne, *Angew. Chem. Int. Ed.* **2021**, *60*, 23128 – 23133.

[10] A. V. Narsaiah, K. Nagaiah, *Synth. Commun*. **2003**, *33*, 3825 – 3832.

[11] Raguin, O.; Fournie-Zaluski, M. C.; Romieu, A.; Pelegrin, A.;Chatelet, F.; Pelaprat, D.; Barbet, J.; Roques, B. P.; Gruaz-Guyon, A, *Angew. Chem. Int. Ed* **2005**, *44*, 4058 – 4061.

[12] P. Abeijón, J. M. Blanco, F. Fernµndez, M. D. García, C. López, *Eur. J. Org. Chem.* **2006**, *2006*, 759 – 764.

[13] Y. Liu, Y. Mao, Y. Hu, J. Gui, L. Wang, W. Wang, S. Zhang, *Adv. Synth. Catal.* **2019**, *361*, 1554–1558.

[14] Y. Yang, B. Han, F. Dong, J. Lv, H. Lu, Y. Sun, Z. Lei, Z, Yang, H, Ma, *Org. Lett.* **2022**, *24*, 4409 – 4414.

[15] D. Kühbeck, G. Saidulu, K. R. Reddy, D. D. Díaz, *Green Chem.* **2012**, *14*, 378 – 392.

[16] P. L. Kalar, K. Jain, S. Agrawal, S. Khan, R. Vishwakarma, A. Shivhare, M. M. Deshmukh, K. Das. *J. Org. Chem.* **2023**, *88*, 16829 – 16844.

[17] J. Zhu, M. Xu, B. Gong, A. Lin, S. Gao, *Org. Lett.* **2023**, *25*, 3271 – 3275.

[18] F. Wang, J. Li, A. L. Sinn, W. E. Knabe, M. Khanna, I. Jo,J. M. Silver, K. Oh, L. Li, G. E. Sandusky, G. W. Sledge, Jr., H.Nakshatri, D. R. Jones, K. E. Pollok, S. O. Meroueh. *J. Med. Chem.* **2011**, *54*, 7193 – 7205.

[19] S. Selvakumar, R. Sakamoto, and K. Maruoka, *Chem. Eur. J.* **2016**, *22*, 6552 – 6555.

[20] J. Wu, P. S. Grant, X. Li, A. Noble, V. K. Aggarwal, *Angew. Chem.* **2019**, *131*, 5753 – 5757.

[21] H. Ren, P. Zhang, J. Xu, W. Ma, D. Tu, C. Lu, H. Yan, *J. Am. Chem. Soc.* **2023**, *145*, 7638 – 7647.

[22] T. Fan, X. Ma, Y. Liu, C. Jiang, Y. Xu, Y. Chen, *J. Org. Chem.* **2022**, *87*, 5846 – 5855.

[23] Y. Zhang, T. Zhu, Y. Lin, X. Wei, X. Xie, R. Lin, Z. Zhang,W. Fang, J.-J. Zhang, Y. Zhang, M.-Y. Hu, L. Cai, Z. Chen, *Org. Biomol. Chem.* **2024**, *22*, 5561–5568.

[24] D. M. Kitcatt, E. Pogacar, L. Mi, S. Nicolle, A.-L. Lee, *J. Org. Chem.* **2024**, *89*, 16055–16059.

[25] N. P. Ramirez, J. C. Gonzalez-Gomez, *Eur. J. Org. Chem.* **2017**, 2154 – 2163.

[26] S. C. Chen, Q. Zhu, H. Chen, Z. Chen, T. Luo, *Chem. Eur. J.* **2023**, *29*, e202203425.

[27] K. Zhang, L. Chang, Q. An, X. Wang, Z. Zuo, *J. Am. Chem. Soc.* **2019**, *141*, 10556 – 10564.

[28] G. Feng, X. Wang, J. Jin, *Eur. J. Org. Chem.* **2019**, *2019*, 6728 – 6732.

[29] Ádám Márk Pálvölgyi, F. Ehrschwendtner, M. Schnürch, K. Bica-Schröder, *Org. Biomol. Chem.*, **2022**, *20*, 7245 – 7249.

[30] T. Selvia, K. Srinivasan, *Adv. Synth. Catal.* **2015**, *357*, 2111 – 2118.

[31] Q. Xia, X. Li, X. Fu, Y. Zhou, Y. Peng, J. Wang, G. Song, *J. Org. Chem.* **2021**, *86*, 9914 – 9923.

[32] L. Zheng, X. Guo, Y. Li, Y. Wu, X. Xue, P. Wang, *Angew. Chem. Int. Ed.* **2023**, *62*, e202216373.

[33] S. Tripathi, R. Kapoor, L. D. S. Yadav, *Adv. Synth. Catal.* **2018**, *360*, 1407 – 1413.

[34] X. Li, Q. Gu, X. Dong, X. Meng, X. Liu, *Angew. Chem. Int. Ed.* **2018**, *57*, 7668 – 7672.

[35] Q. Jiang, J. Jia, B. Xu, A. Zhao, C. C. Guo, *J. Org. Chem.* **2015**, *80*, 3586 – 3596.

[36] L. Köring, A Stepen, B. Birenheide, S. Barth, M. Leskov, R. Schoch, F. Krämer, F. Breher, J. Paradies, *Angew. Chem. Int. Ed.* **2023**, *62*, e202216959.

[37] J. H. Bai, X. J. Qi, W. Sun, T. Y. Yu, P. F. Xu, *Adv. Synth. Catal.* **2021**, *363*, 2084 – 2088.

[38] X. Y. Zhang, W. Z. Weng, H. Liang, H. Yang, B. Zhang, *Org. Lett.* **2018**, *20*, 4686 – 4690.

[39] M. Saleem, A. Ratwan, P. Yamini, D. Yadagiri, *Org. Lett.* **2024**, *26*, 2039 – 2044.

[40] R. Rathore, J. K. Kochi, *Adv. Phys. Org. Chem.* **2000**, *35*, 193.

[41] A. I. Hanopolskyi, V. A. Smaliak, A. I. Novichkov, S. N. Semenov, *ChemSystemsChem* **2021**, *3*, e2000026.

[42] J. Kaur, M. J. P. Mandigma, N. Bapat, J. P. Barham, *Angew. Chem., Int, Ed.* **2025**, e202423190.

[43] Pitzer, L.; Sandfort, F.; Strieth-Kalthoff, F.; Glorius, F. *J. Am. Chem. Soc.* **2017**, *139*, 13652–13655.

[44] S. C. Lee, L.Y. Lee, Z. N. Tsai, Y. H. Lee, Y. T. Tsao, P. G. Huang, C. K. Cheng, H. B. Lin, T. W. Chen, C. H. Yang, C. C. Chiu, H. H. Liao, *Org. Lett.* **2022**, *24*, 85–89.

[45] Gaussian Revision C.01 M. J. Frisch, G. W. Trucks, H. B. Schlegel, G. E.Scuseria, M. A. Robb, J. R. Cheeseman, G. Scalmani, V. Barone, G. A.Petersson, H. Nakatsuji, X. Li, M. Caricato, A. V. Marenich, J. Bloino, B. G.Janesko, R. Gomperts, B. Mennucci, H. P. Hratchian, J. V. Ortiz, A. F.Izmaylov, J. L. Sonnenberg, D. Williams-Young, F. Ding, F. Lipparini, F.Egidi, J. Goings, B. Peng, A. Petrone, T. Henderson, D. Ranasinghe, V. G.Zakrzewski, J. Gao, N. Rega, G. Zheng, W. Liang, M. Hada, M. Ehara, K.Toyota, R. Fukuda, J. Hasegawa, M. Ishida, T. Nakajima, Y. Honda, O.Kitao, H. Nakai, T. Vreven, K. Throssell, J. A. Montgomery, Jr., J. E. Peralta,F. Ogliaro, M. J. Bearpark, J. J. Heyd, E. N. Brothers, K. N. Kudin, V. N.Staroverov, T. A. Keith, R. Kobayashi, J. Normand, K. Raghavachari, A. P.Rendell, J. C. Burant, S. S. Iyengar, J. Tomasi, M. Cossi, J. M. Millam, M.Klene, C. Adamo, R. Cammi, J. W. Ochterski, R. L. Martin, K. Morokuma,O. Farkas, J. B. Foresman, D. J. Fox, Gaussian Inc., Wallingford CT, **2016**..

[46] S. Grimme, J. Antony, S. Ehrlich, H. Krieg, *J. Chem. Phys*. **2010**, *132*, 154104.

[47] V. Marenich, C. J. Cramer, D. G. Truhlar, *J. Phys. Chem. B* **2009**, *113*, 6378.

[Back to the contents](#)

8. NMR spectra

^{1}H NMR (300 MHz, $CDCl_3$) of **1a**, See procedure

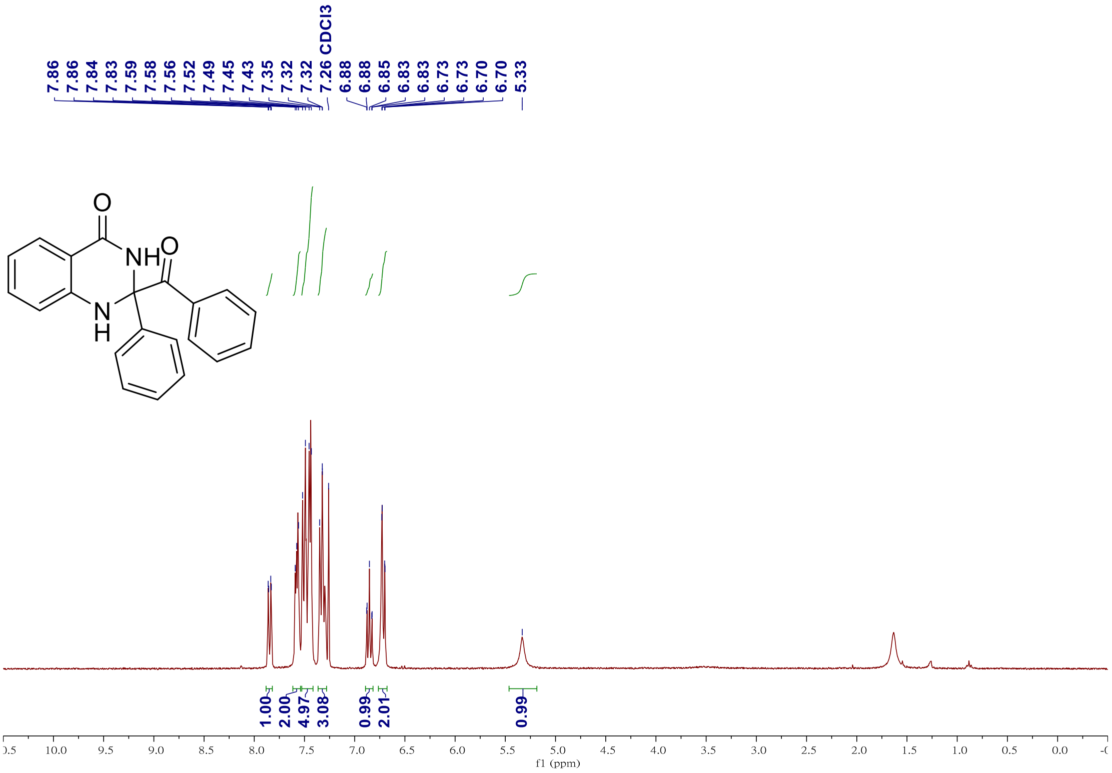

^{1}H NMR (300 MHz, $CDCl_3$) of **1a’**

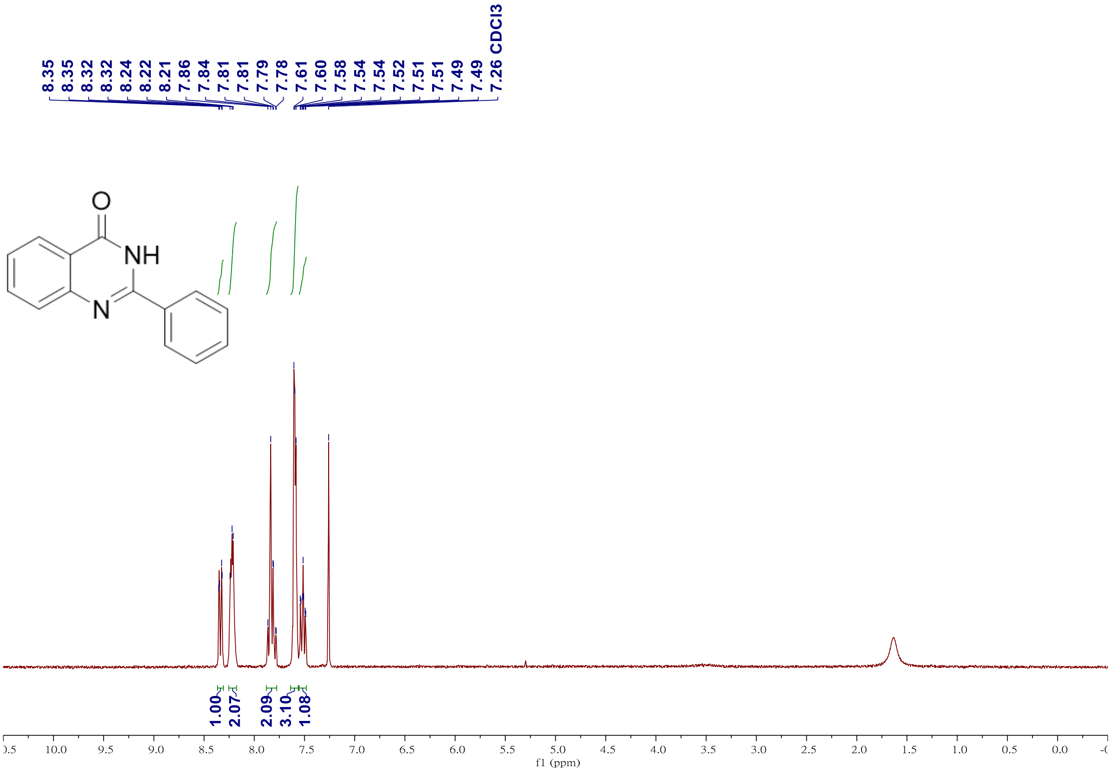

Back to the contents

^{1}H NMR (300 MHz, DMSO-d_6) of **1b,** *See procedure*

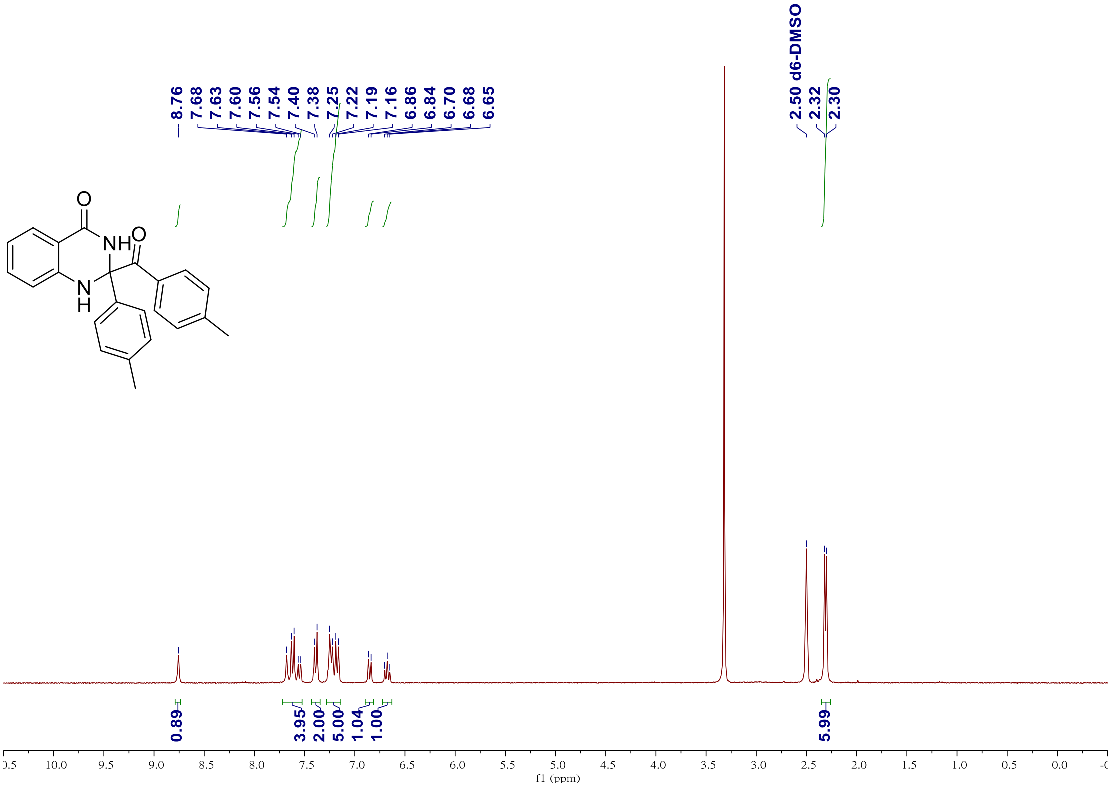

^{1}H NMR (400 MHz, $CDCl_3$) of **1c,** *See procedure*

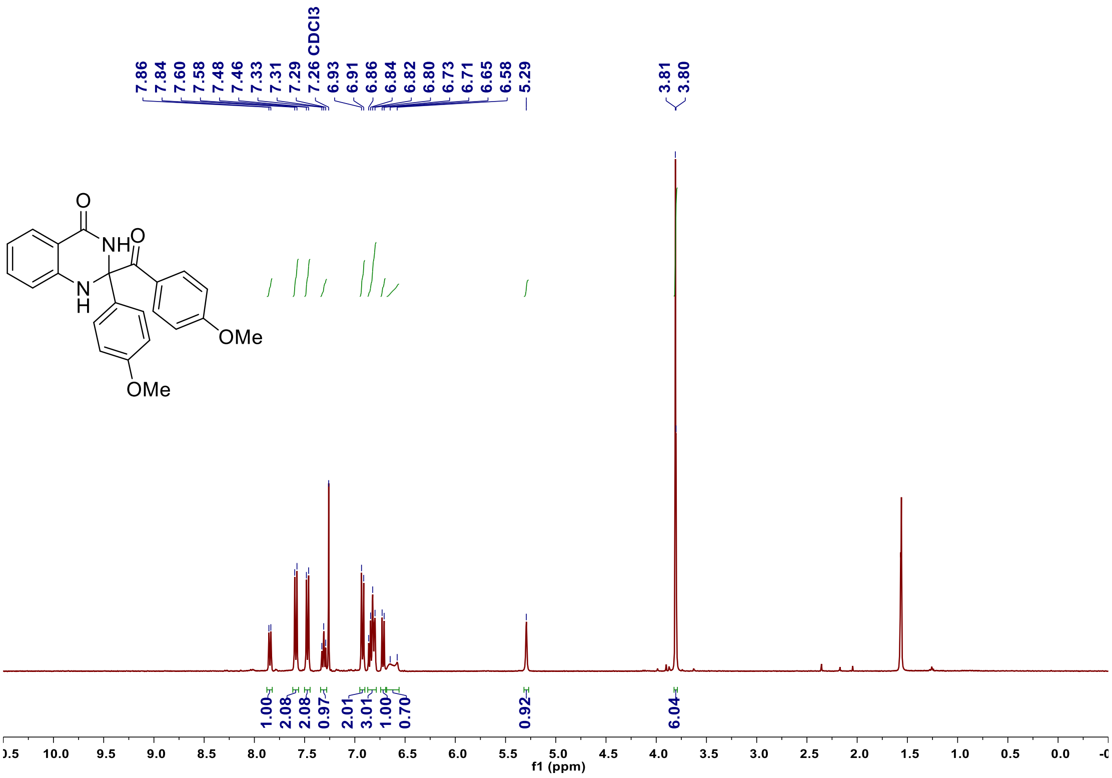

Back to the contents

^{13}C NMR (101 MHz, $CDCl_3$) of **1c**

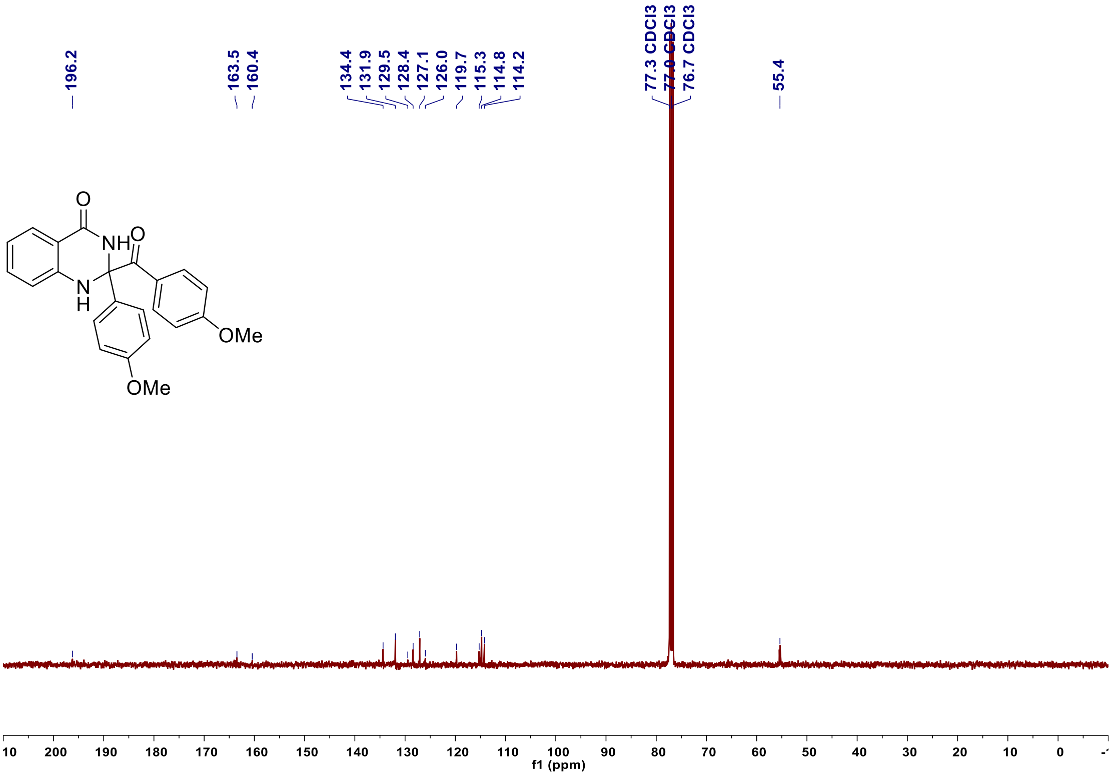

^{1}H NMR (300 MHz, $CDCl_3$) of **1d**, See procedure

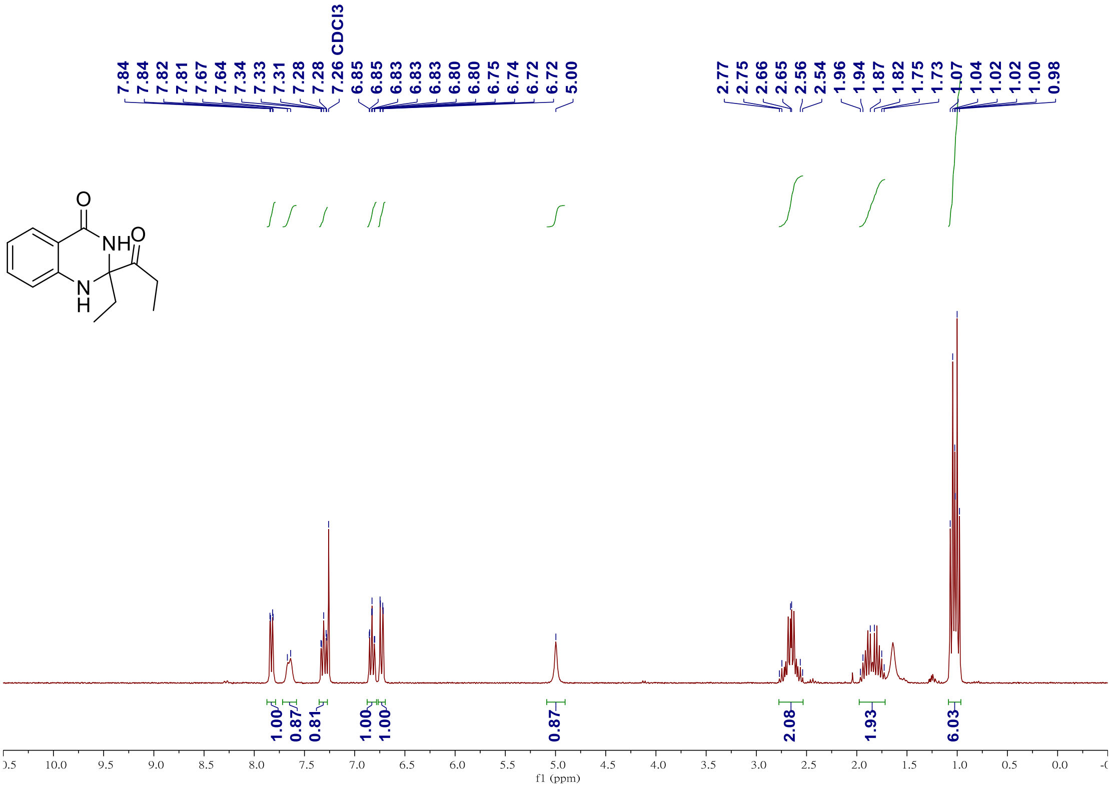

Back to the contents

^{1}H NMR (300 MHz, DMSO-d_6) of **1e**, See procedure

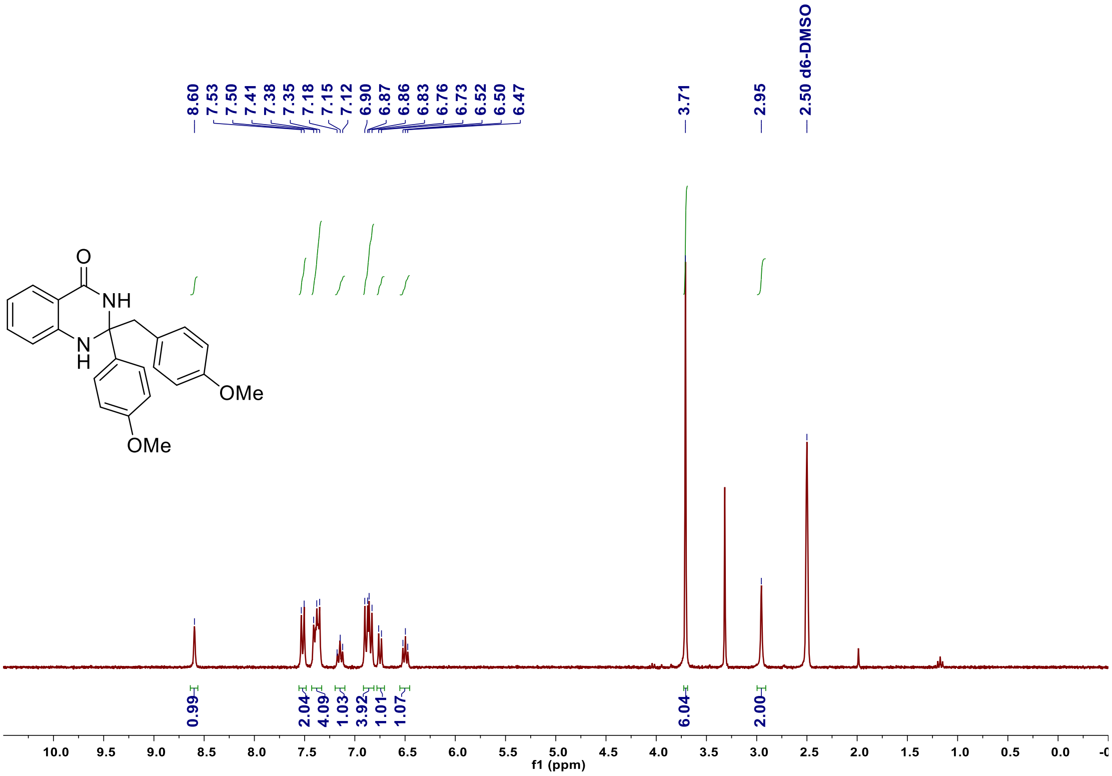

^{1}H NMR (300 MHz, DMSO-d_6) of **1f**, See procedure

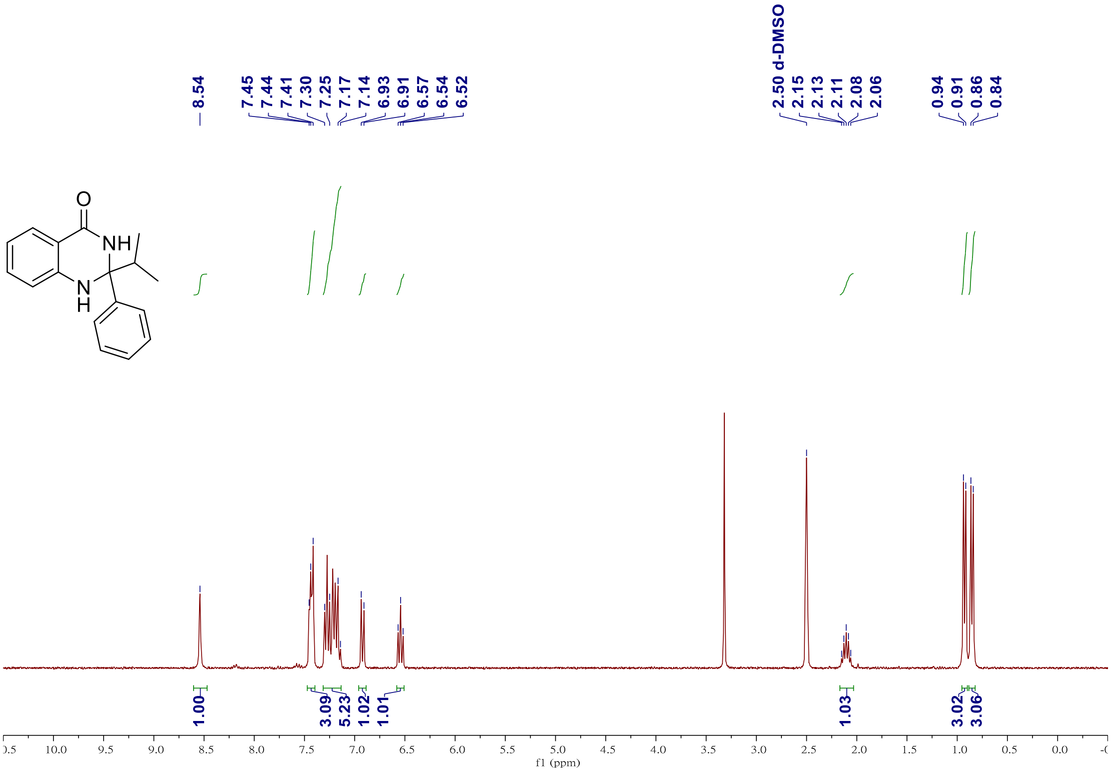

Back to the contents

^{1}H NMR (400 MHz, $CDCl_3$) of **1g**, See procedure

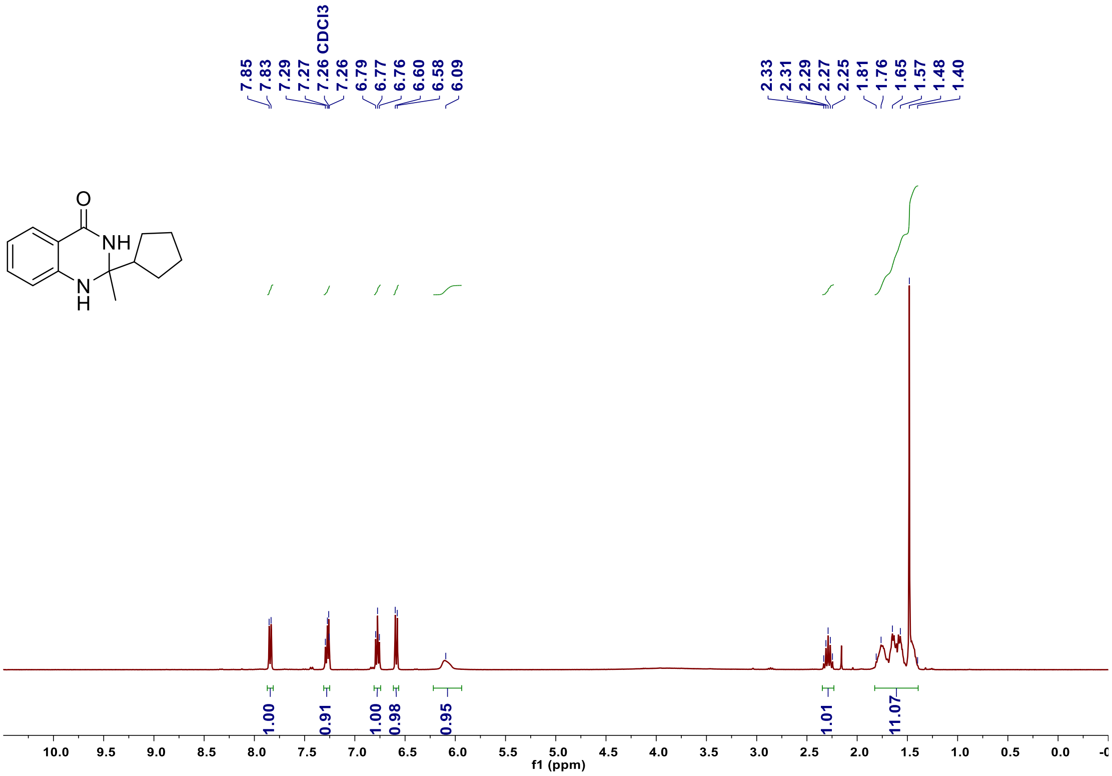

^{1}H NMR (300 MHz, $CDCl_3$) of **1h**, See procedure

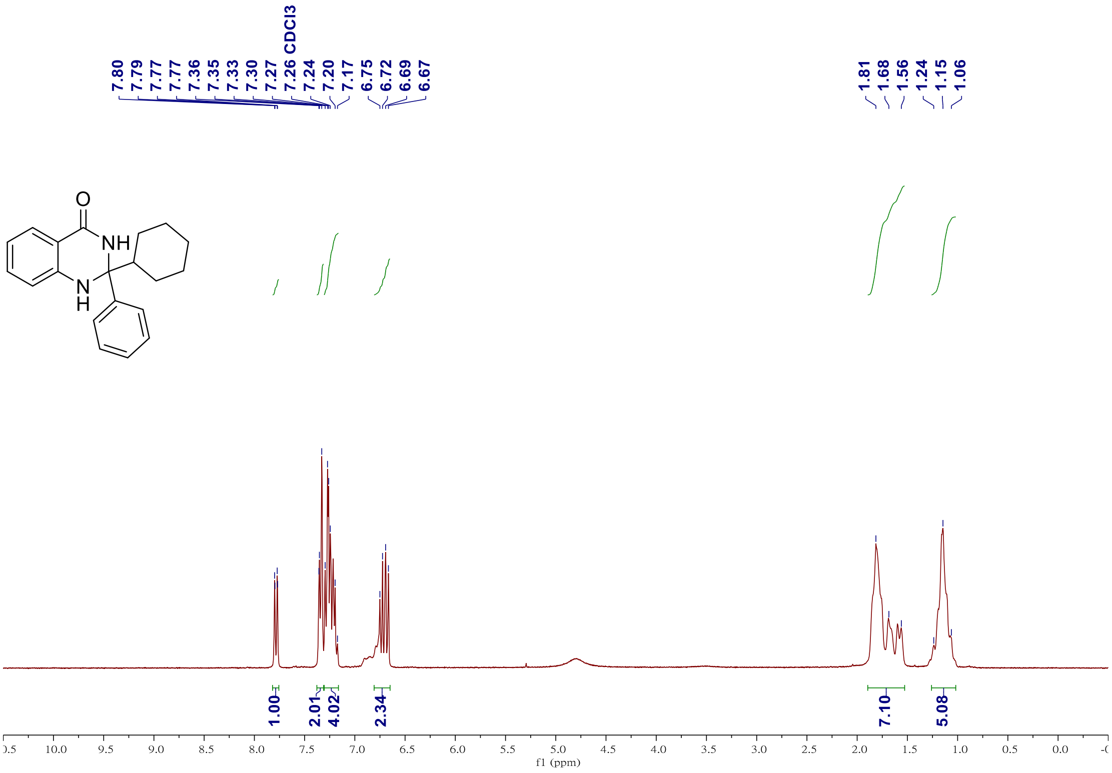

[Back to the contents](#)

^{1}H NMR (300 MHz, DMSO-d_6) of **1i**, See procedure

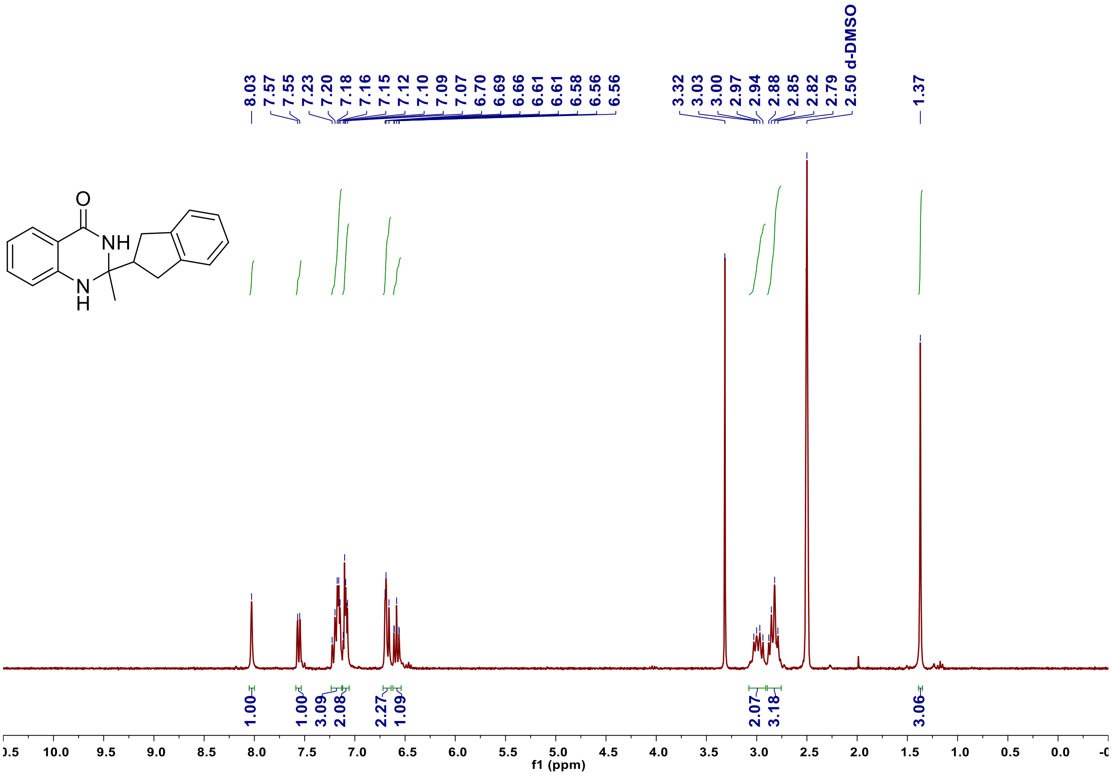

^{1}H NMR (400 MHz, $CDCl_3$) of **1j**, See procedure

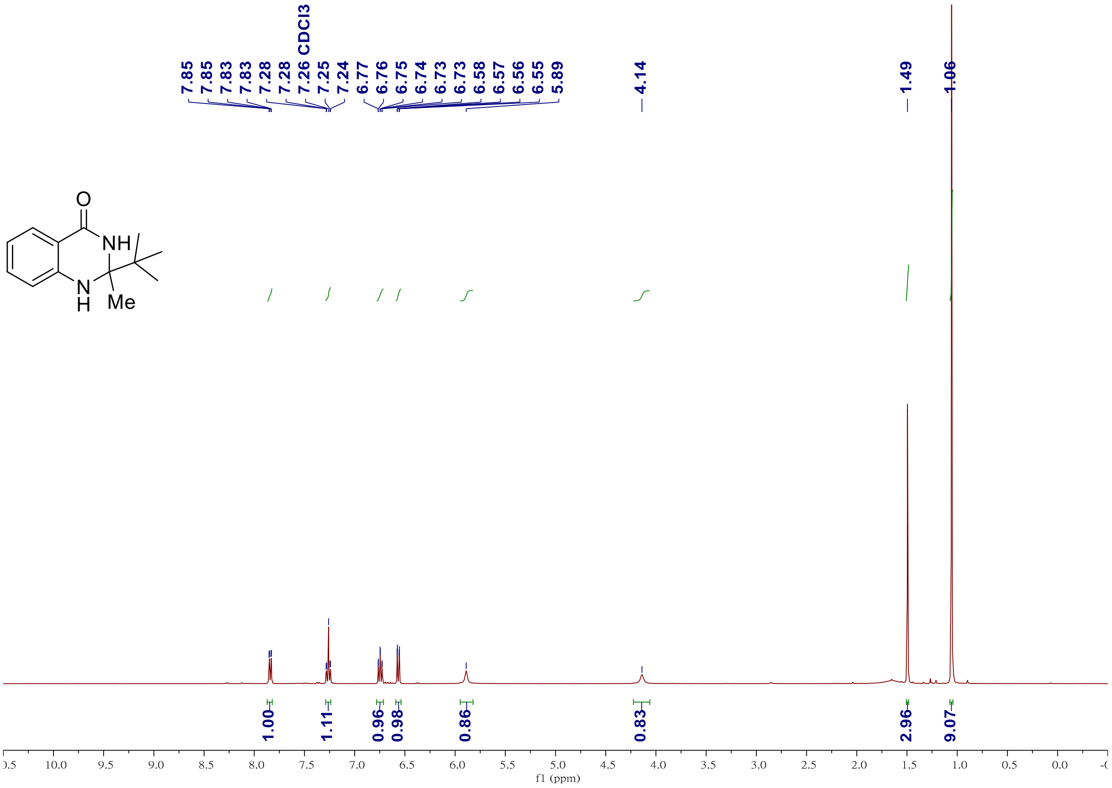

[Back to the contents](#)

^{1}**H NMR** (400 MHz, $CDCl_3$) of **1k**, See procedure

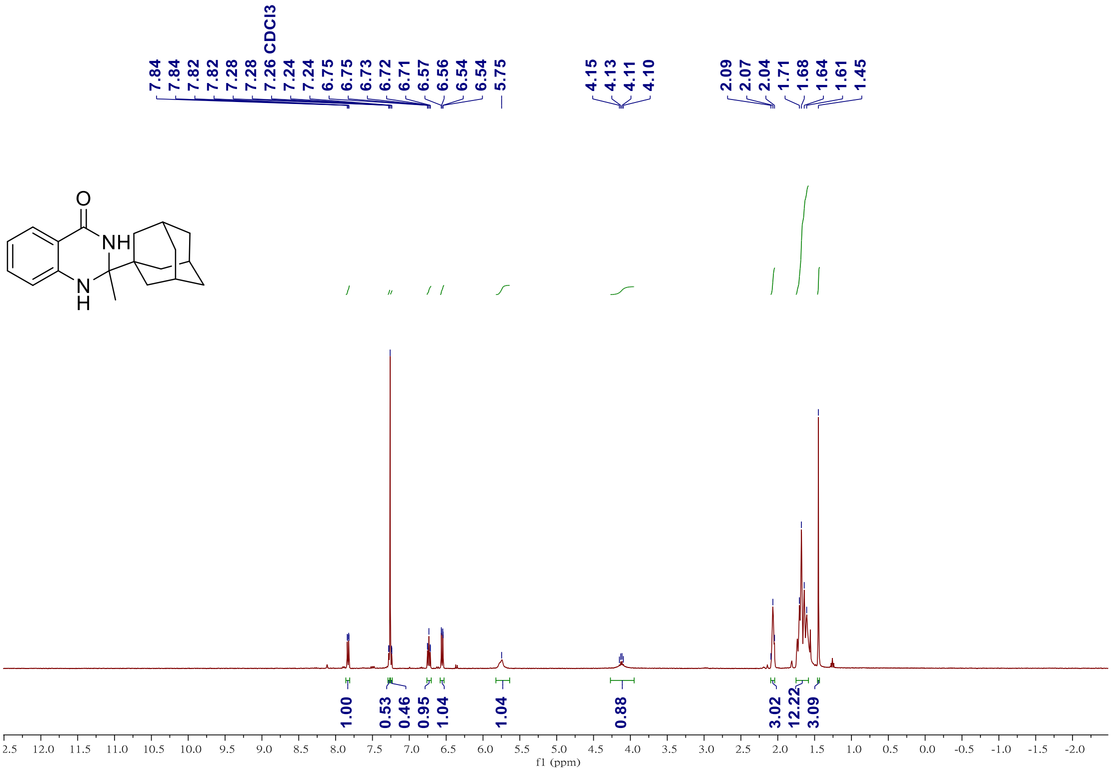

^{1}**H NMR** (300 MHz, DMSO-d_6) of **1l**, See procedure

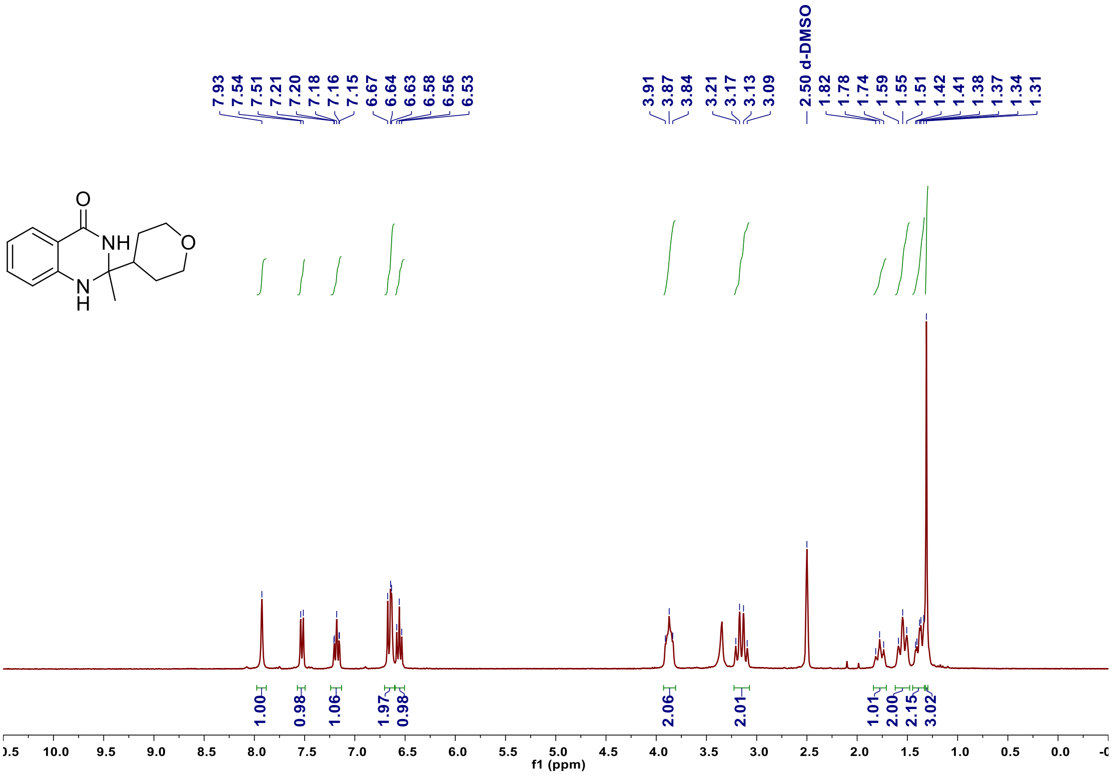

Back to the contents

¹H NMR (400 MHz, $CDCl_3$) of **1m**, See procedure

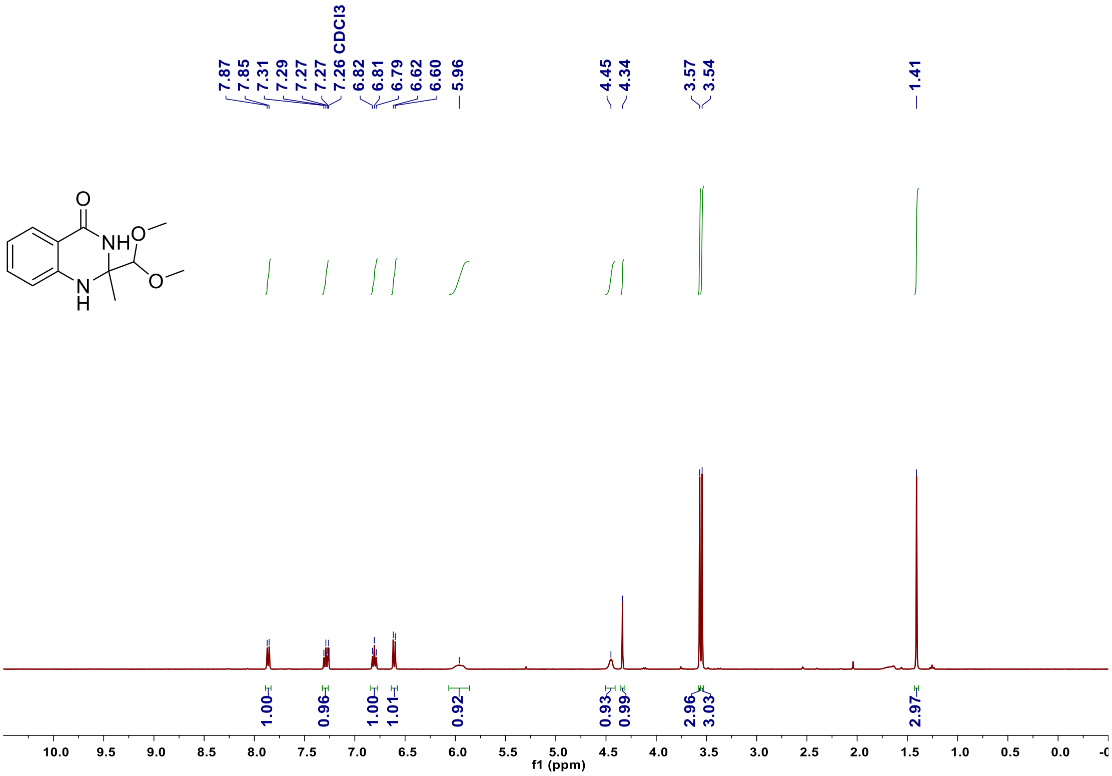

¹³C NMR (101 MHz, $CDCl_3$) of **1m**

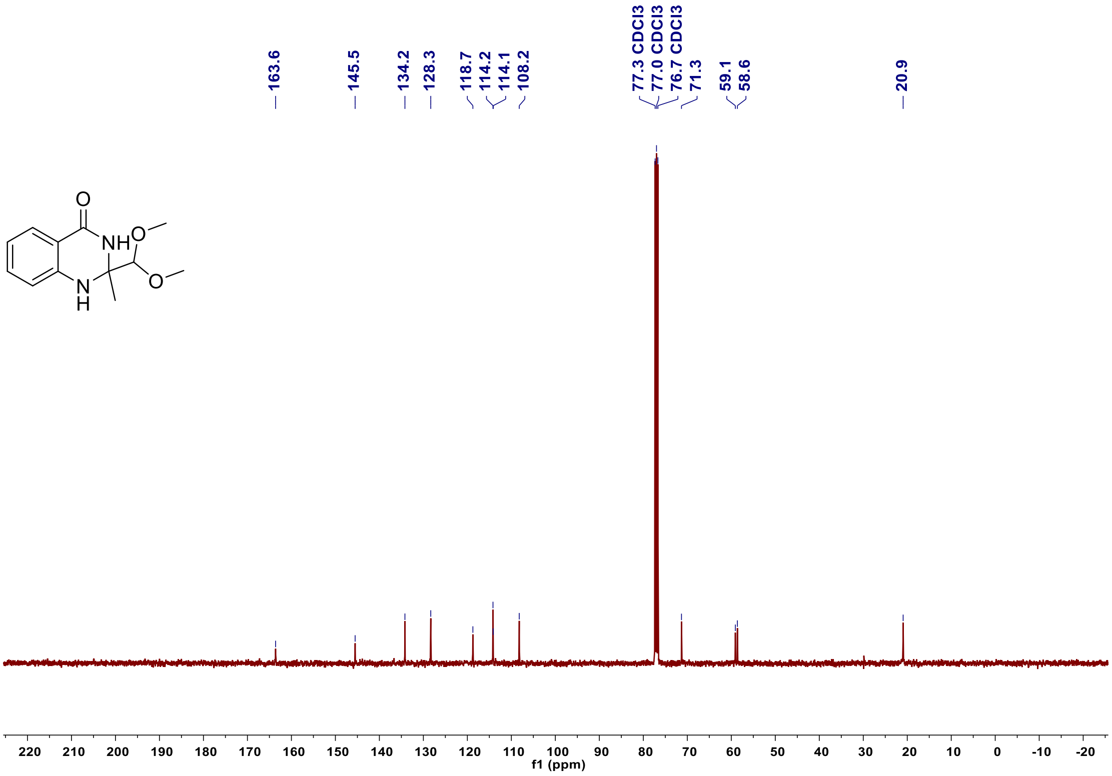

[Back to the contents]

^{1}H NMR (300 MHz, $CDCl_3$) of **2b**, See procedure

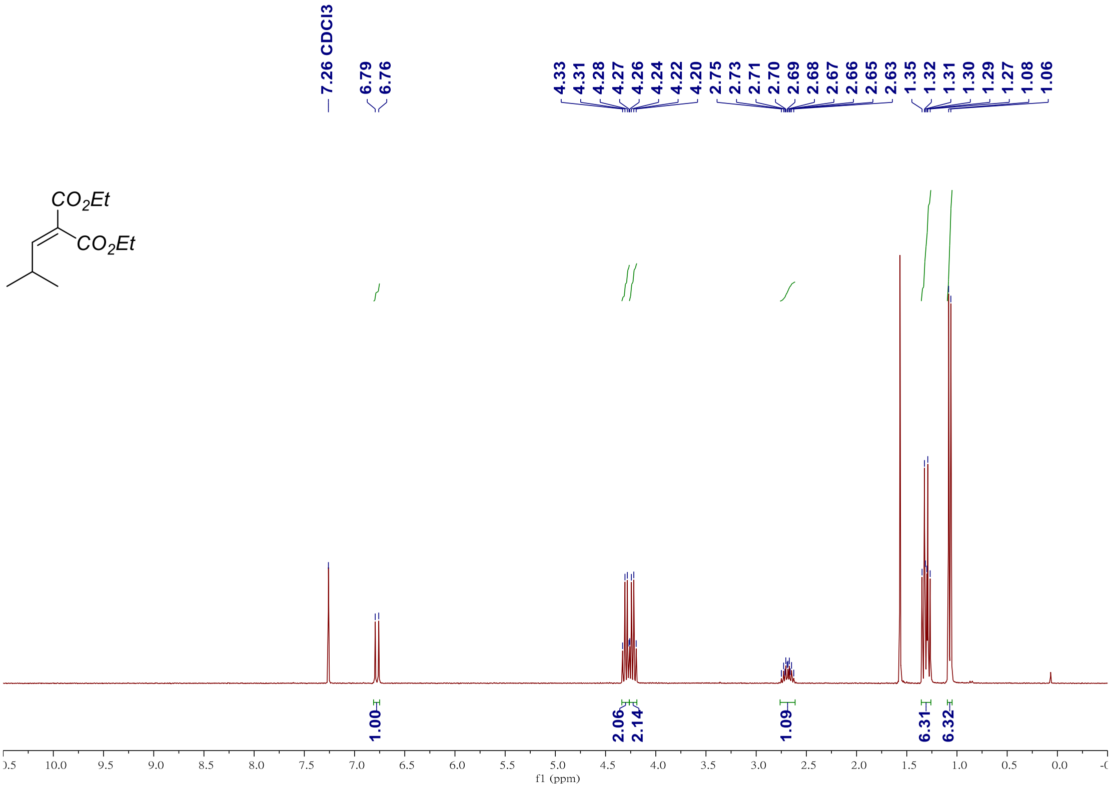

^{1}H NMR (300 MHz, $CDCl_3$) of **2c**, See procedure

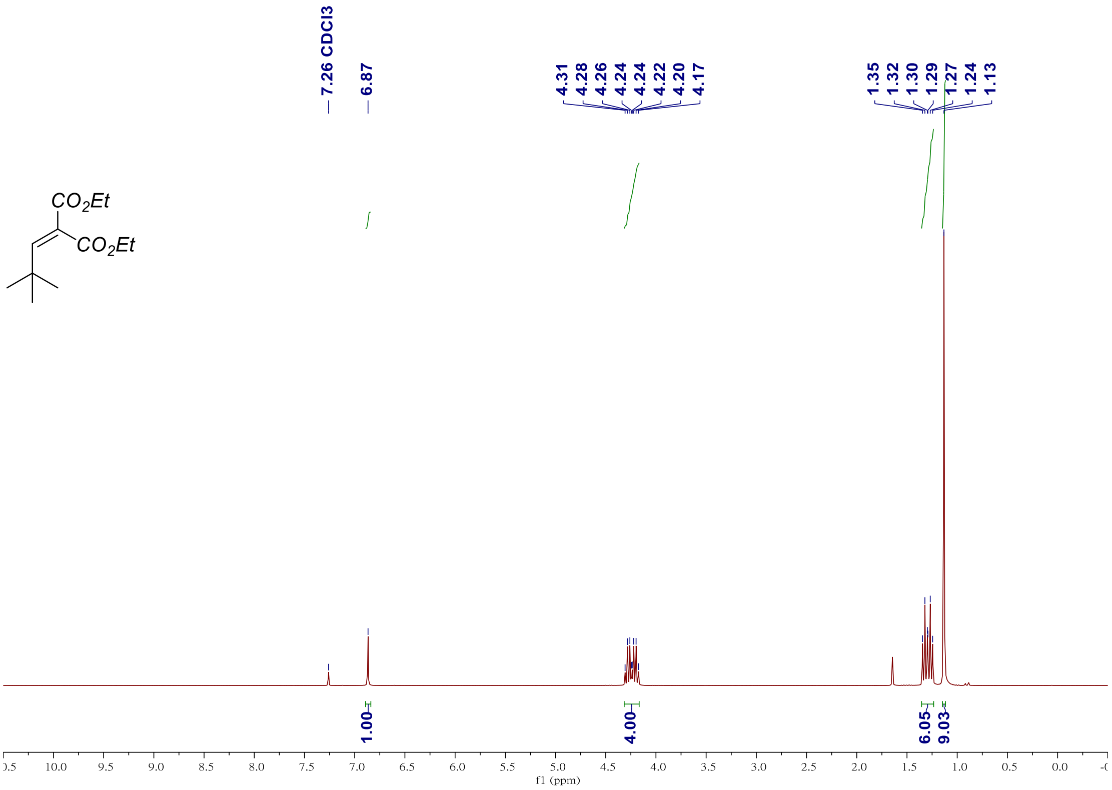

[Back to the contents](#)

^{1}H NMR (300 MHz, $CDCl_3$) of **2d**, [See procedure](#)

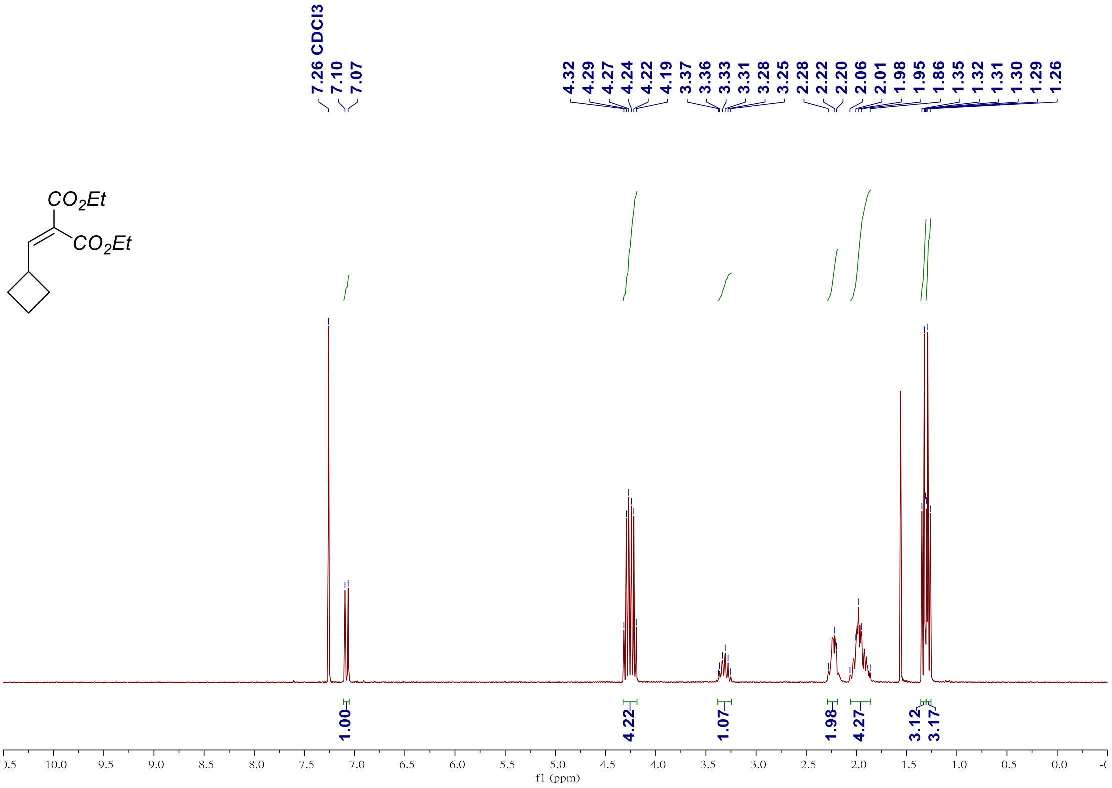

^{13}C{^{1}H} NMR (101 MHz, $CDCl_3$) of **2d**

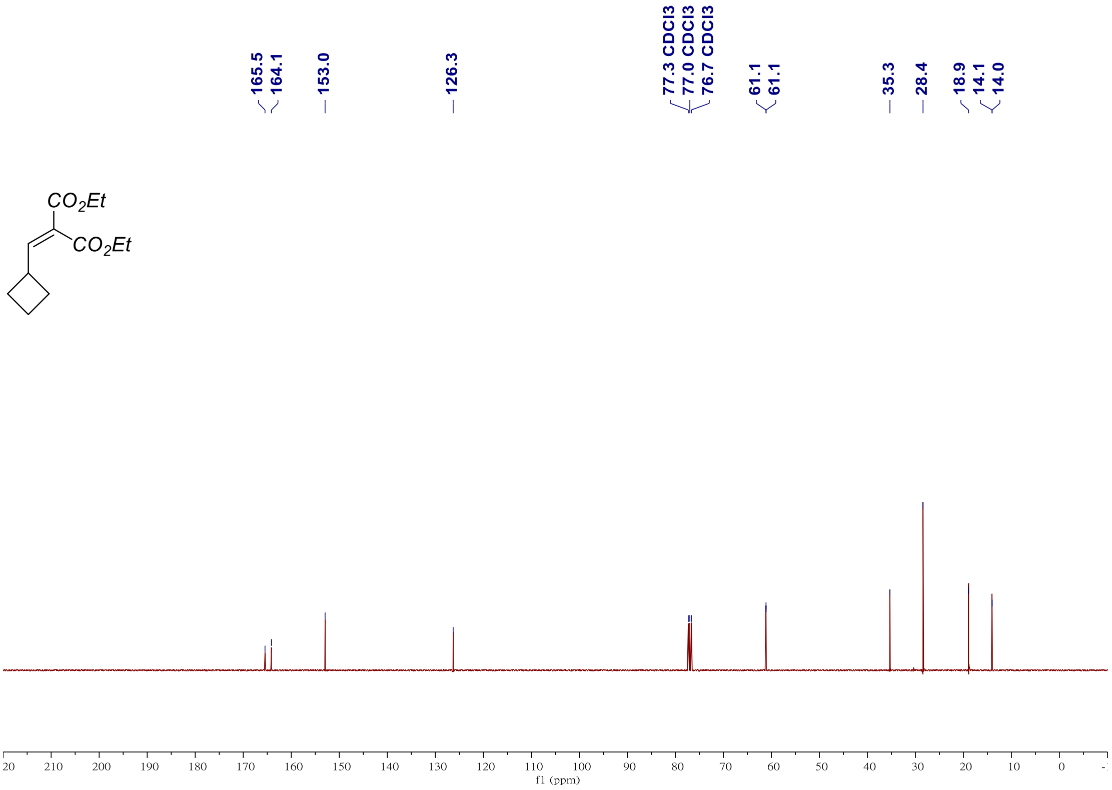

[Back to the contents](#)

^{1}**H NMR** (300 MHz, $CDCl_3$) of **2e**, See procedure

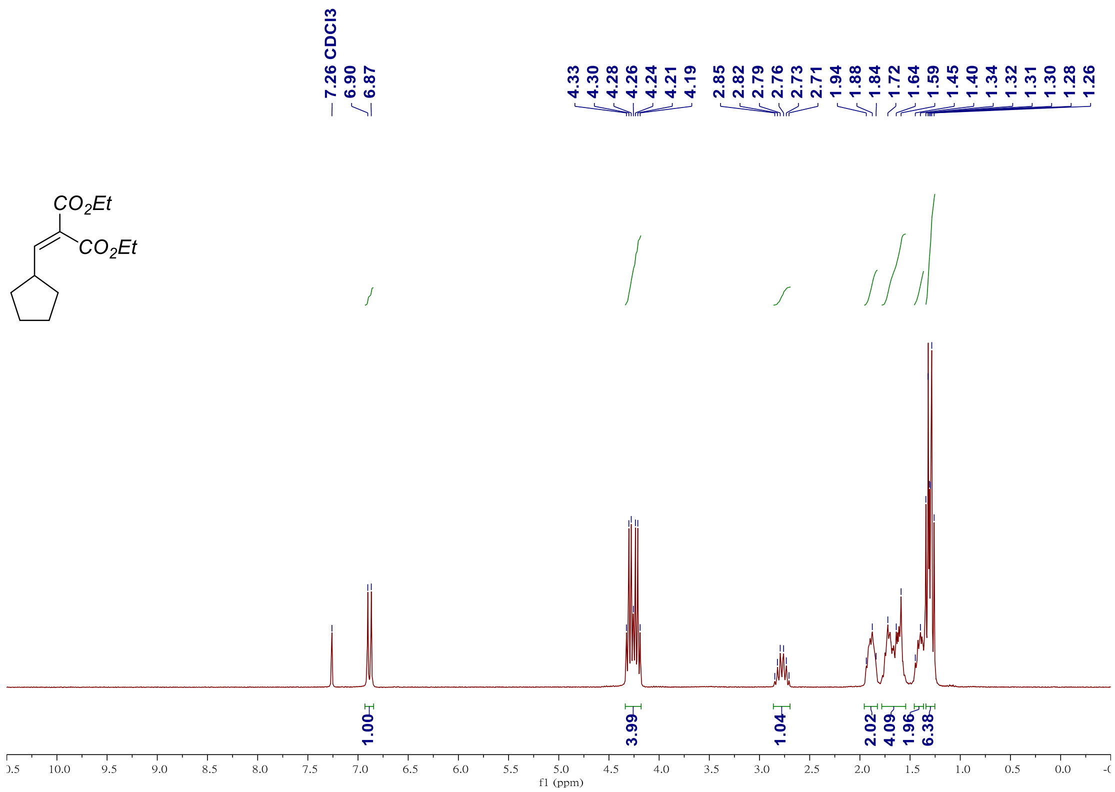

^{1}**H NMR** (300 MHz, $CDCl_3$) of **2f**, See procedure

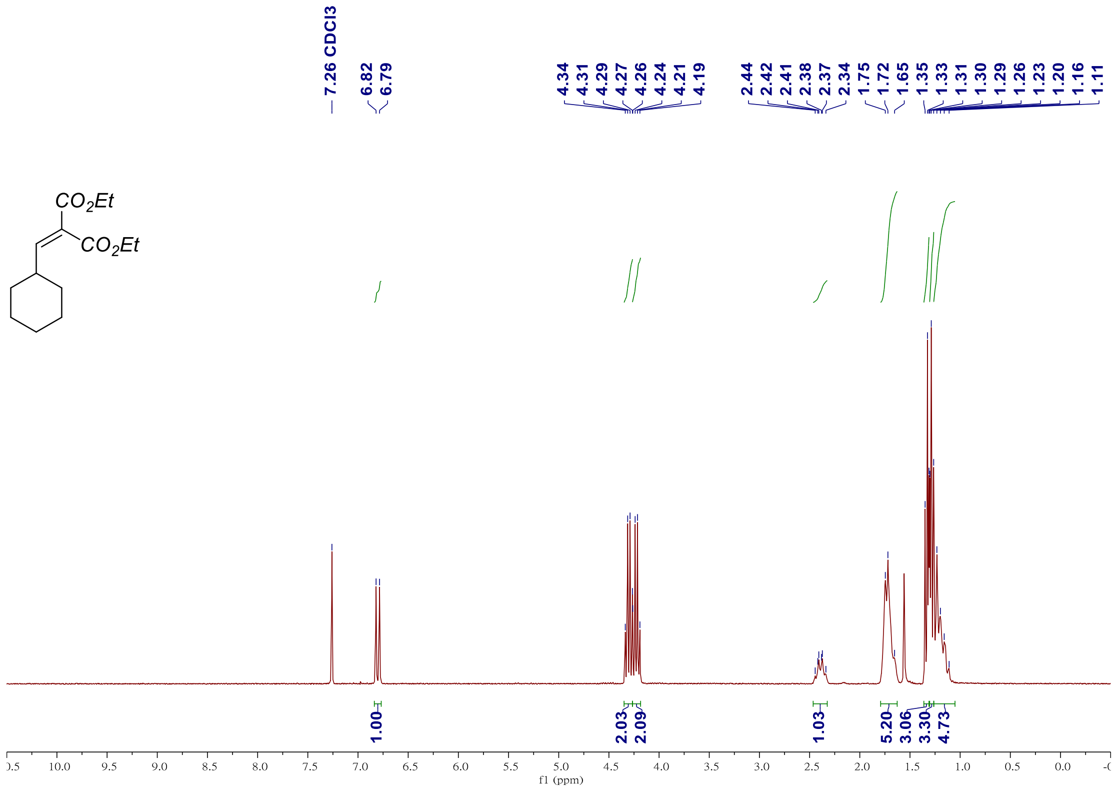

[Back to the contents](#)

^{1}H NMR (300 MHz, $CDCl_3$) of **2h**, See procedure

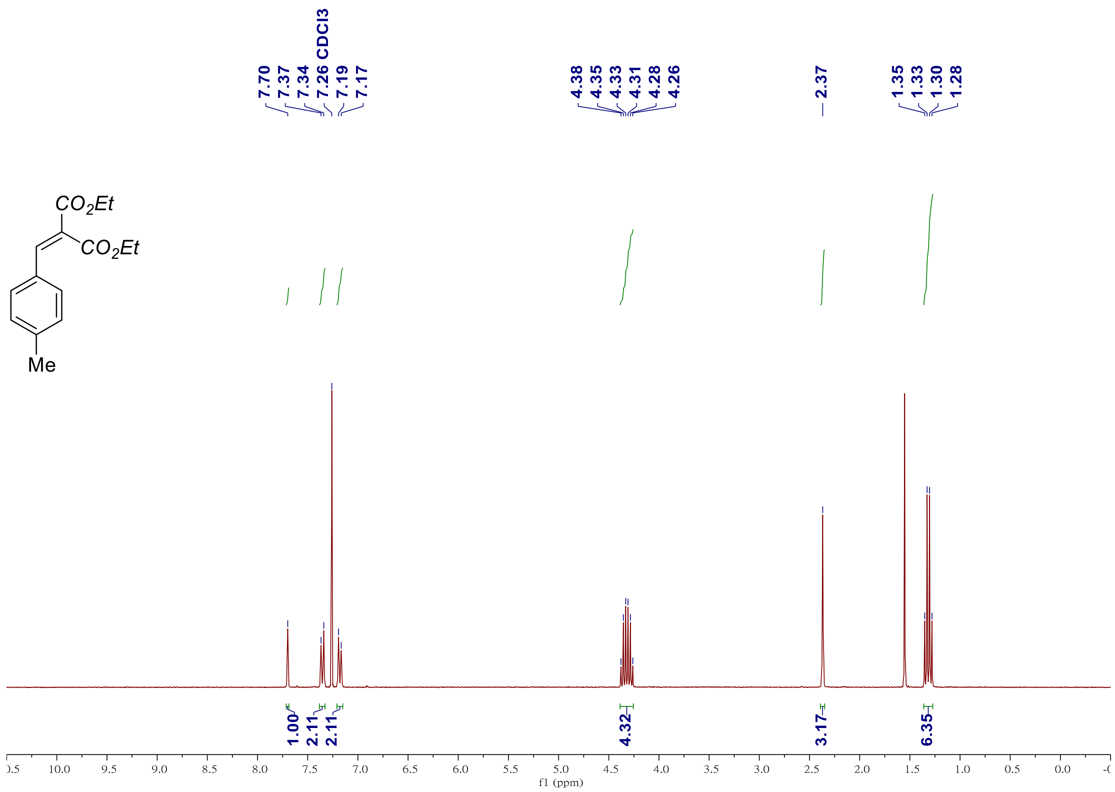

^{1}H NMR (300 MHz, $CDCl_3$) of **2i**, See procedure

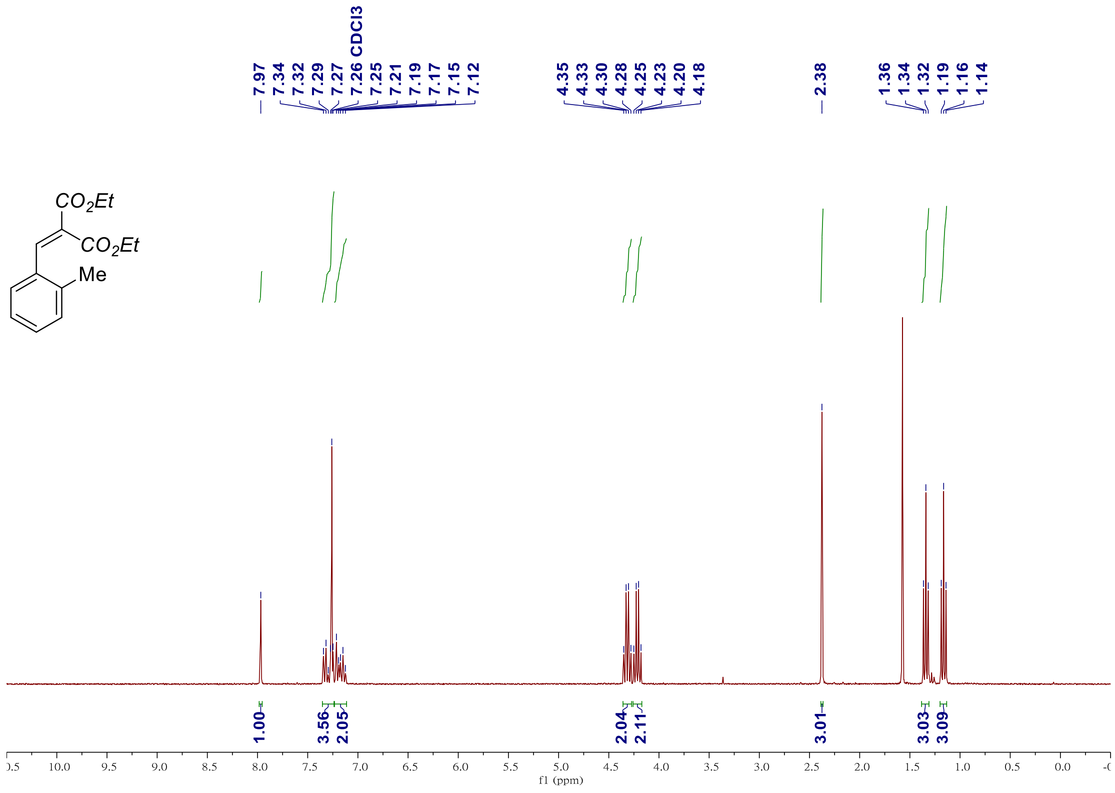

[Back to the contents](#)

^{1}H NMR (300 MHz, $CDCl_3$) of **2j**, [See procedure](#)

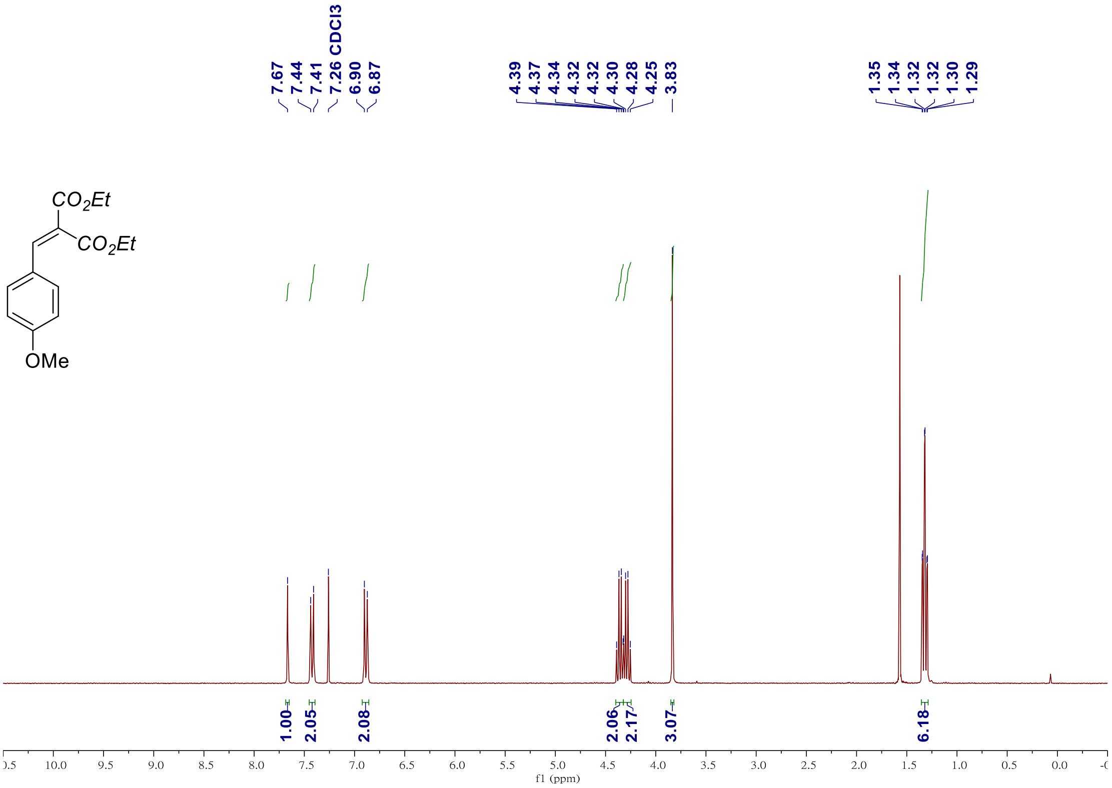

^{1}H NMR (300 MHz, $CDCl_3$) of **2k**, [See procedure](#)

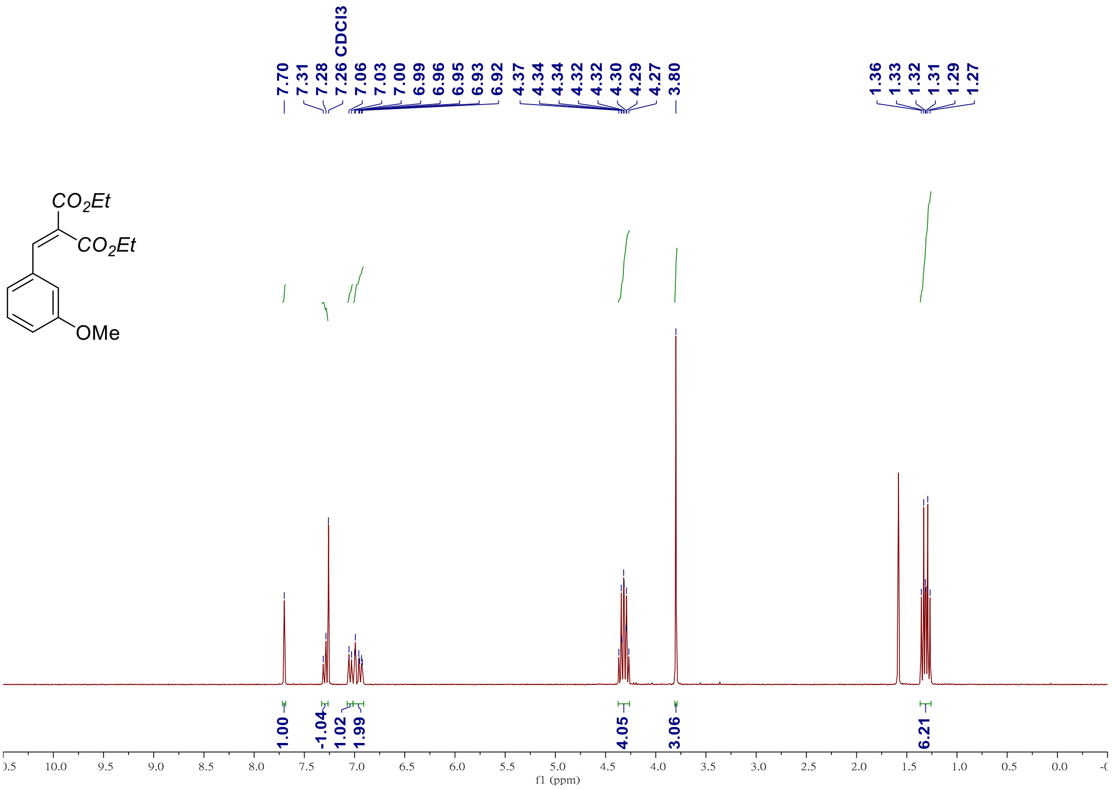

Back to the contents

^{1}H NMR (300 MHz, $CDCl_3$) of **2l**, See procedure

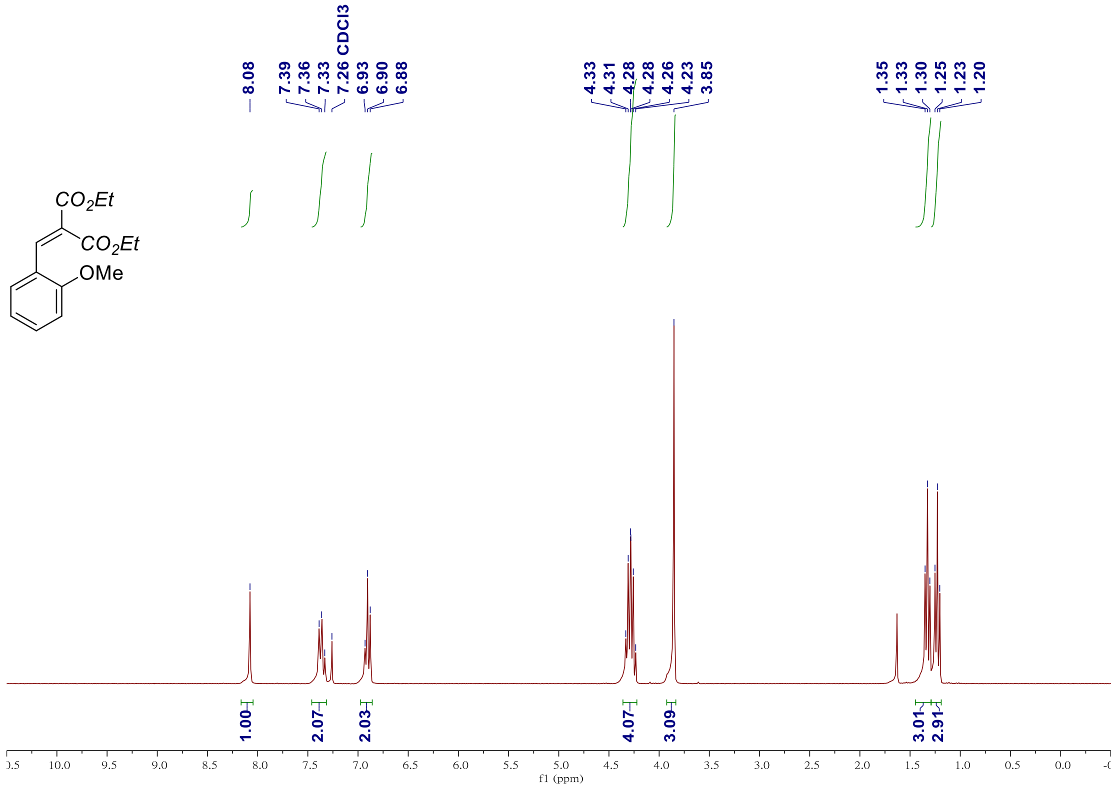

^{1}H NMR (300 MHz, $CDCl_3$) of **2m**, See procedure

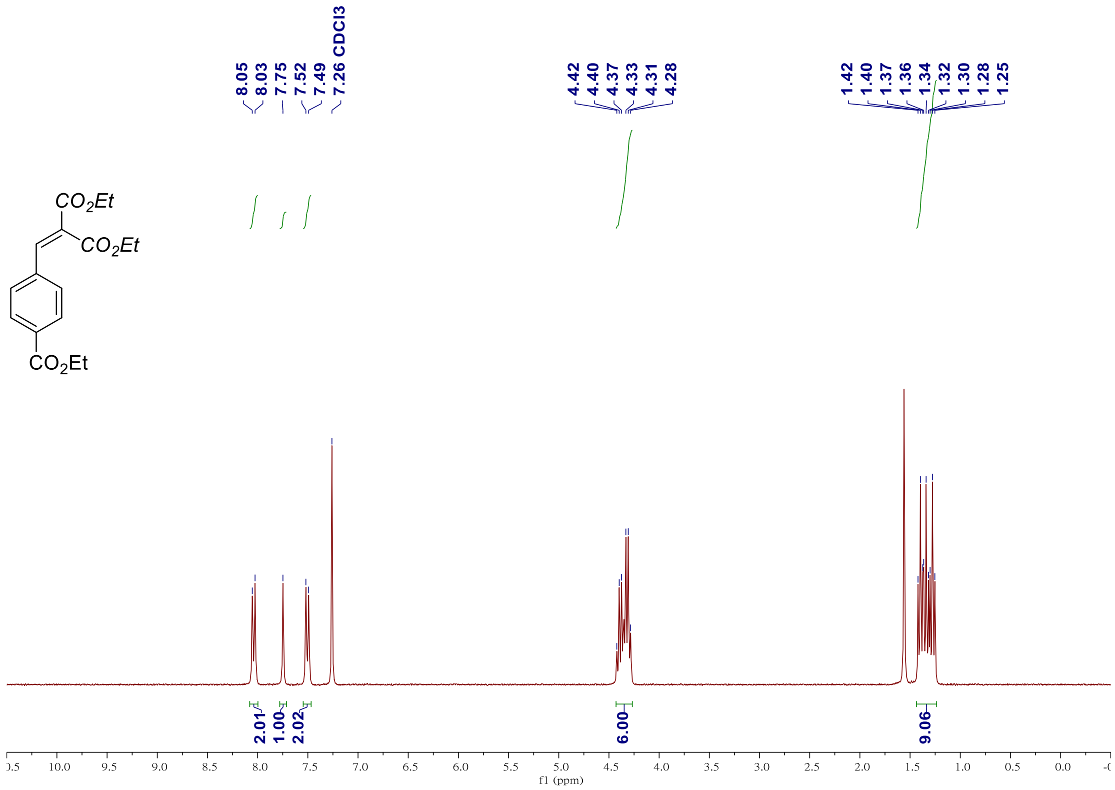

Back to the contents

^{1}H NMR (300 MHz, $CDCl_3$) of **2n**, See procedure

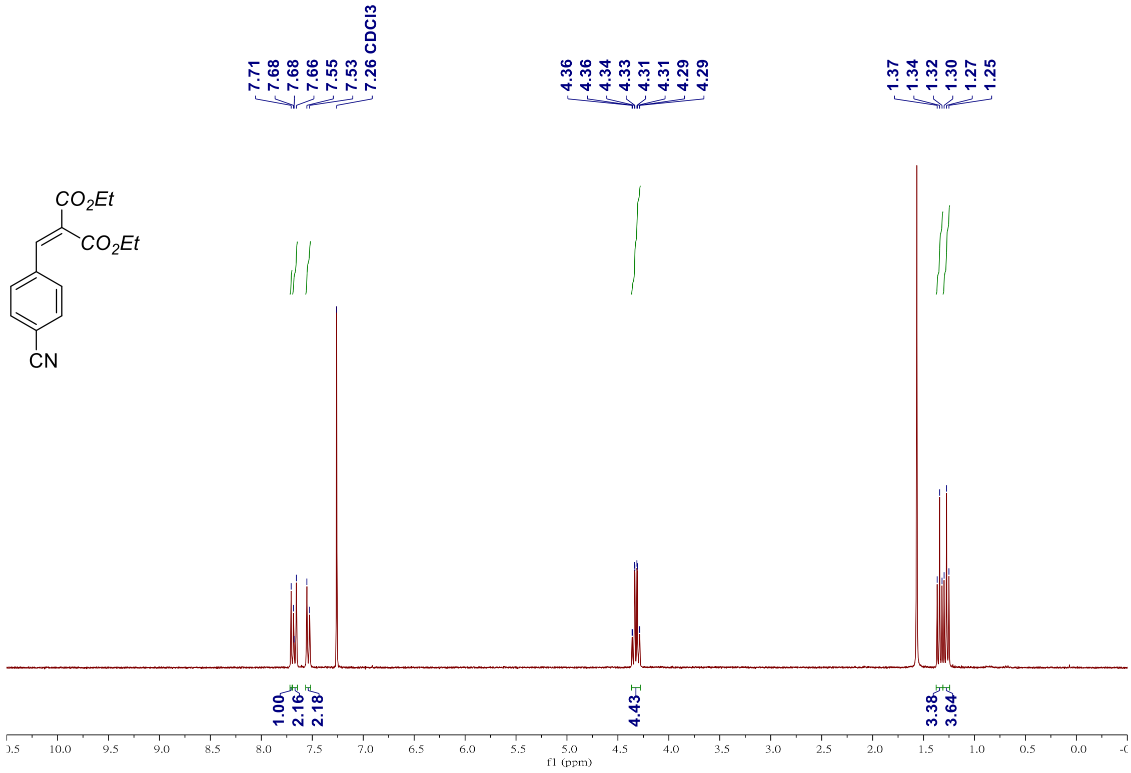

^{1}H NMR (300 MHz, $CDCl_3$) of **2o**, See procedure

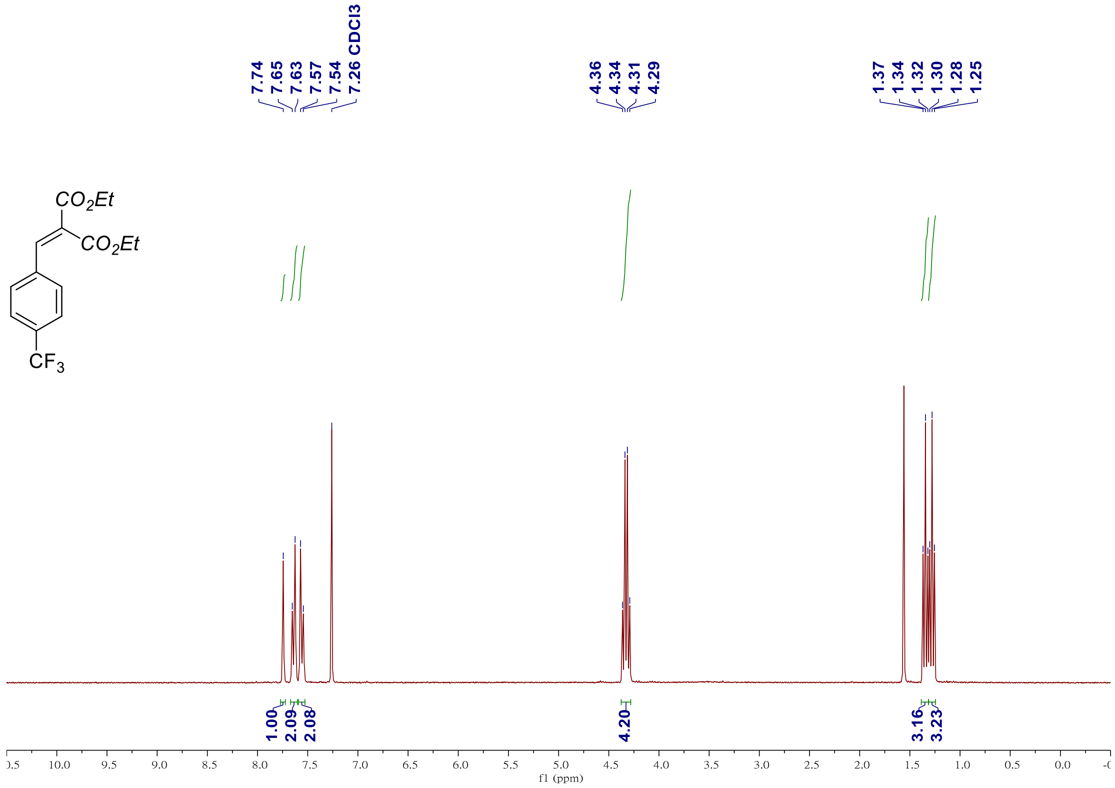

[Back to the contents](#)

^{1}H NMR (300 MHz, $CDCl_3$) of **2p**, [See procedure](#)

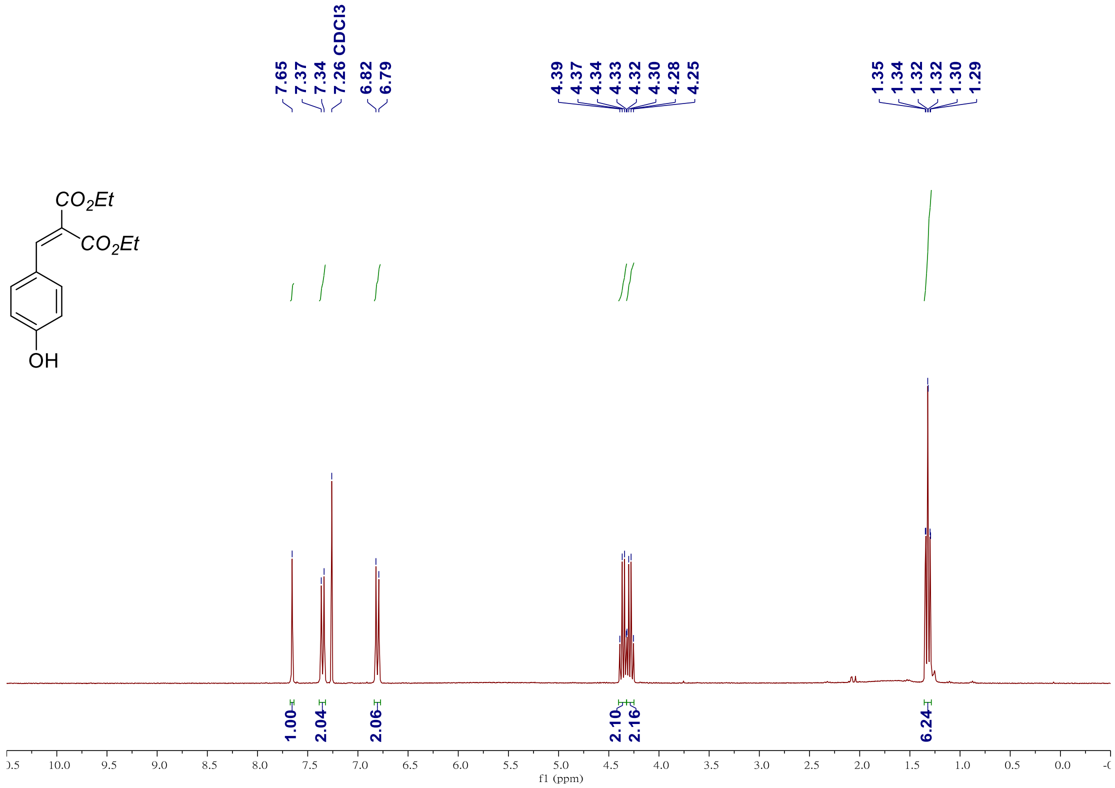

^{1}H NMR (300 MHz, $CDCl_3$) of **2q**, [See procedure](#)

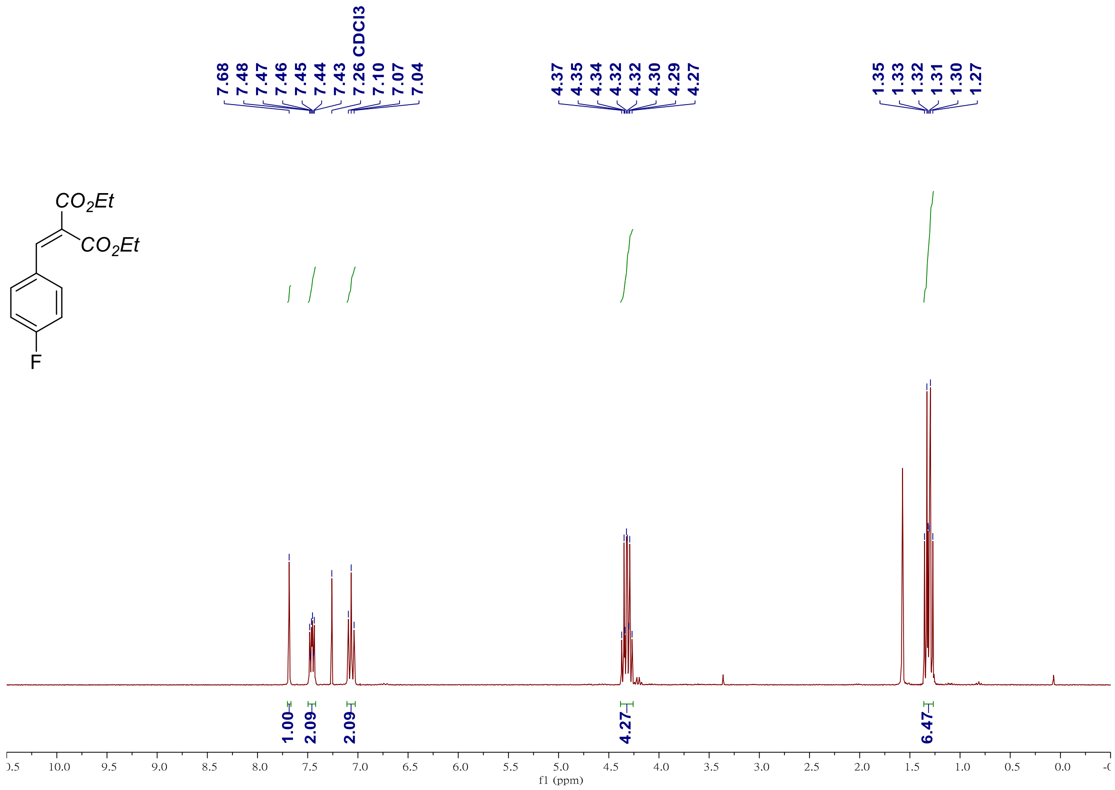

Back to the contents

^{1}H NMR (300 MHz, $CDCl_3$) of **2r**, See procedure

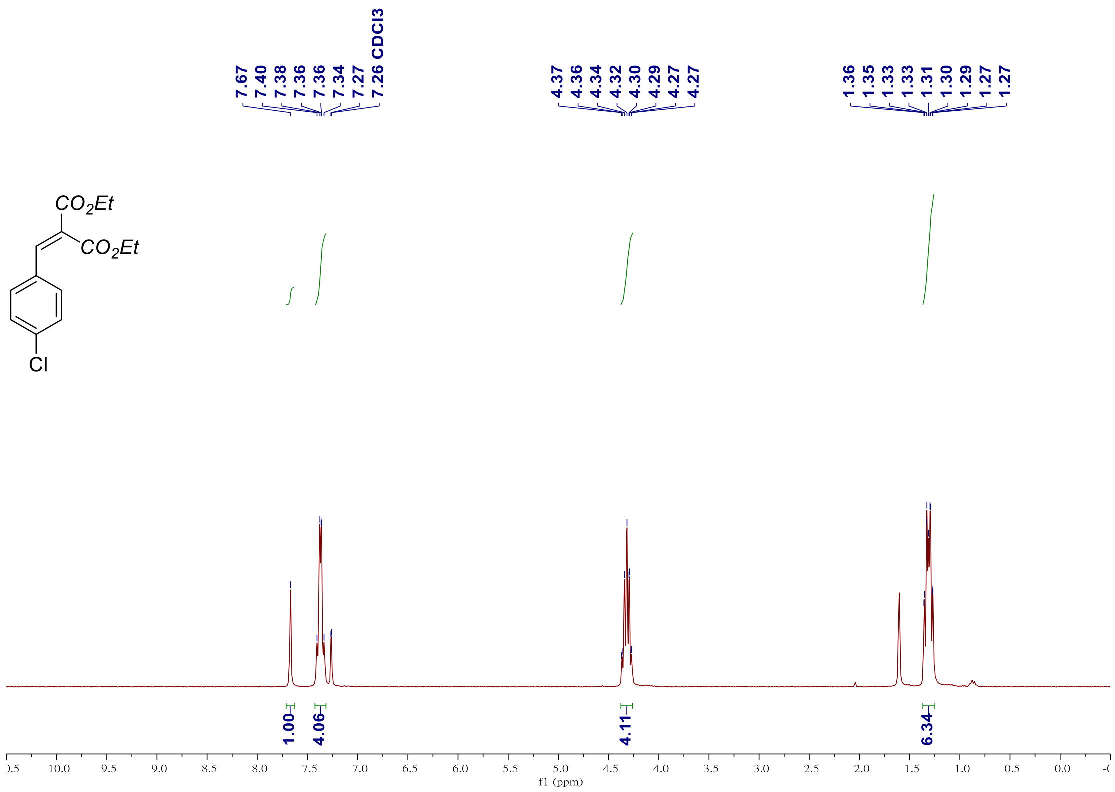

^{1}H NMR (300 MHz, $CDCl_3$) of **2s**, See procedure

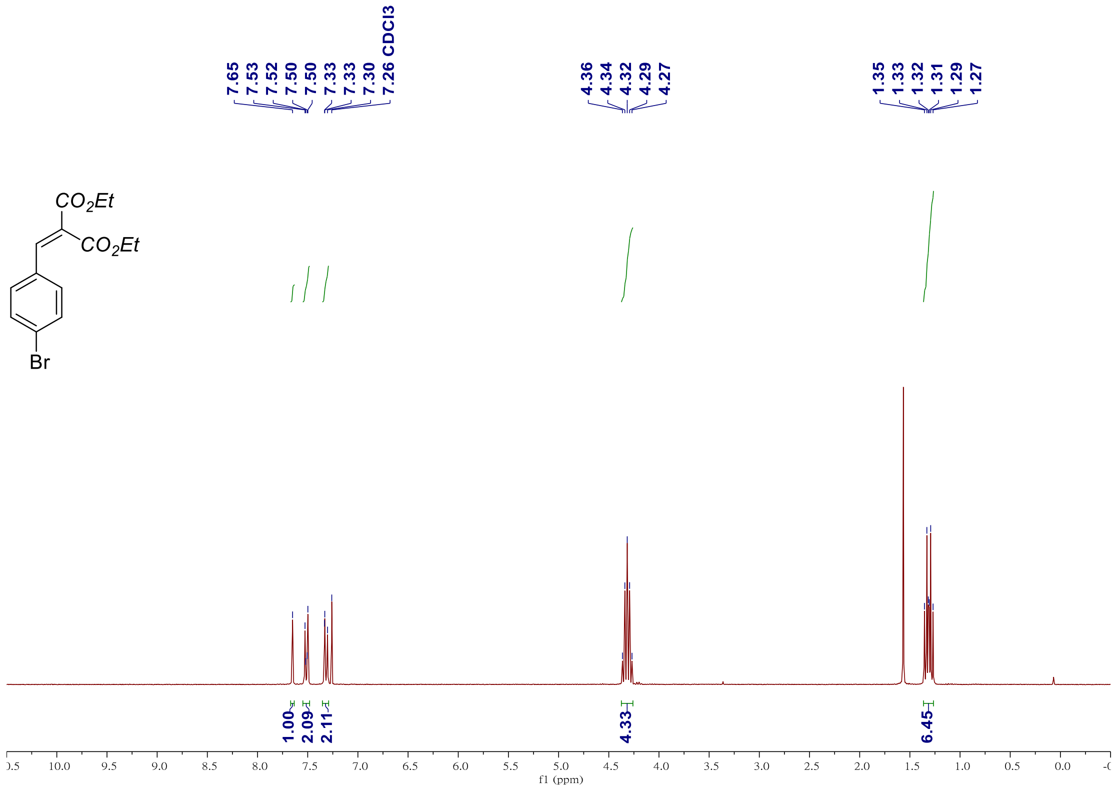

Back to the contents

^{1}H NMR (300 MHz, $CDCl_3$) of **2t**, See procedure

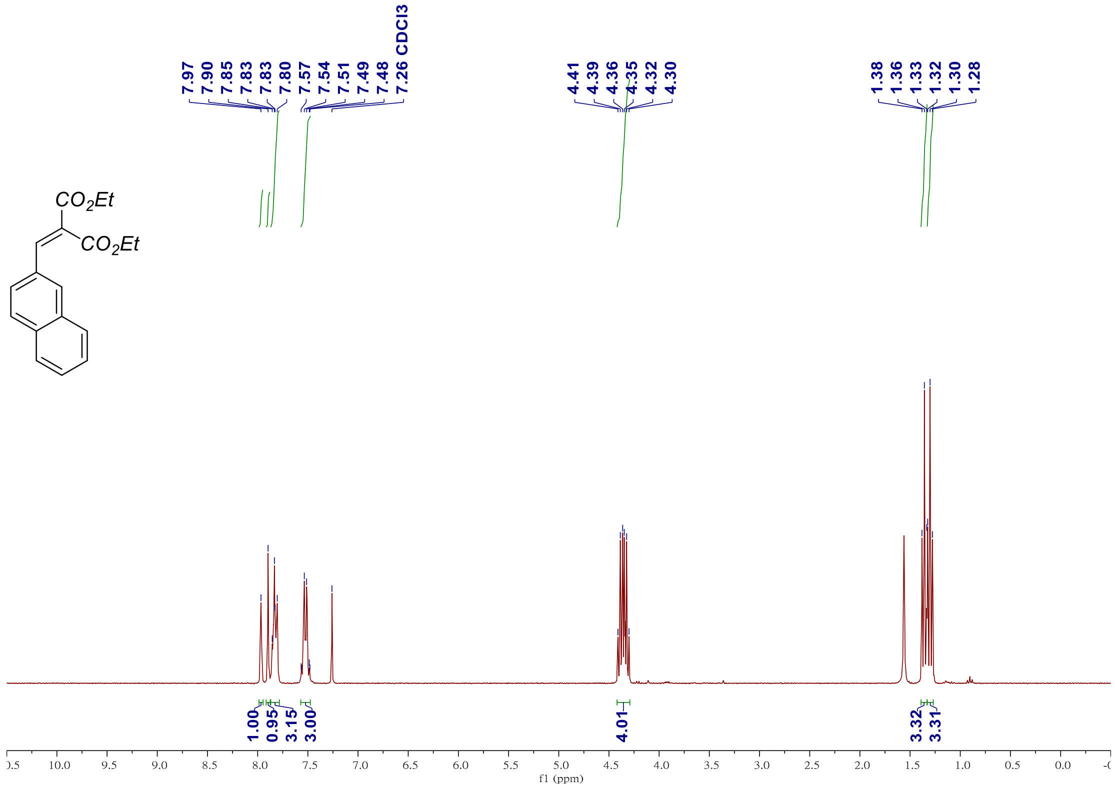

^{1}H NMR (300 MHz, $CDCl_3$) of **2u**, See procedure

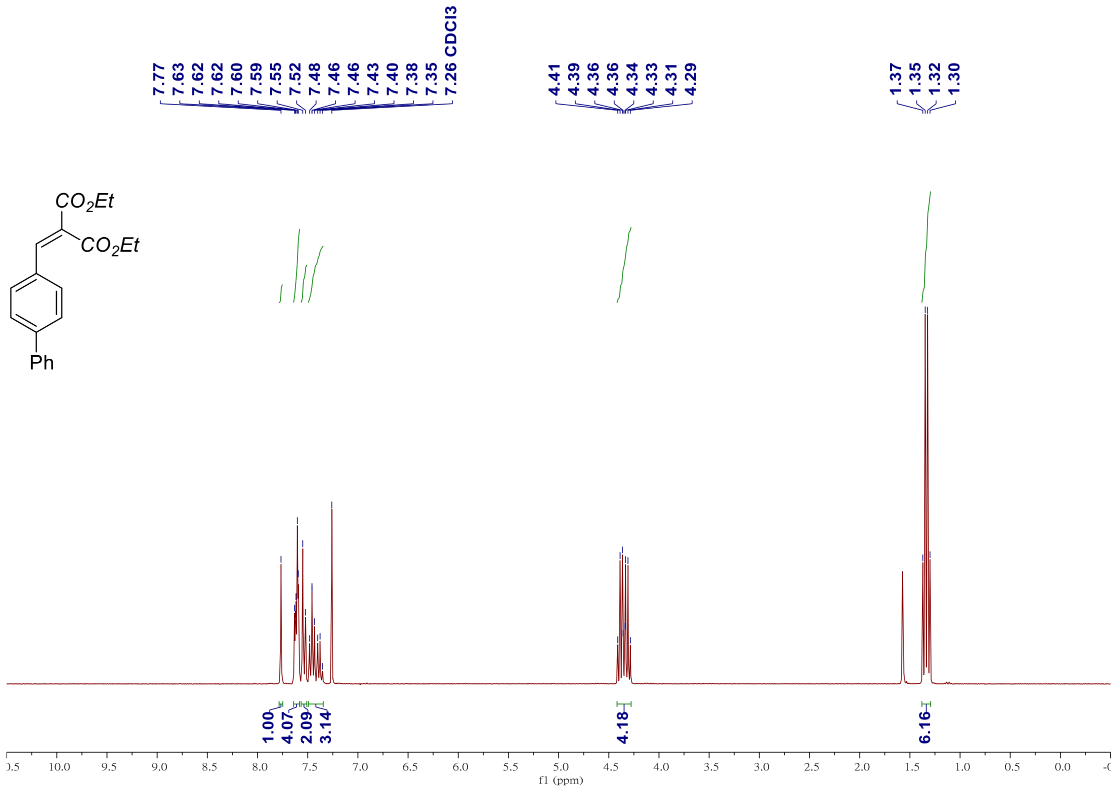

Back to the contents

^{1}H NMR (300 MHz, $CDCl_3$) of **2v**, See procedure

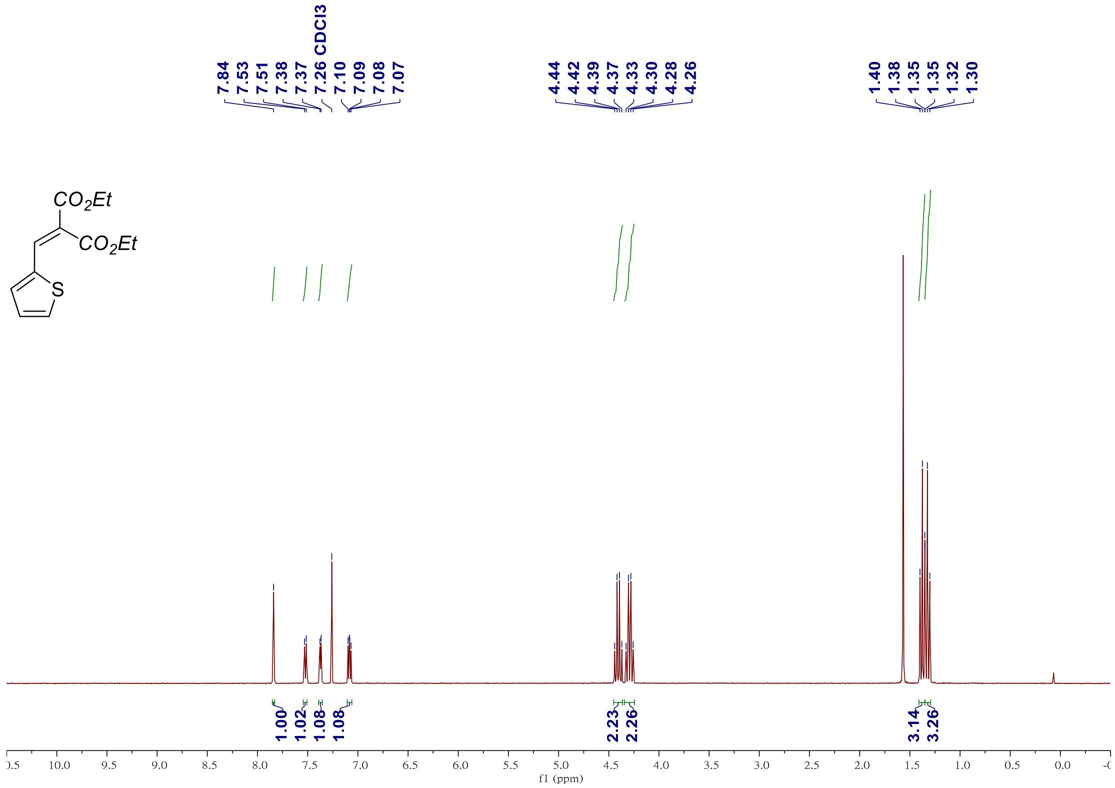

^{1}H NMR (300 MHz, $CDCl_3$) of **2w**, See procedure

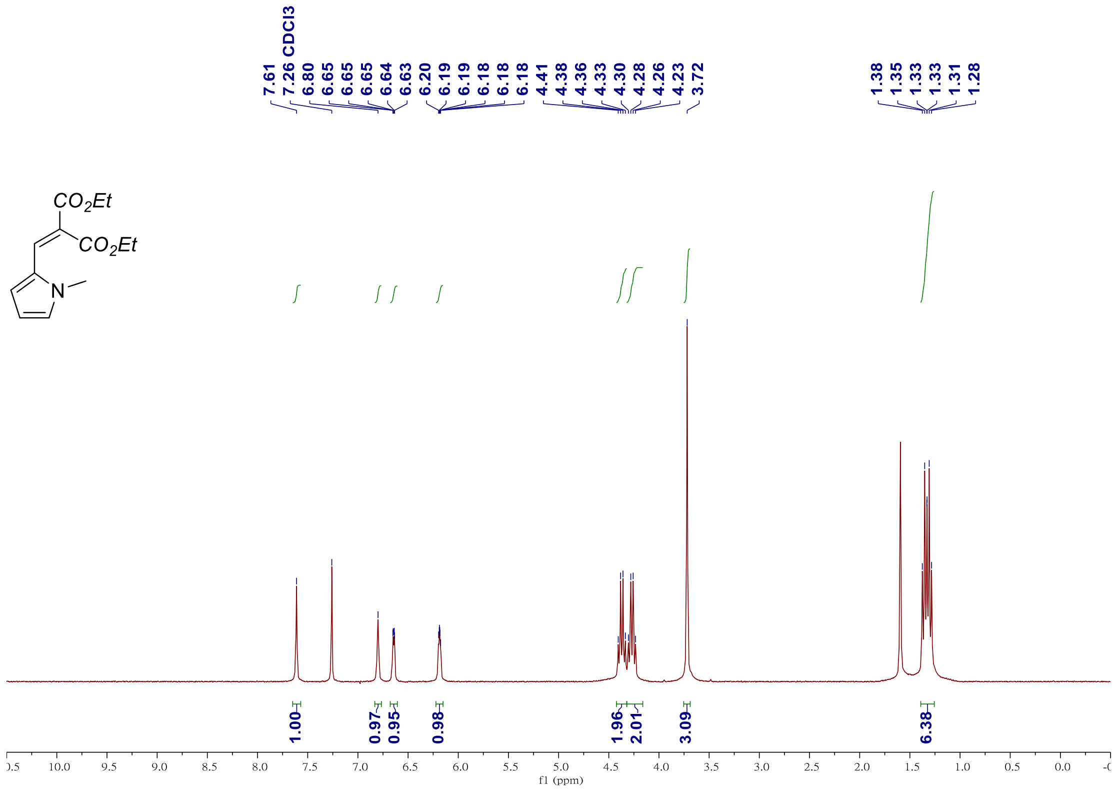

Back to the contents

^{1}H NMR (300 MHz, $CDCl_3$) of **2x**, See procedure

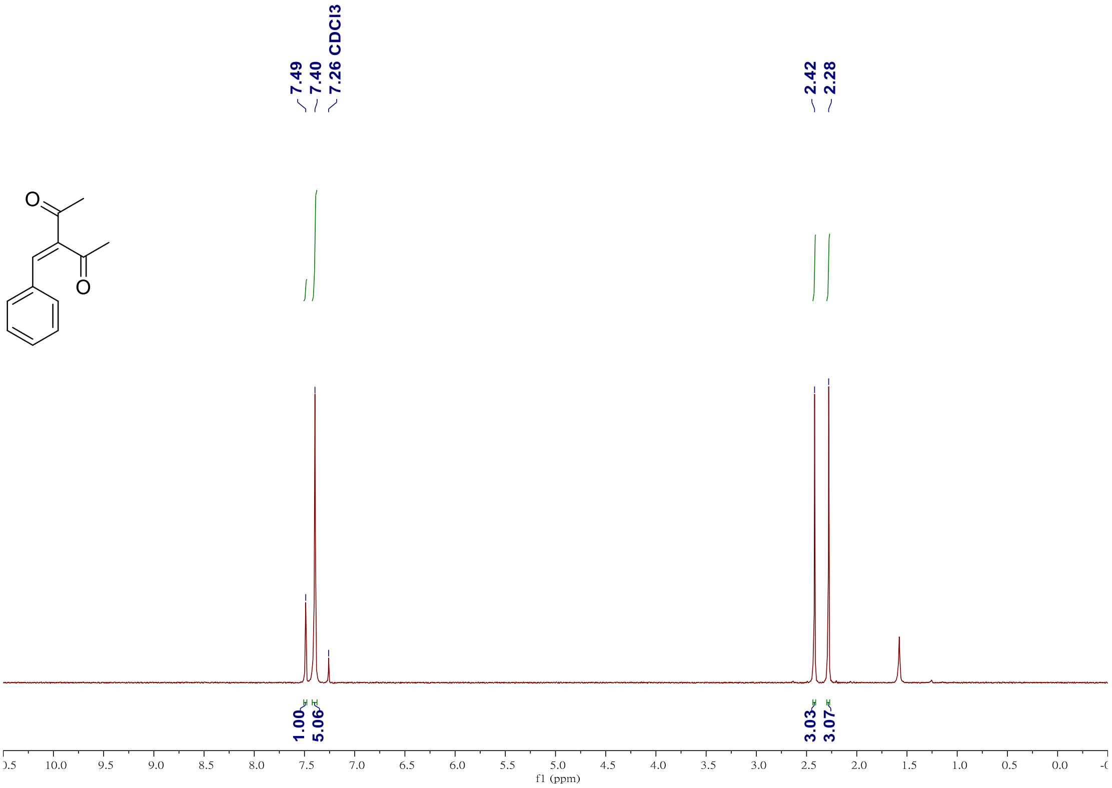

^{1}H NMR (300 MHz, $CDCl_3$) of **2y**, See procedure

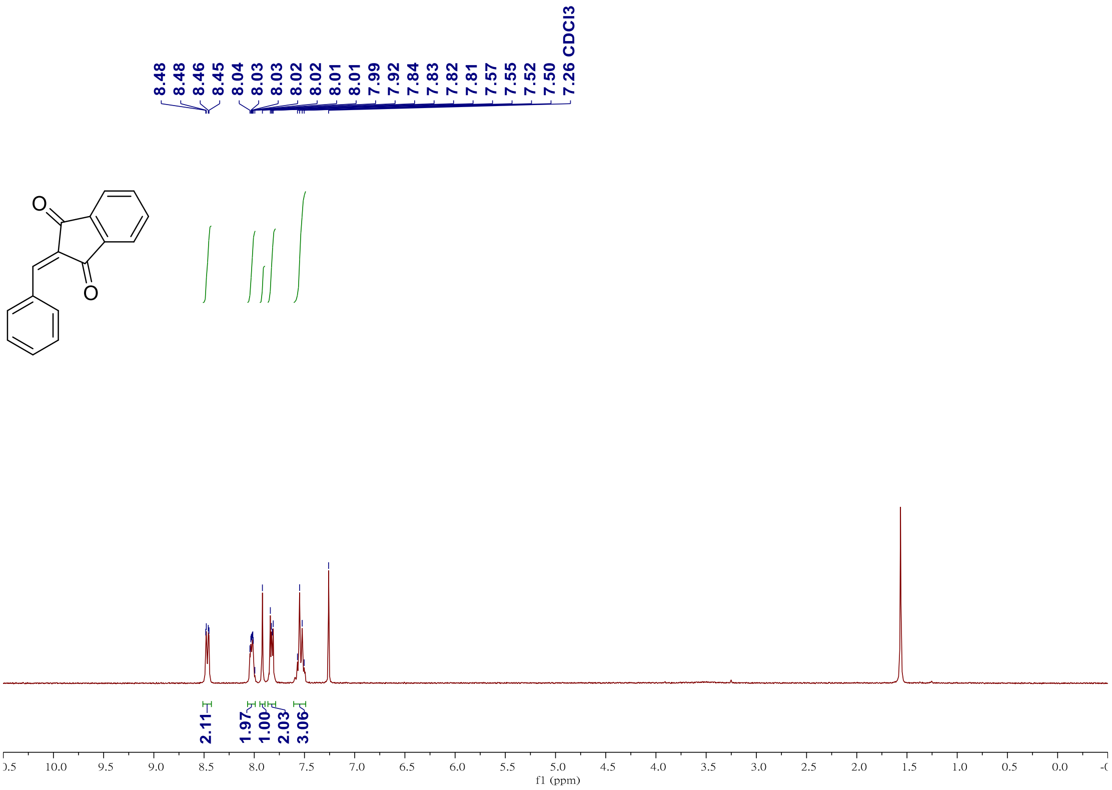

[Back to the contents](#)

^{1}H NMR (300 MHz, $CDCl_3$) of **2z**, See procedure

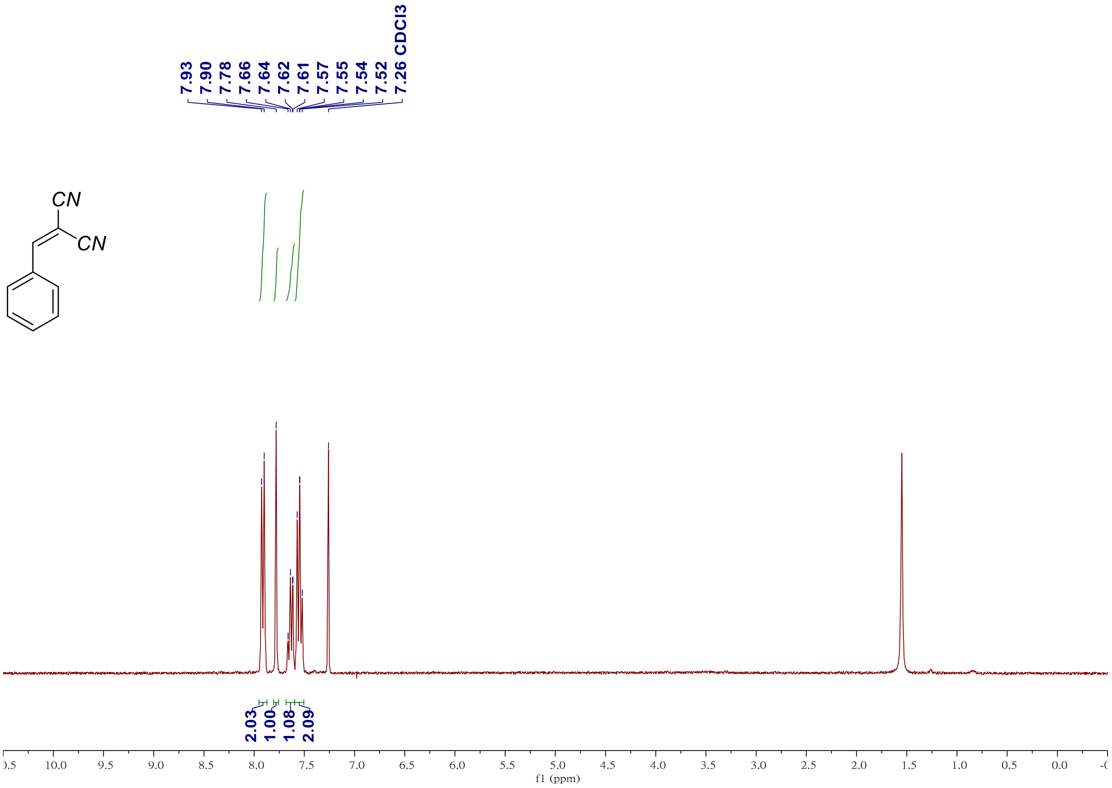

^{1}H NMR (300 MHz, $CDCl_3$) of **2aa**, See procedure

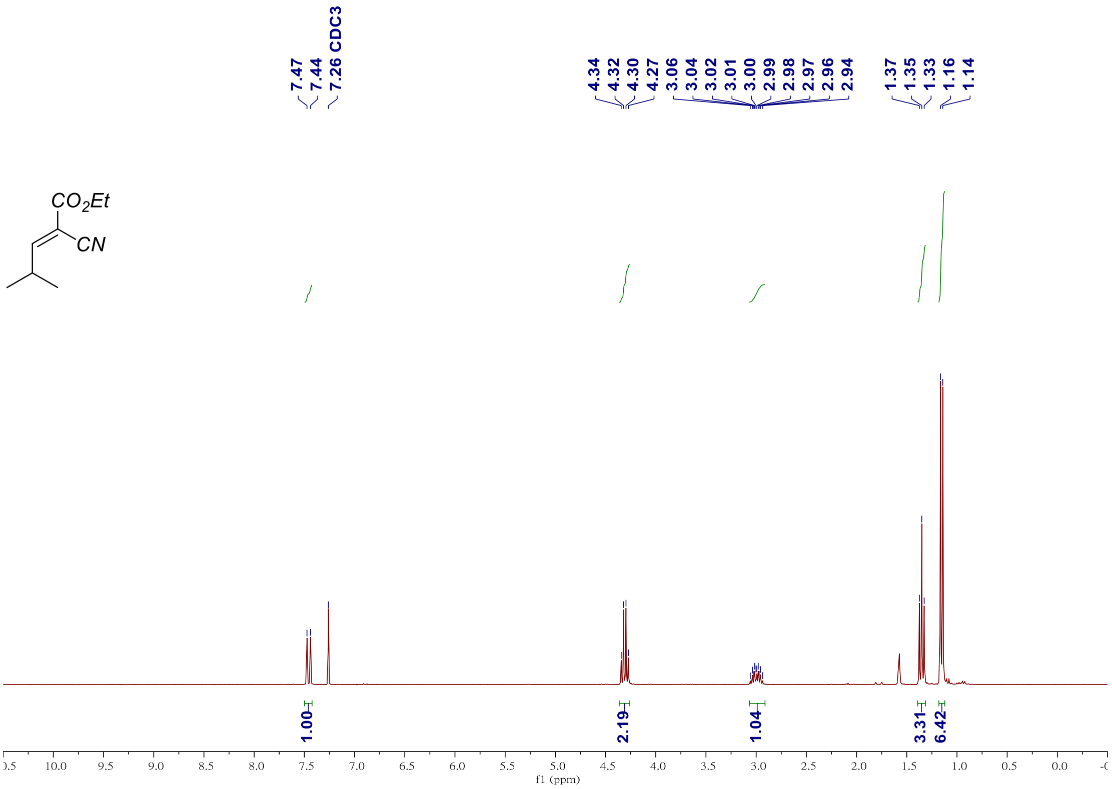

Back to the contents

^{1}H NMR (300 MHz, $CDCl_3$) of **2ab**, See procedure

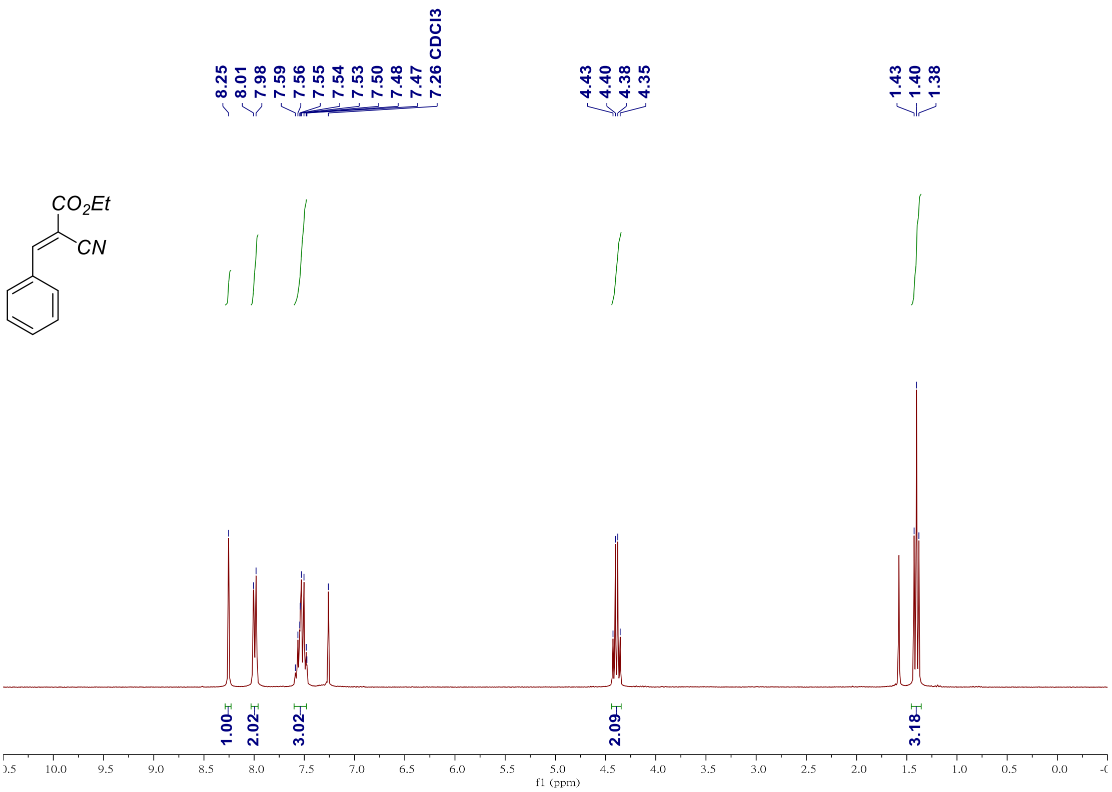

^{1}H NMR (400 MHz, $CDCl_3$) of **2ad**, See procedure

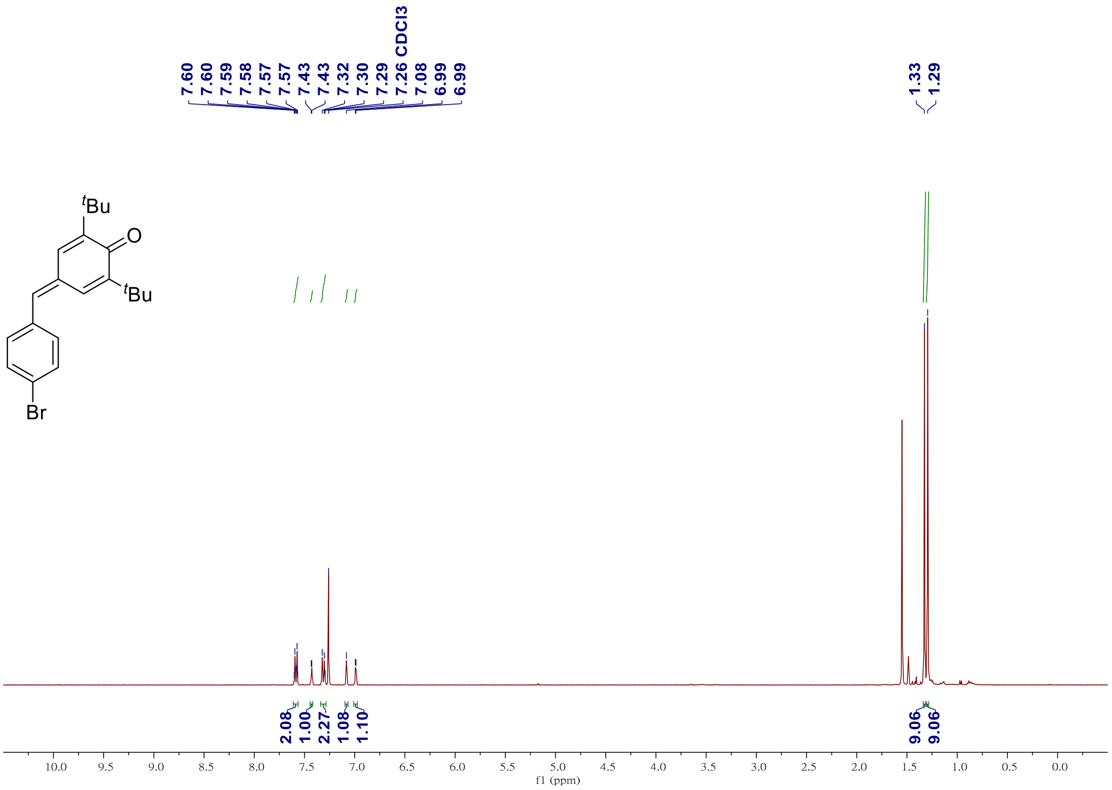

[Back to the contents](#)

¹H NMR (300 MHz, $CDCl_3$) of **2ae**, [See procedure](#)

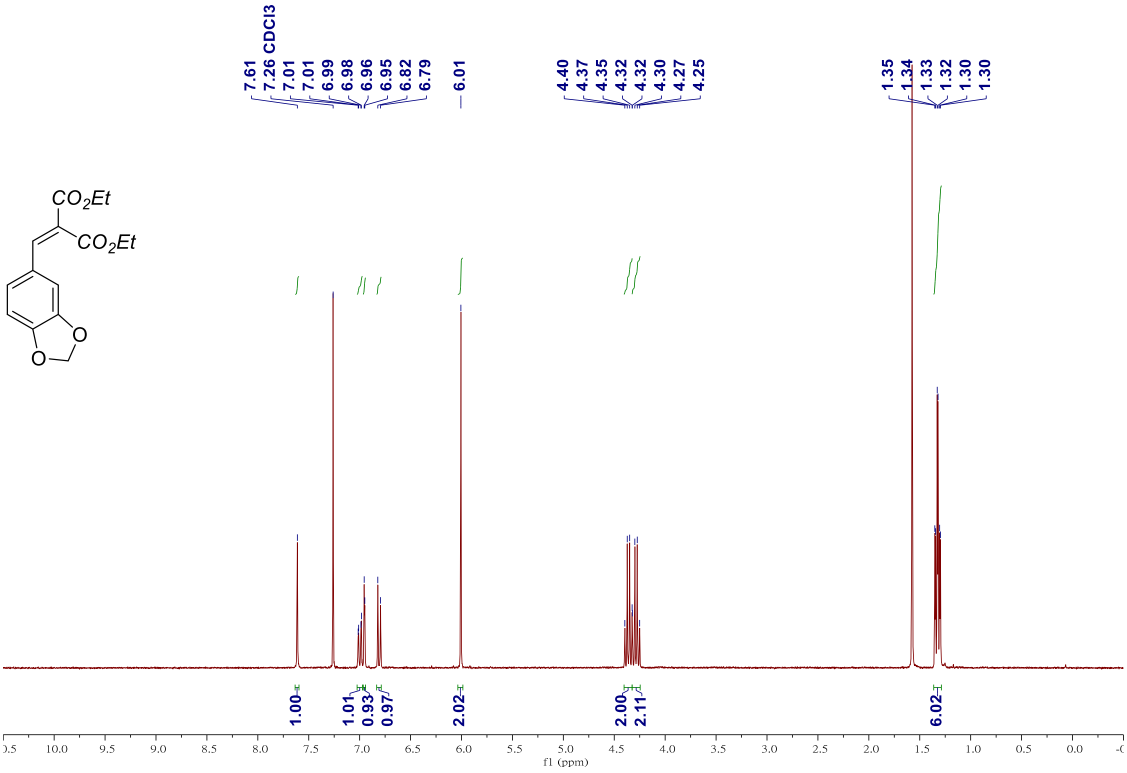

¹H NMR (300 MHz, $CDCl_3$) of **2af**, [See procedure](#)

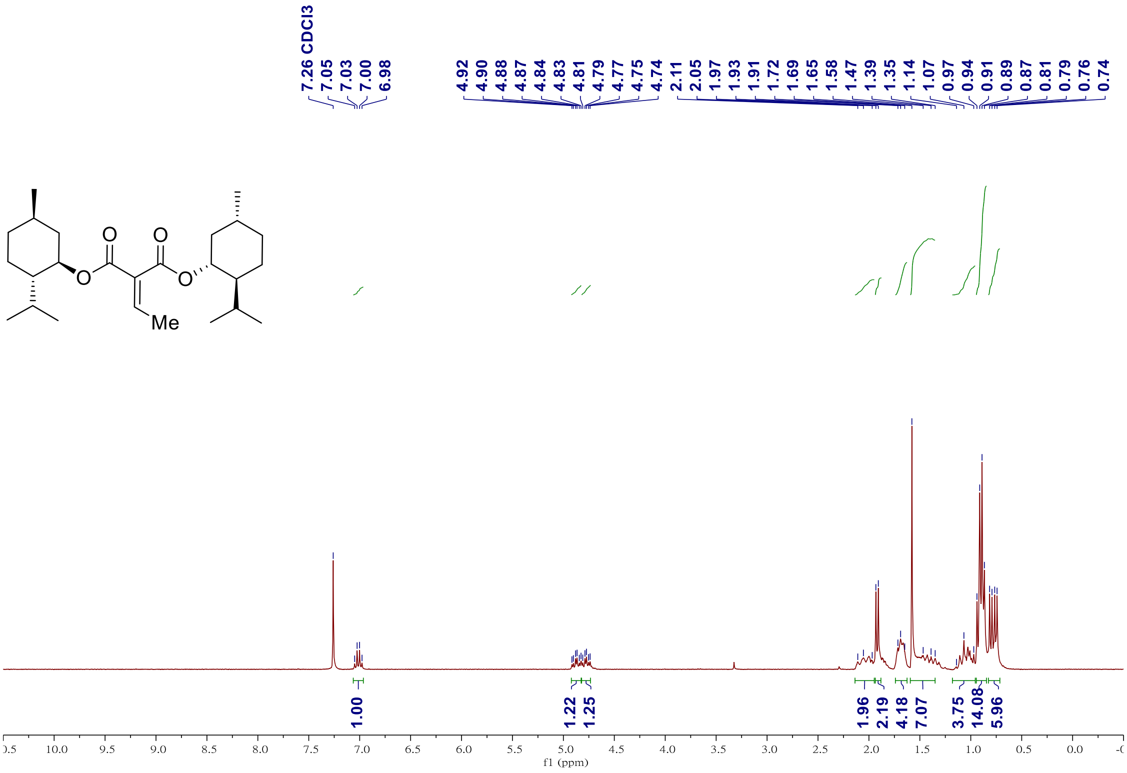

Back to the contents

¹H NMR (300 Hz, $CDCl_3$) of **2ag**, See procedure

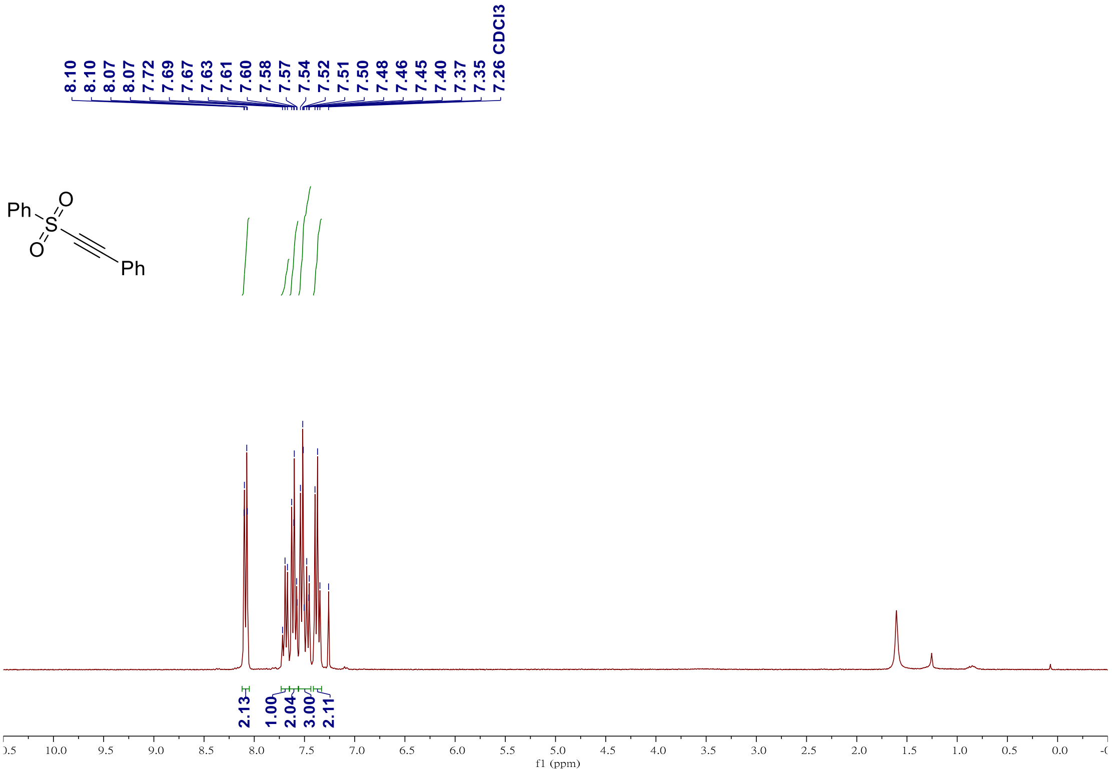

¹H NMR (300 Hz, $CDCl_3$) of **2ah**, See procedure

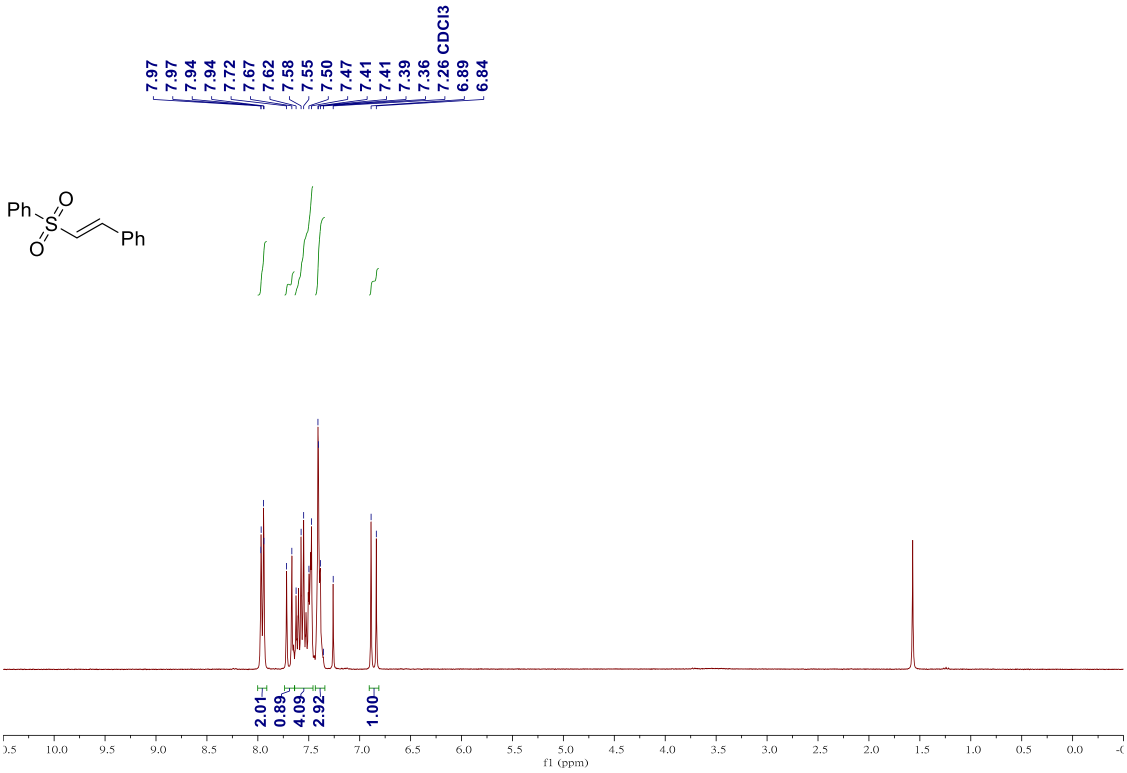

Back to the contents

^{1}H NMR (300 Hz, $CDCl_3$) of **2ai**, See procedure

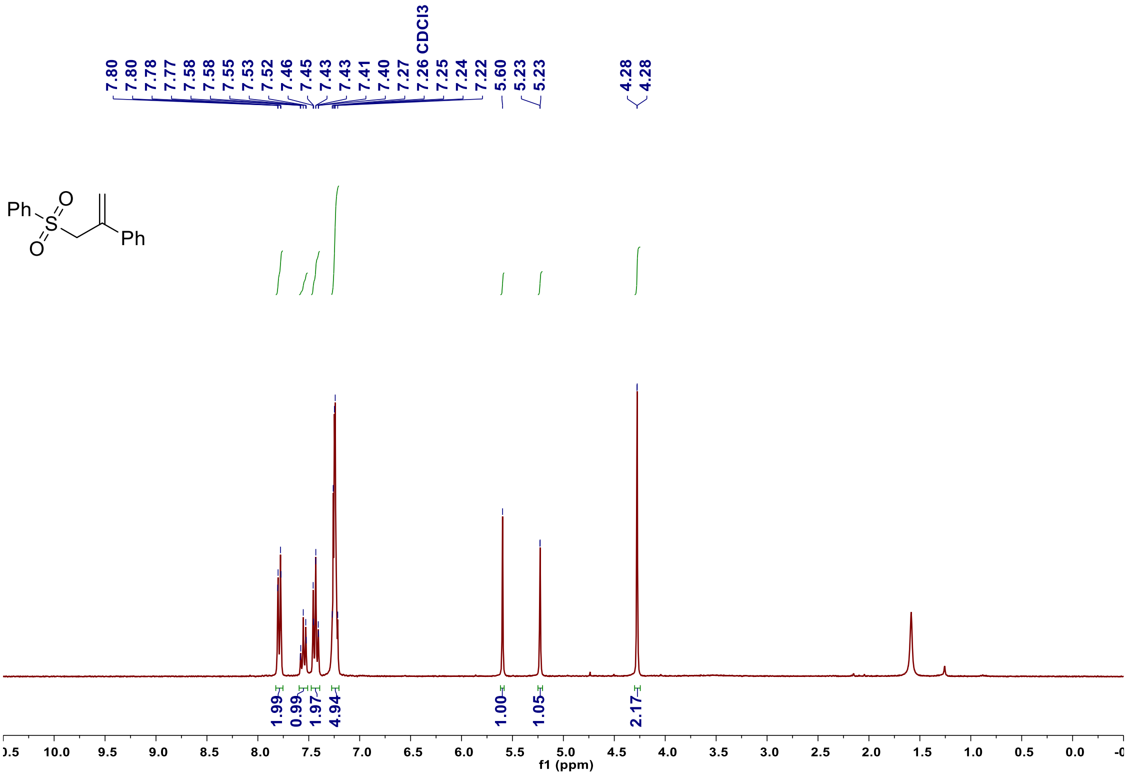

^{1}H NMR (300 Hz, $CDCl_3$) of **2aj**, See procedure

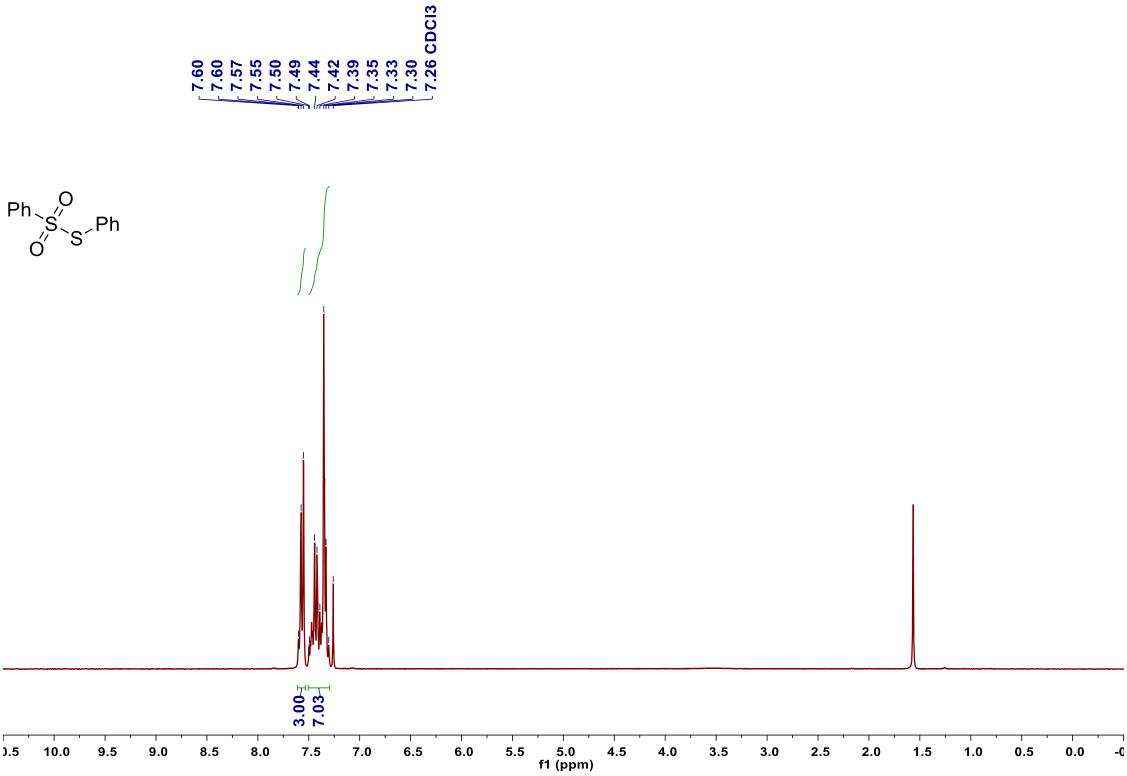

Back to the contents

^{1}**H NMR** (300 Hz, $CDCl_3$) of **2ak**, See procedure

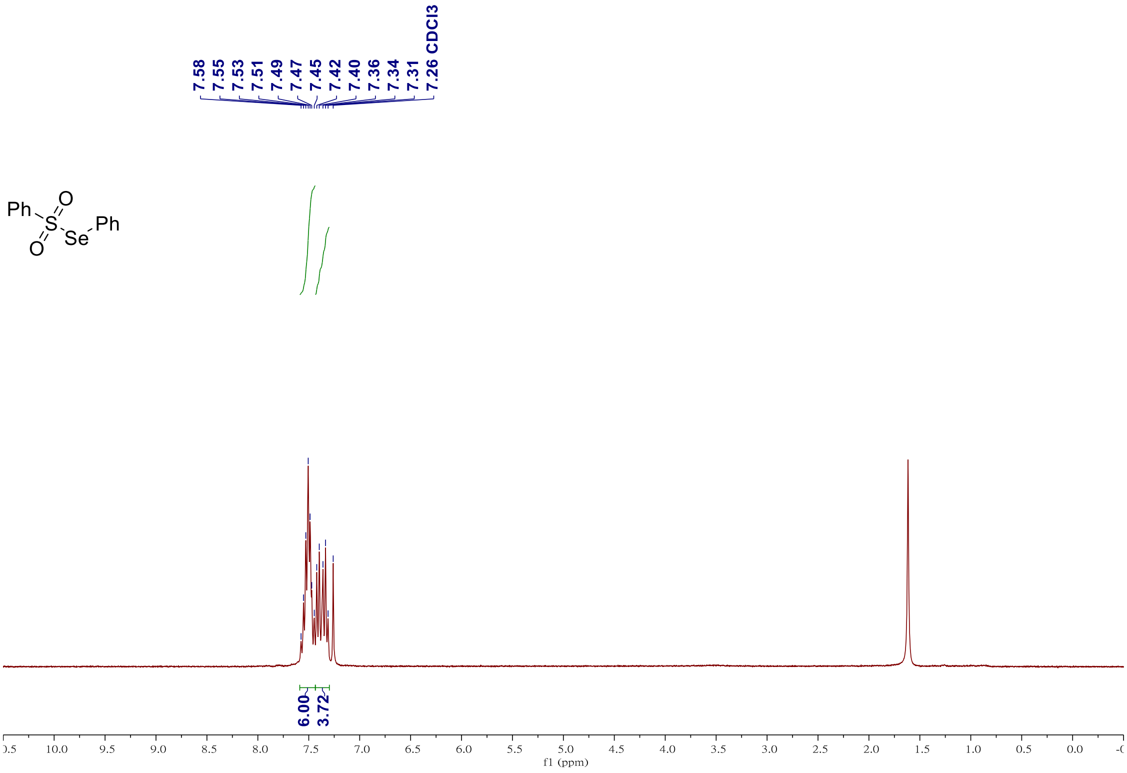

^{1}**H NMR** (300 Hz, $CDCl_3$) of **2al**, See procedure

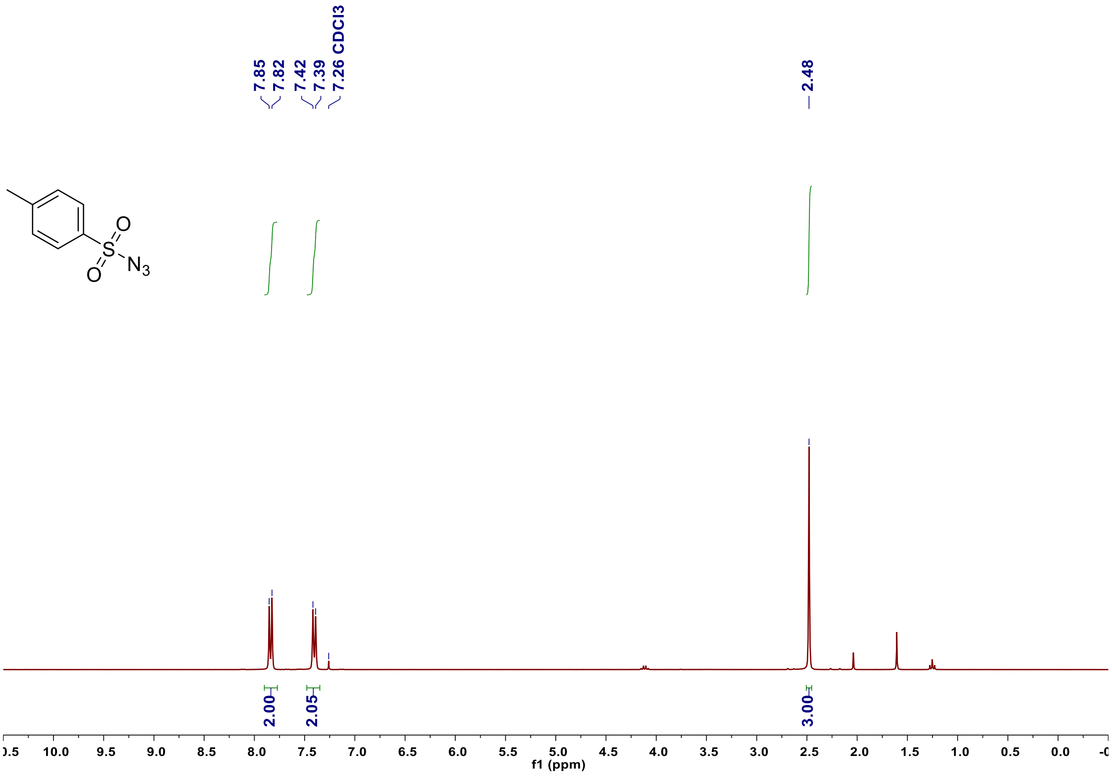

[Back to the contents](#)

^{1}H NMR (500 MHz, $CDCl_3$) of **3a**, *See procedure*

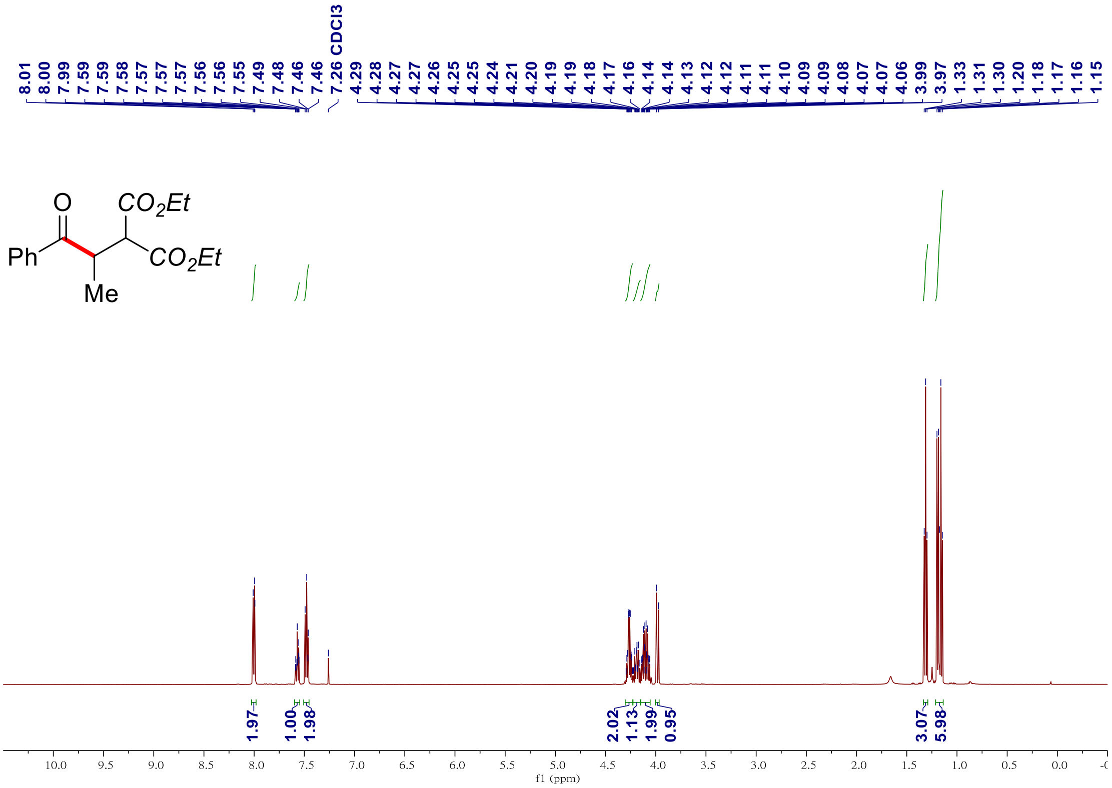

^{13}C{^{1}H} NMR (101 MHz, $CDCl_3$) of **3a**

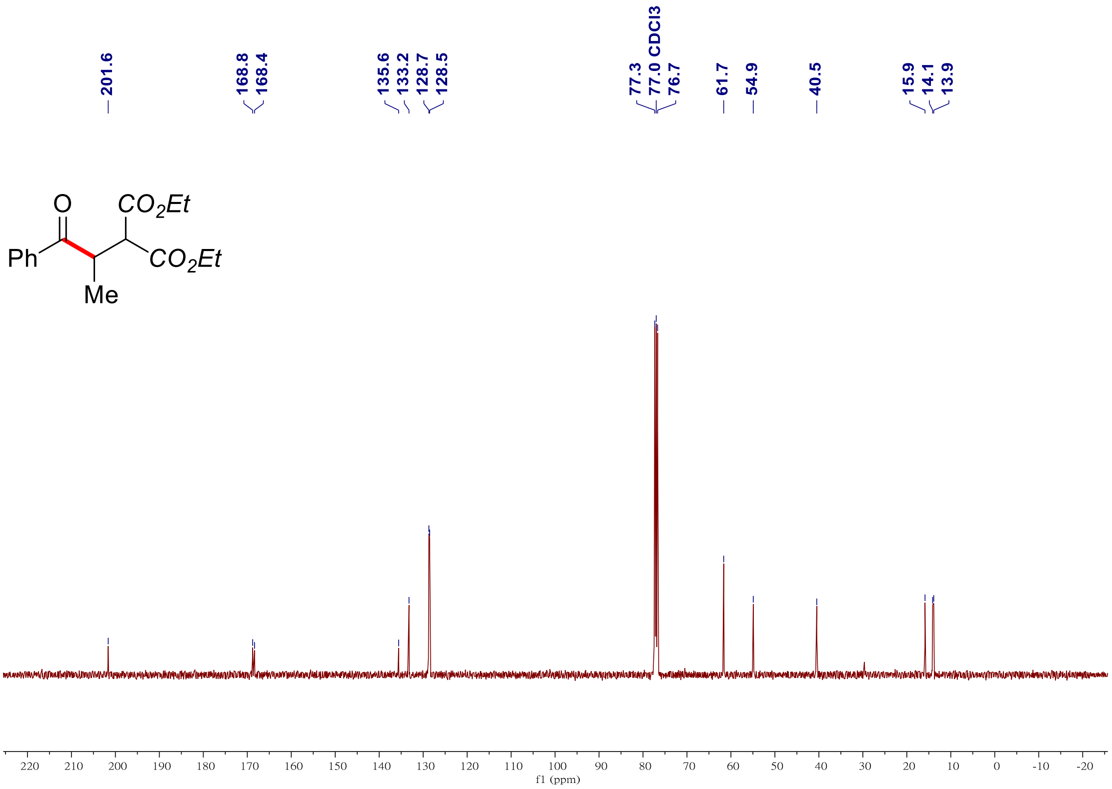

[Back to the contents](#)

^{1}H NMR (300 MHz, $CDCl_3$) of **3b**, See procedure

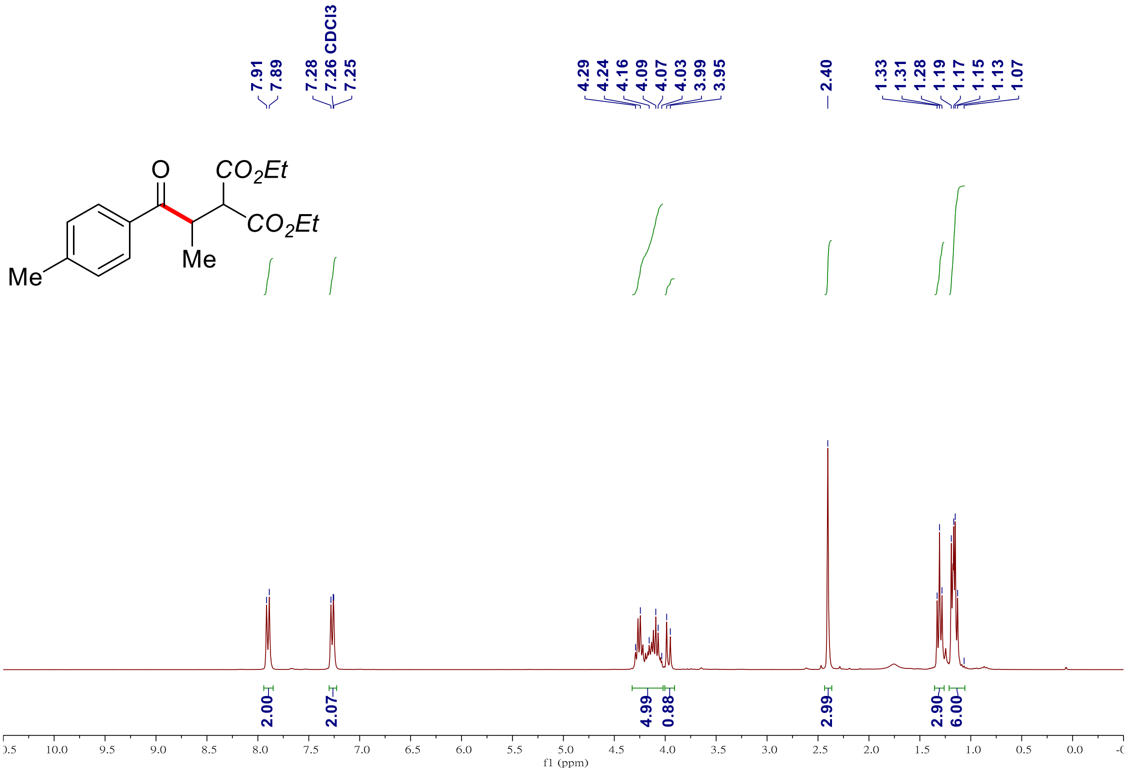

^{13}C{^{1}H} NMR (101 MHz, $CDCl_3$) of **3b**

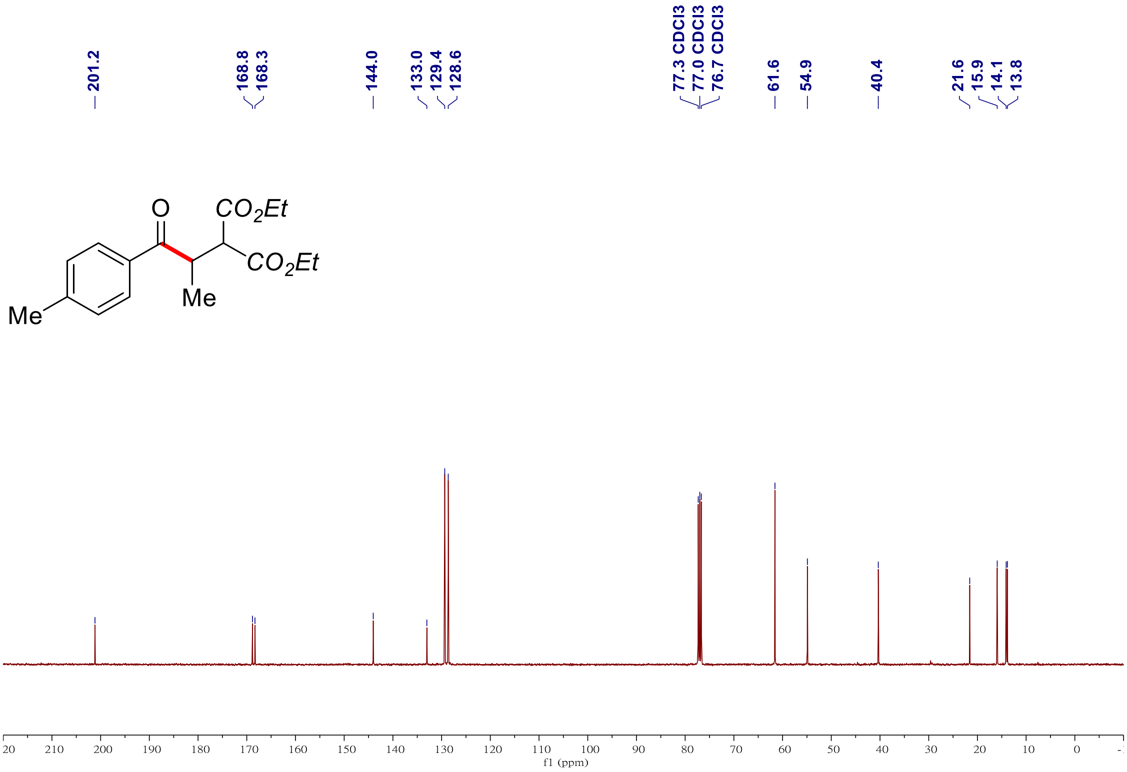

Back to the contents

^{1}H NMR (400 MHz, $CDCl_3$) of **3c**, See procedure

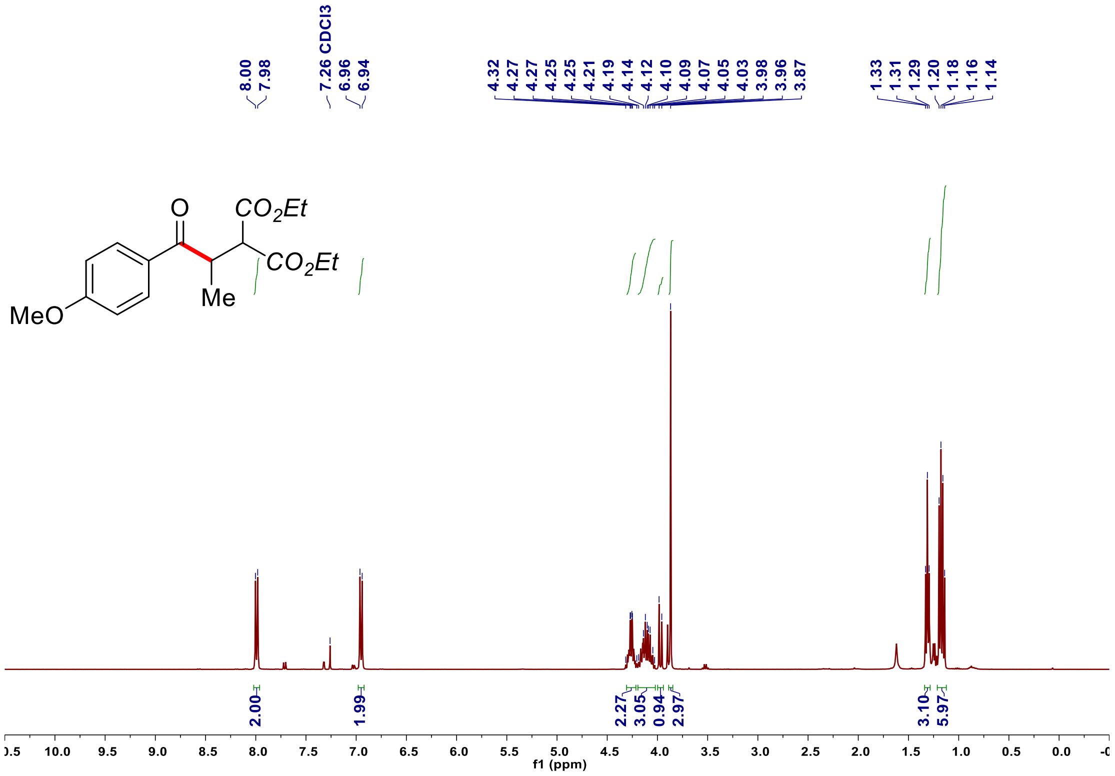

^{13}C{^{1}H} NMR (101 MHz, $CDCl_3$) of **3c**

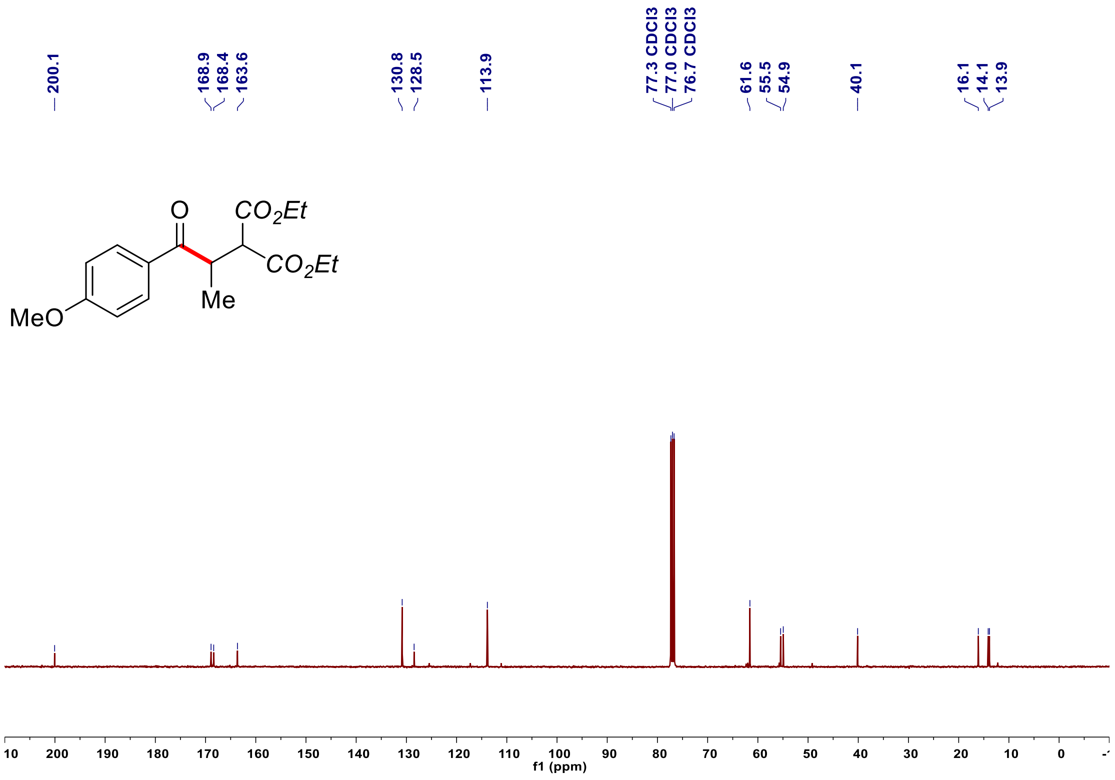

[Back to the contents](#)

^{1}H NMR (300 MHz, $CDCl_3$) of **3d**, See procedure

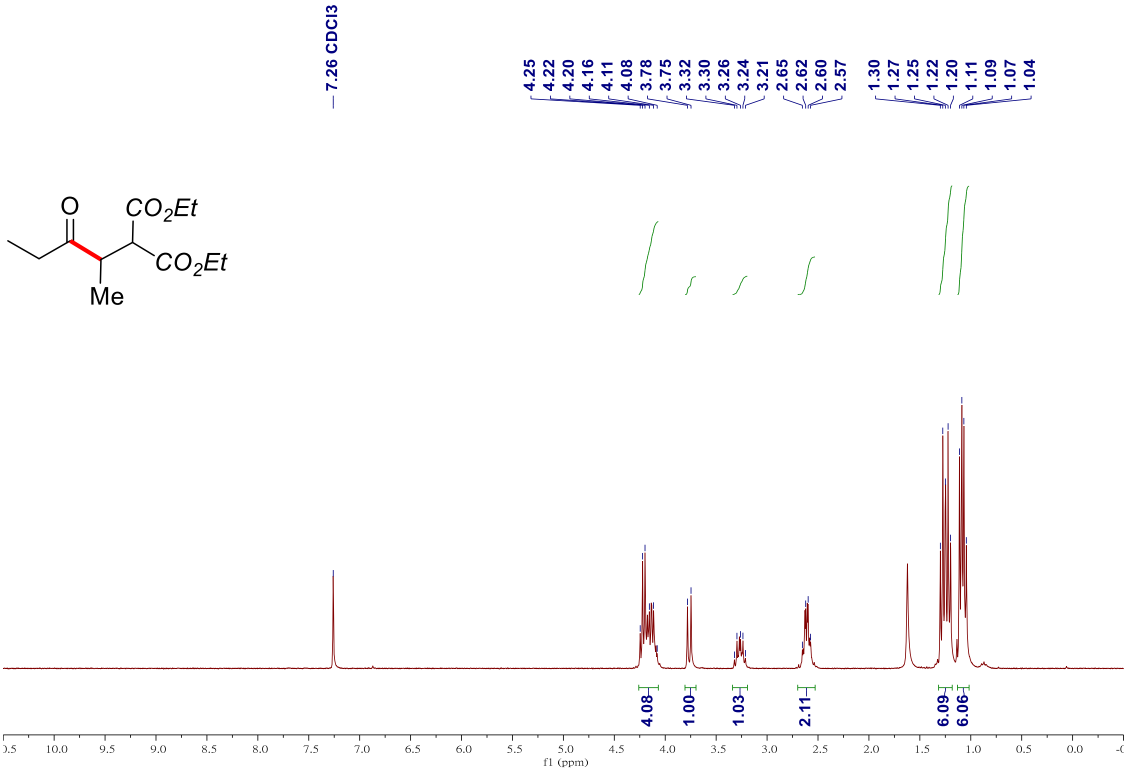

^{13}C{^{1}H} NMR (101 MHz, $CDCl_3$) of **3d**

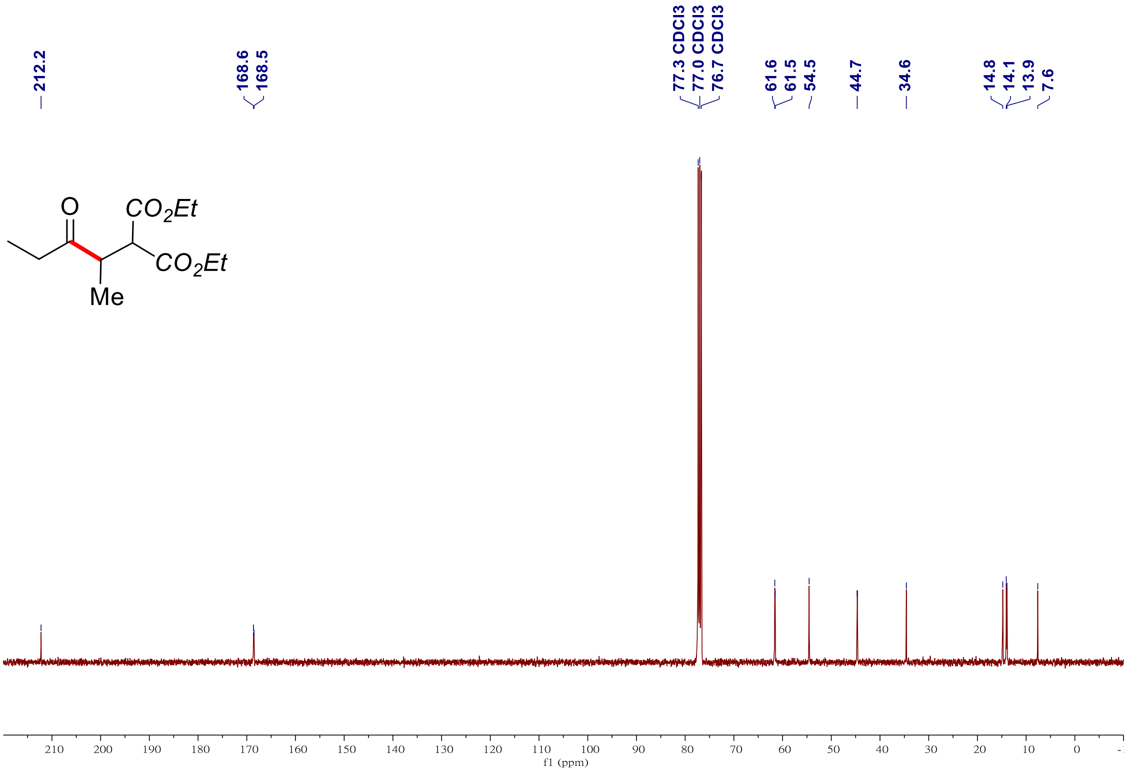

Back to the contents

¹H NMR (400 MHz, $CDCl_3$) of **3e**, See procedure

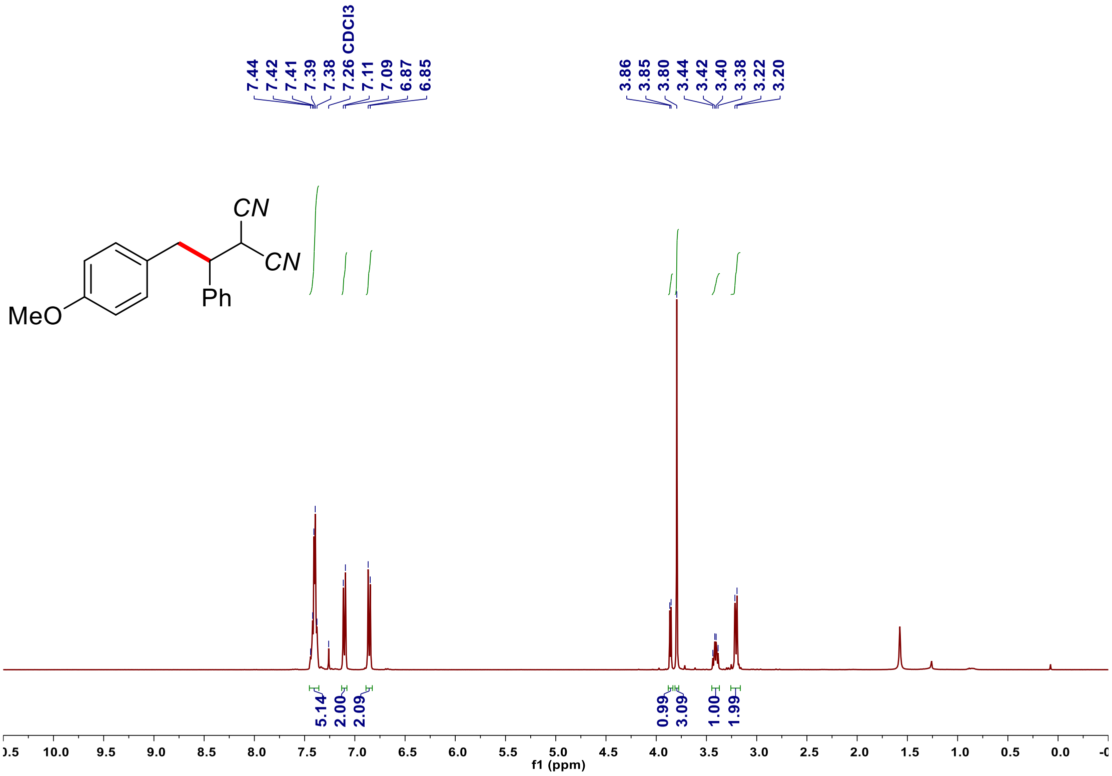

¹³C{¹H} NMR (101 MHz, $CDCl_3$) of **3e**

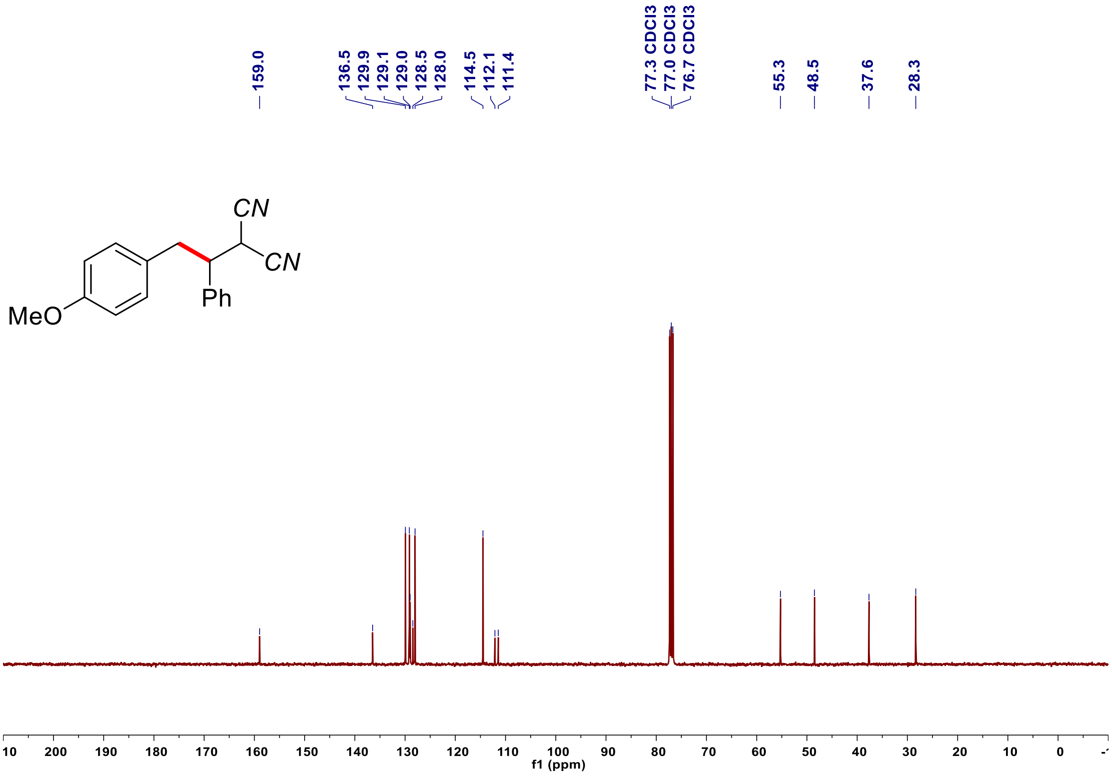

[Back to the contents](#)

^{1}H NMR (300 MHz, $CDCl_3$) of **3f**, See procedure

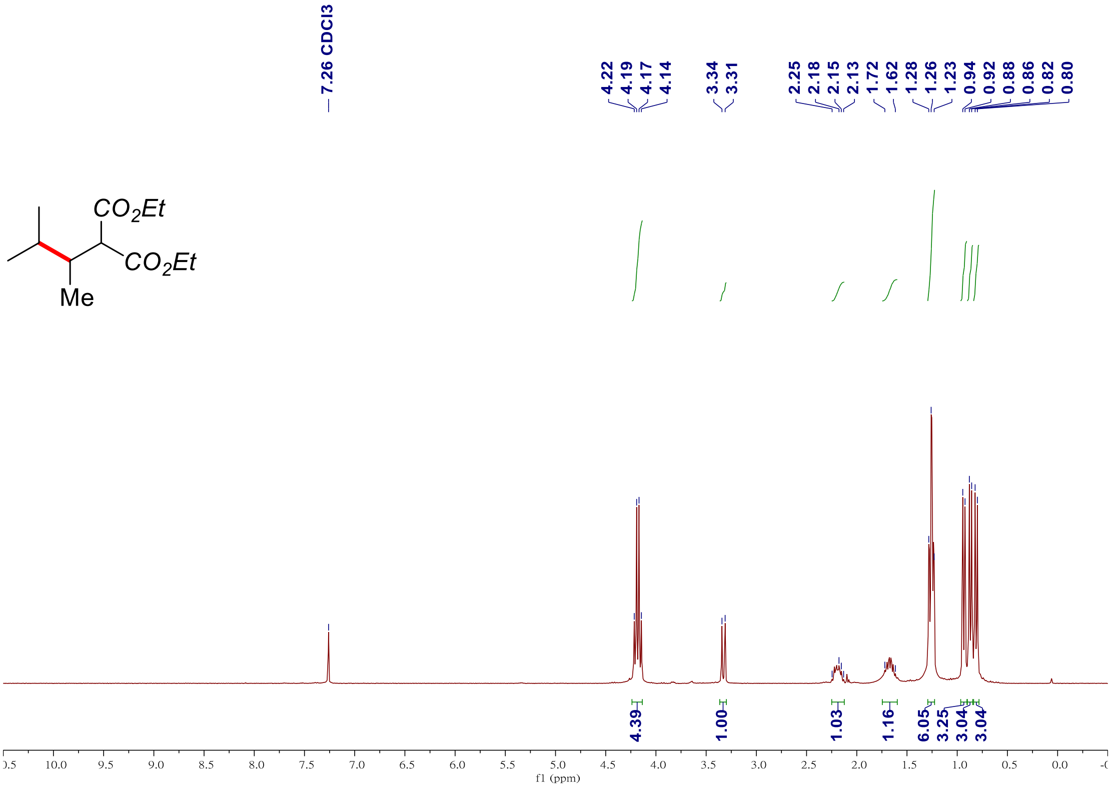

^{1}H NMR (400 MHz, $CDCl_3$) of **3g**, See procedure

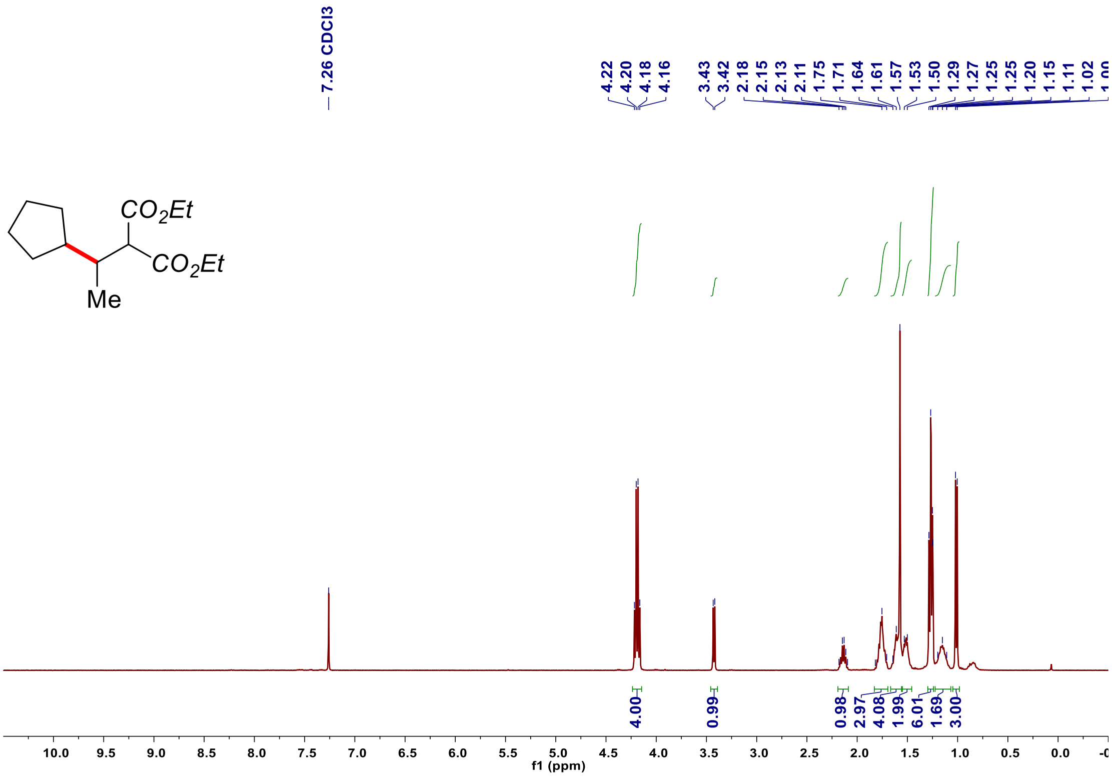

[Back to the contents](#)

$^{13}C\{^{1}H\}$ NMR (101 MHz, $CDCl_3$) of **3g**

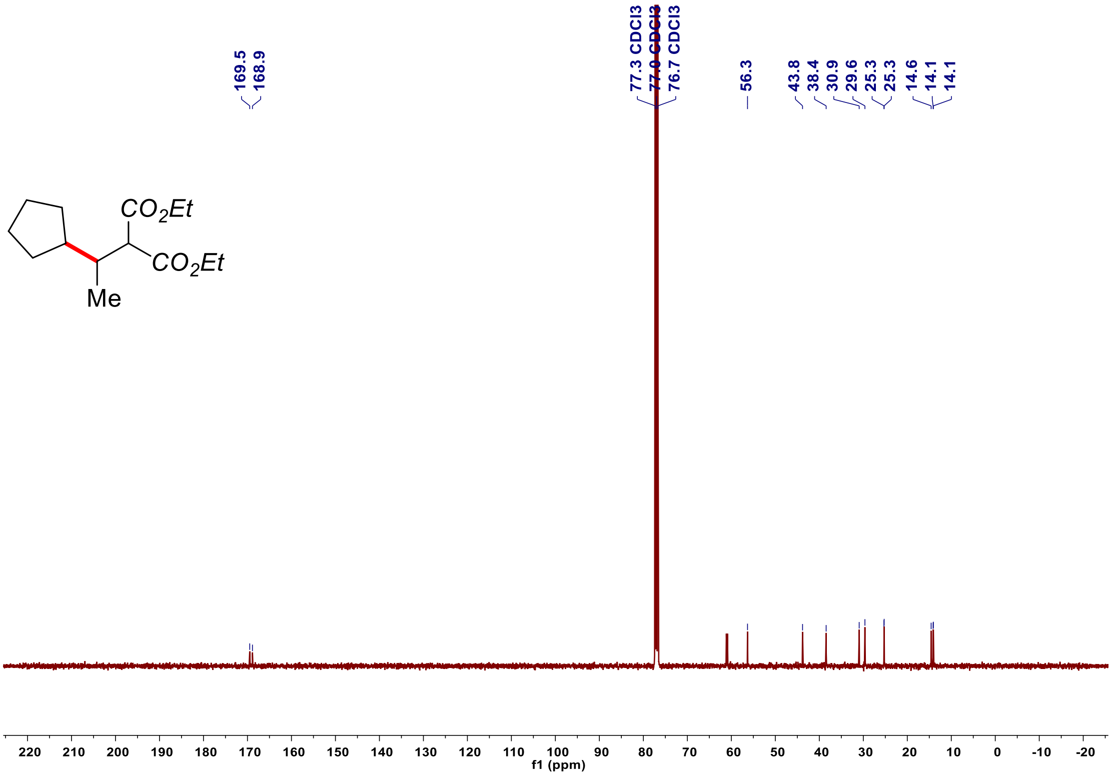

^{1}H NMR (300 MHz, $CDCl_3$) of **3h**, See procedure

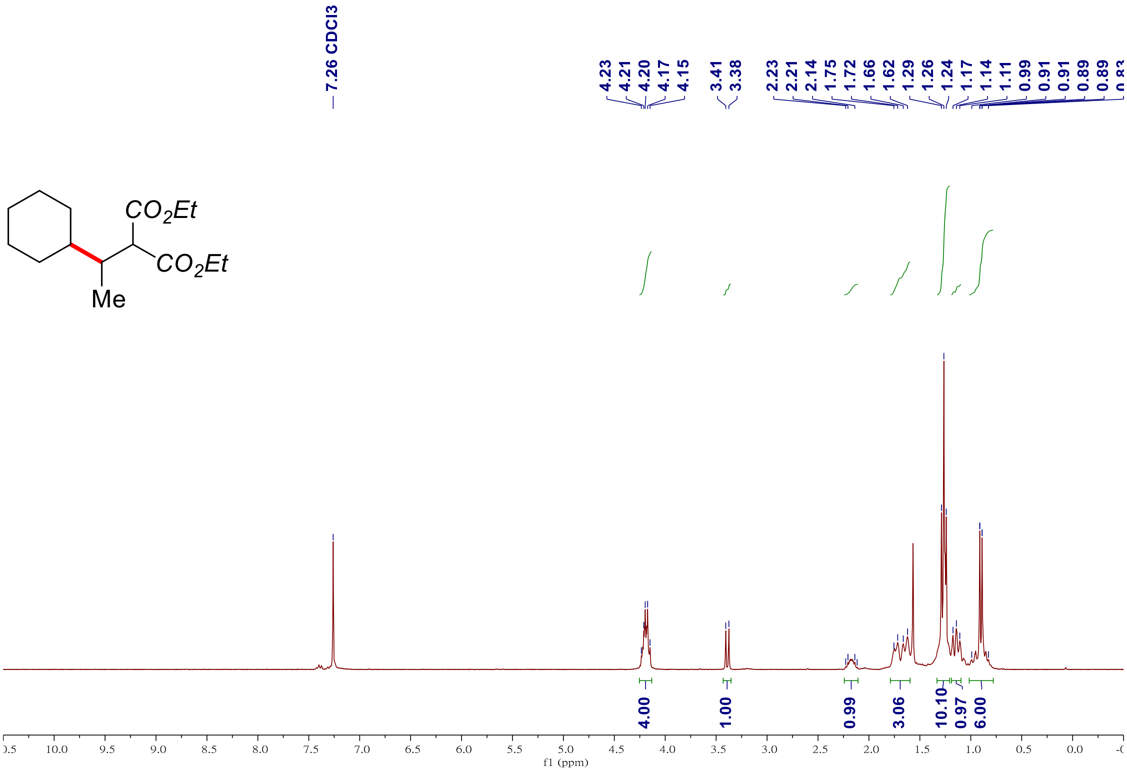

Back to the contents

$^{13}C\{^{1}H\}$ NMR (101 MHz, $CDCl_3$) of **3h**

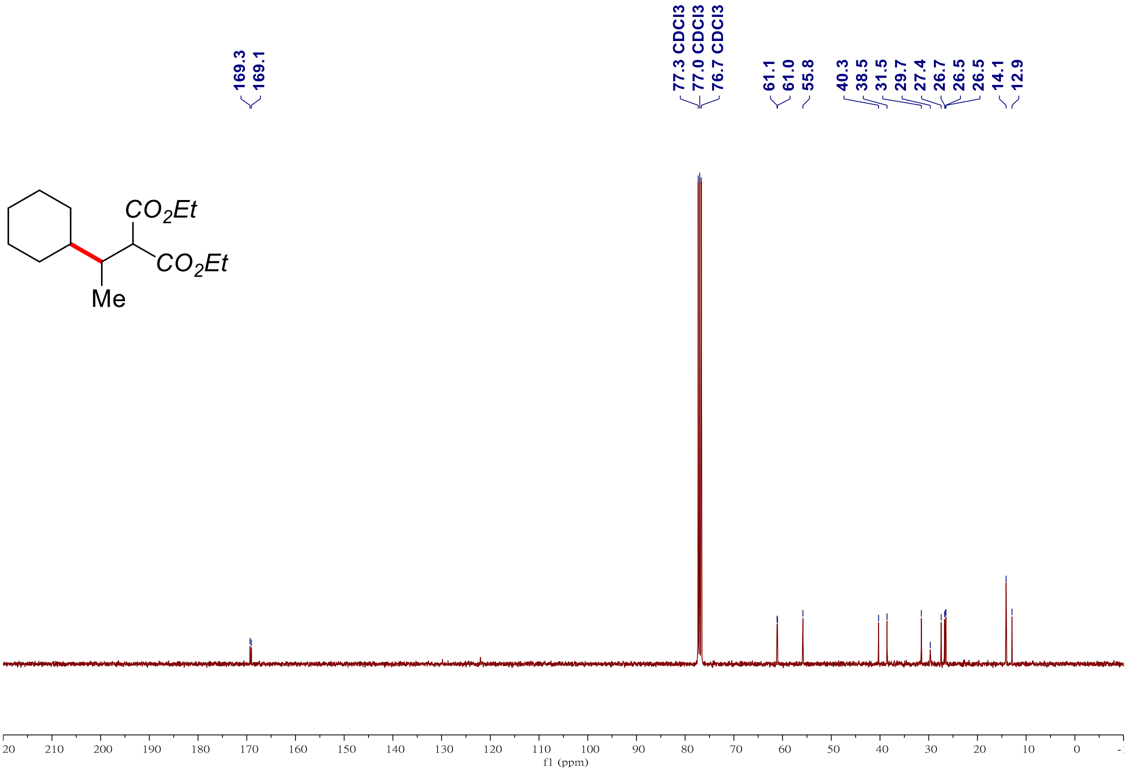

^{1}H NMR (400 MHz, $CDCl_3$) of **3i**, See procedure

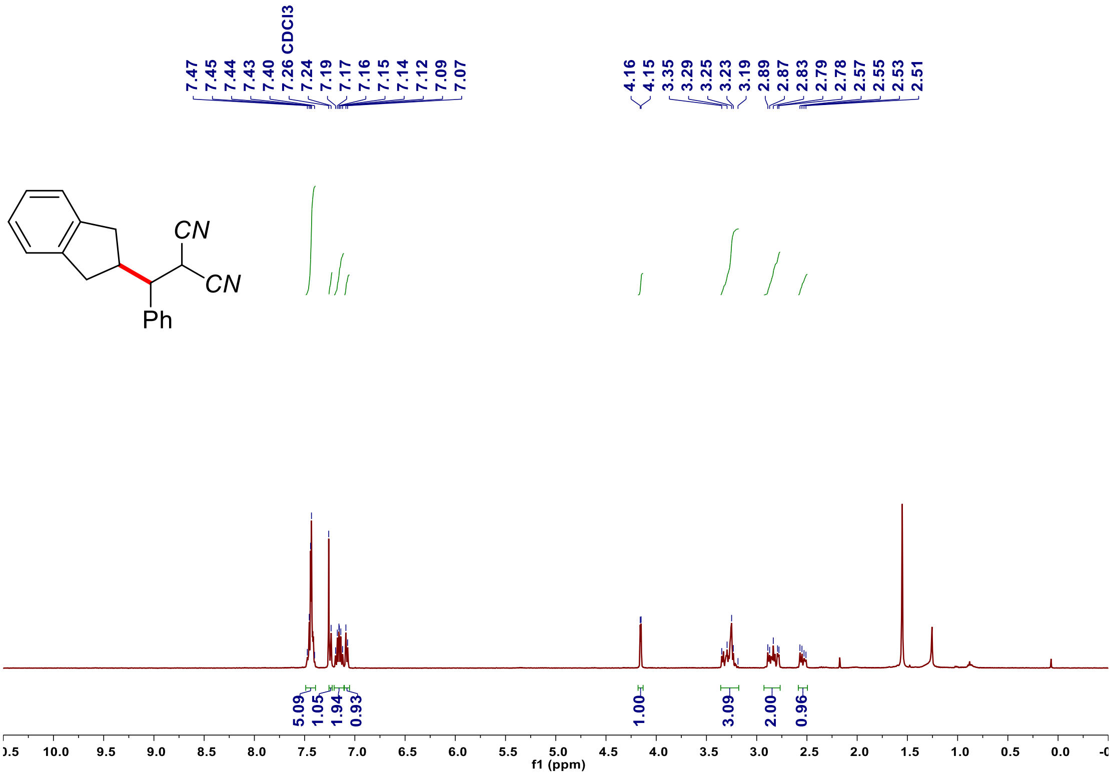

Back to the contents

$^{13}C\{^{1}H\}$ NMR (101 MHz, $CDCl_3$) of **3i**

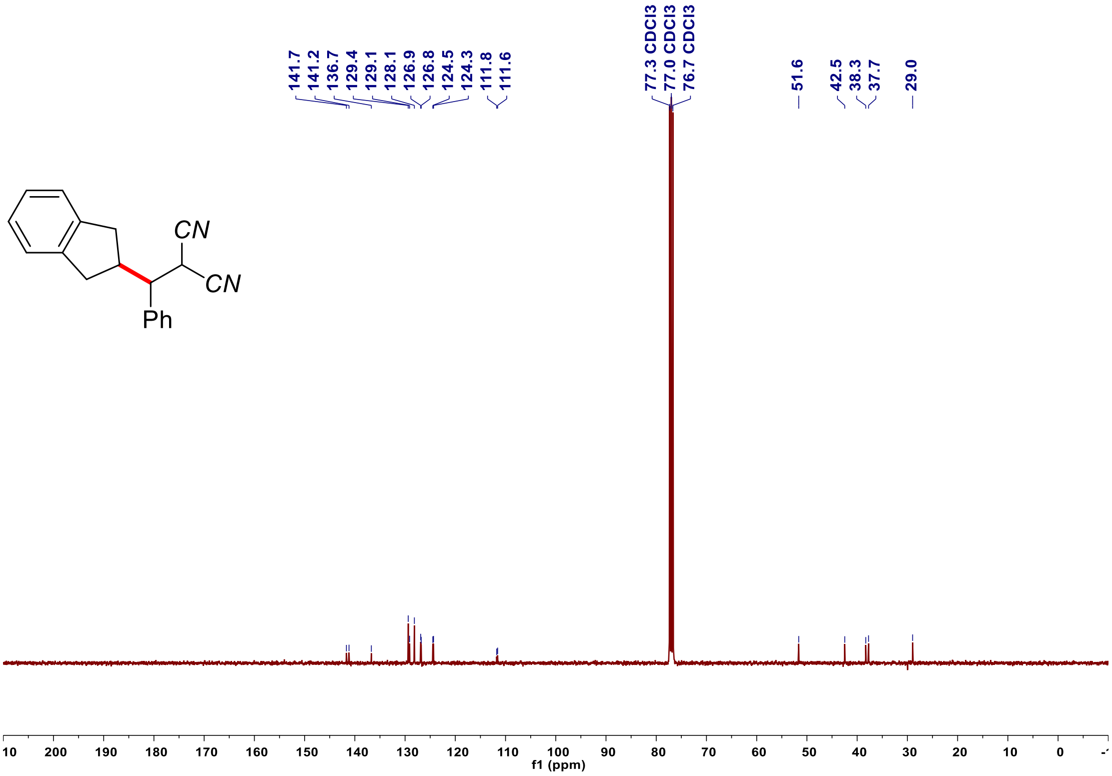

^{1}H NMR (400 MHz, $CDCl_3$) of **3j**, See procedure

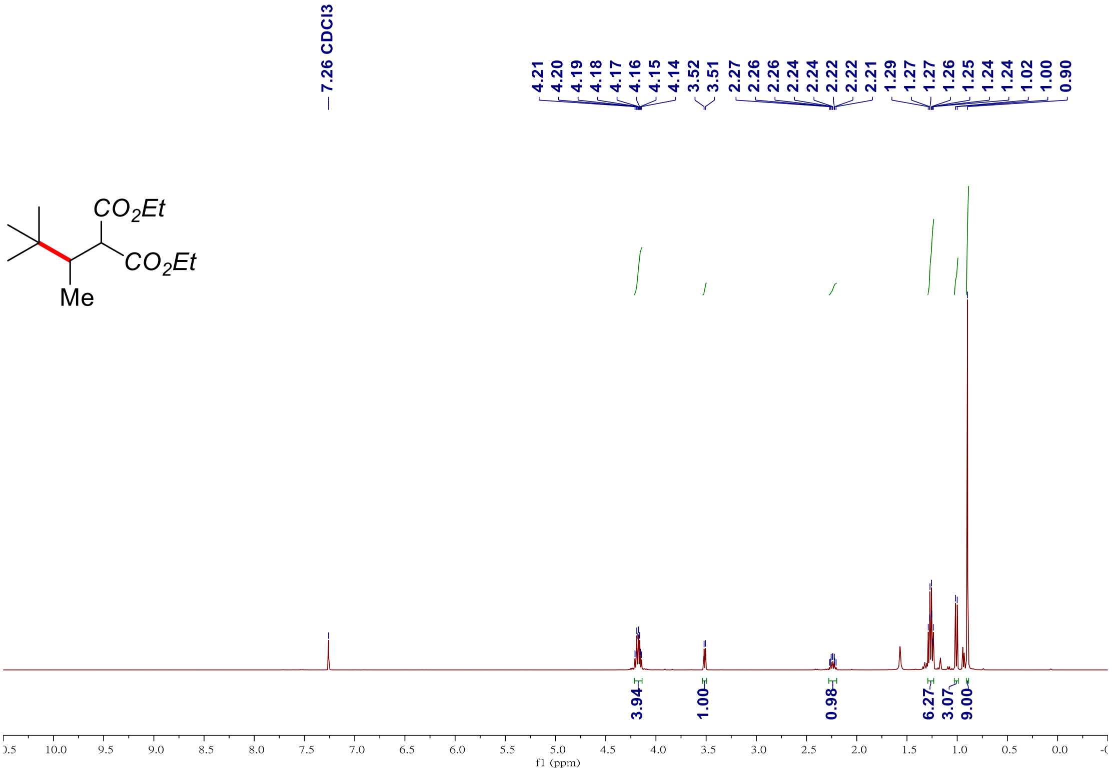

Back to the contents

$^{13}C\{^1H\}$ NMR (101 MHz, $CDCl_3$) of **3j**

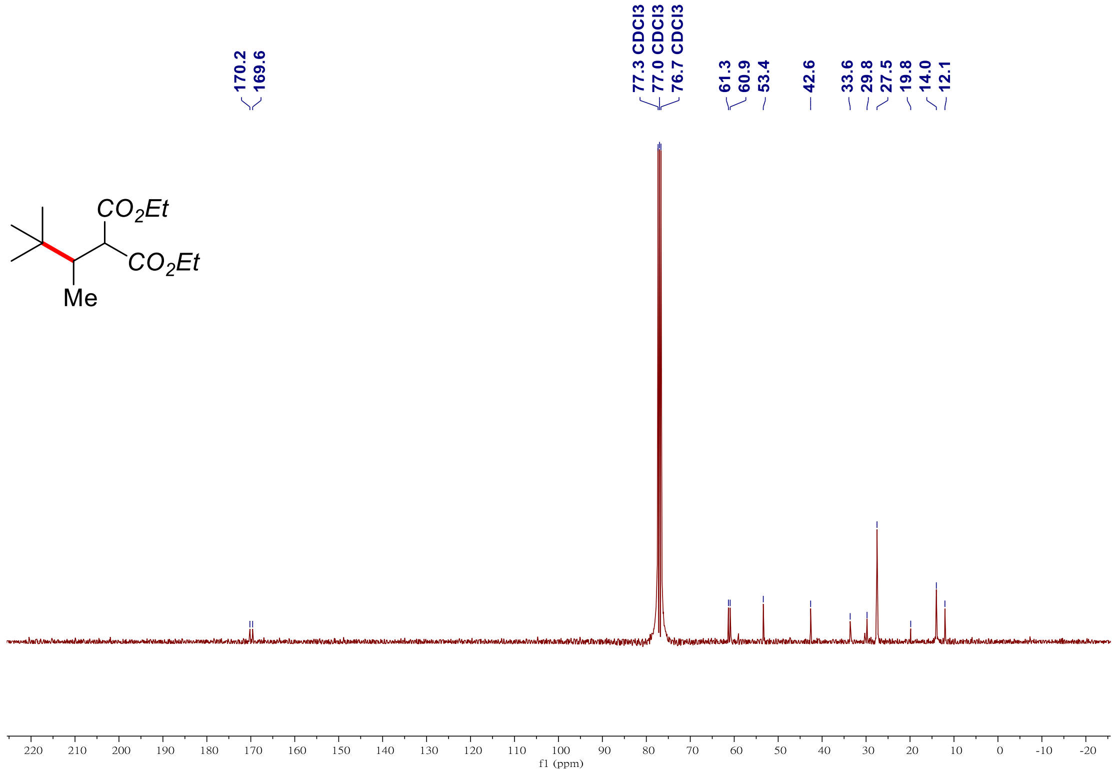

^1H NMR (300 MHz, $CDCl_3$) of **3k**, See procedure

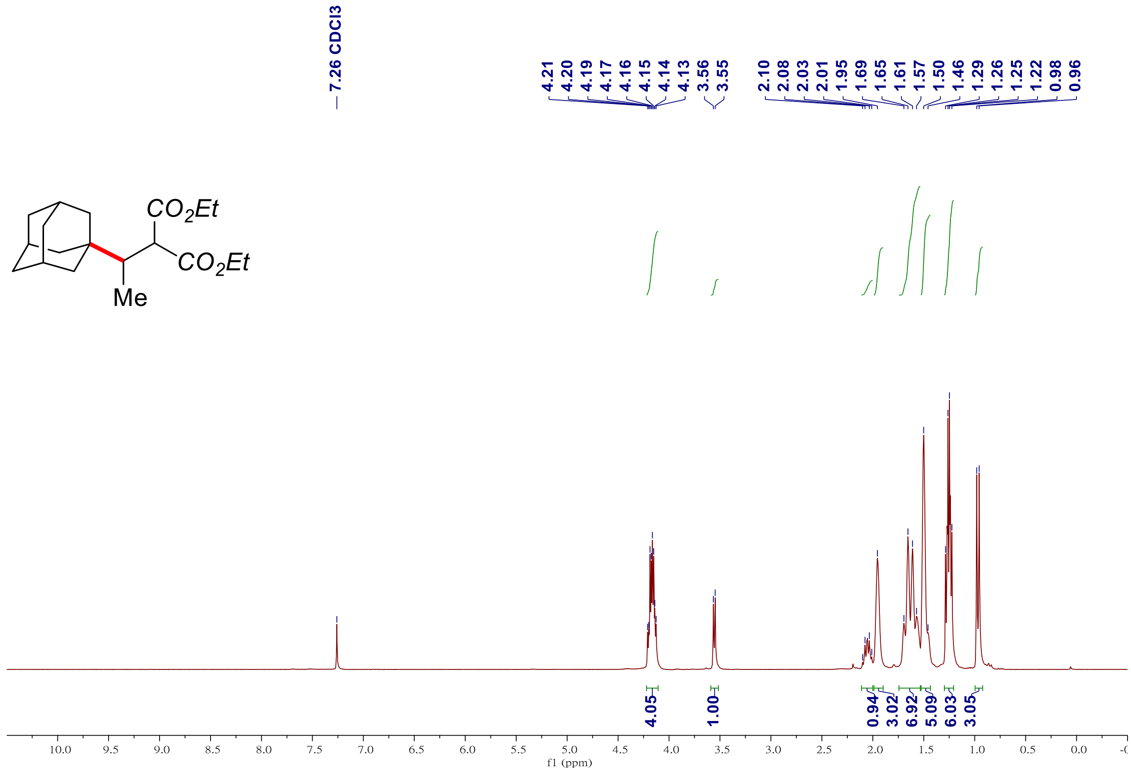

Back to the contents

$^{13}C\{^1H\}$ NMR (101 MHz, $CDCl_3$) of **3k**

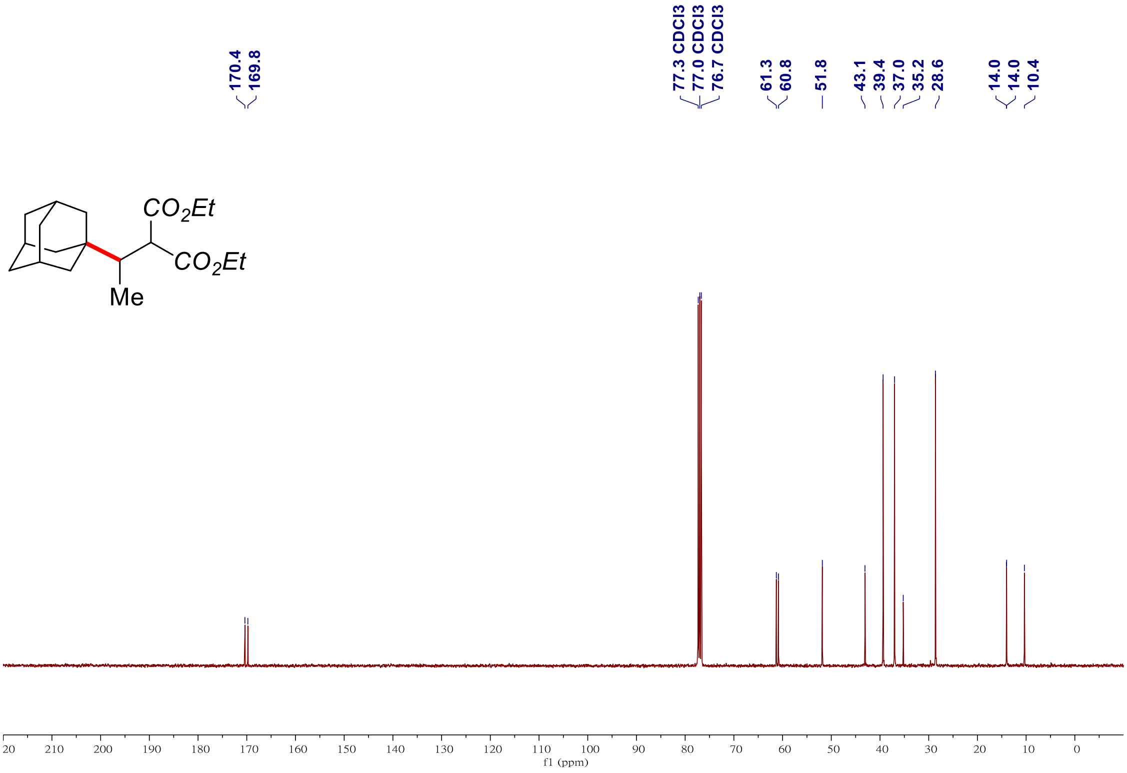

^1H NMR (400 MHz, $CDCl_3$) of **3l**, See procedure

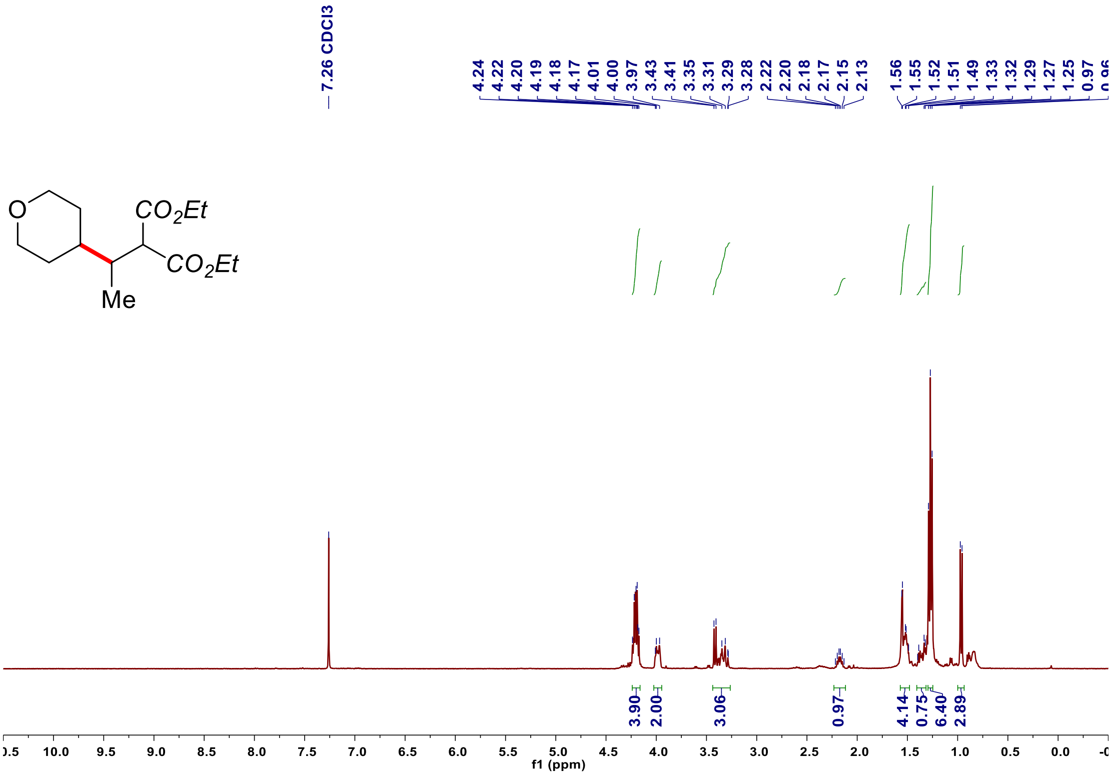

[Back to the contents](#)

$^{13}C\{^1H\}$ NMR (101 MHz, $CDCl_3$) of **3l**

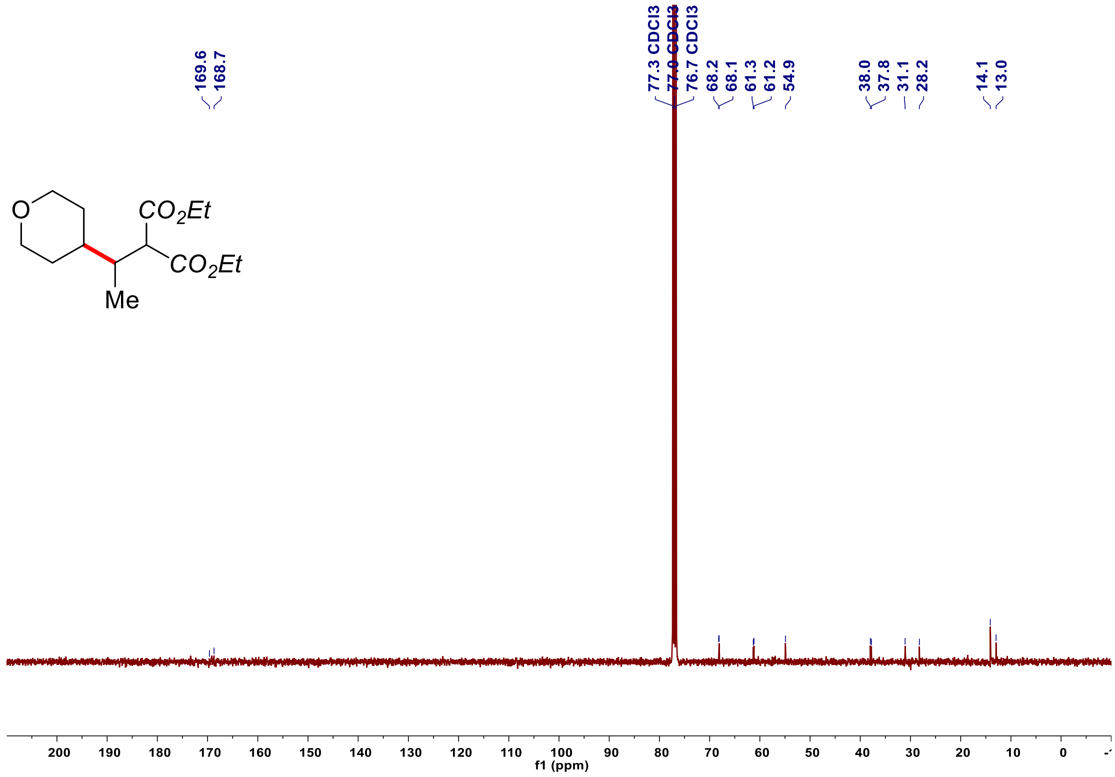

H NMR (400 MHz, $CDCl_3$) of **3m**, [See procedure](#)

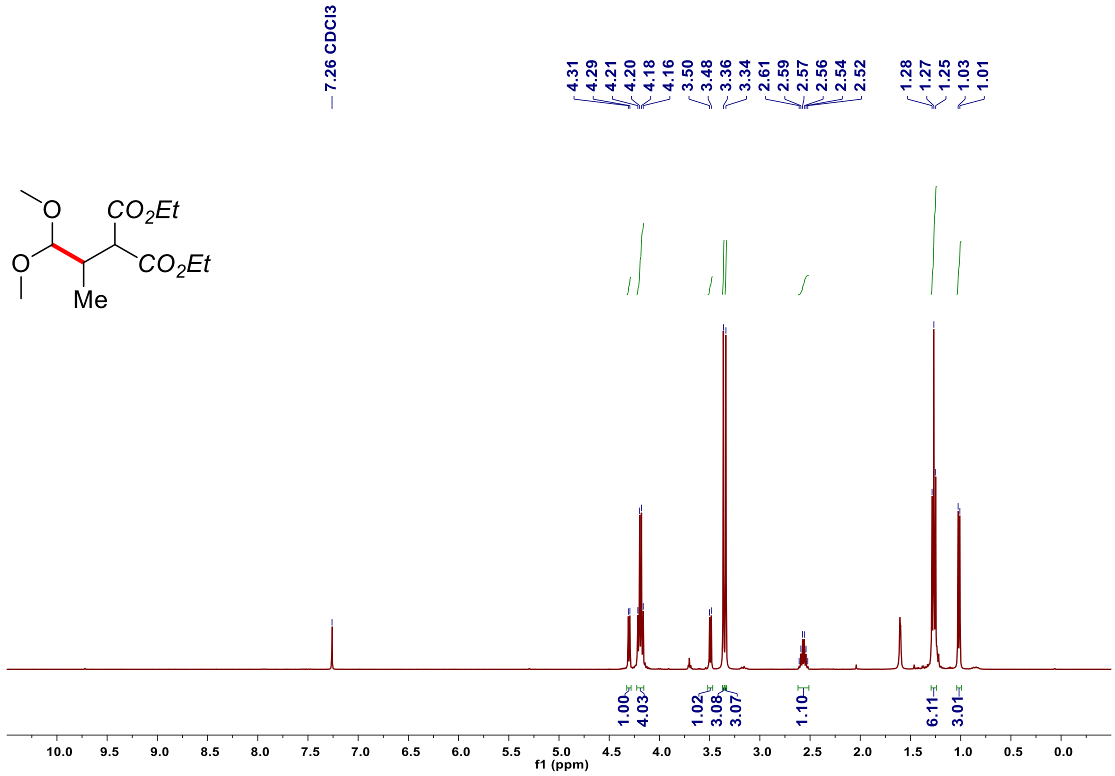

[Back to the contents](#)

$^{13}C\{^1H\}$ NMR (101 MHz, $CDCl_3$) of **3m**

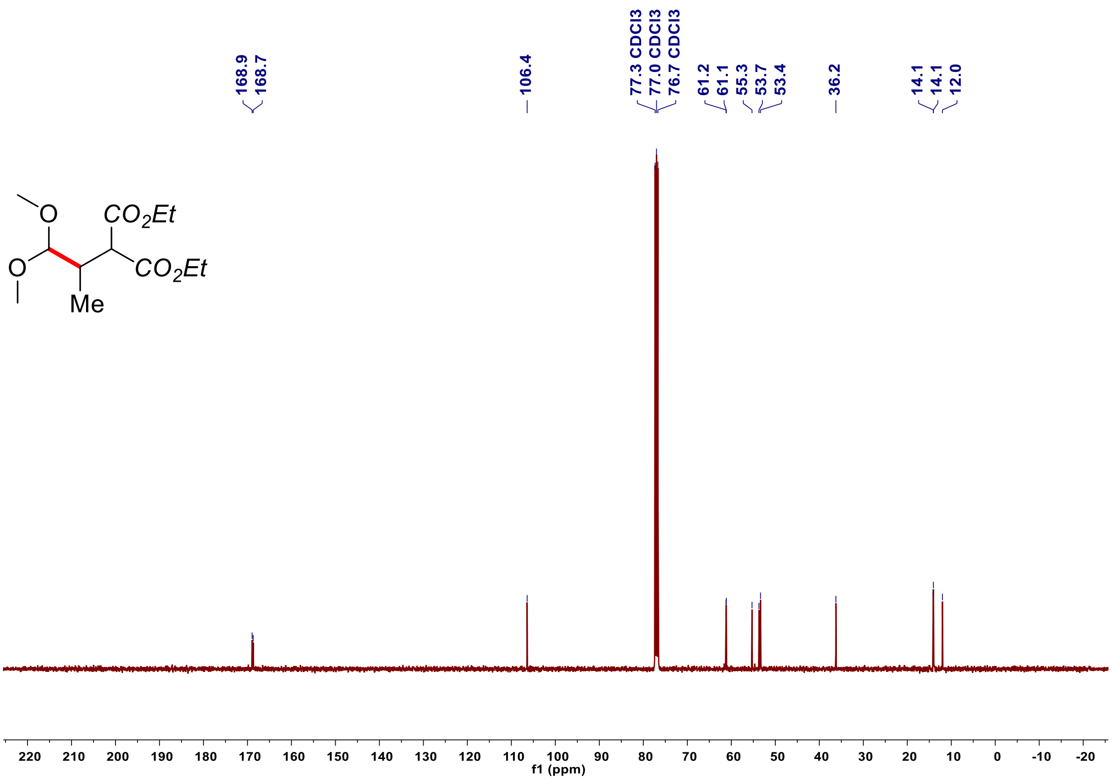

^1H NMR (300 MHz, $CDCl_3$) of **4a**, [See procedure](#)

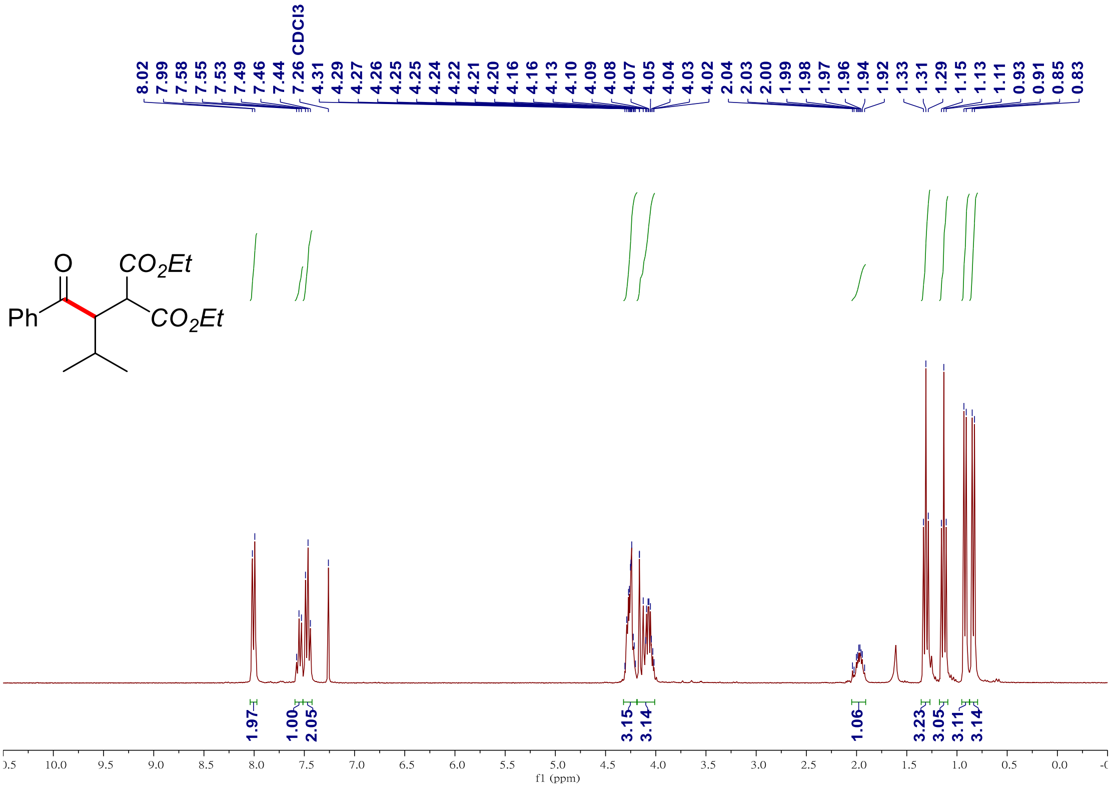

Back to the contents

$^{13}C\{^{1}H\}$ NMR (101 MHz, $CDCl_3$) of **4a**

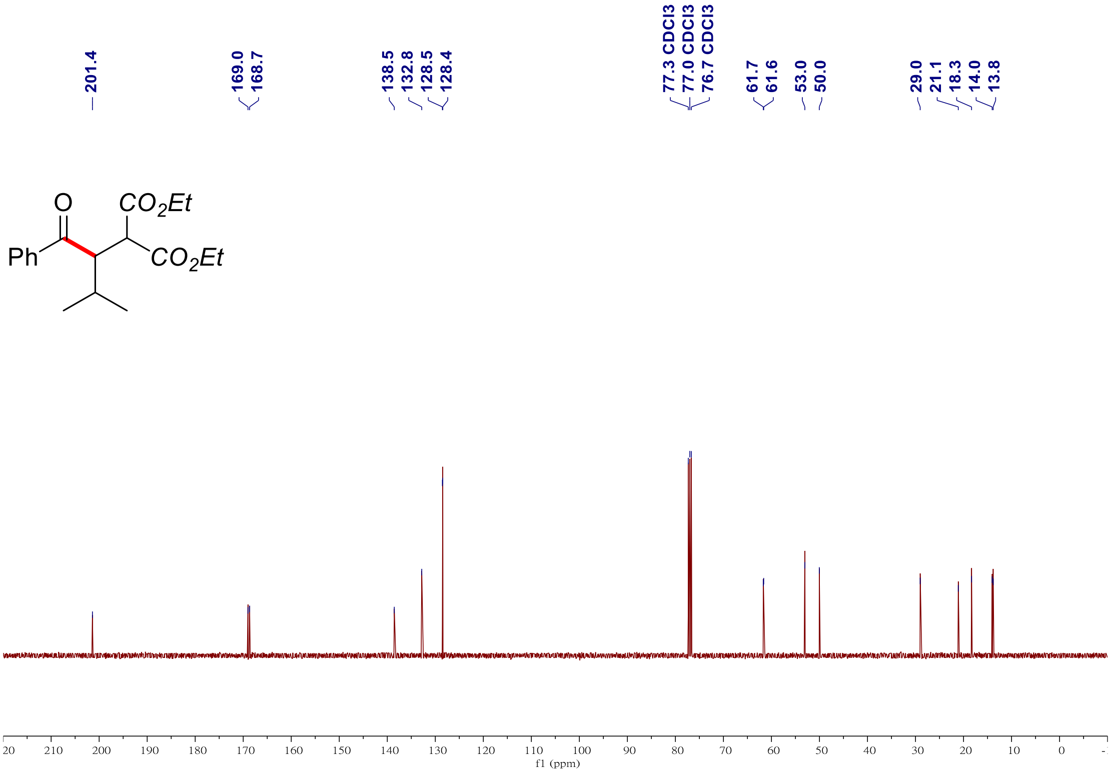

^{1}H NMR (300 MHz, $CDCl_3$) of **4b**, See procedure

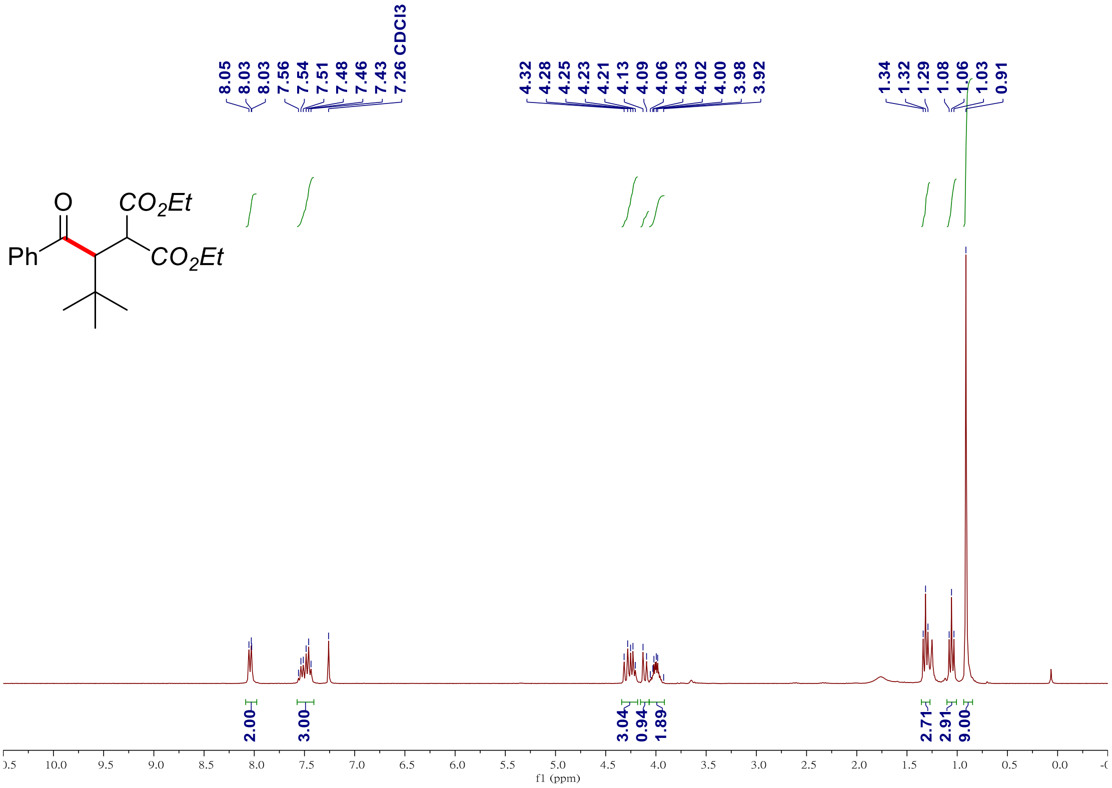

Back to the contents

$^{13}C\{^1H\}$ NMR (101 MHz, $CDCl_3$) of **4b**

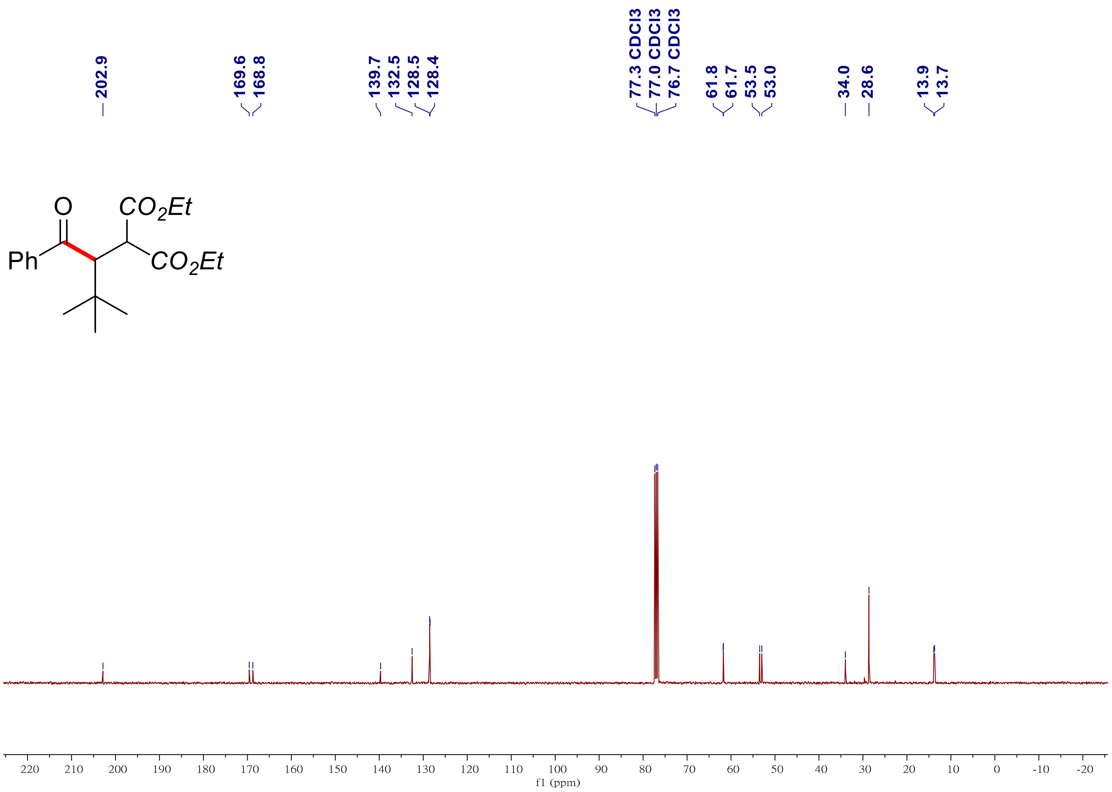

^1H NMR (300 MHz, $CDCl_3$) of **4c**, See procedure

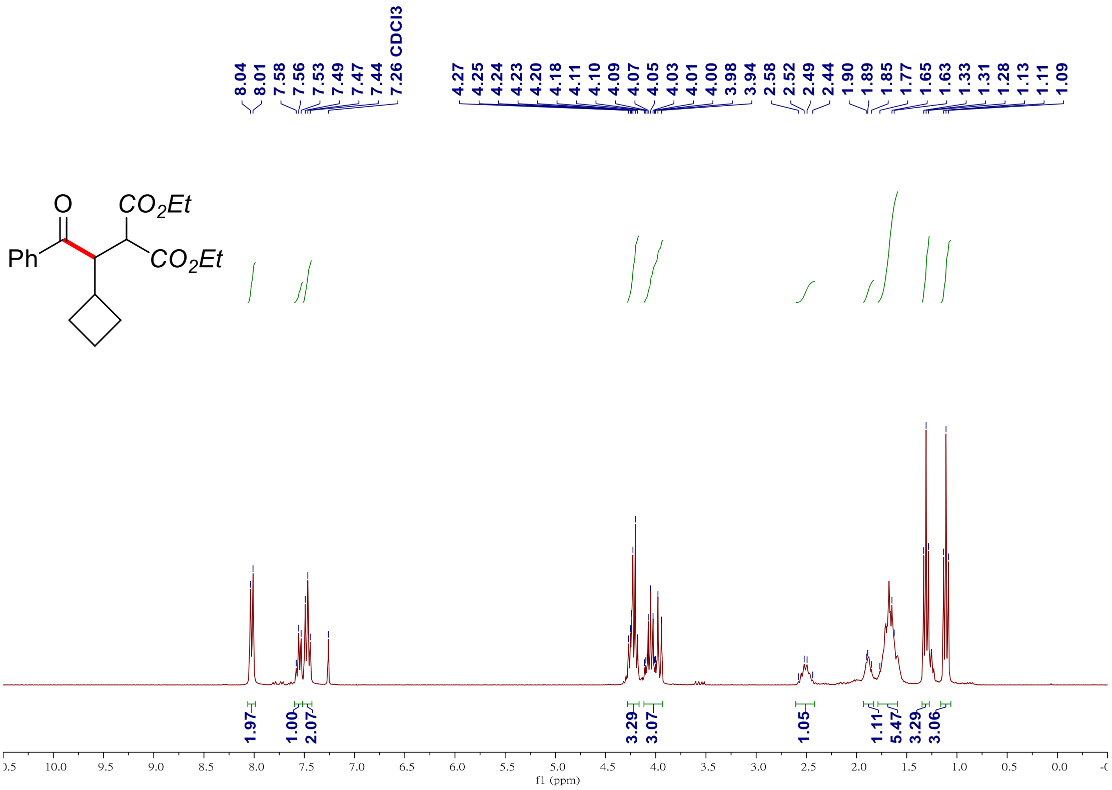

[Back to the contents](#)

$^{13}C\{^{1}H\}$ NMR (101 MHz, $CDCl_3$) of **4c**

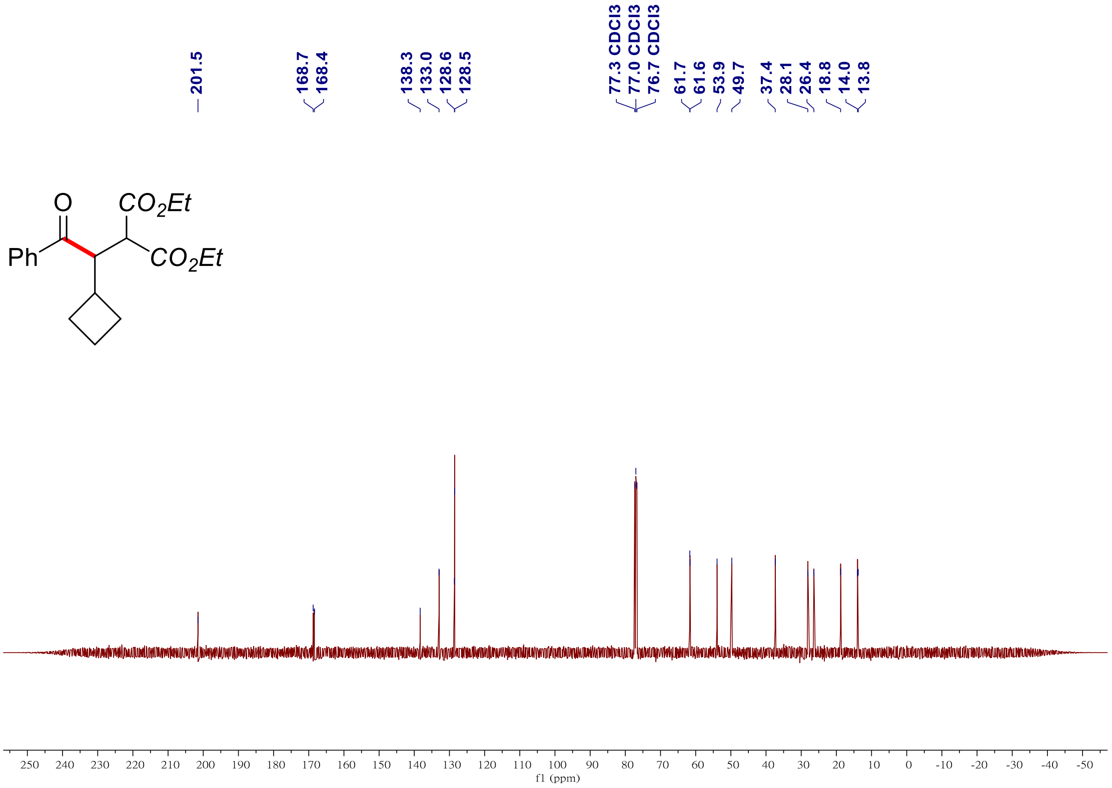

^{1}H NMR (300 MHz, $CDCl_3$) of **4d**, [See procedure](#)

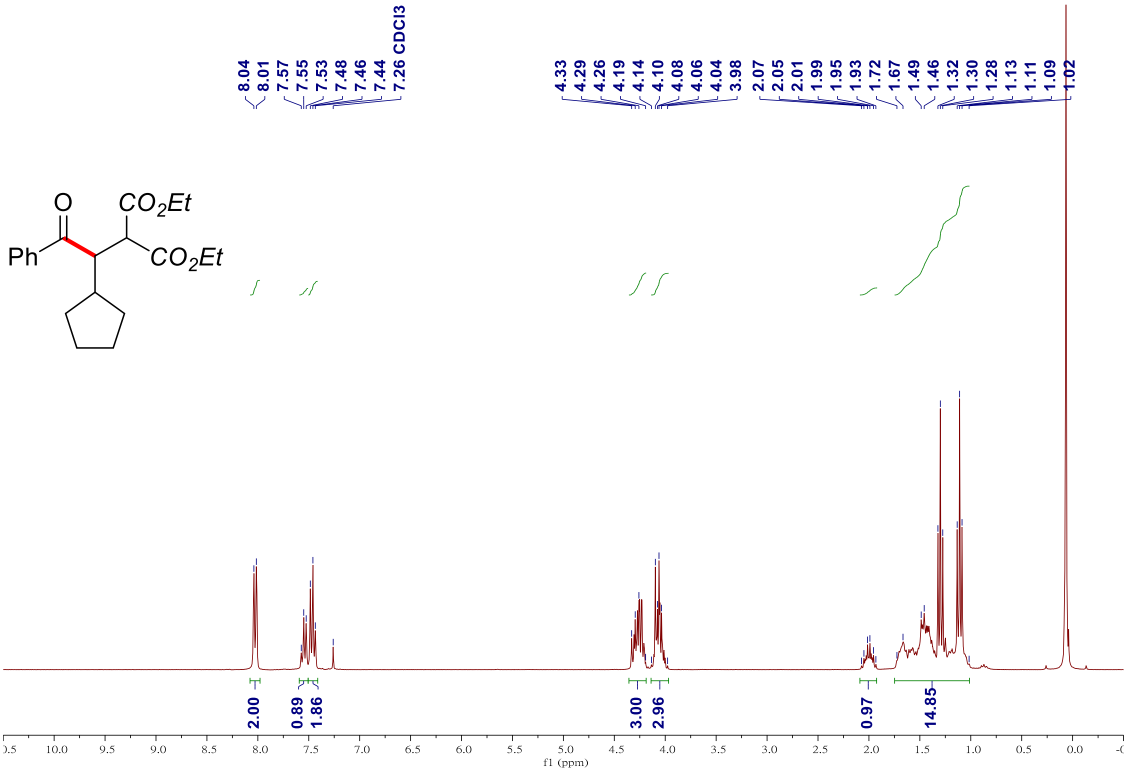

Back to the contents

$^{13}C\{^1H\}$ NMR (101 MHz, $CDCl_3$) of **4d**

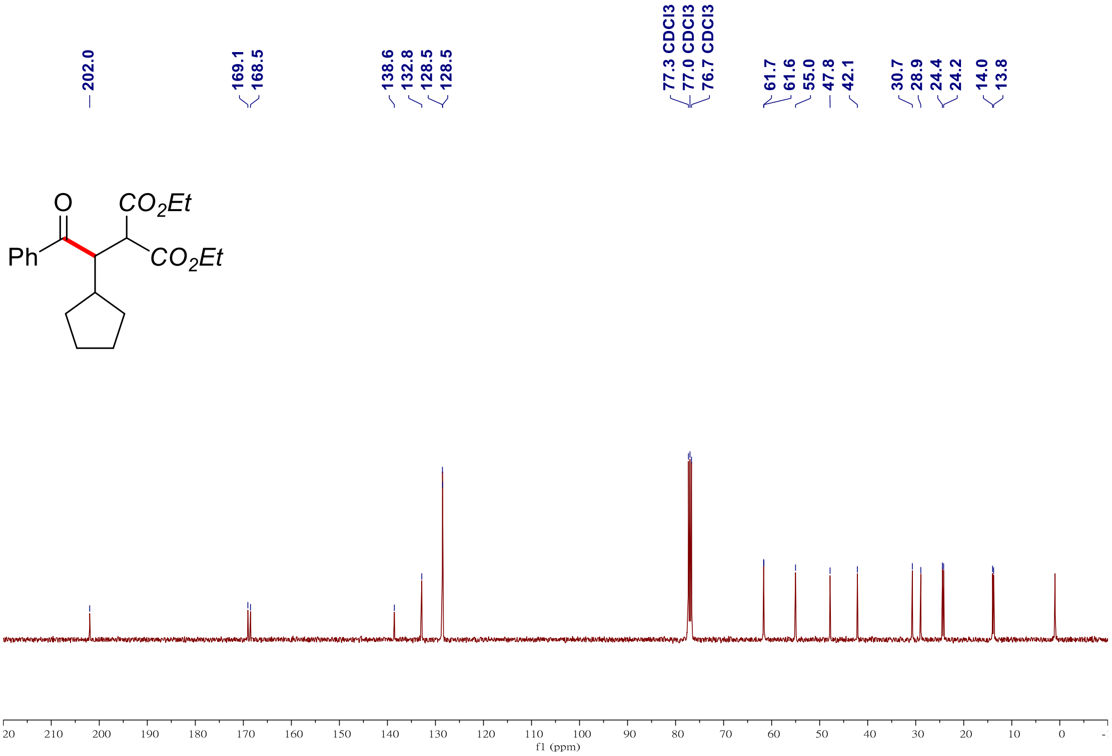

^1H NMR (400 MHz, $CDCl_3$) of **4e**, See procedure

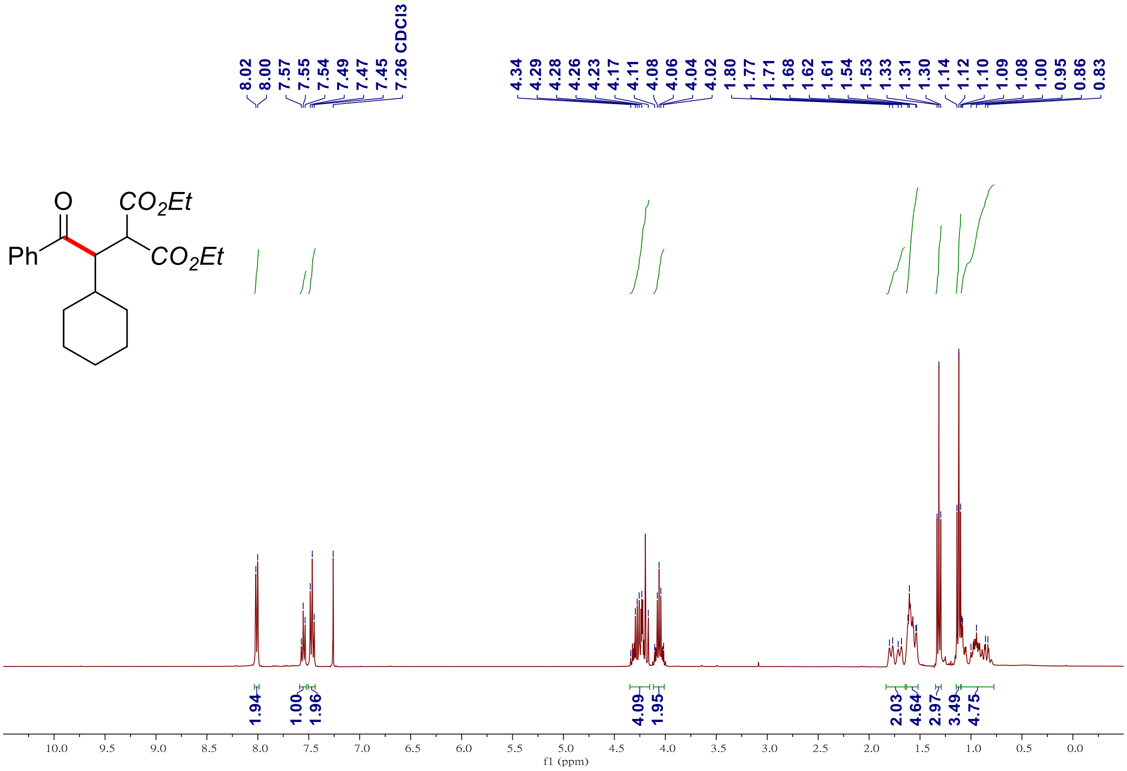

[Back to the contents](#)

$^{13}C\{^{1}H\}$ NMR (101 MHz, $CDCl_3$) of **4e**

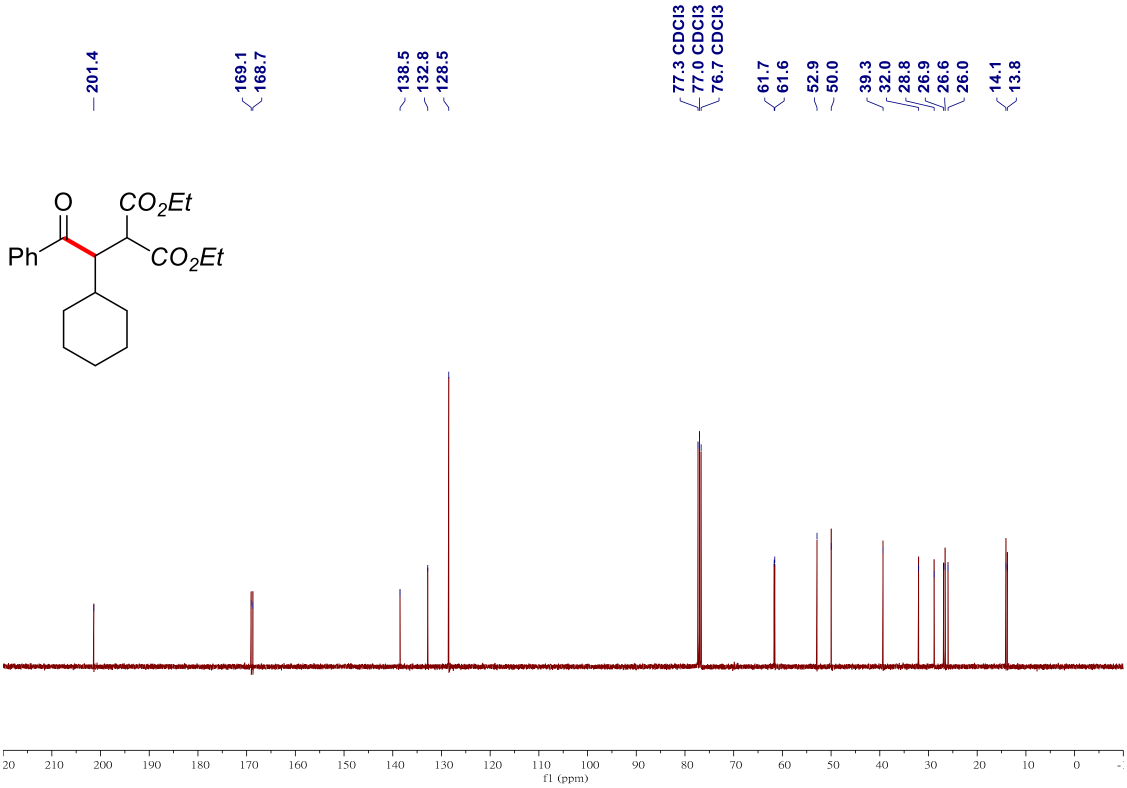

^{1}H NMR (300 MHz, $CDCl_3$) of **4f**, [See procedure](#)

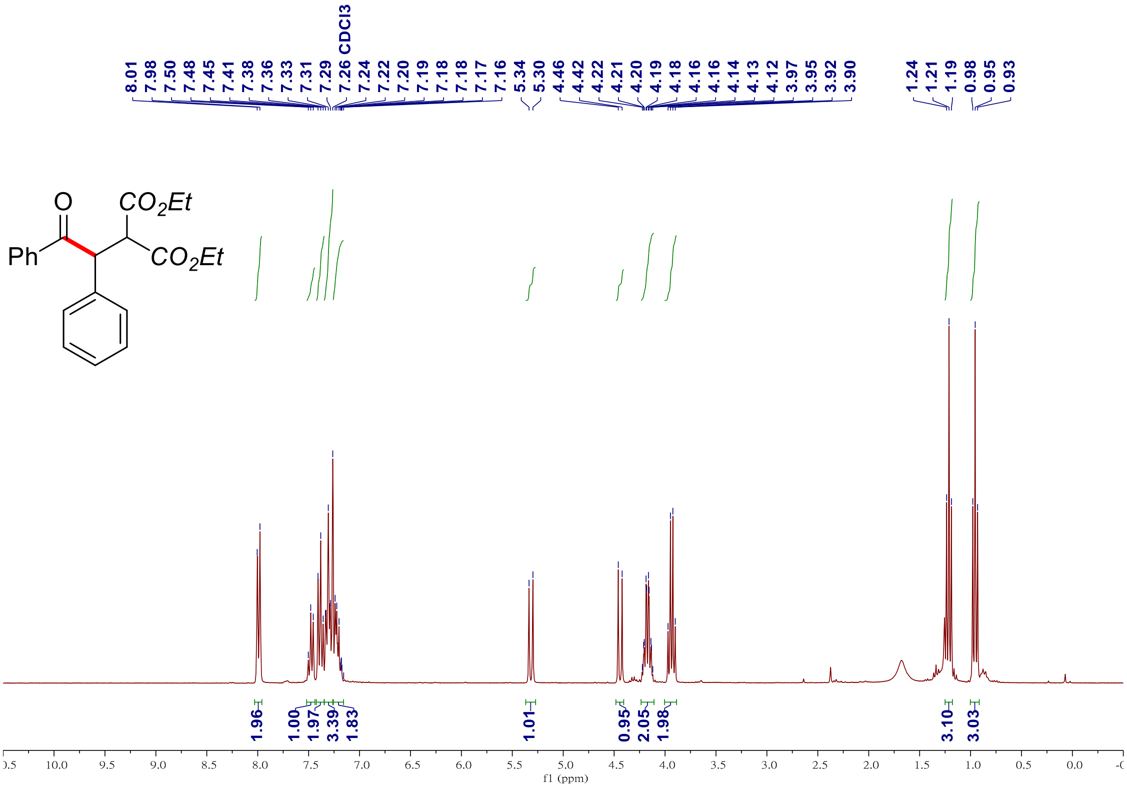

Back to the contents

$^{13}C\{^{1}H\}$ NMR (101 MHz, $CDCl_3$) of **4f**

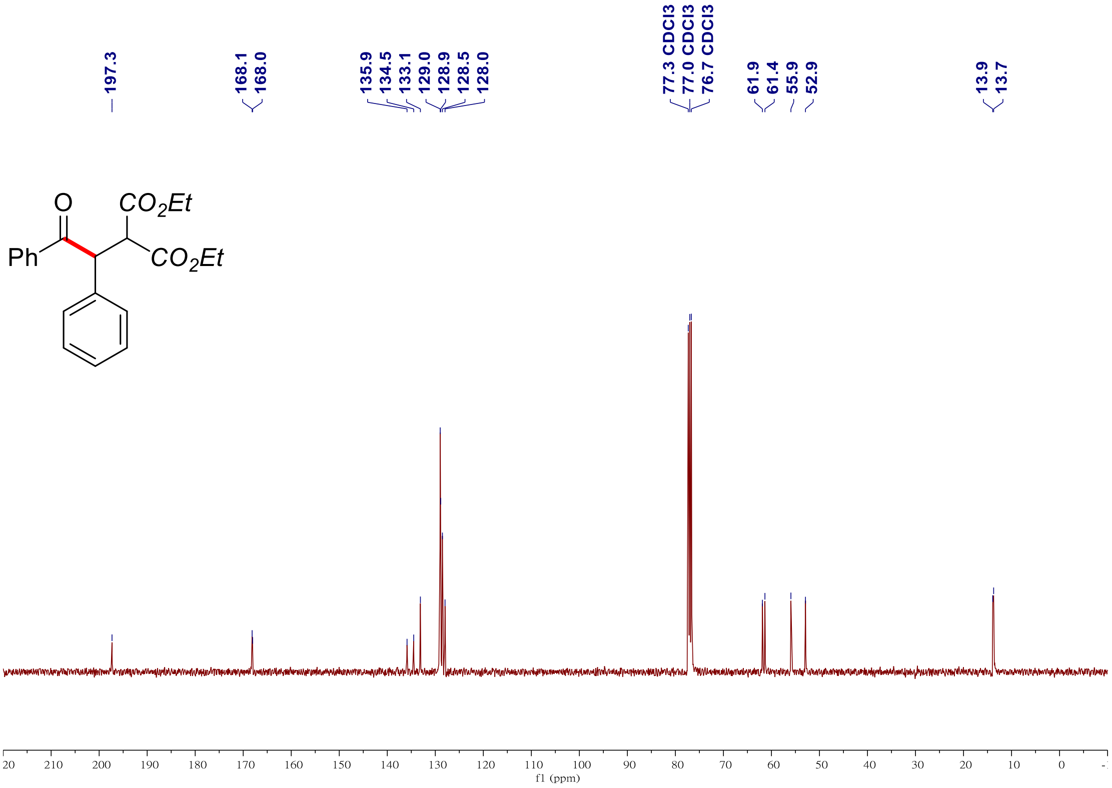

^{1}H NMR (300 MHz, $CDCl_3$) of **4g**, See procedure

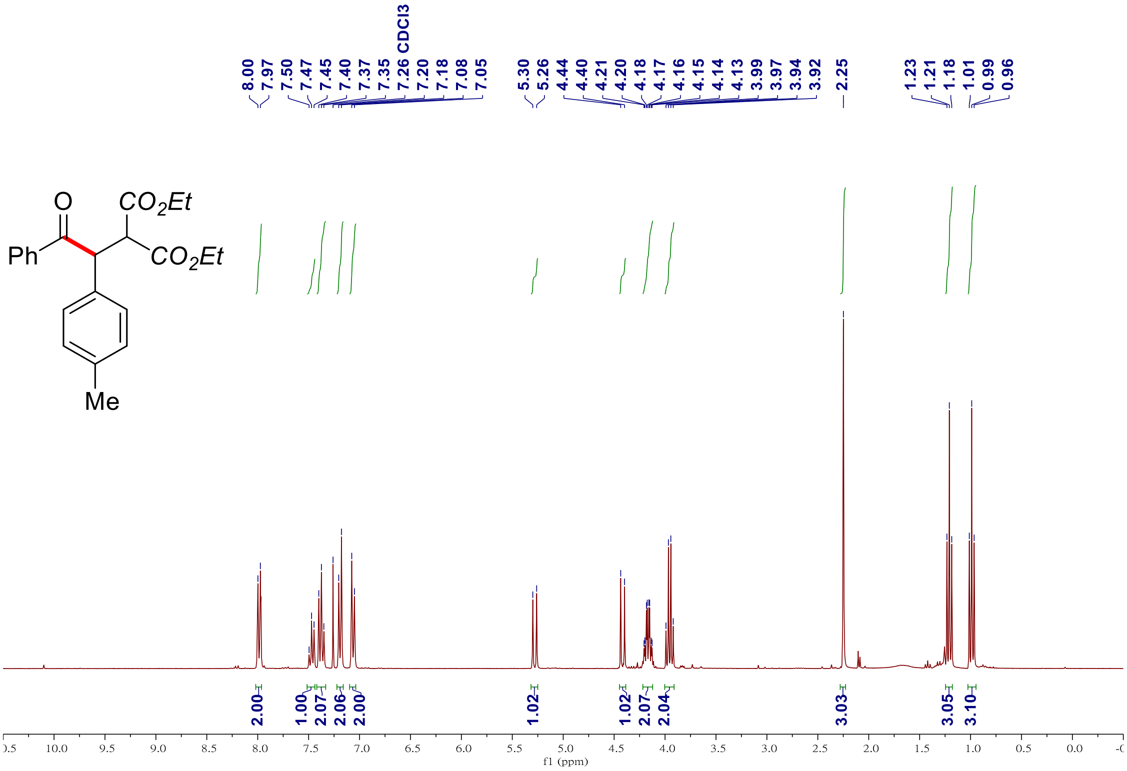

[Back to the contents](#)

$^{13}C\{^1H\}$ NMR (101 MHz, $CDCl_3$) of **4g**

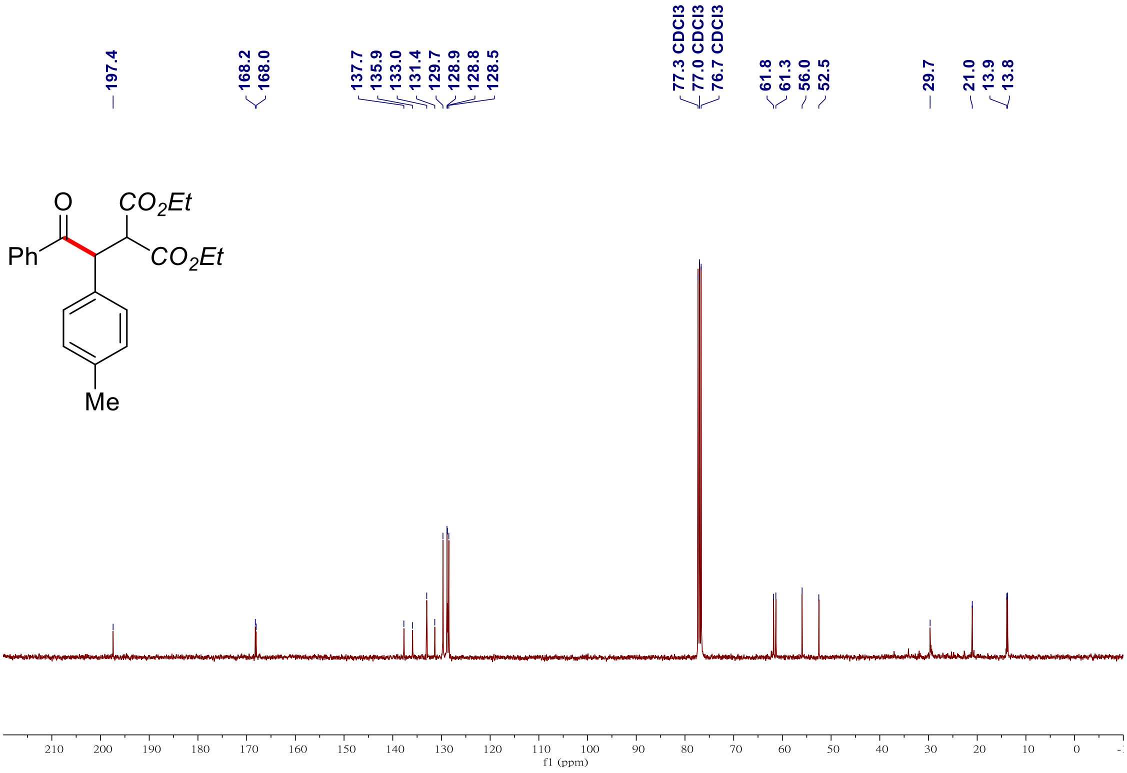

^1H NMR (300 MHz, $CDCl_3$) of **4h**, [See procedure](#)

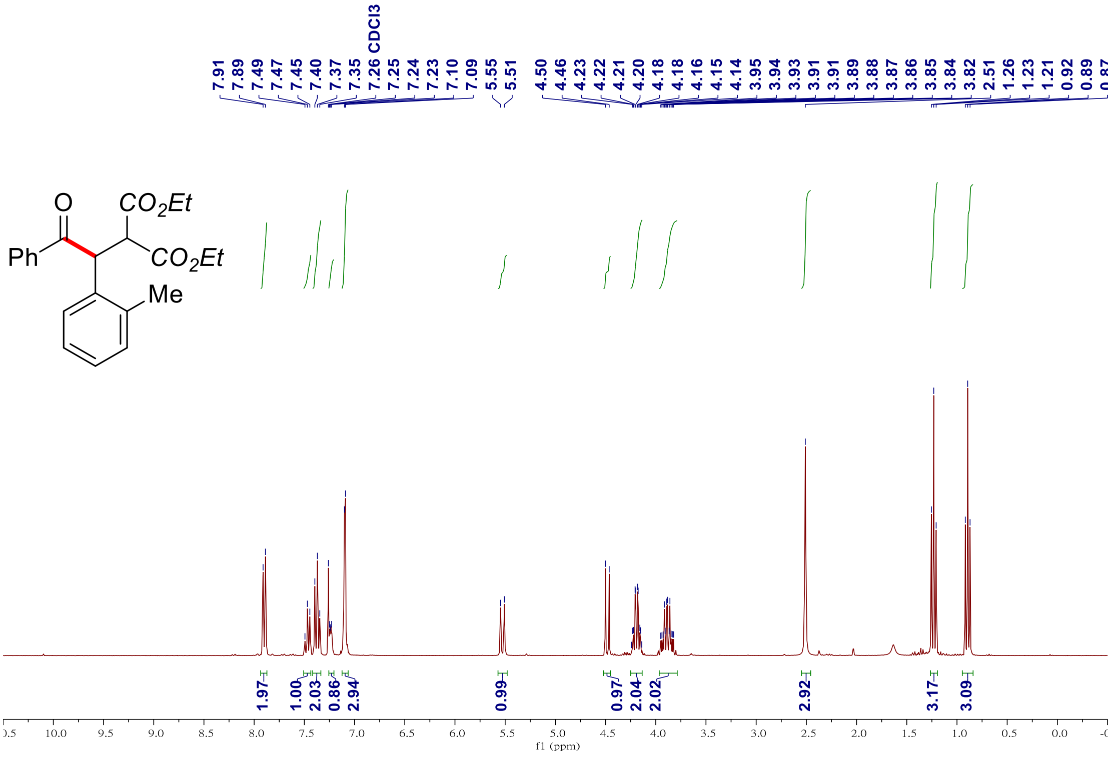

[Back to the contents](#)

^{13}C{^{1}H} NMR (101 MHz, CDCl$_3$) of **4h**

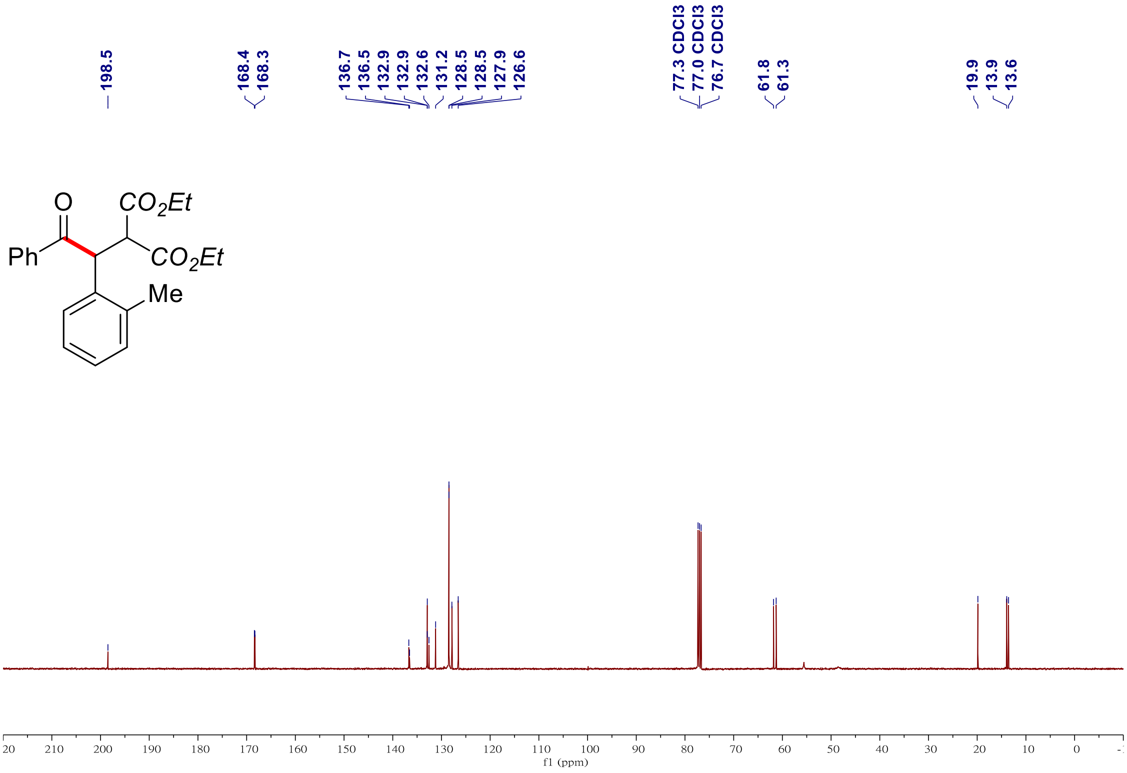

^{1}H NMR (300 MHz, CDCl$_3$) of **4i**, See procedure

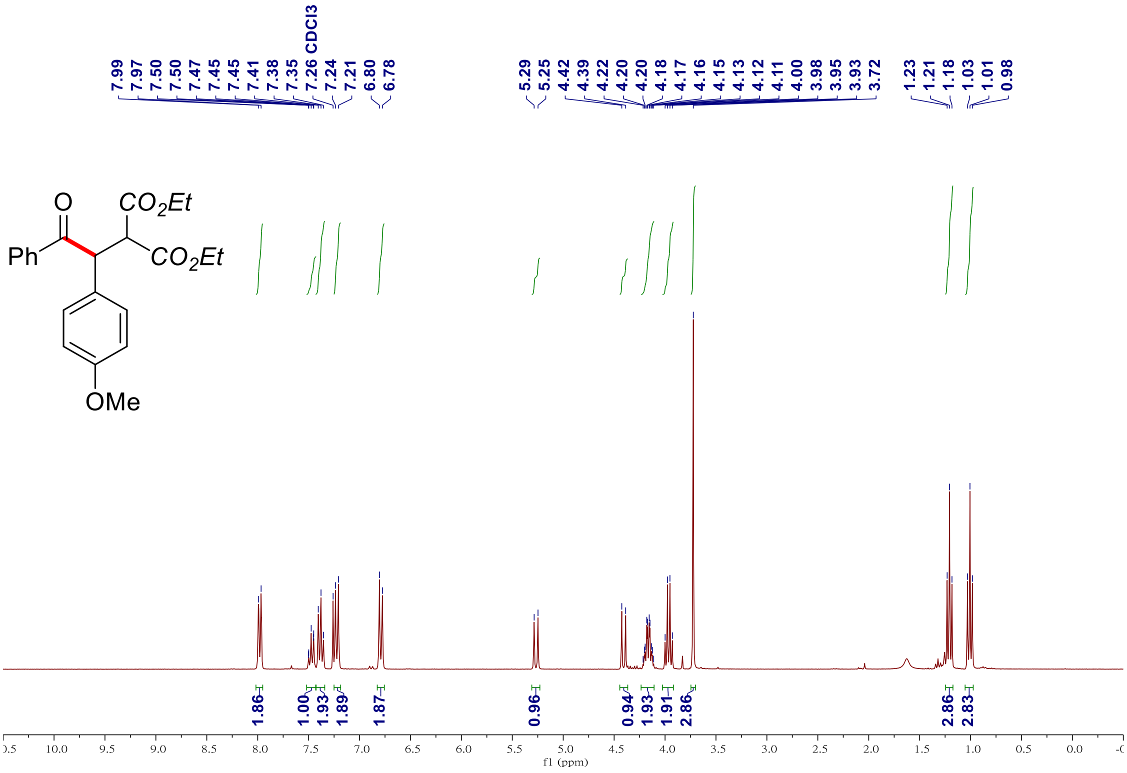

[Back to the contents](#)

$^{13}C\{^{1}H\}$ NMR (101 MHz, $CDCl_3$) of **4i**

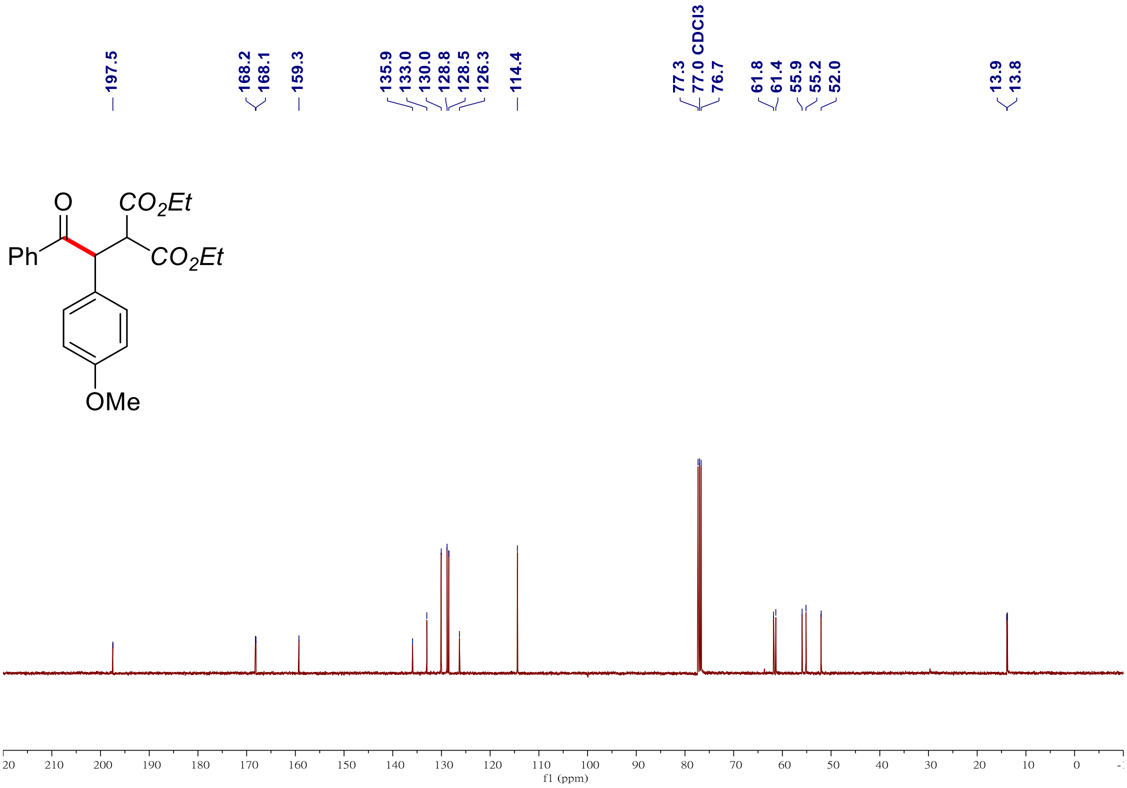

^{1}H NMR (300 MHz, $CDCl_3$) of **4j**, [See procedure](#)

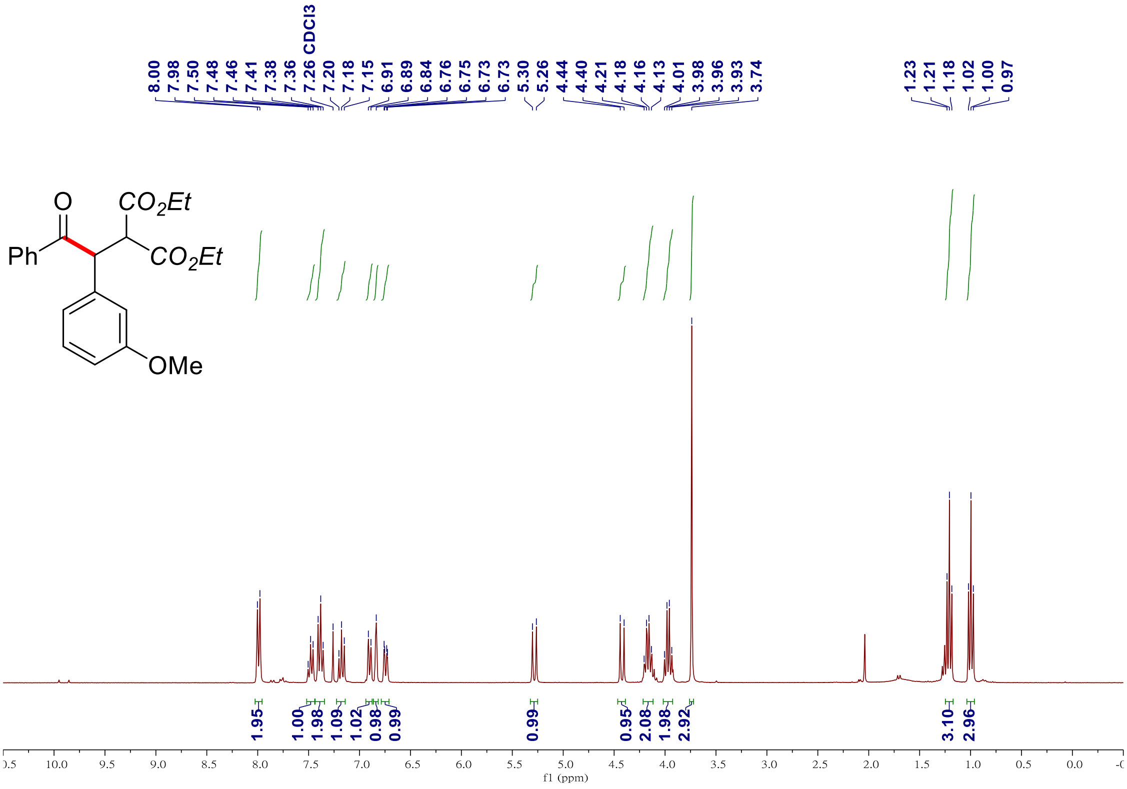

[Back to the contents](#)

$^{13}C\{^{1}H\}$ NMR (101 MHz, $CDCl_3$) of **4j**

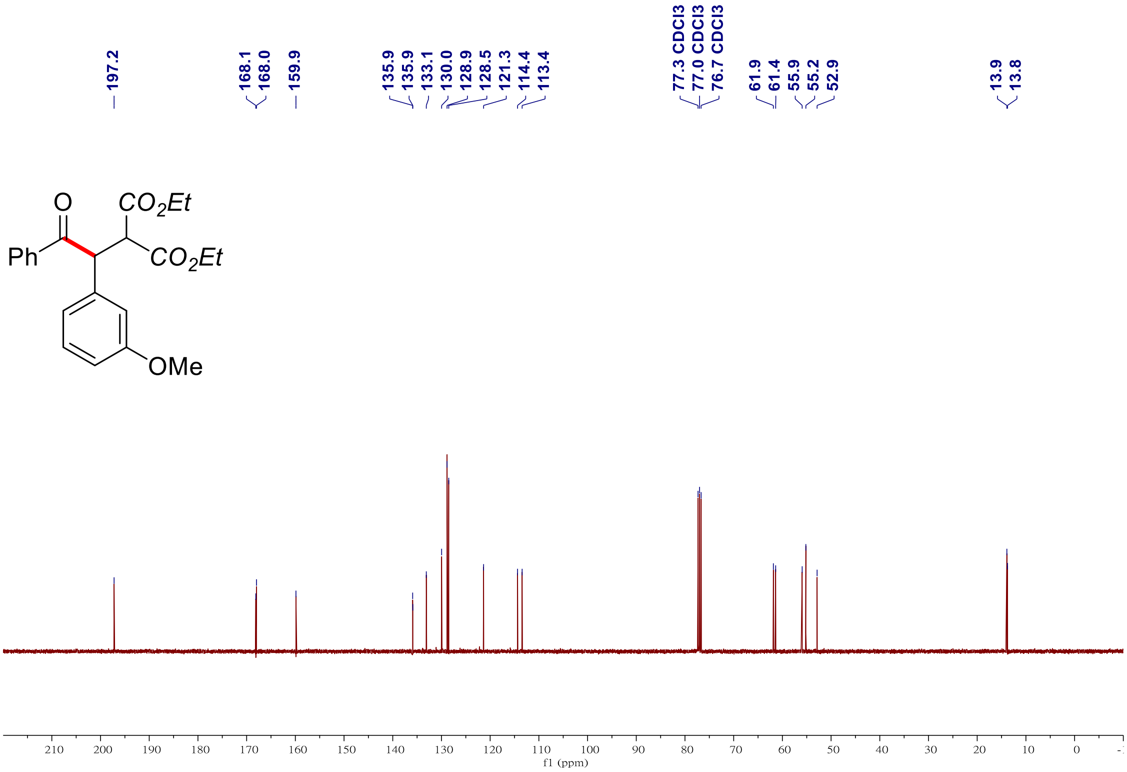

^{1}H NMR (400 MHz, $CDCl_3$) of **4k**, [See procedure](#)

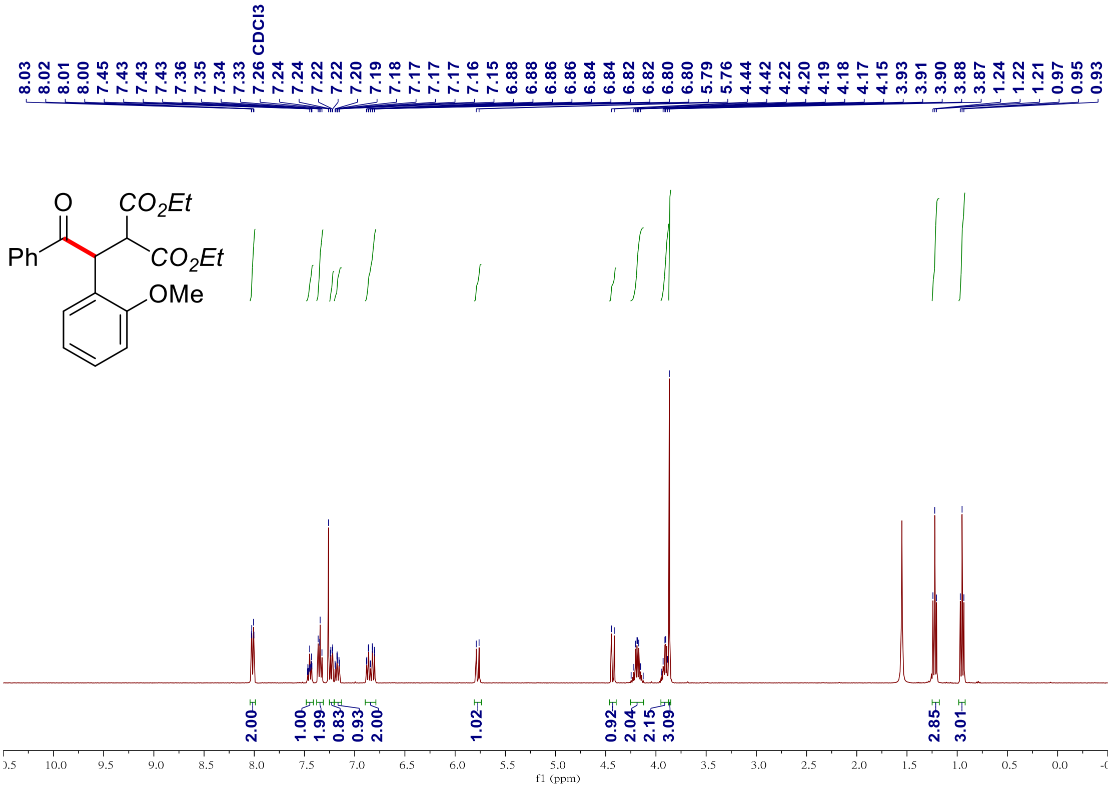

[Back to the contents](#)

$^{13}C\{^1H\}$ NMR (101 MHz, $CDCl_3$) of **4k**

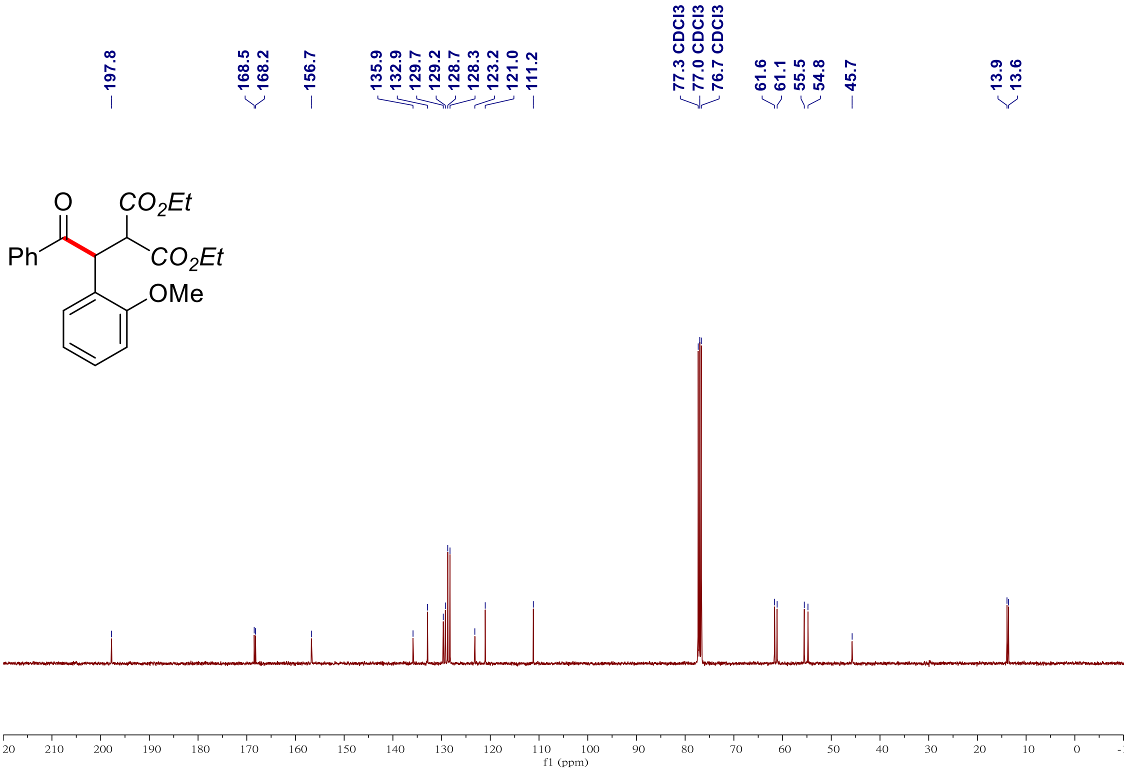

^1H NMR (300 MHz, $CDCl_3$) of **4l**, [See procedure](#)

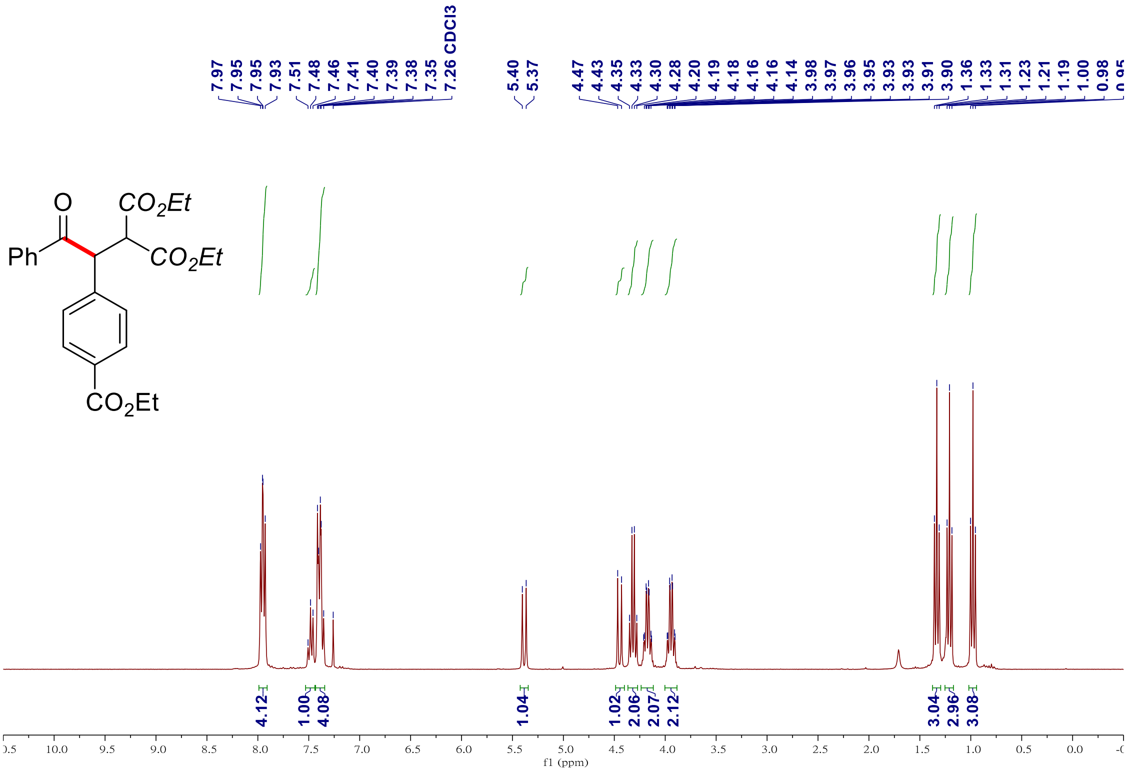

[Back to the contents]

$^{13}C\{^1H\}$ NMR (101 MHz, $CDCl_3$) of **4l**

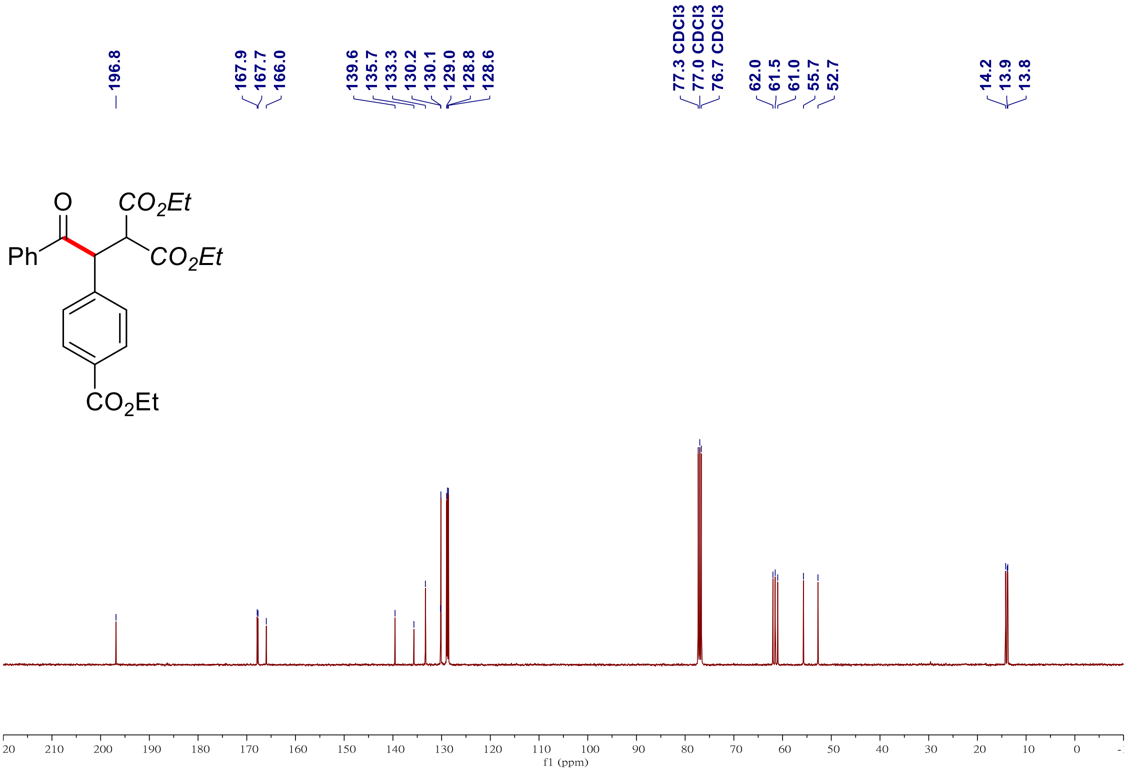

^1H NMR (300 MHz, $CDCl_3$) of **4m**, [See procedure]

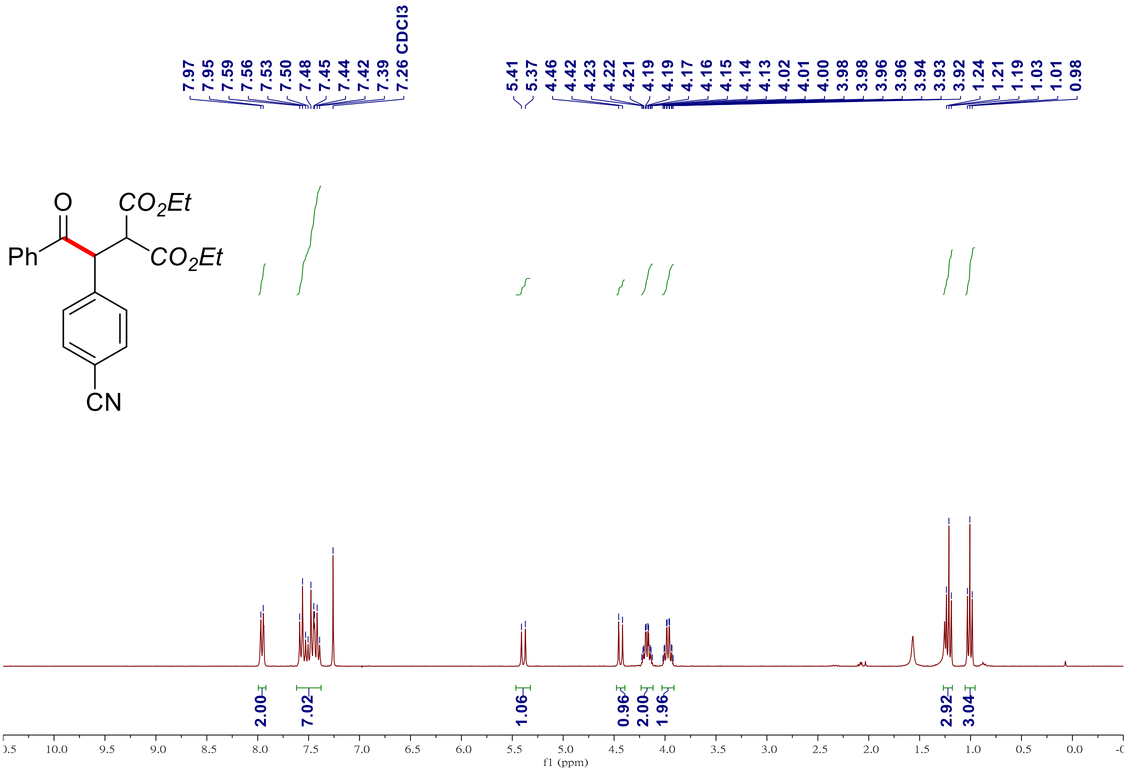

Back to the contents

$^{13}C\{^1H\}$ NMR (101 MHz, $CDCl_3$) of **4m**

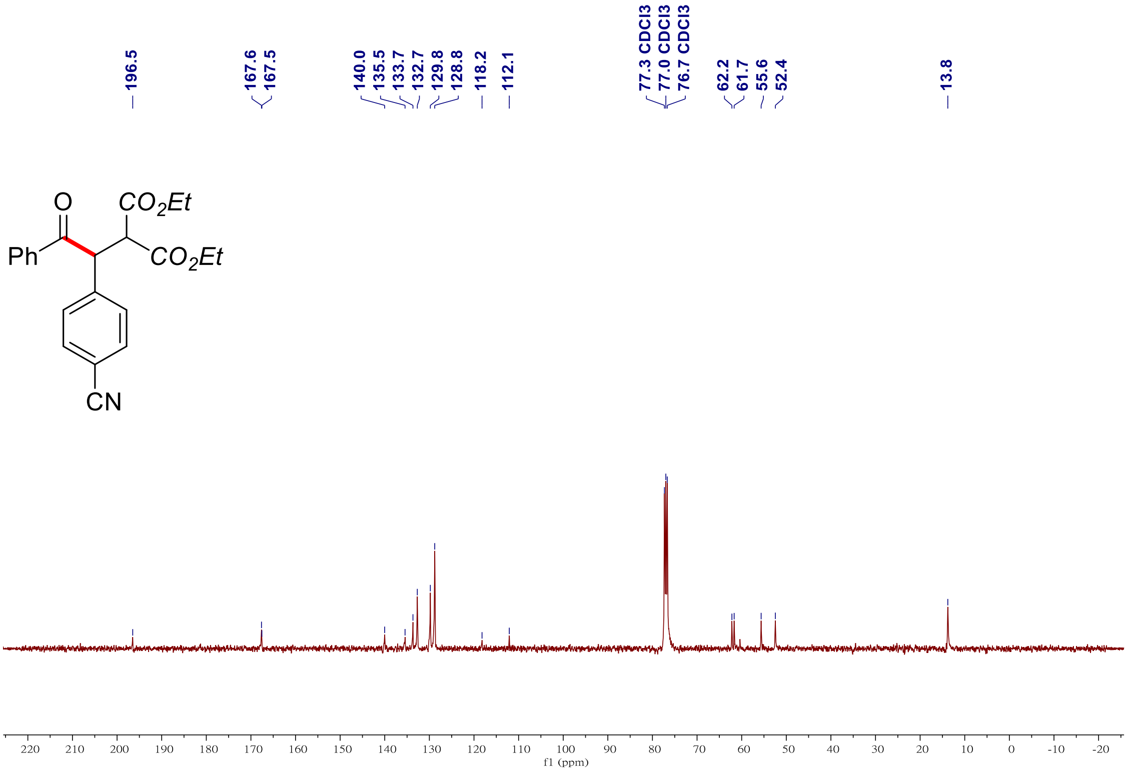

^1H NMR (300 MHz, $CDCl_3$) of **4n**, See procedure

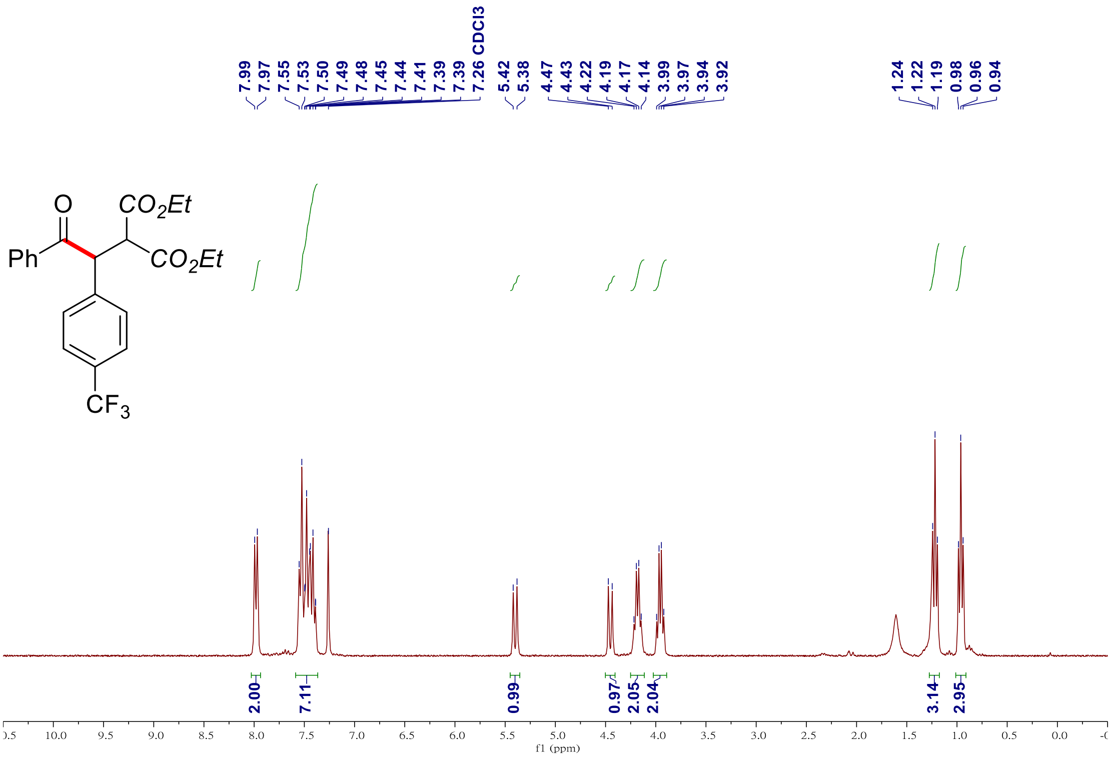

[Back to the contents](#)

$^{13}C\{^{1}H\}$ NMR (101 MHz, $CDCl_3$) of **4n**

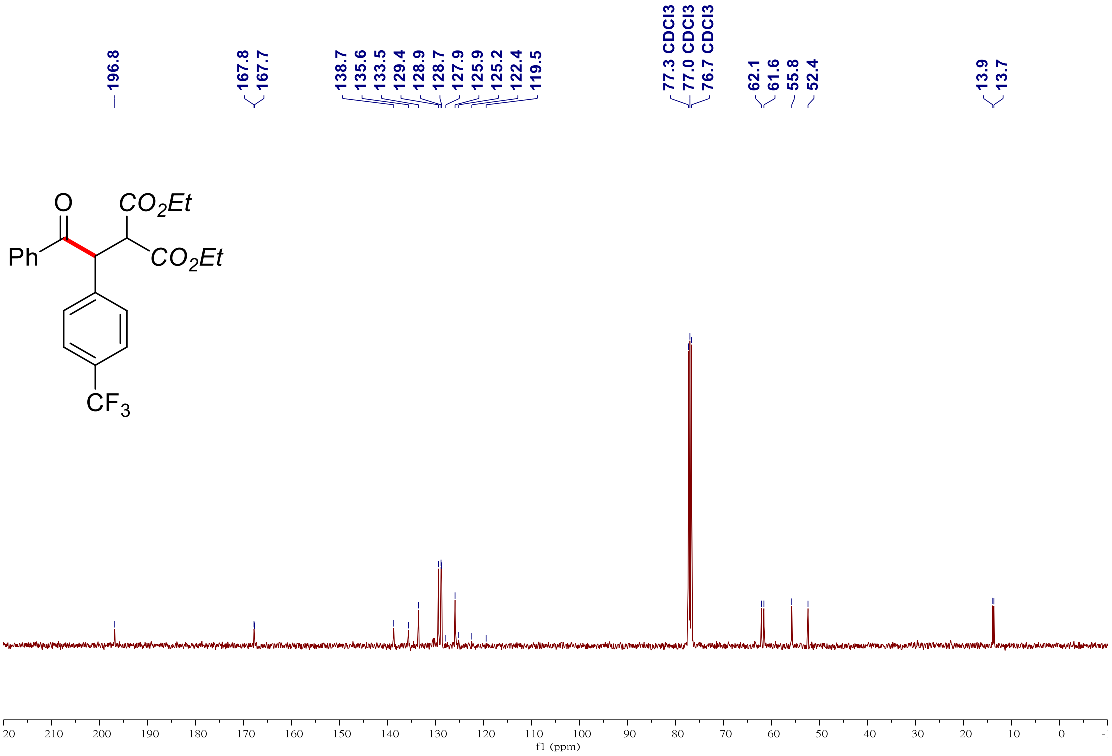

^{19}F NMR (282 MHz, $CDCl_3$) of **4n**

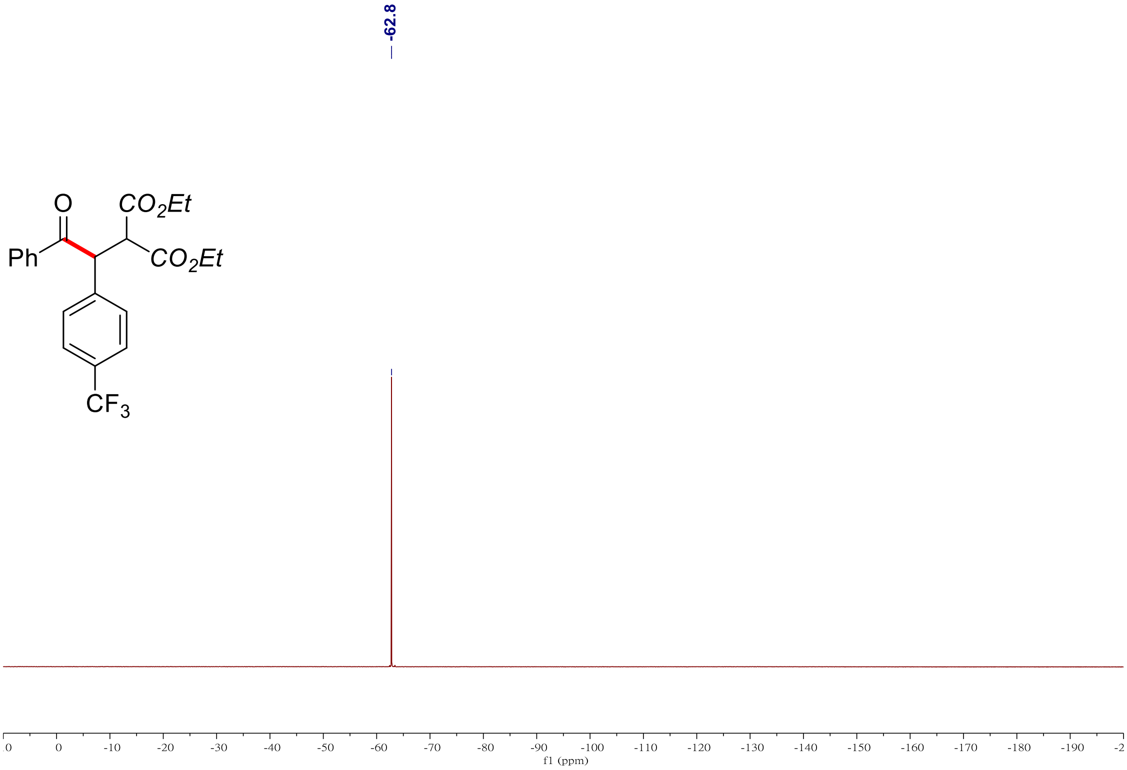

Back to the contents

^{1}H NMR (300 MHz, $CDCl_3$) of **4o**, See procedure

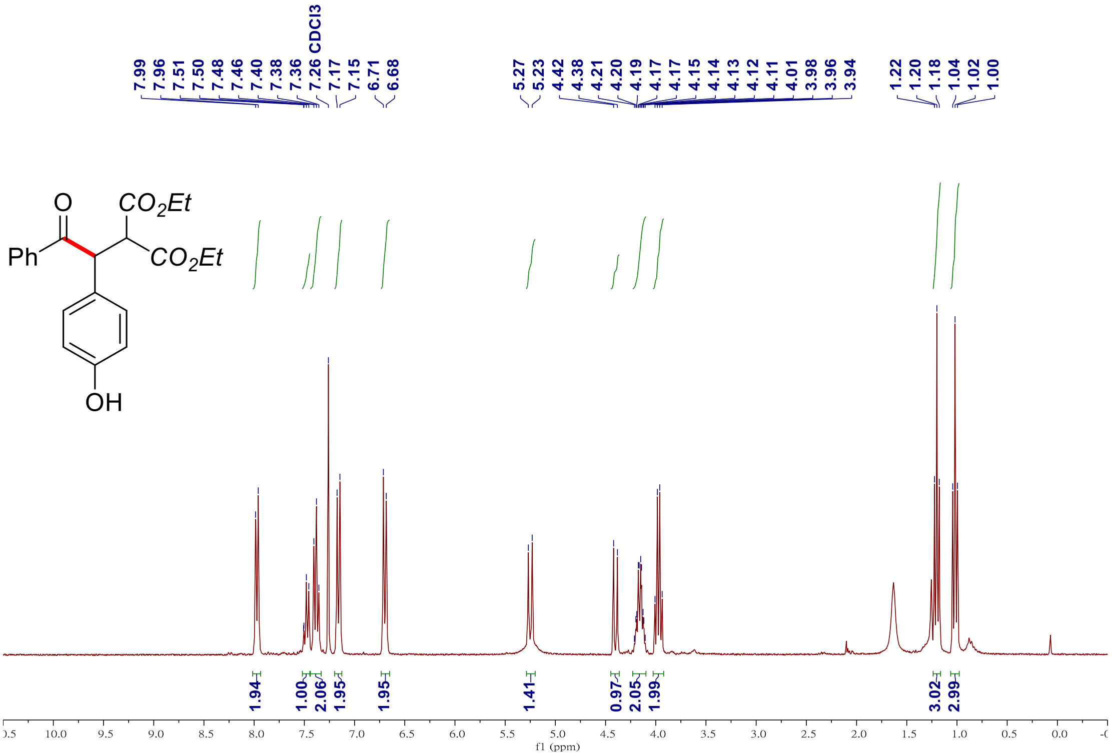

^{13}C{^{1}H} NMR (101 MHz, $CDCl_3$) of **4o**

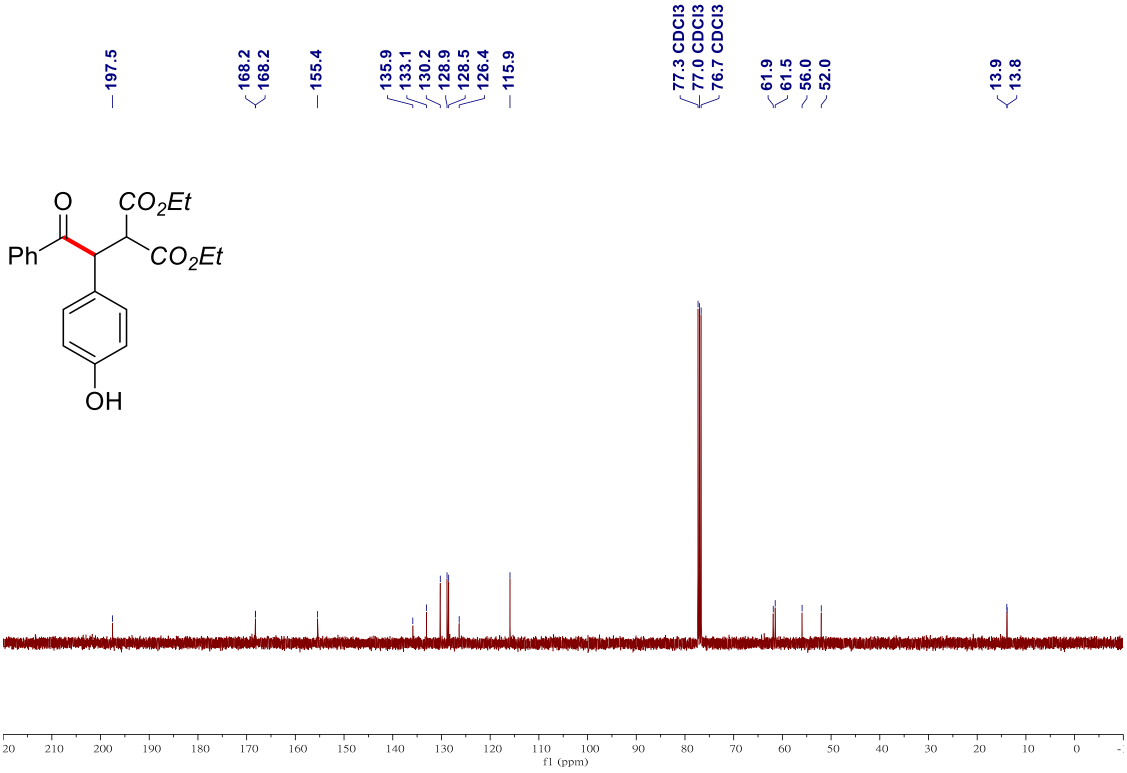

[Back to the contents]

^{1}H NMR (300 MHz, $CDCl_3$) of **4p**, See procedure

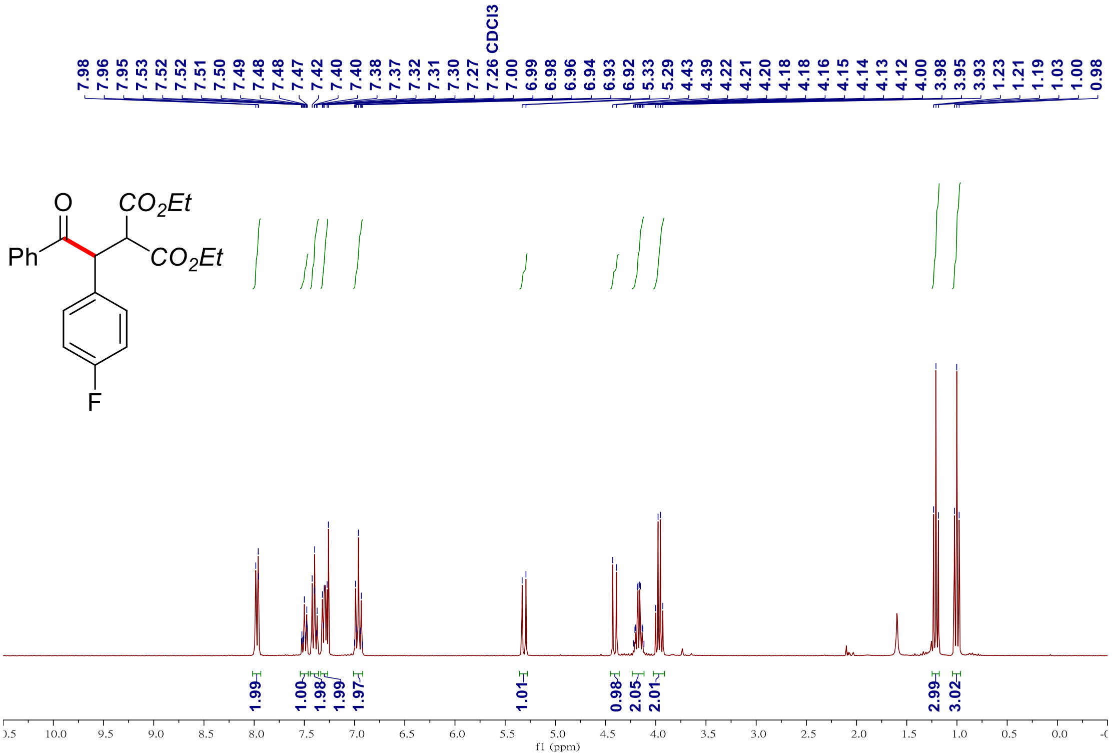

$^{13}C\{^1H\}$ NMR (101 MHz, $CDCl_3$) of **4p**

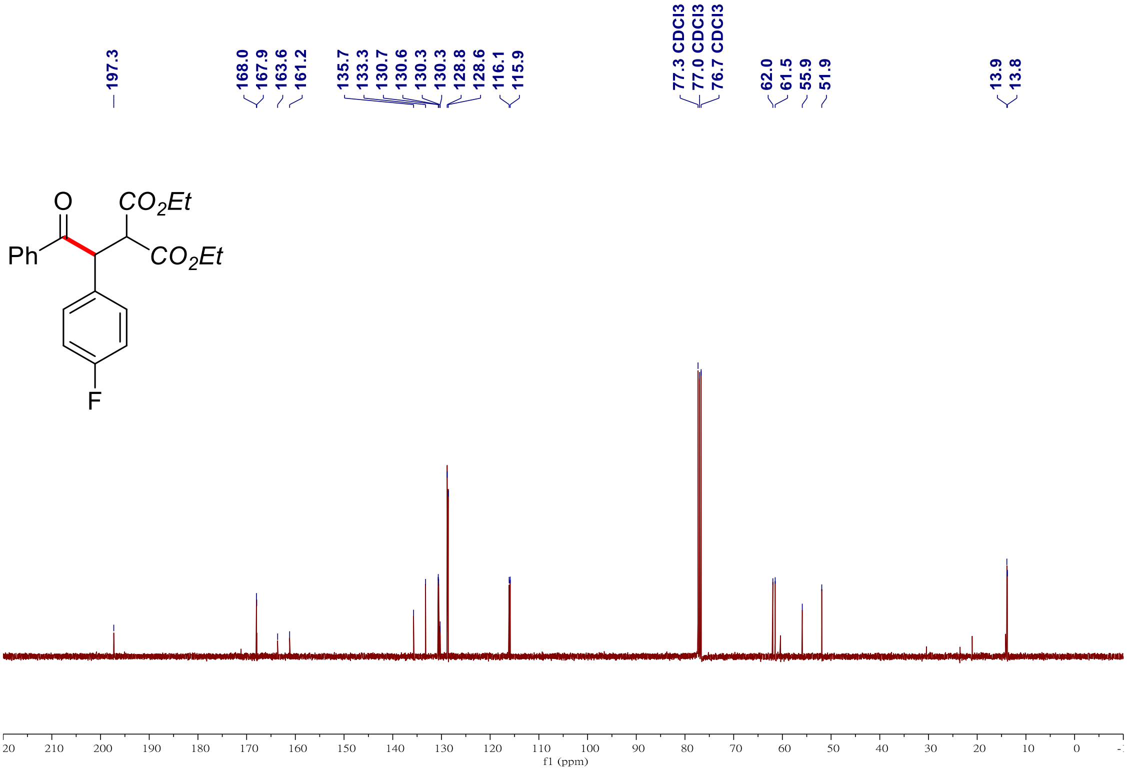

Back to the contents

^{19}F NMR (282 MHz, $CDCl_3$) of **4p**

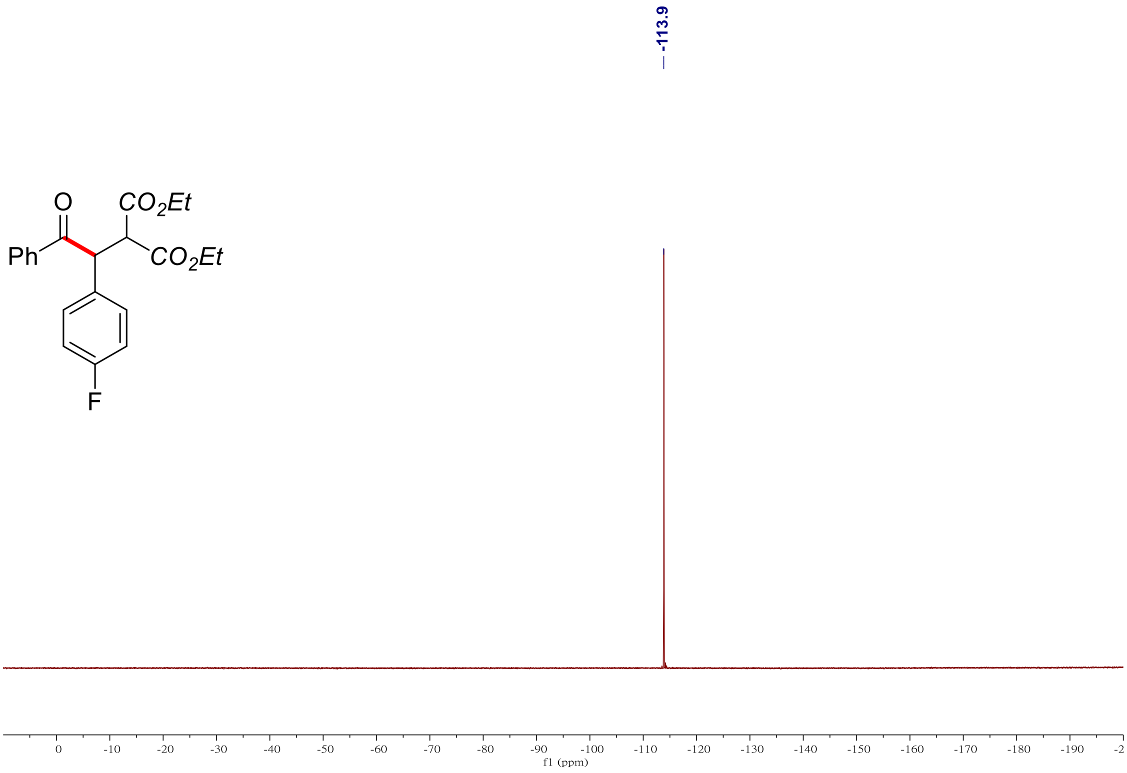

^{1}H NMR (300 MHz, $CDCl_3$) of **4q**, See procedure

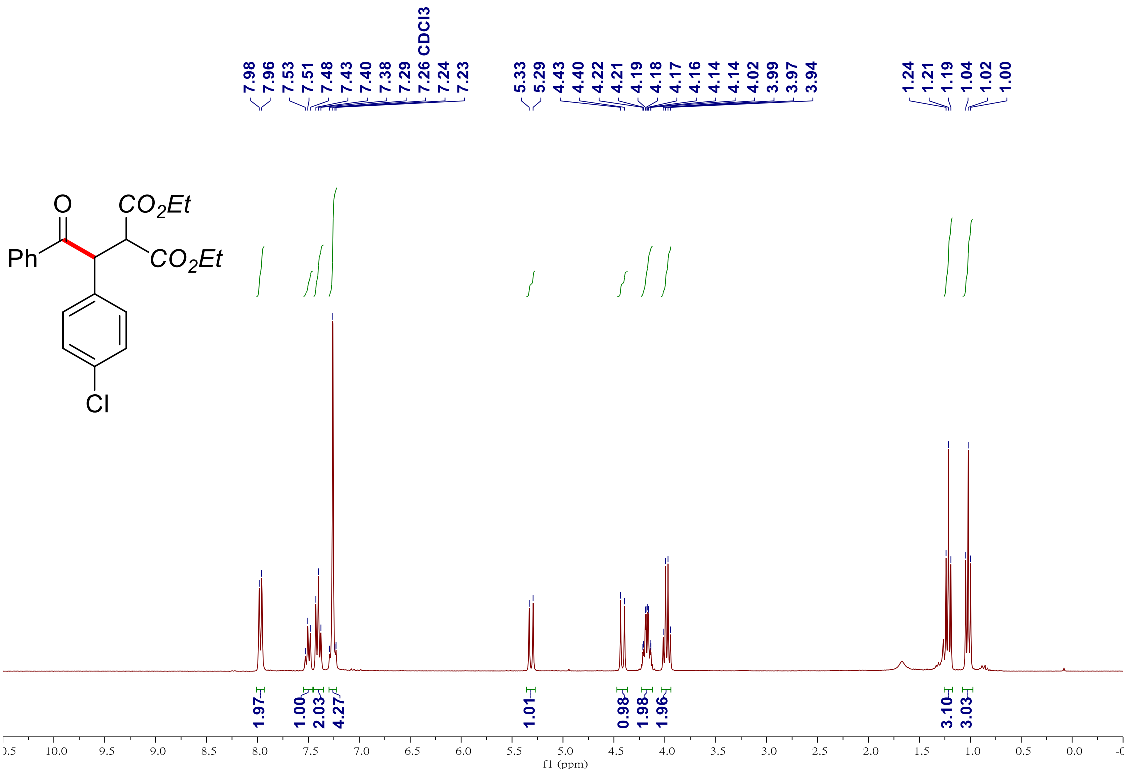

[Back to the contents](#)

$^{13}C\{^1H\}$ NMR (101 MHz, $CDCl_3$) of **4q**

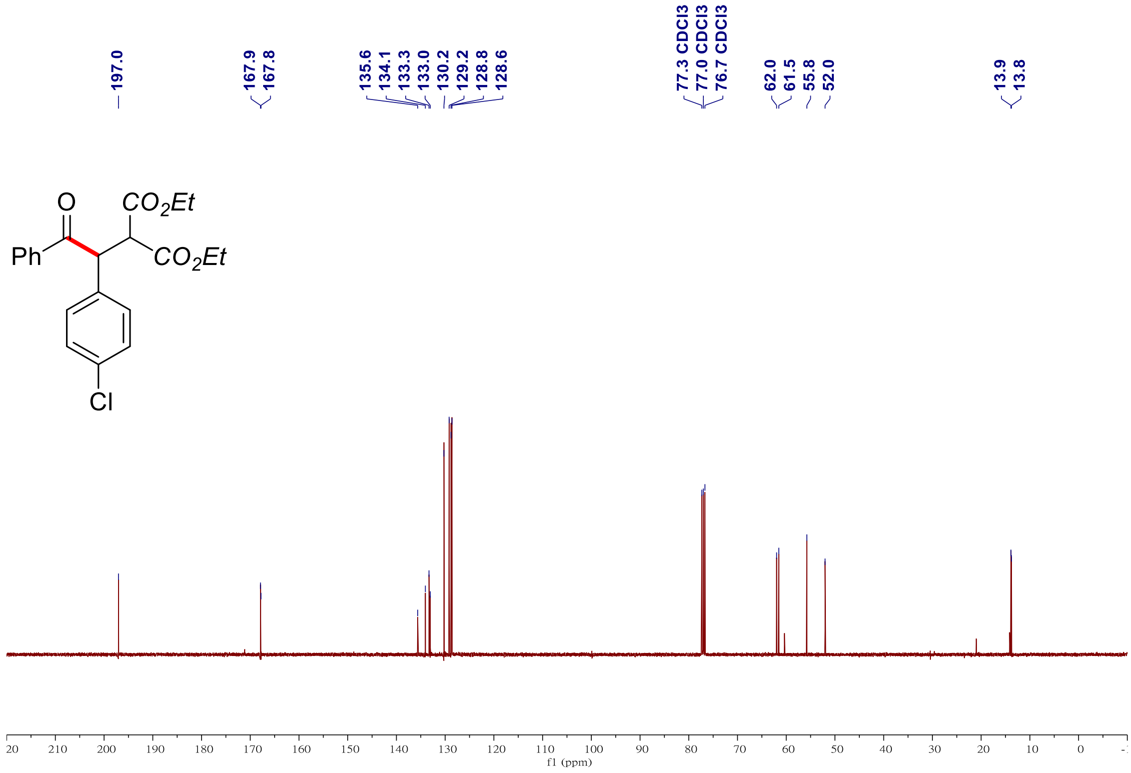

^1H NMR (300 MHz, $CDCl_3$) of **4r**, [See procedure](#)

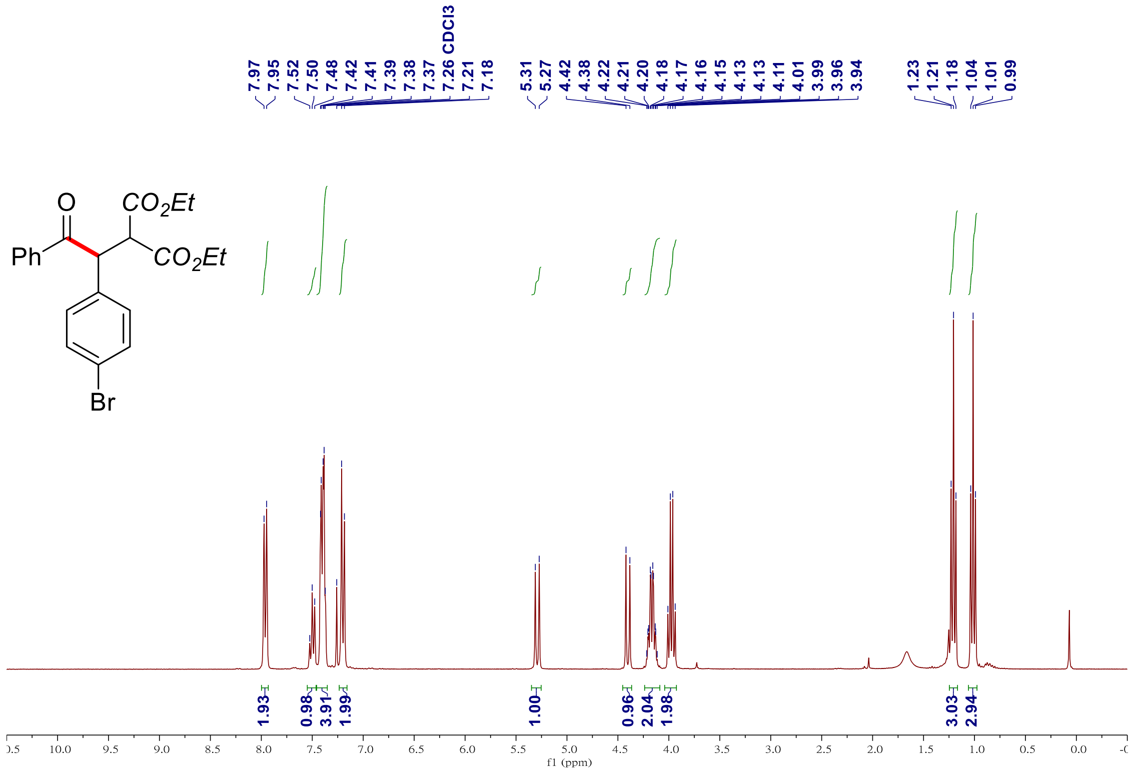

Back to the contents

$^{13}C\{^1H\}$ NMR (101 MHz, $CDCl_3$) of **4r**

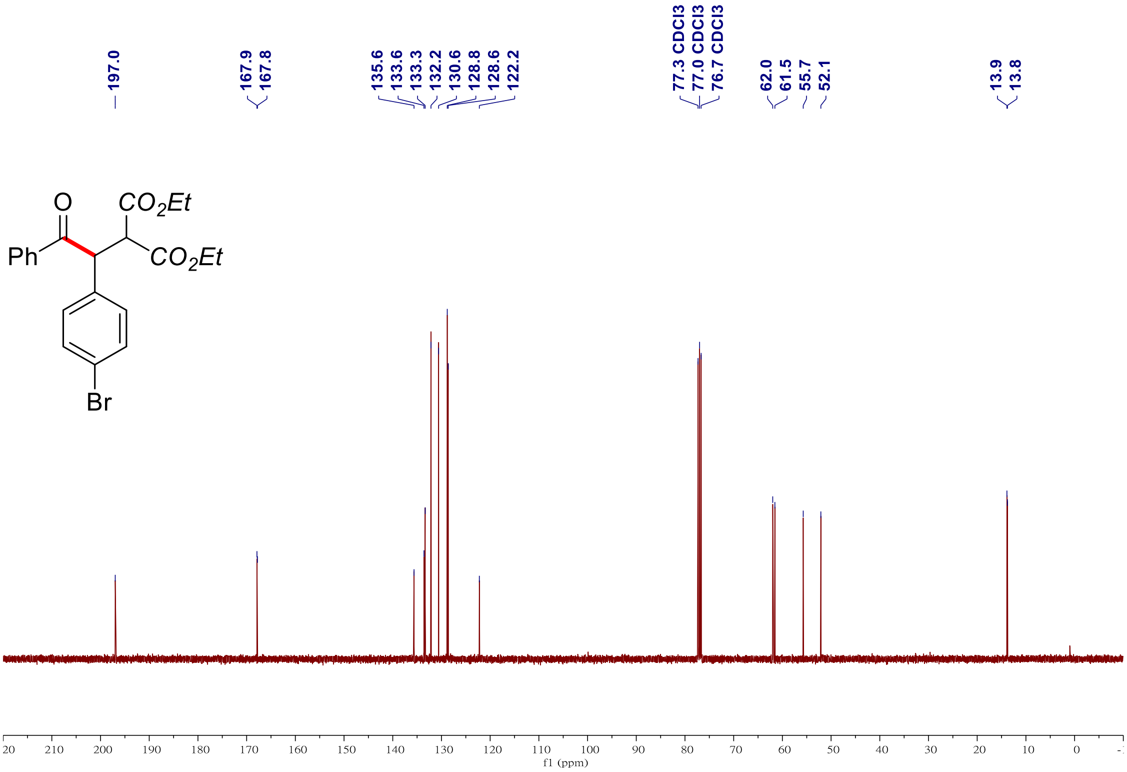

^1H NMR (300 MHz, $CDCl_3$) of **4s**, See procedure

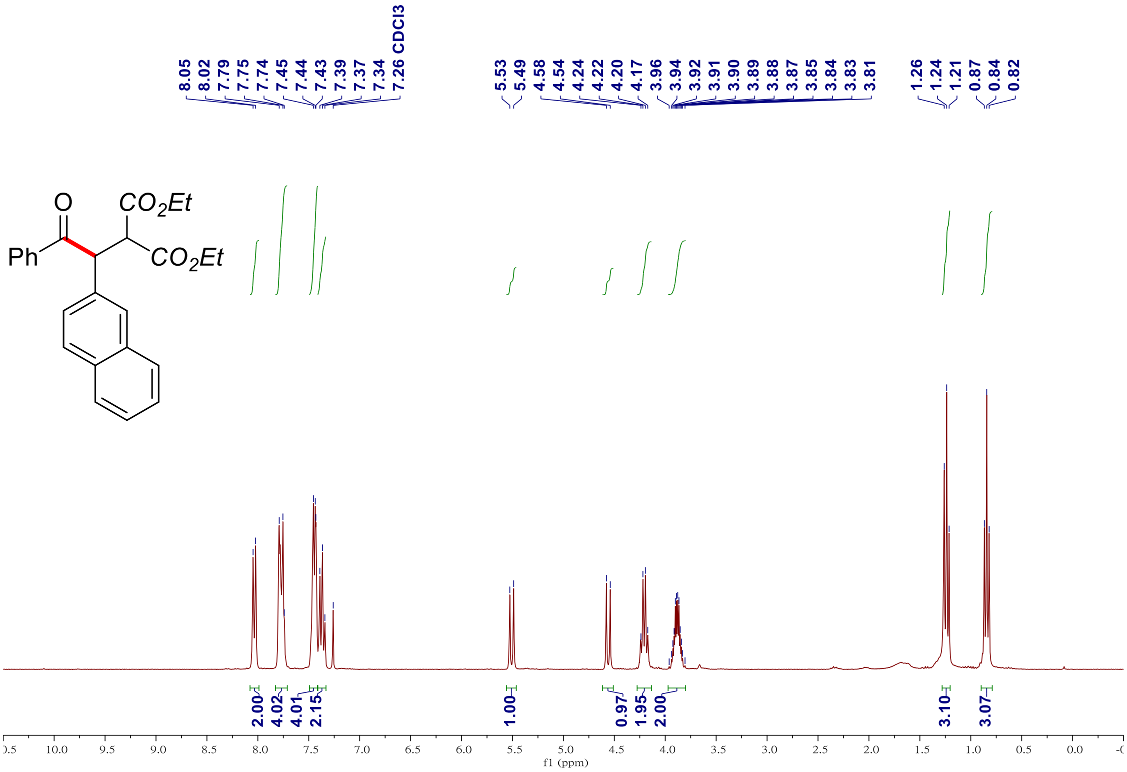

[Back to the contents](#)

$^{13}C\{^1H\}$ NMR (101 MHz, $CDCl_3$) of **4s**

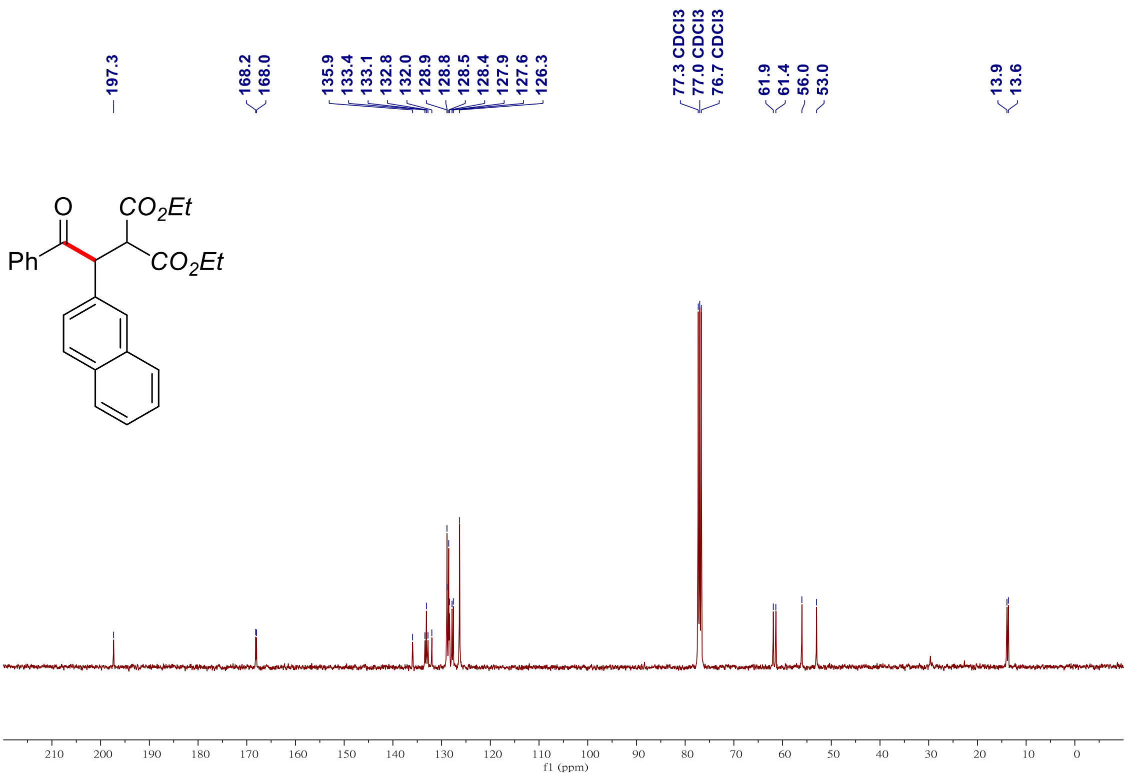

^{1}H NMR (400 MHz, $CDCl_3$) of **4t**, [See procedure](#)

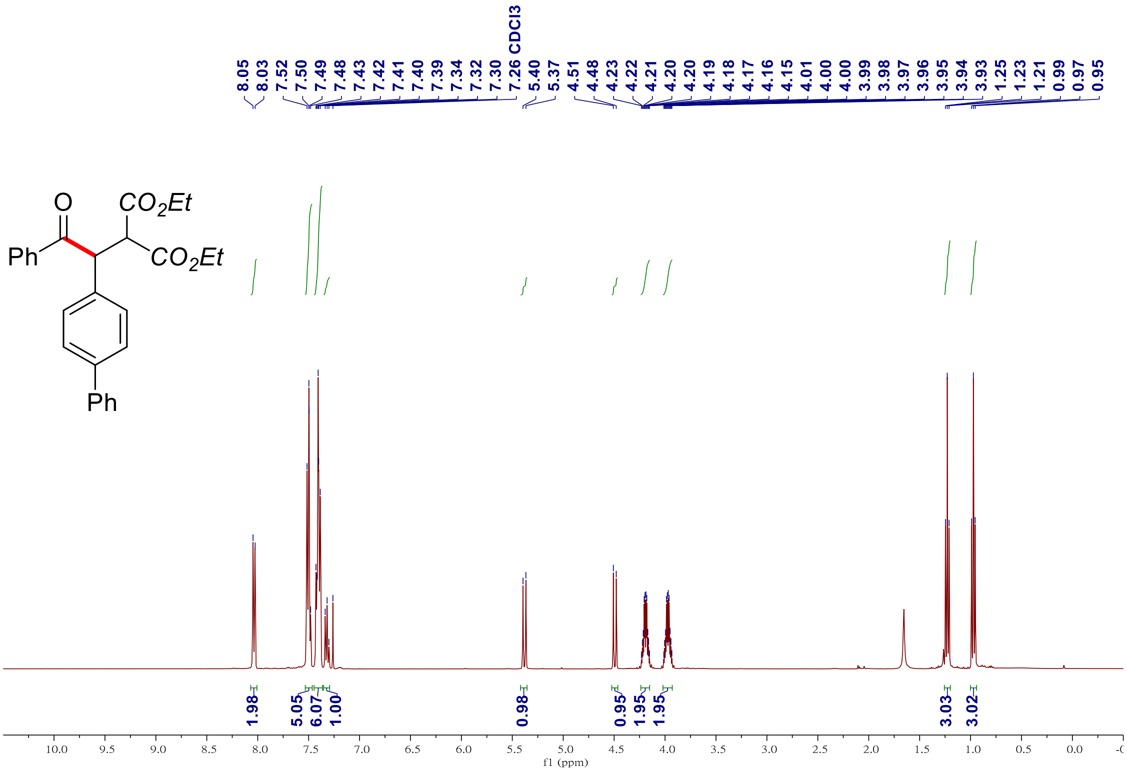

[Back to the contents](#)

$^{13}C\{^{1}H\}$ NMR (101 MHz, $CDCl_3$) of **4t**

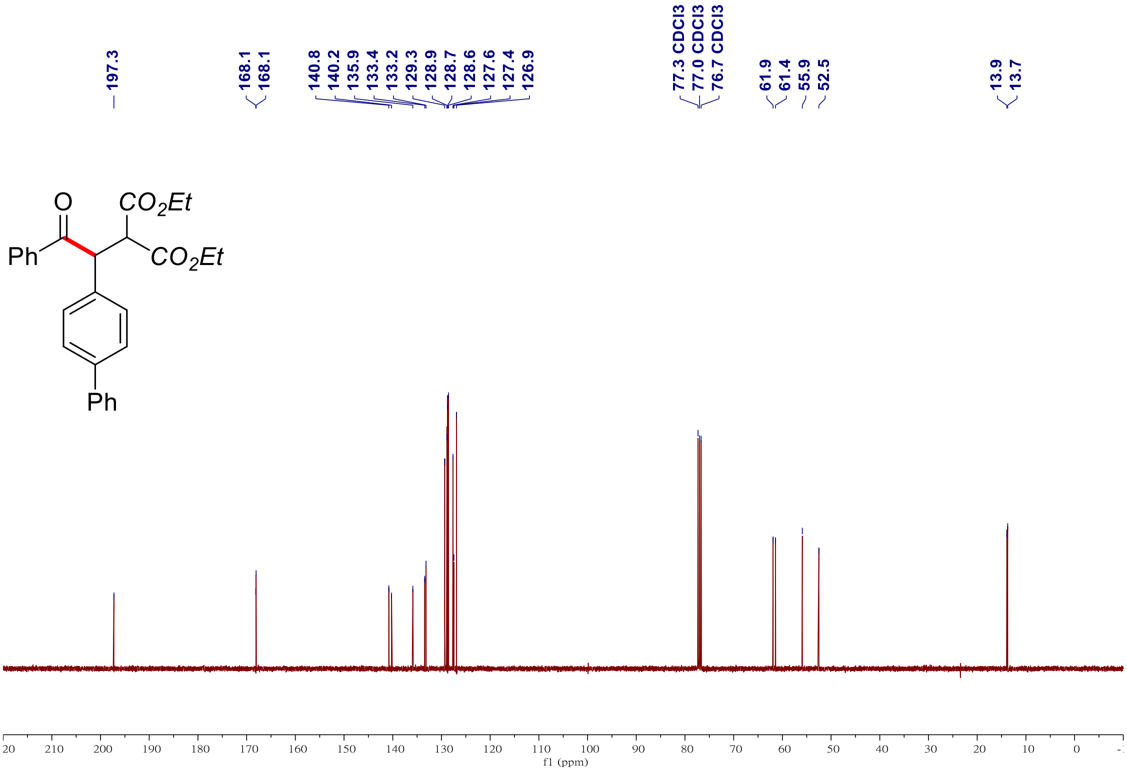

^{1}H NMR (300 MHz, $CDCl_3$) of **4u**, [See procedure](#)

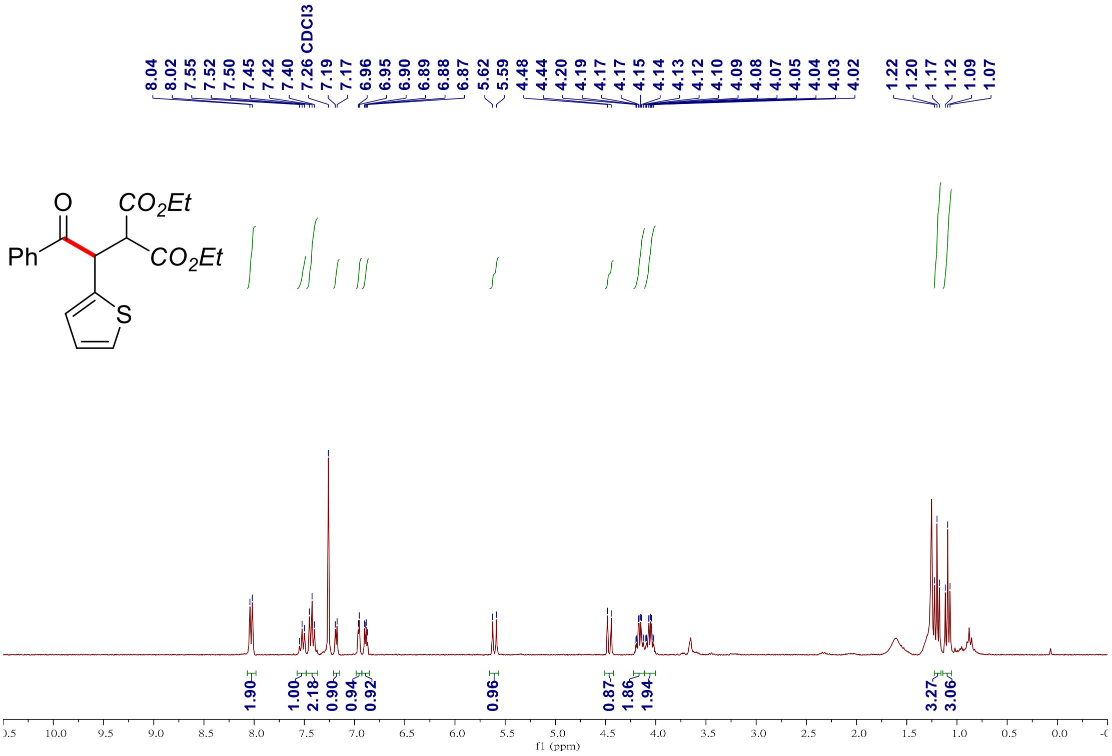

Back to the contents

$^{13}C\{^{1}H\}$ NMR (101 MHz, $CDCl_3$) of **4u**

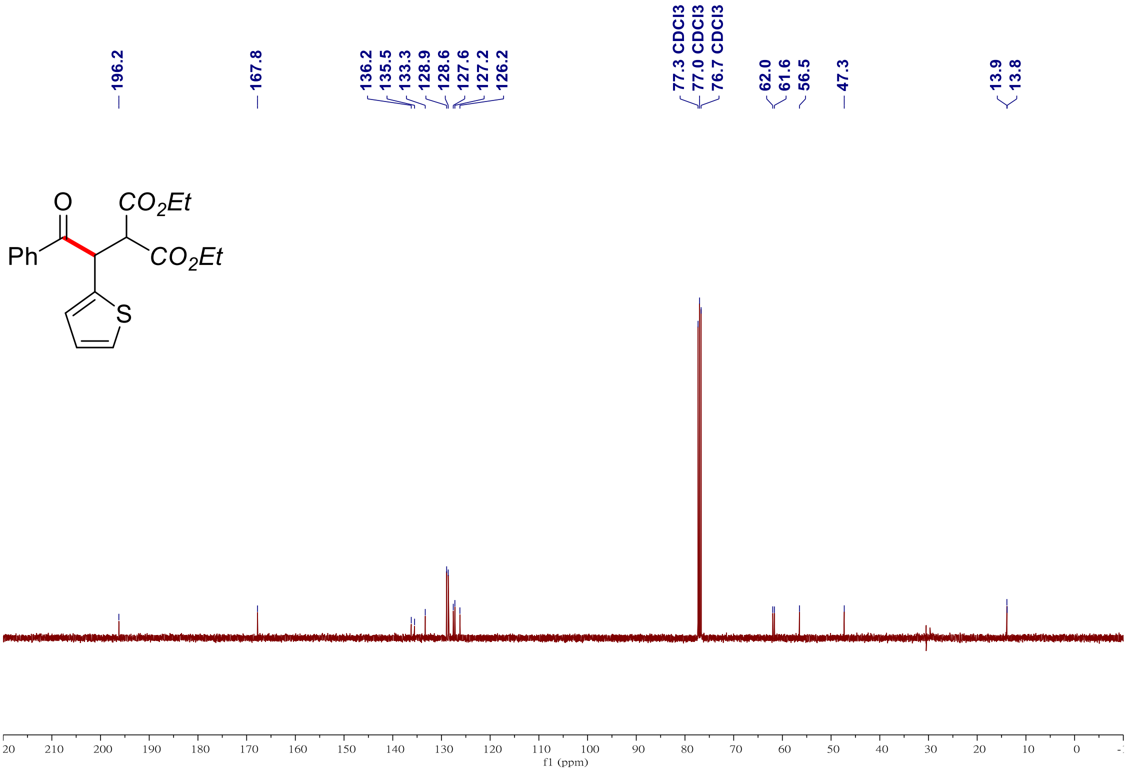

^{1}H NMR (300 MHz, $CDCl_3$) of **4v**, See procedure

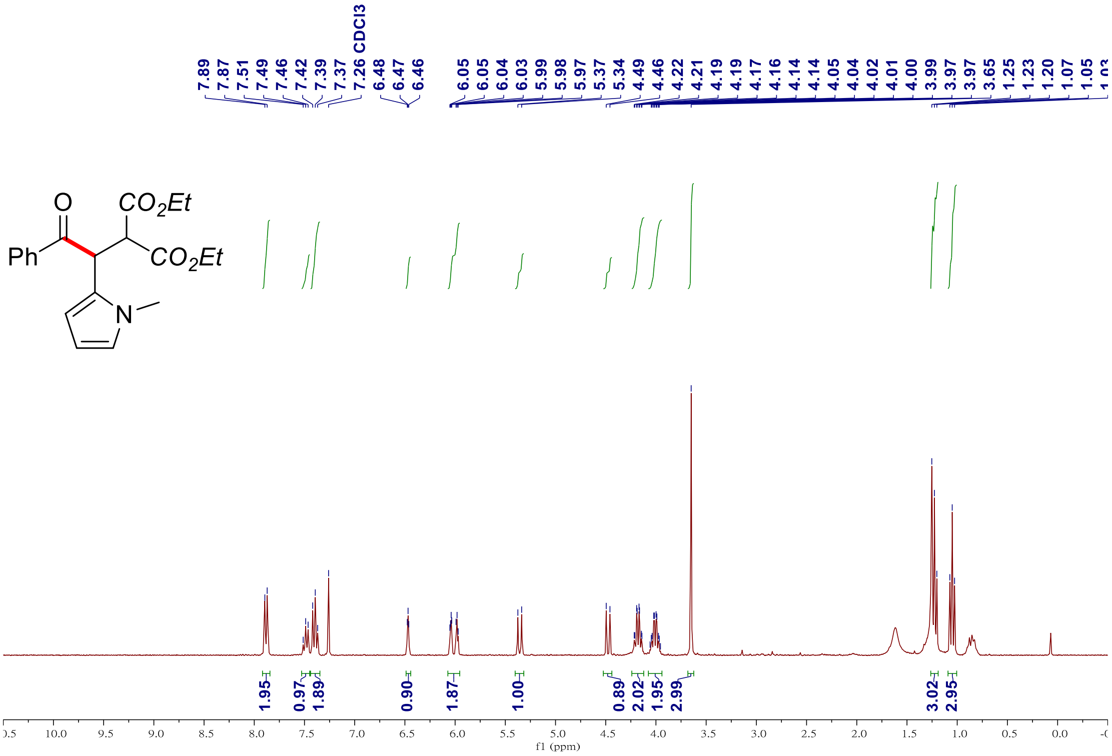

Back to the contents

$^{13}C\{^{1}H\}$ NMR (101 MHz, $CDCl_3$) of **4v**

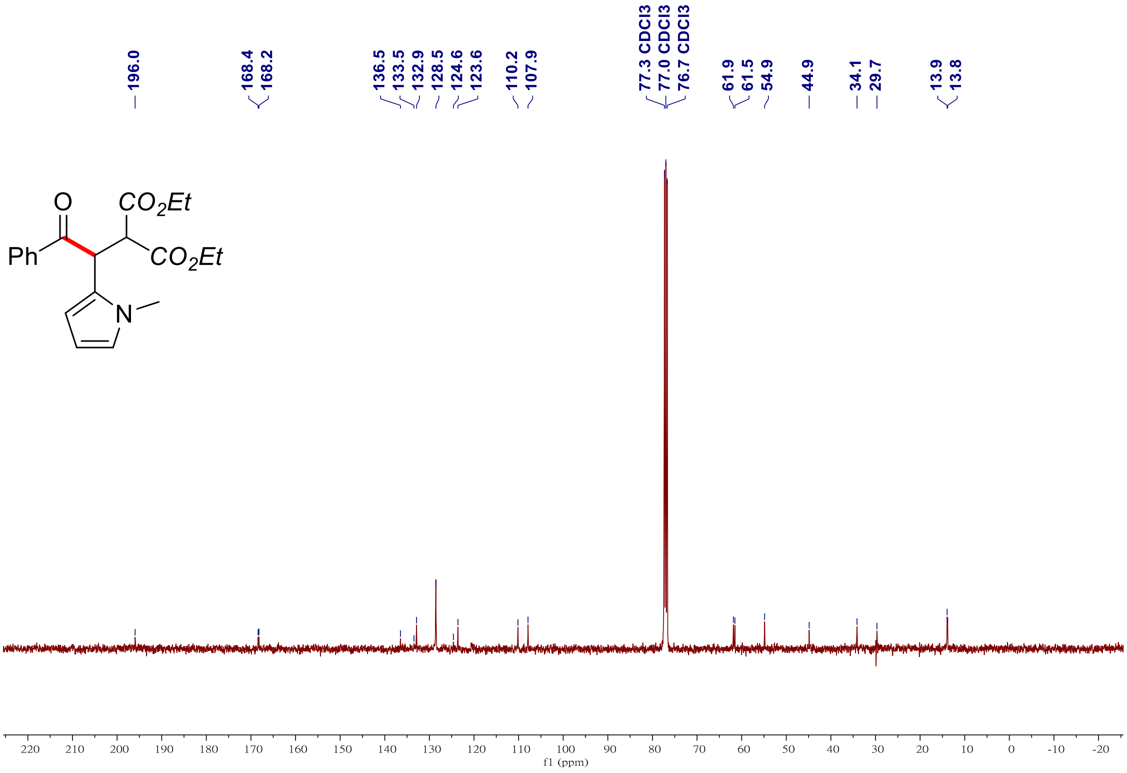

^{1}H NMR (300 MHz, $CDCl_3$) of **4w**, See procedure

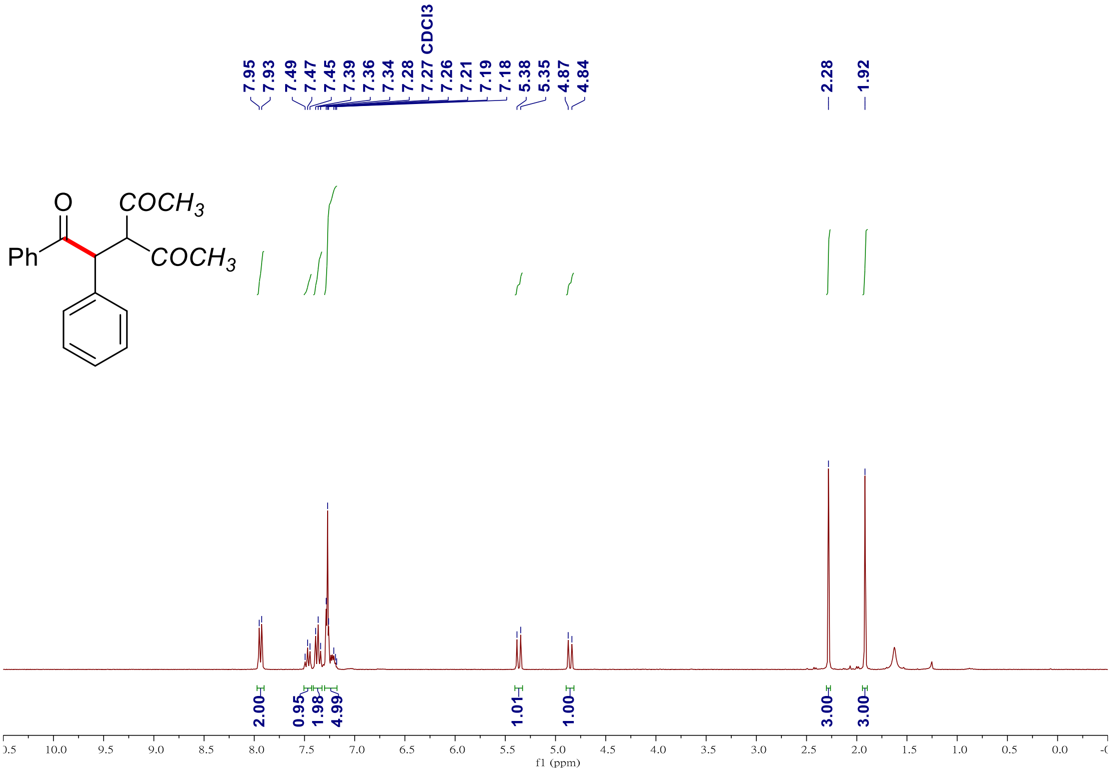

Back to the contents

$^{13}C\{^1H\}$ NMR (101 MHz, $CDCl_3$) of **4w**

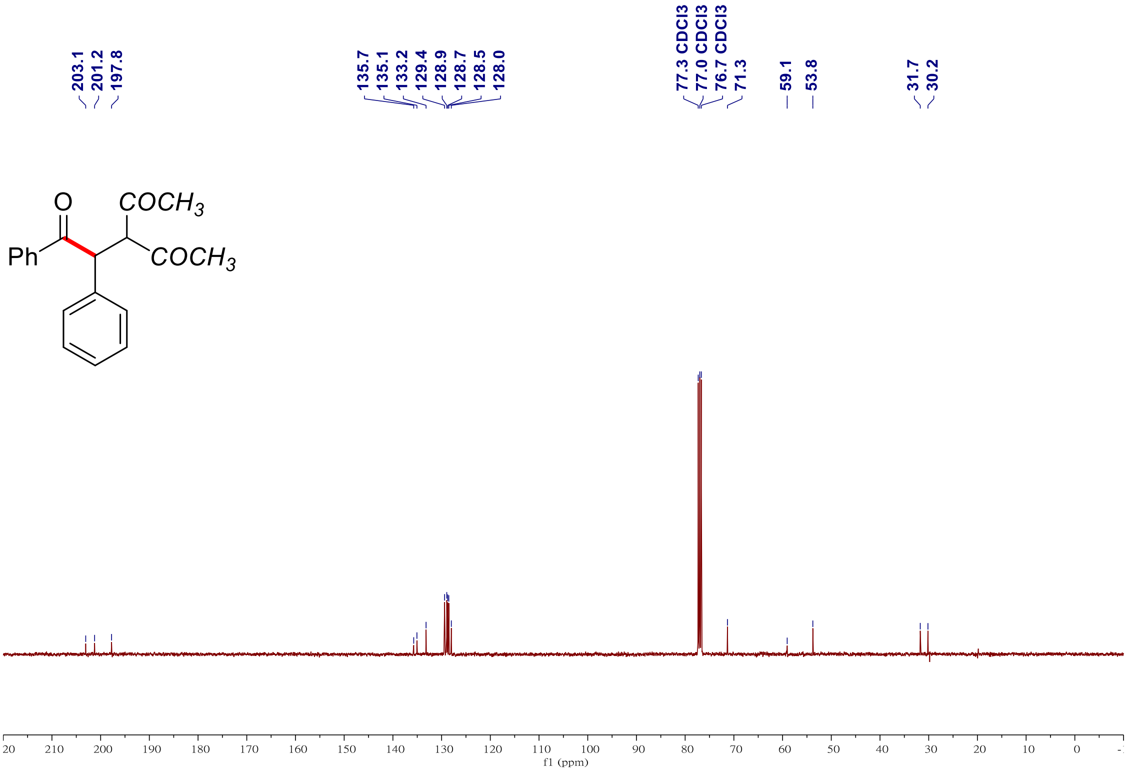

^1H NMR (400 MHz, $CDCl_3$) of **4x**, See procedure

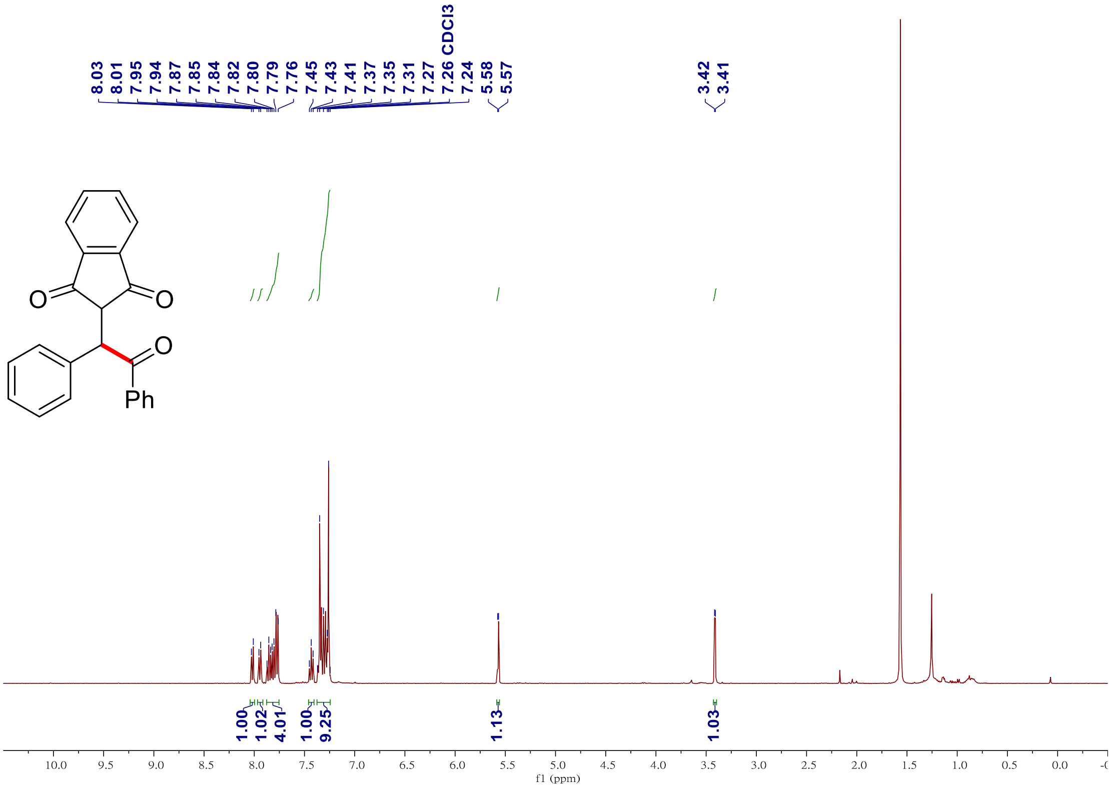

Back to the contents

$^{13}C\{^{1}H\}$ NMR (101 MHz, $CDCl_3$) of **4x**

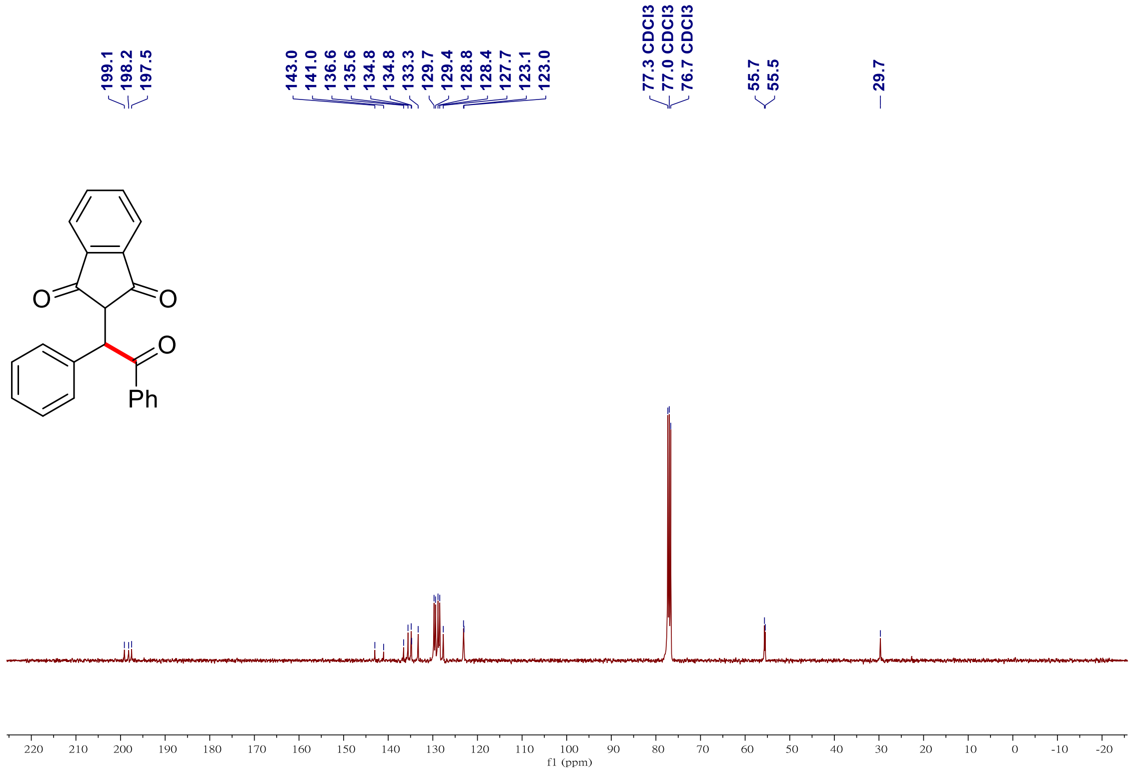

^{1}H NMR (300 MHz, $CDCl_3$) of **4y**, See procedure

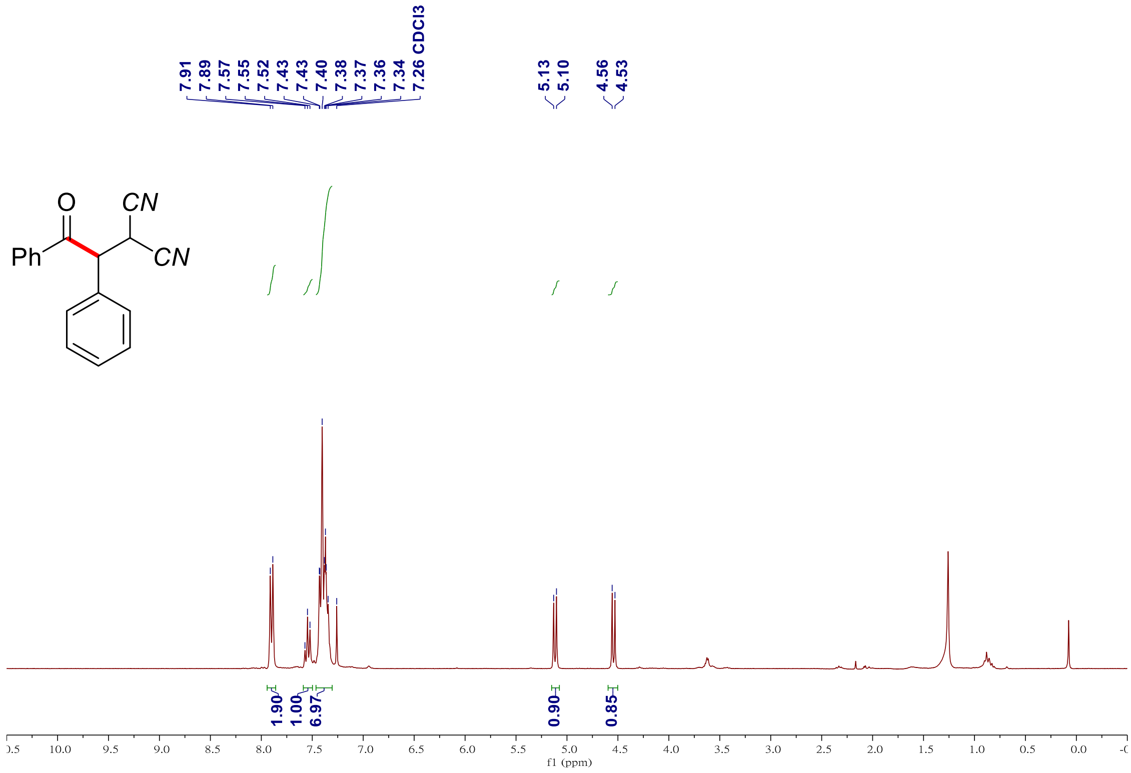

[Back to the contents](#)

$^{13}C\{^1H\}$ NMR (101 MHz, $CDCl_3$) of **4y**

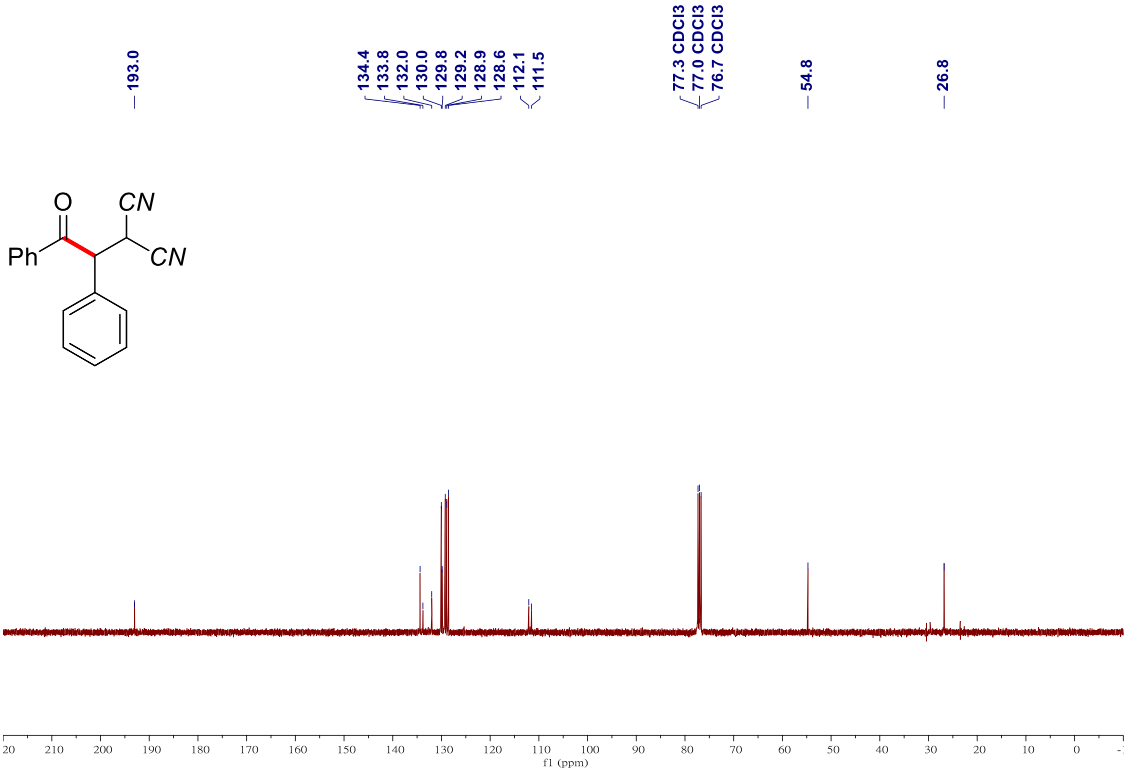

^1H NMR (300 MHz, $CDCl_3$) of **4z**, [See procedure](#)

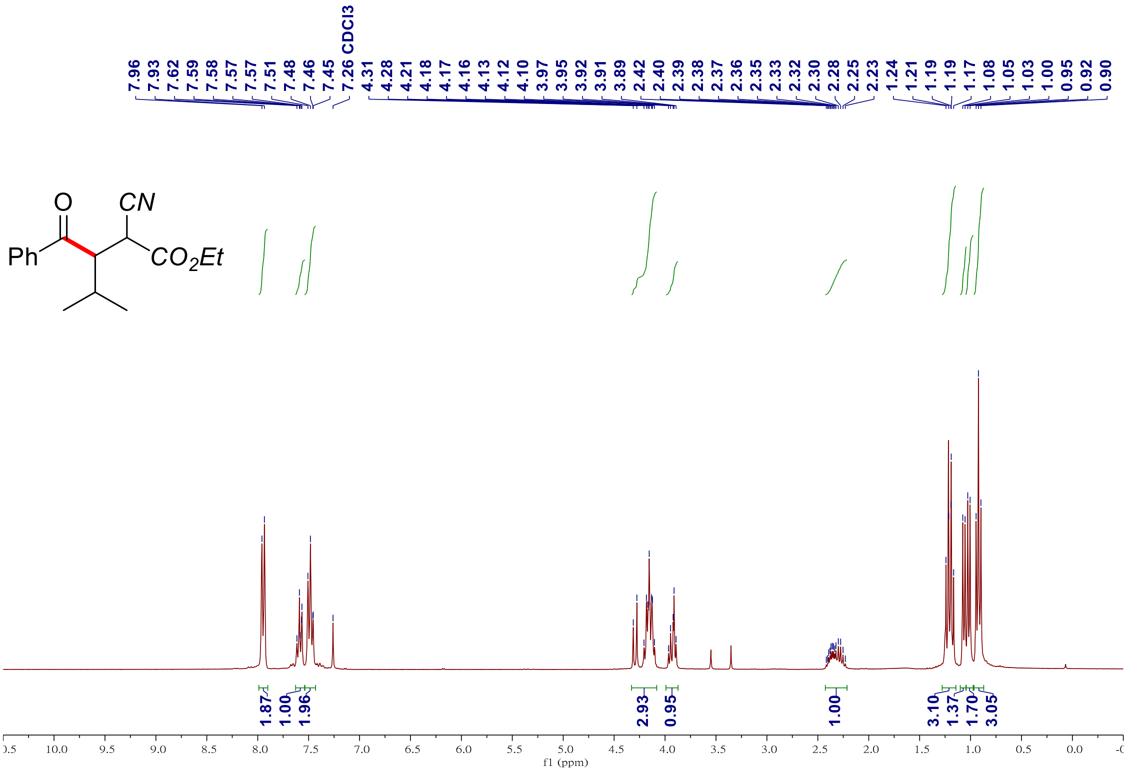

[Back to the contents](#)

$^{13}C\{^1H\}$ NMR (101 MHz, $CDCl_3$) of **4z**

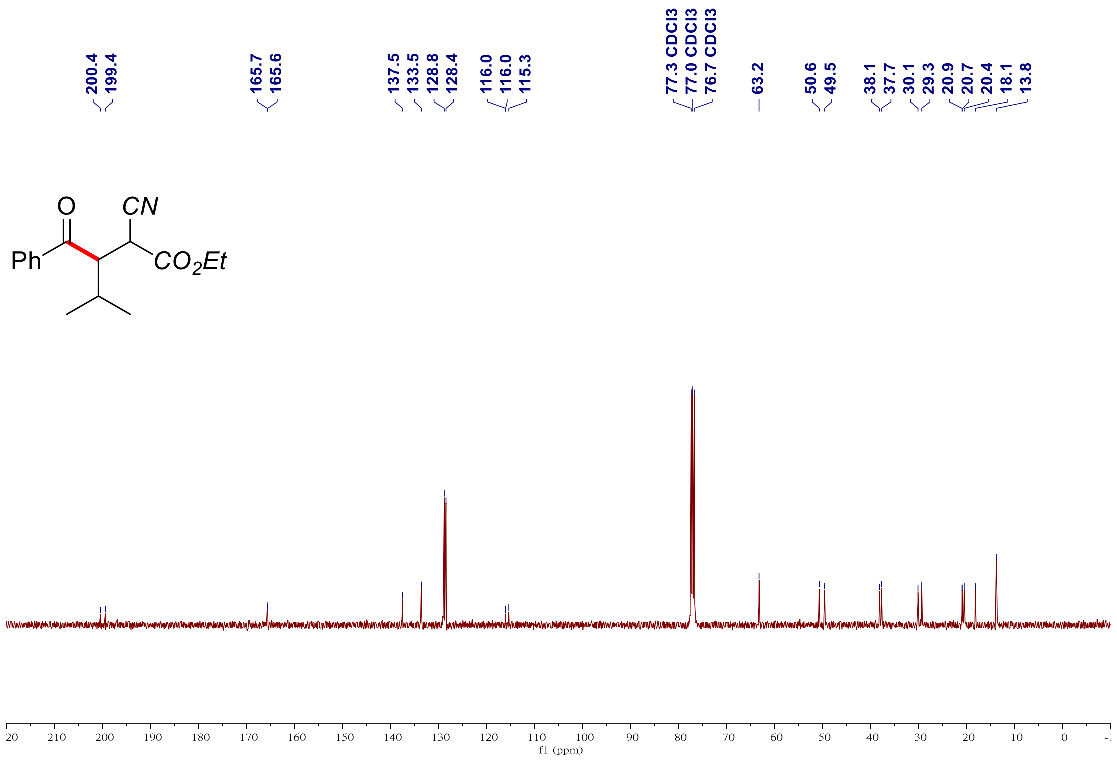

^1H NMR (300 MHz, $CDCl_3$) of **4aa**, [See procedure](#)

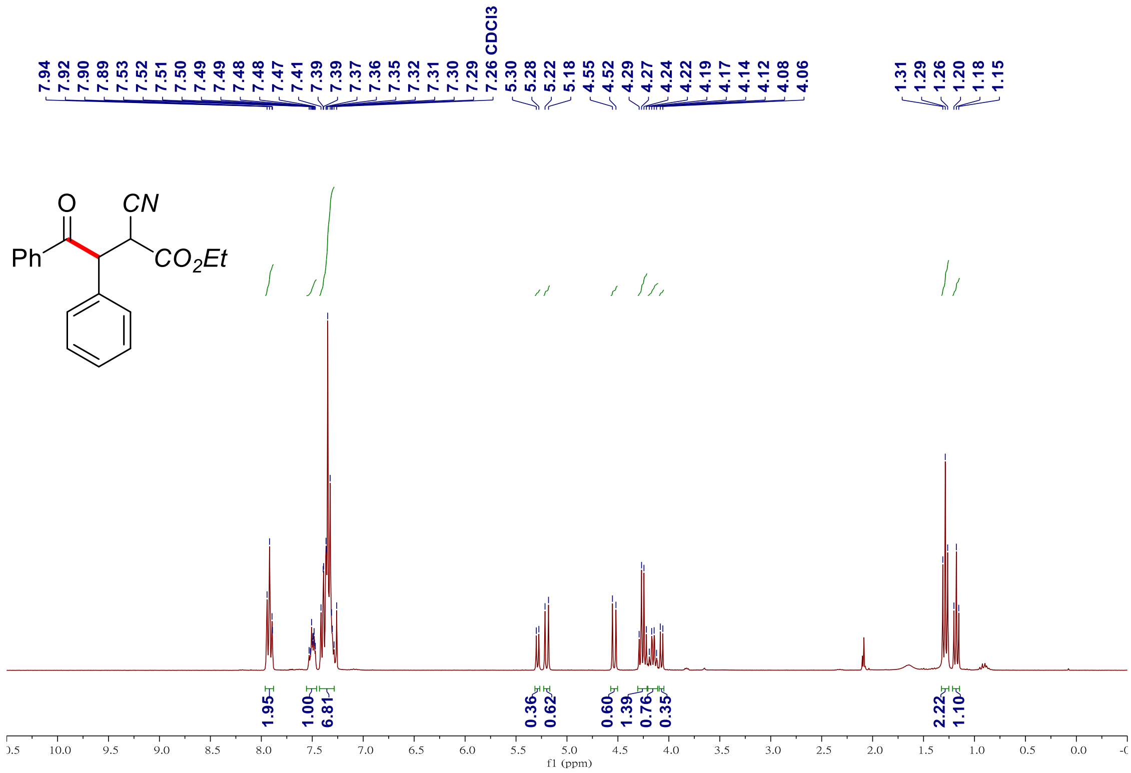

[Back to the contents](#)

$^{13}C\{^1H\}$ NMR (101 MHz, $CDCl_3$) of **4aa**

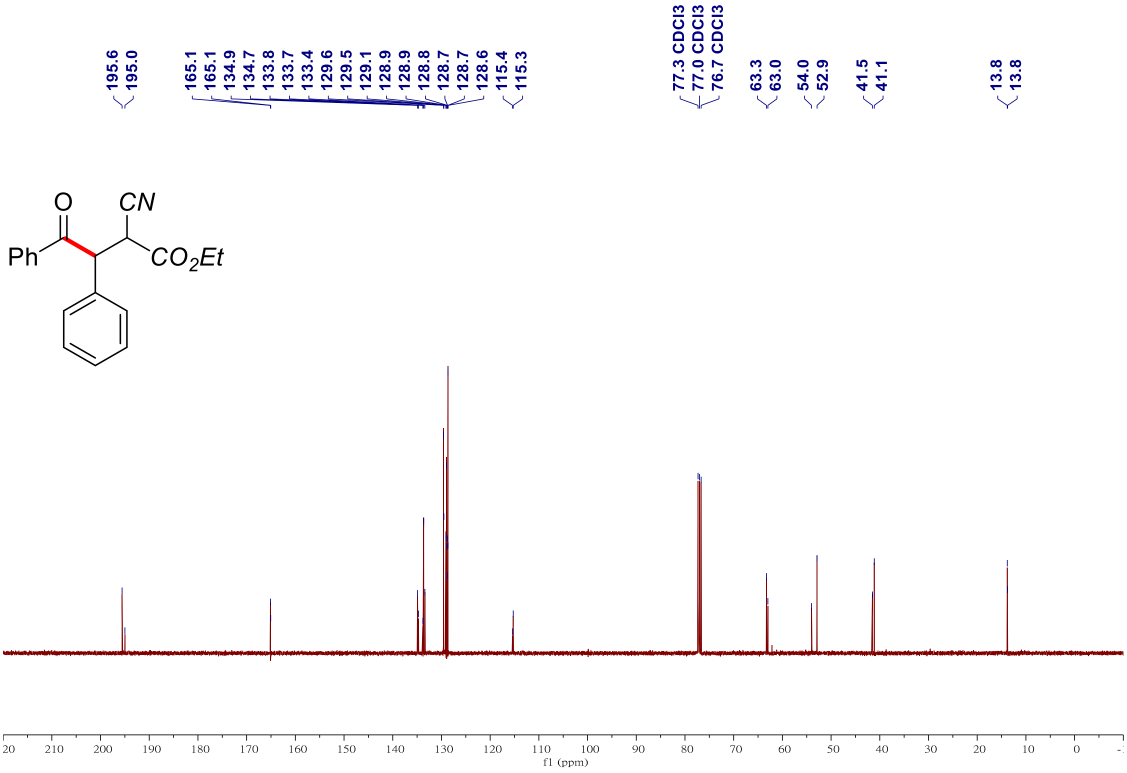

^1H NMR (300 MHz, $CDCl_3$) of **4ab**, [See procedure](#)

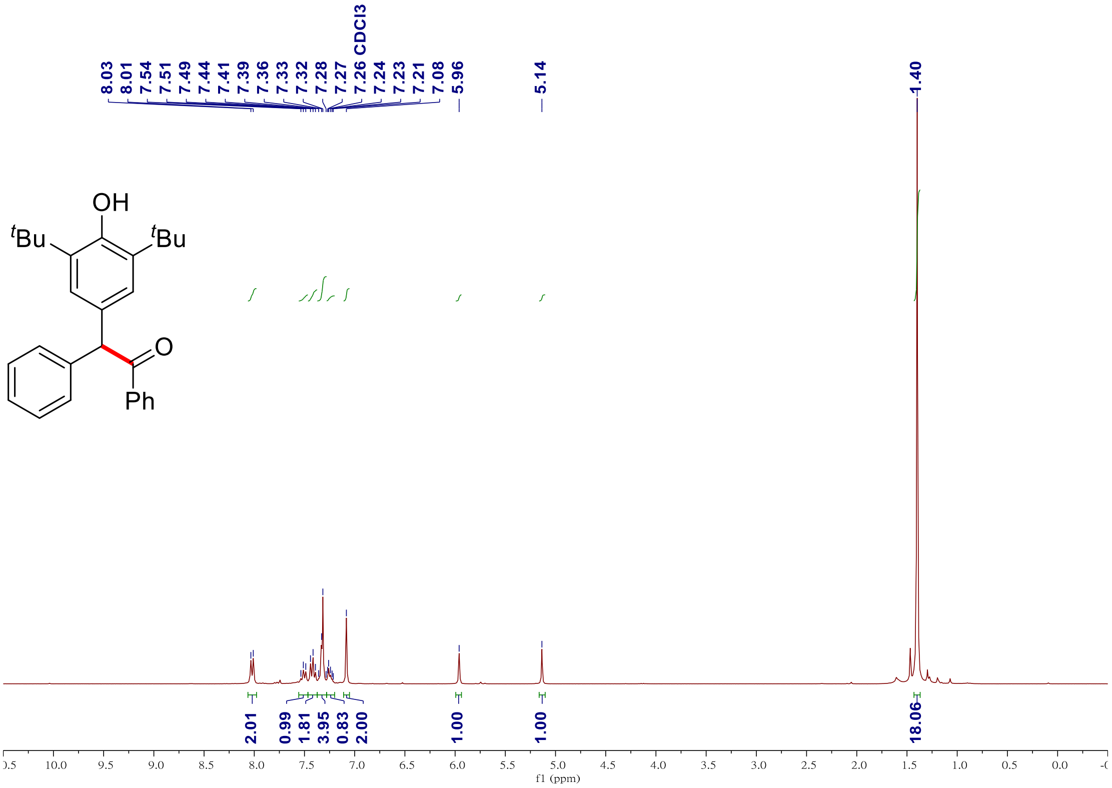

Back to the contents

$^{13}C\{^1H\}$ NMR (101 MHz, $CDCl_3$) of **4ab**

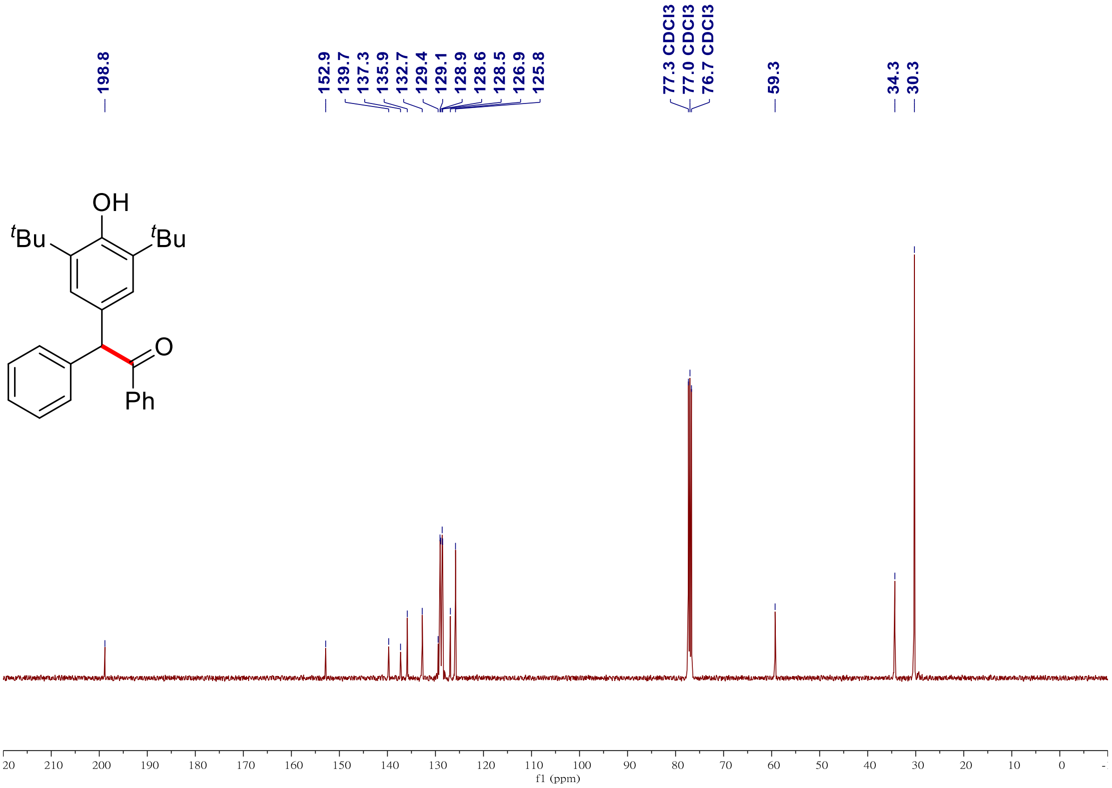

^1H NMR (400 Hz, $CDCl_3$) of **4ac**, See procedure

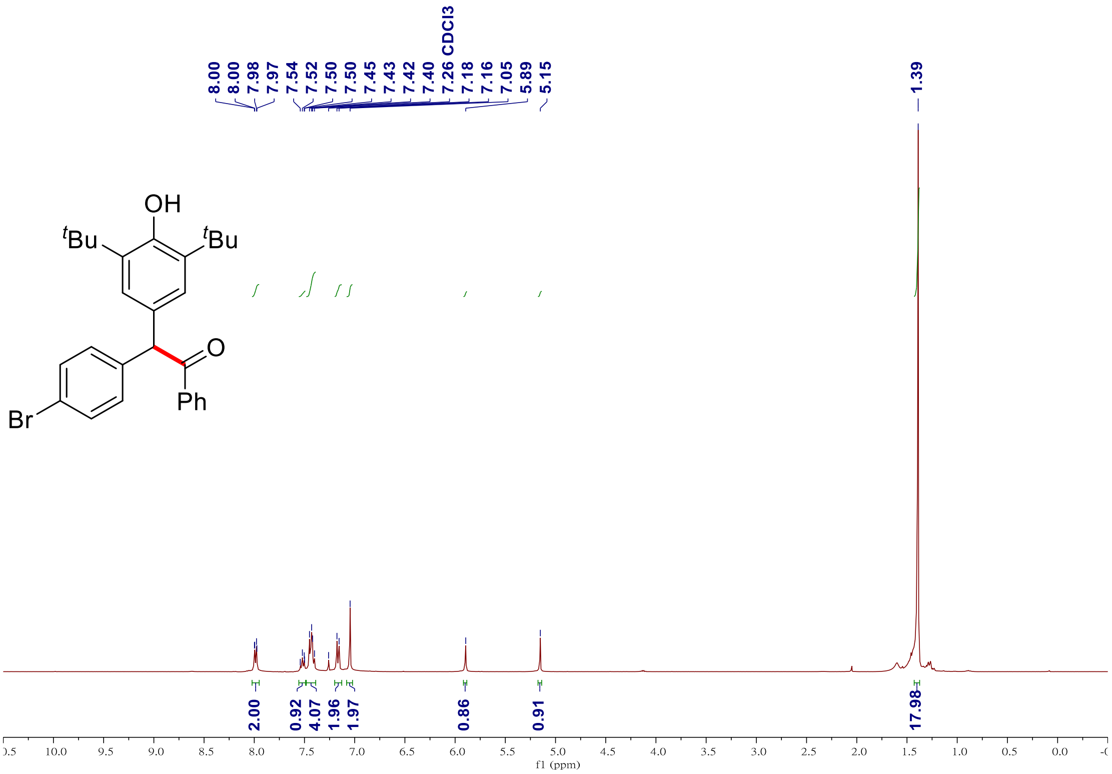

Back to the contents

$^{13}C\{^{1}H\}$ NMR (101 MHz, $CDCl_3$) of **4ac**

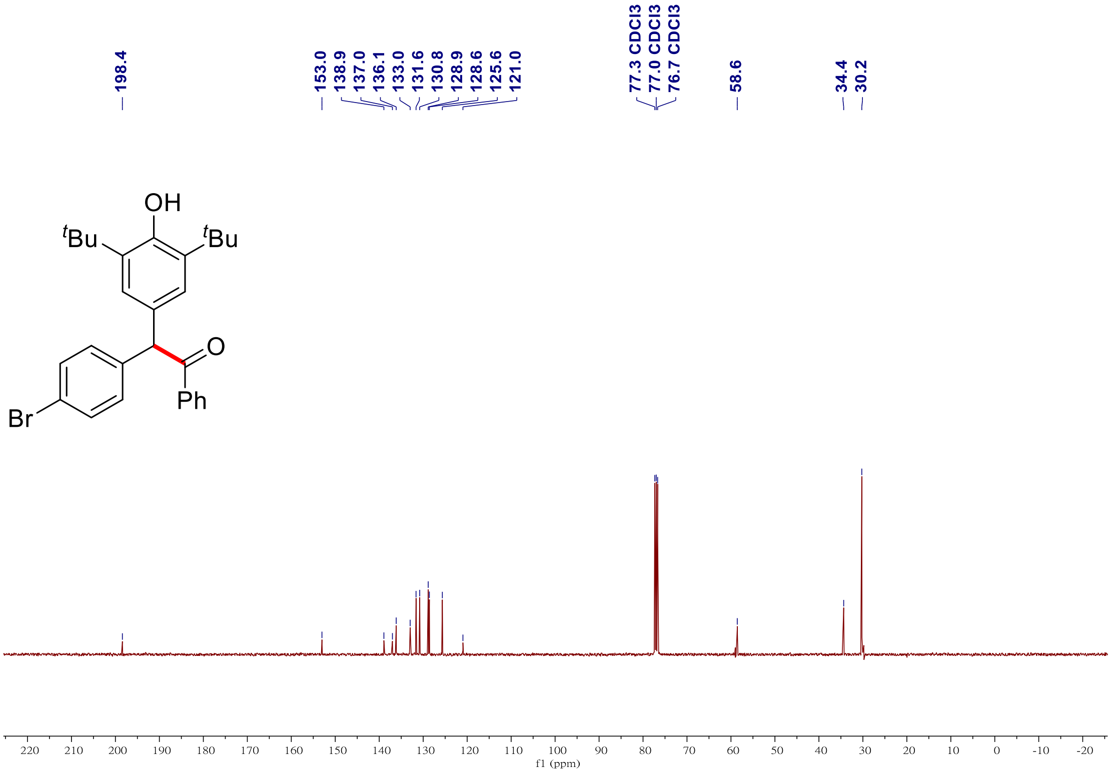

^{1}H NMR (300 MHz, $CDCl_3$) of **4ad**, See procedure

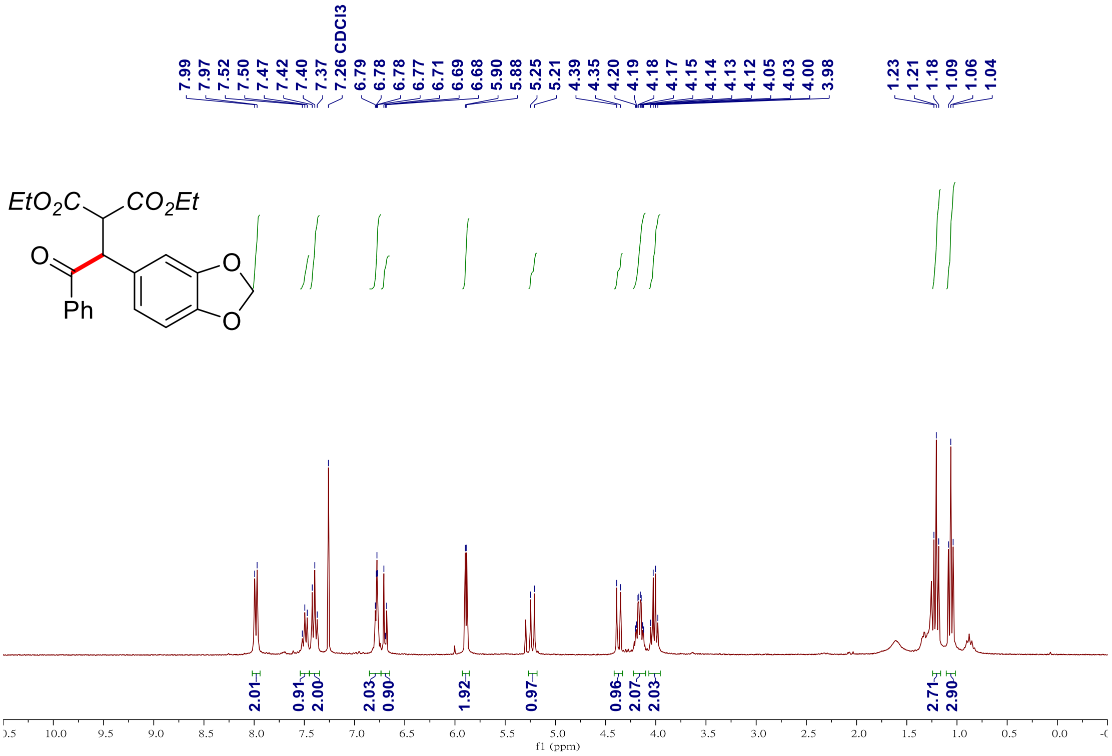

[Back to the contents](#)

$^{13}C\{^1H\}$ NMR (101 MHz, $CDCl_3$) of **4ad**

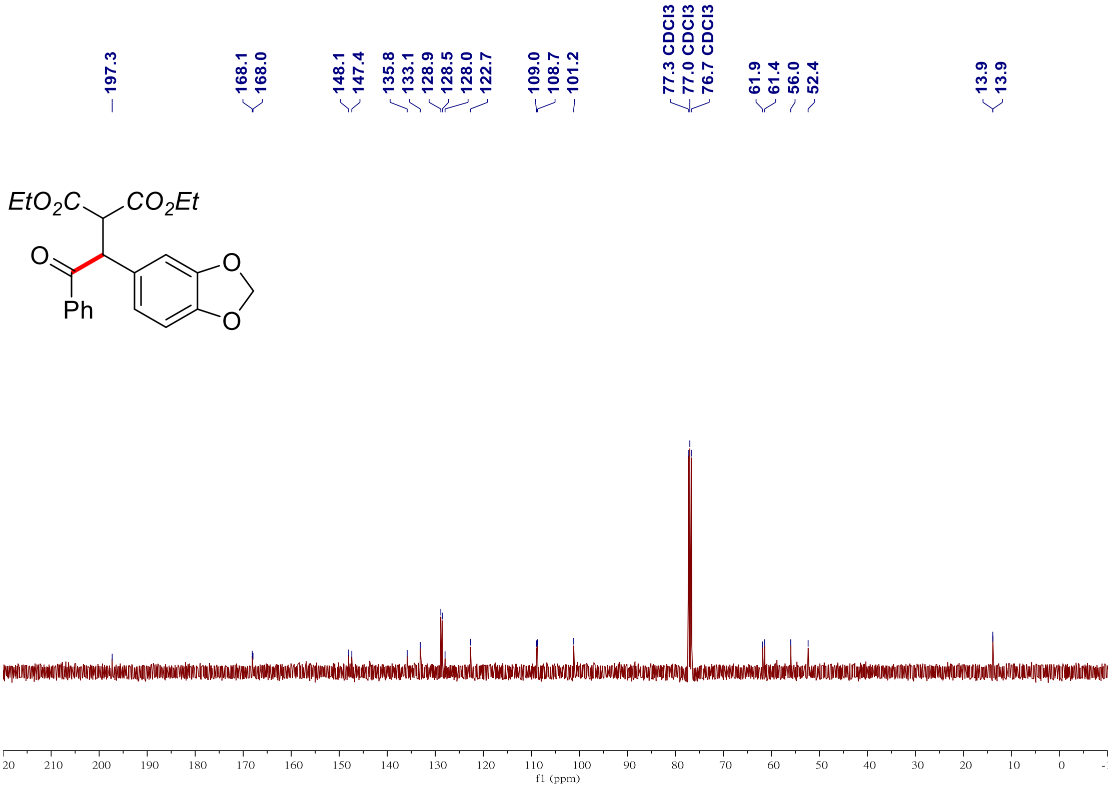

^1H NMR (300 MHz, $CDCl_3$) of **4ae**, [See procedure](#)

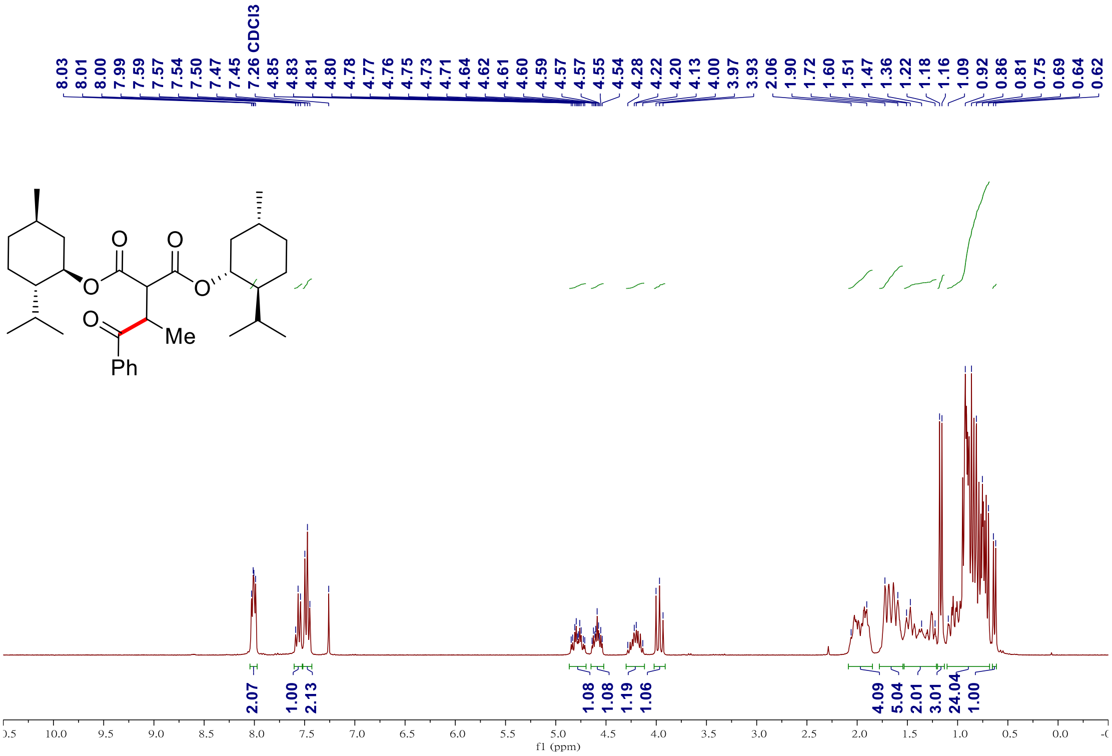

[Back to the contents](#)

$^{13}C\{^1H\}$ NMR (101 MHz, $CDCl_3$) of **4ae**

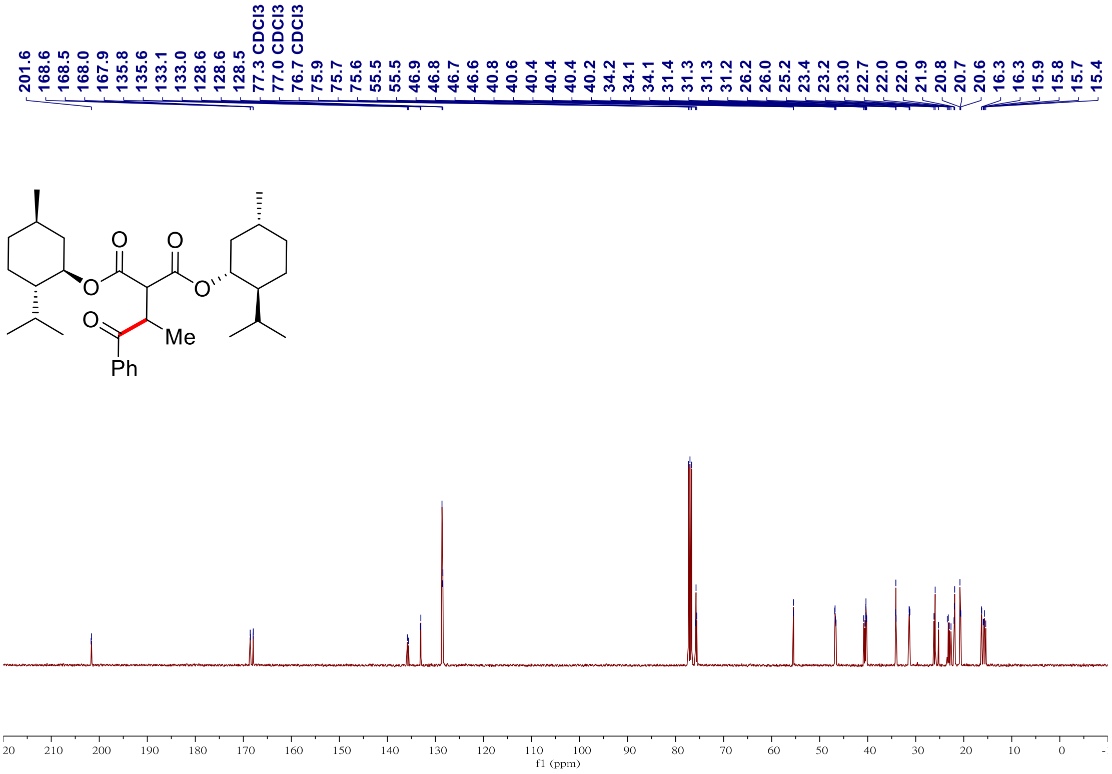

^1H NMR (300 MHz, $CDCl_3$) of **5a**, [See procedure](#)

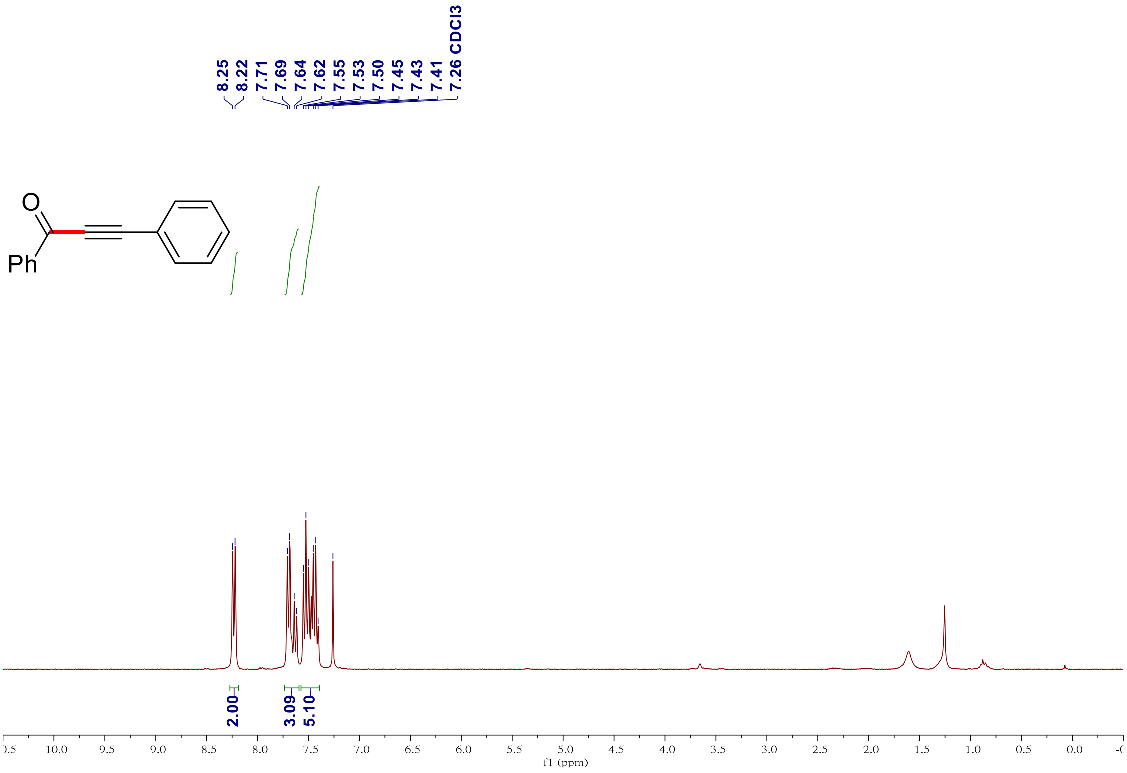

Back to the contents

¹³C NMR (101 MHz, $CDCl_3$) of **5a**

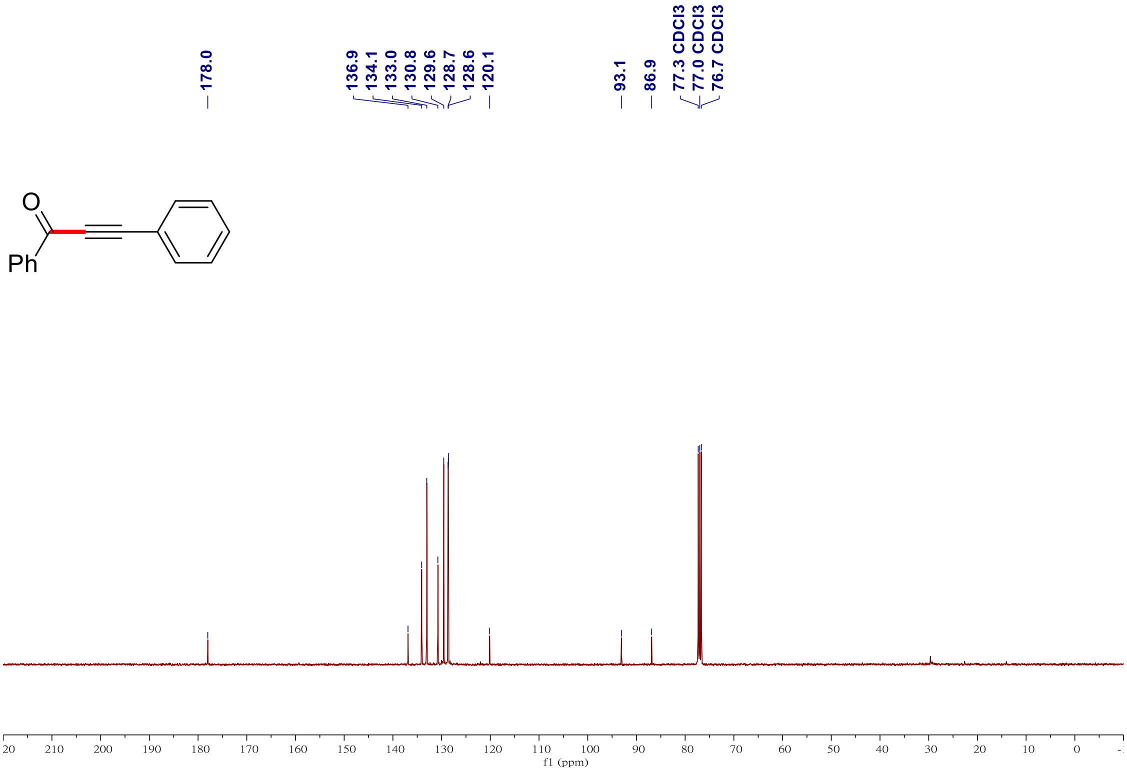

¹H NMR (300 MHz, $CDCl_3$) of **5b**, See procedure

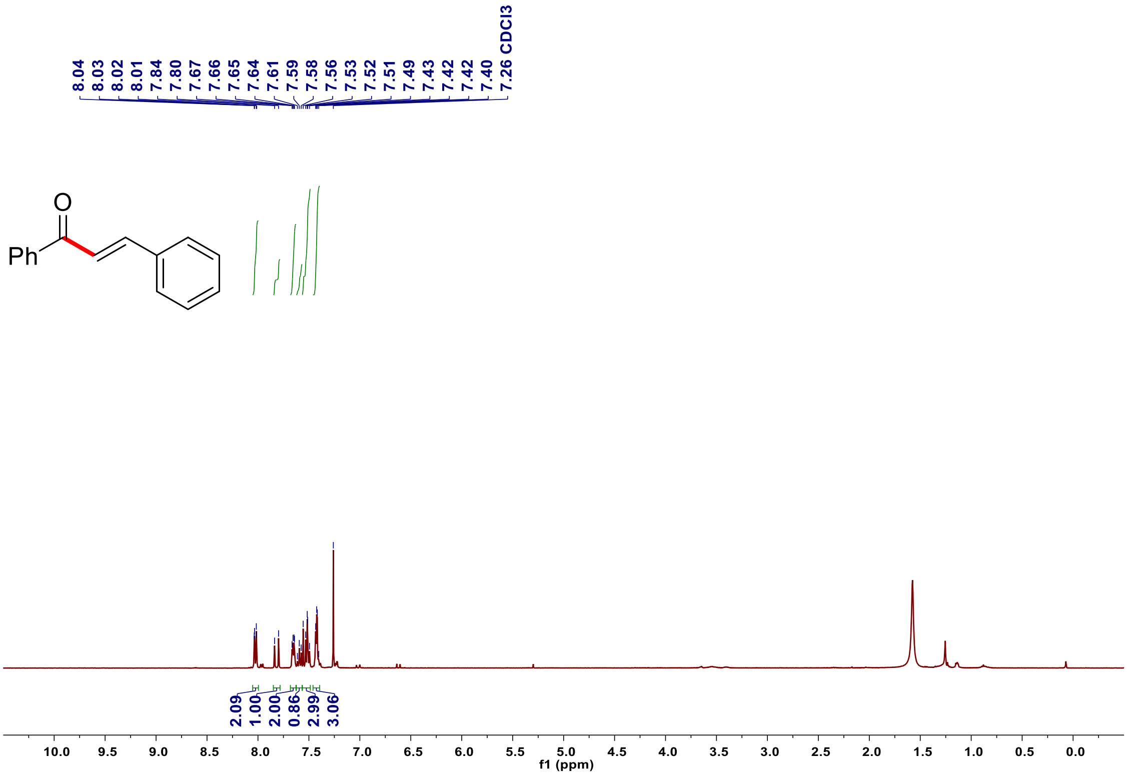

[Back to the contents](#)

^{13}C NMR (101 MHz, $CDCl_3$) of **5b**

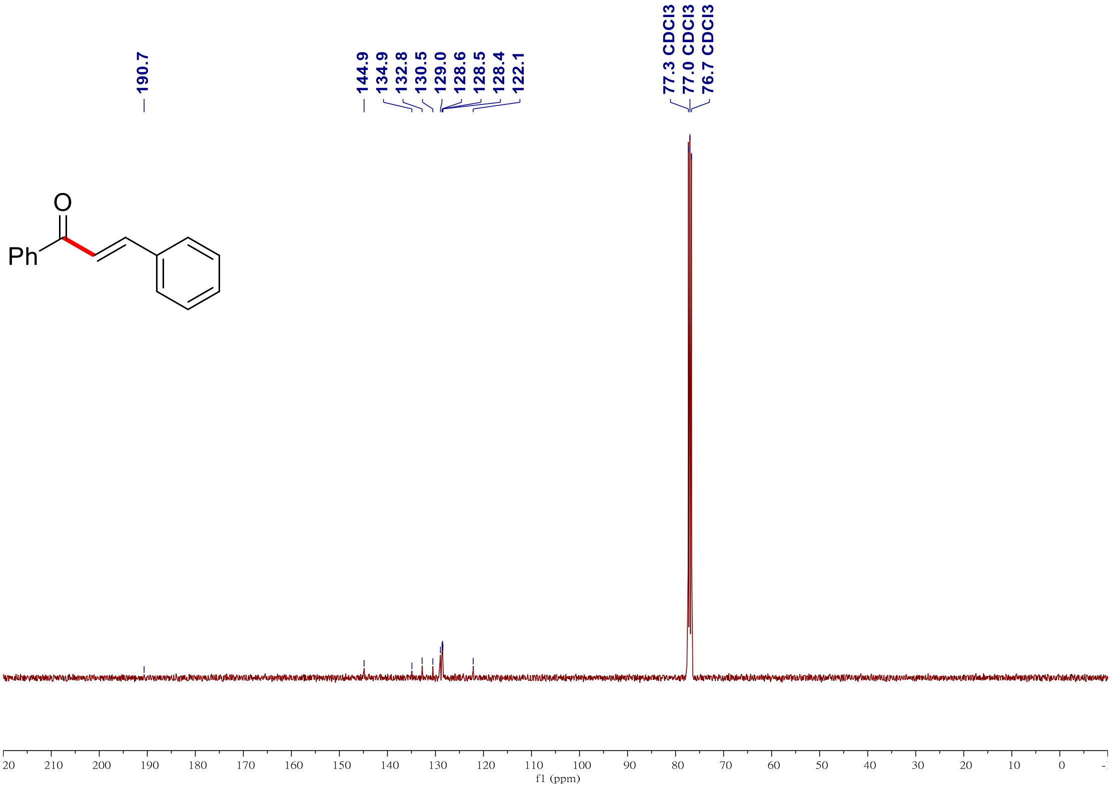

^{1}H NMR (300 MHz, $CDCl_3$) of **5c**, See procedure

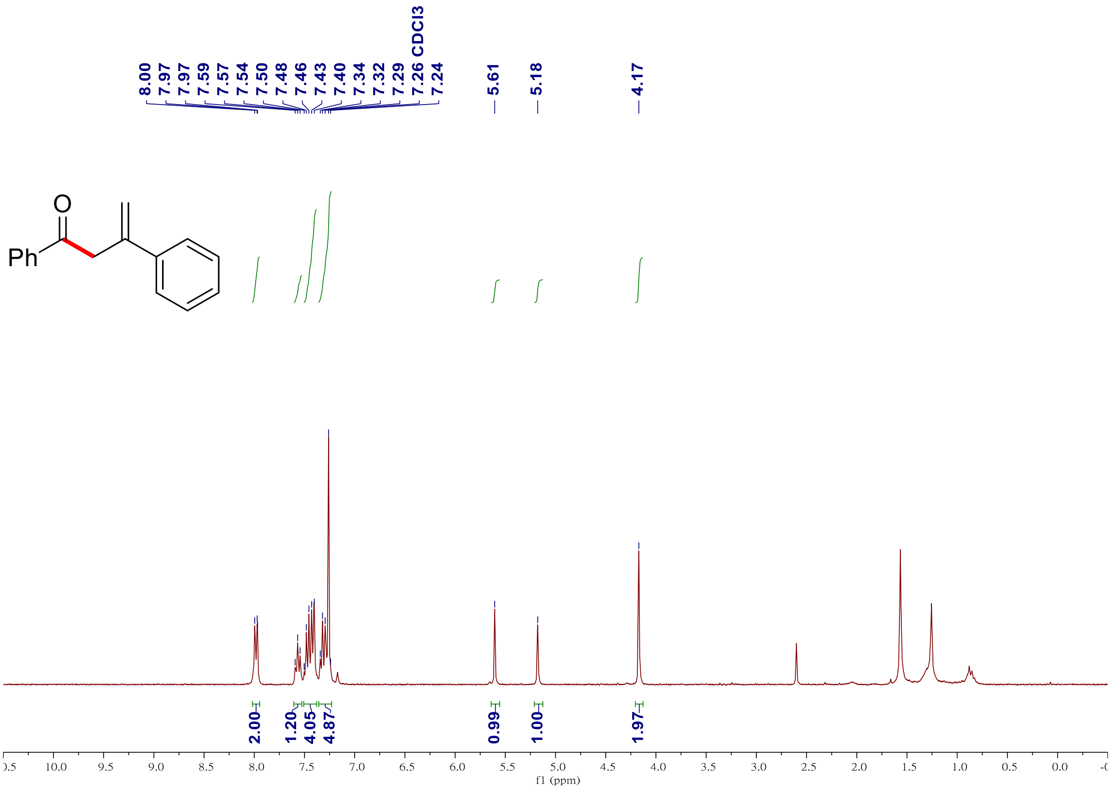

Back to the contents

^{1}H NMR (300 MHz, $CDCl_3$) of **5c'**, See procedure

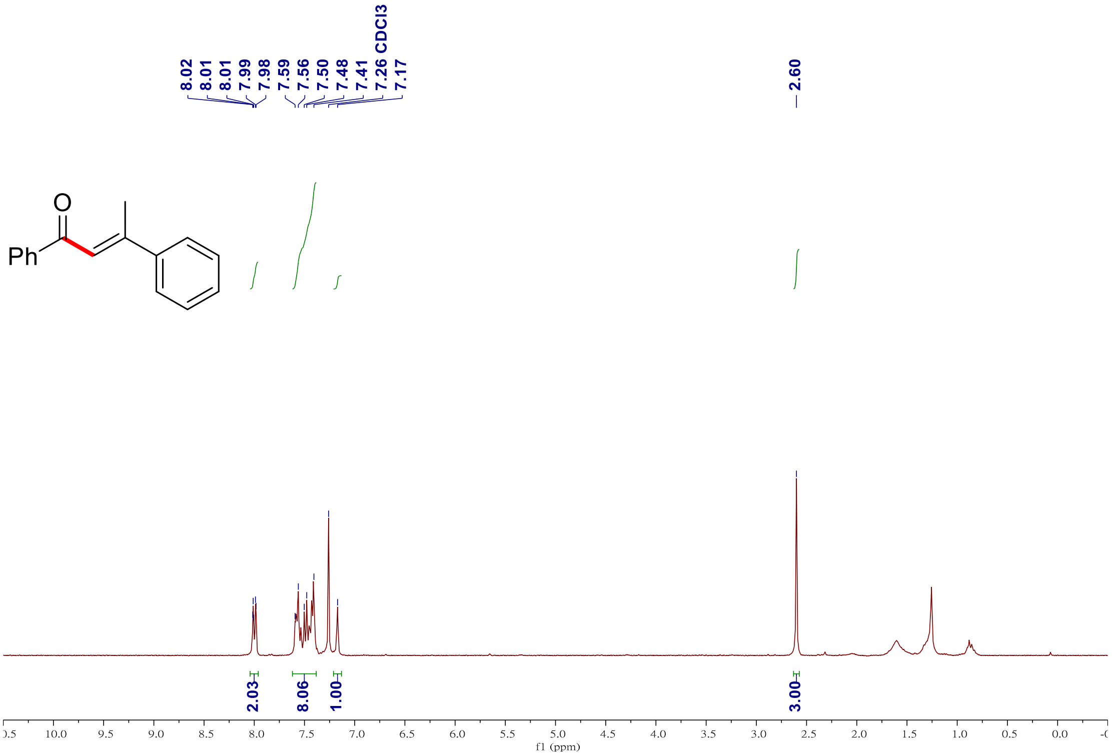

^{1}H NMR (300 MHz, $CDCl_3$) of **5d**, See procedure

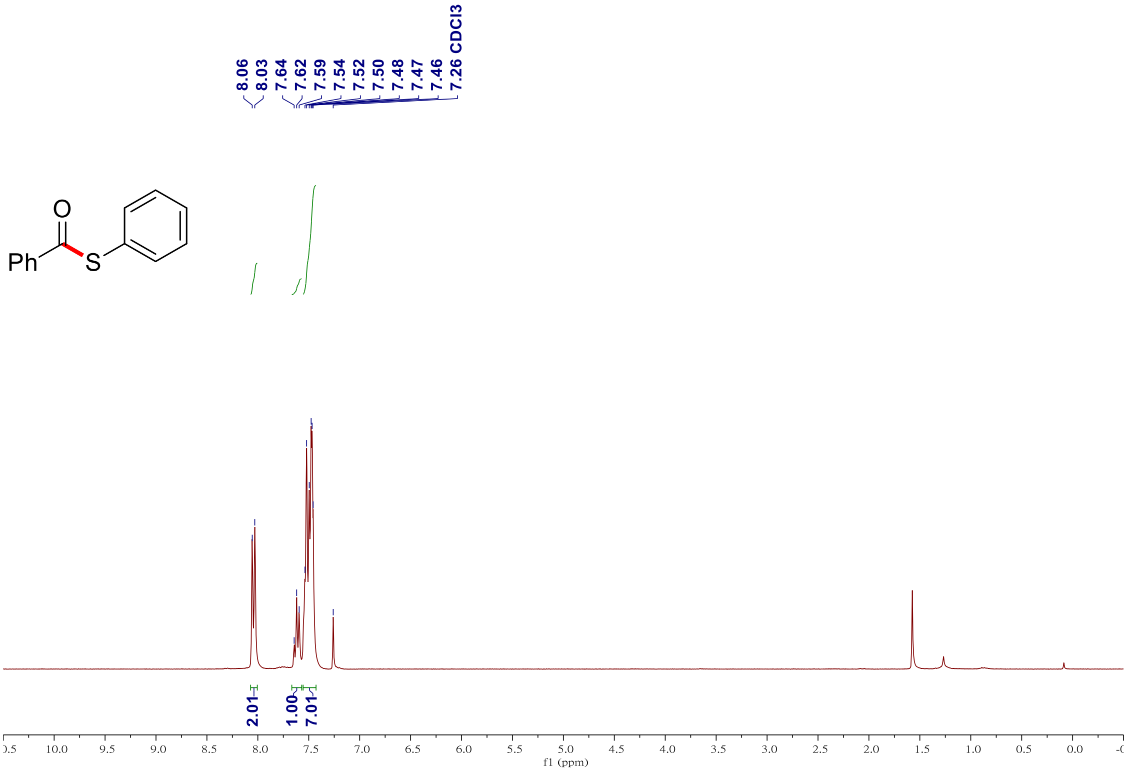

Back to the contents

$^{13}C\{^{1}H\}$ NMR (101 MHz, $CDCl_3$) of **5d**

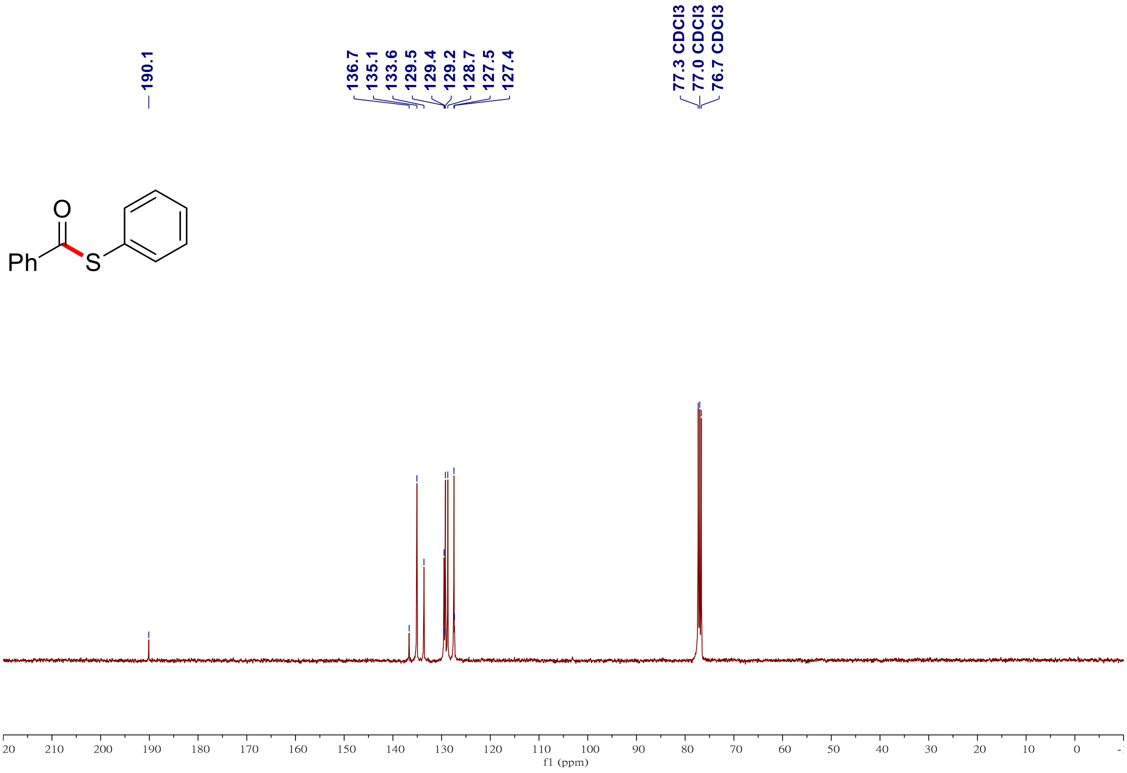

^{1}H NMR (400 MHz, $CDCl_3$) of **5e**, See procedure

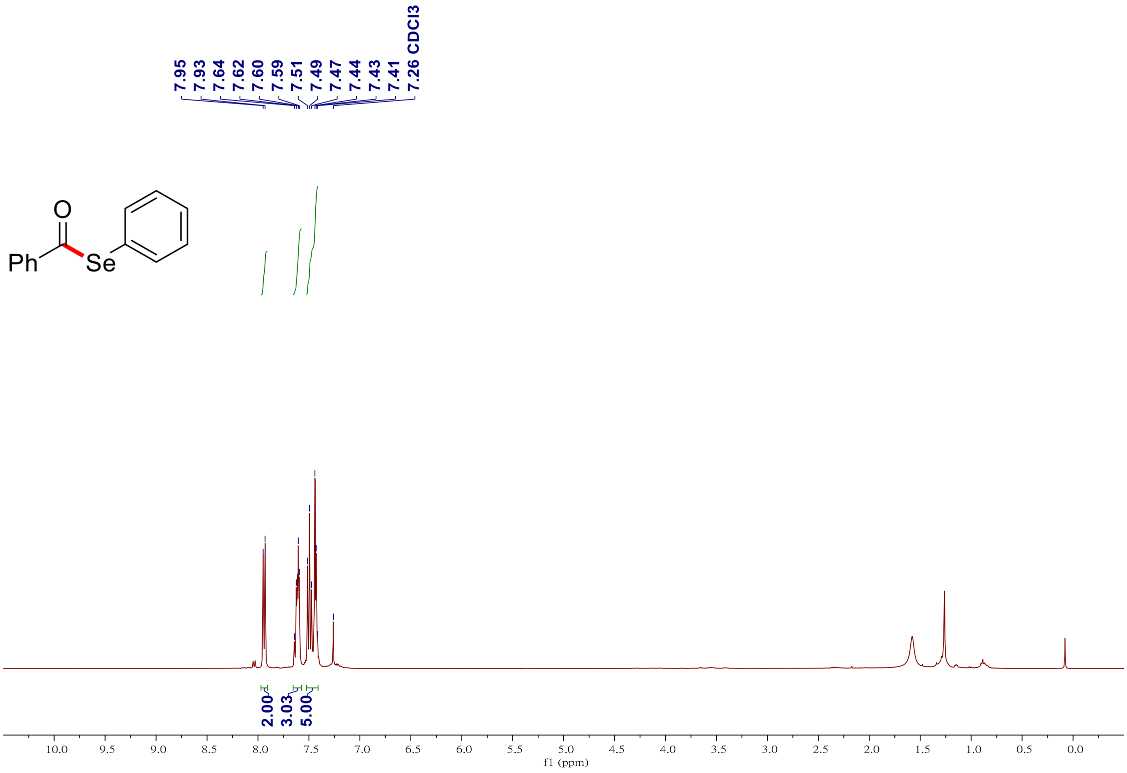

[Back to the contents](#)

$^{13}C\{^1H\}$ NMR (101 MHz, $CDCl_3$) of **5e**

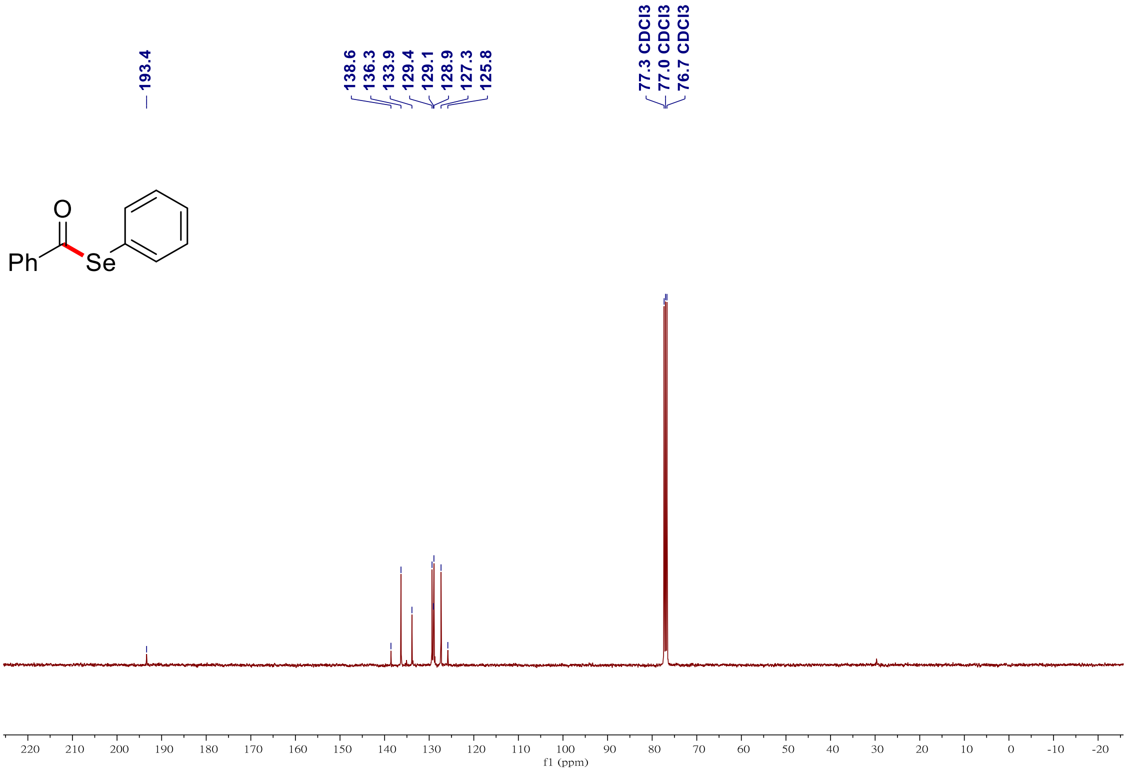

^1H NMR (400 MHz, $CDCl_3$) of **6a**, [See procedure](#)

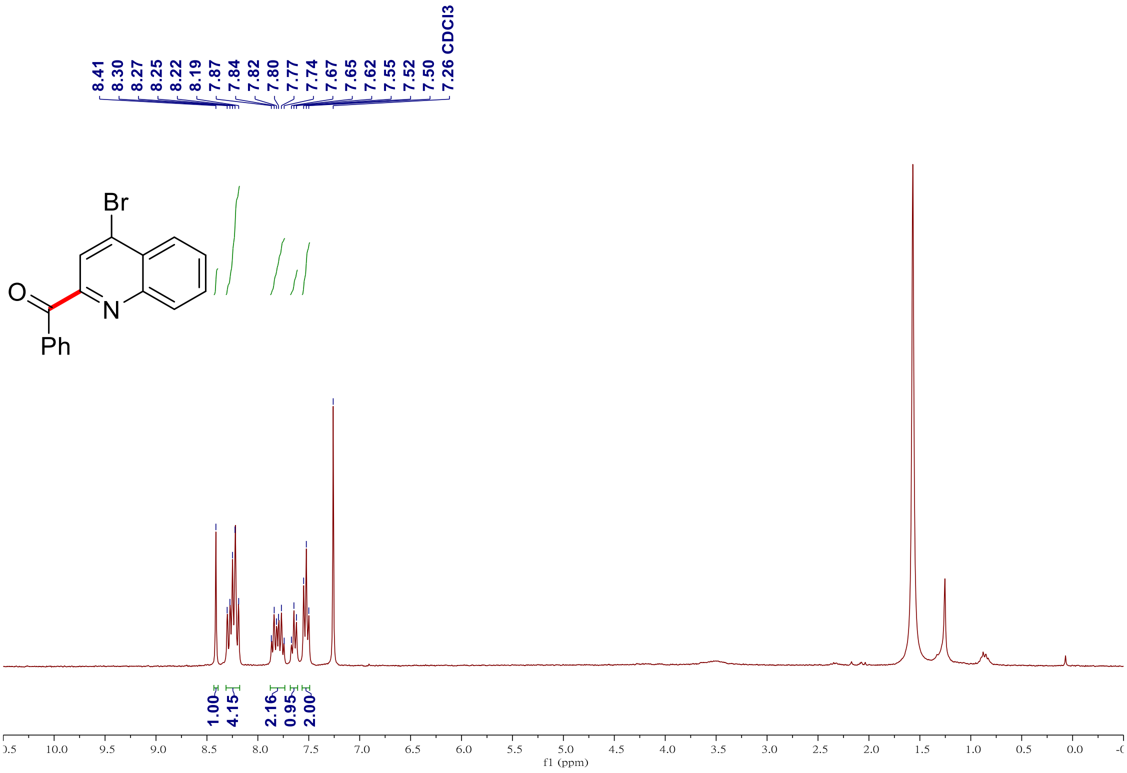

Back to the contents

^{13}**C NMR** (101 MHz, $CDCl_3$) of **6a**

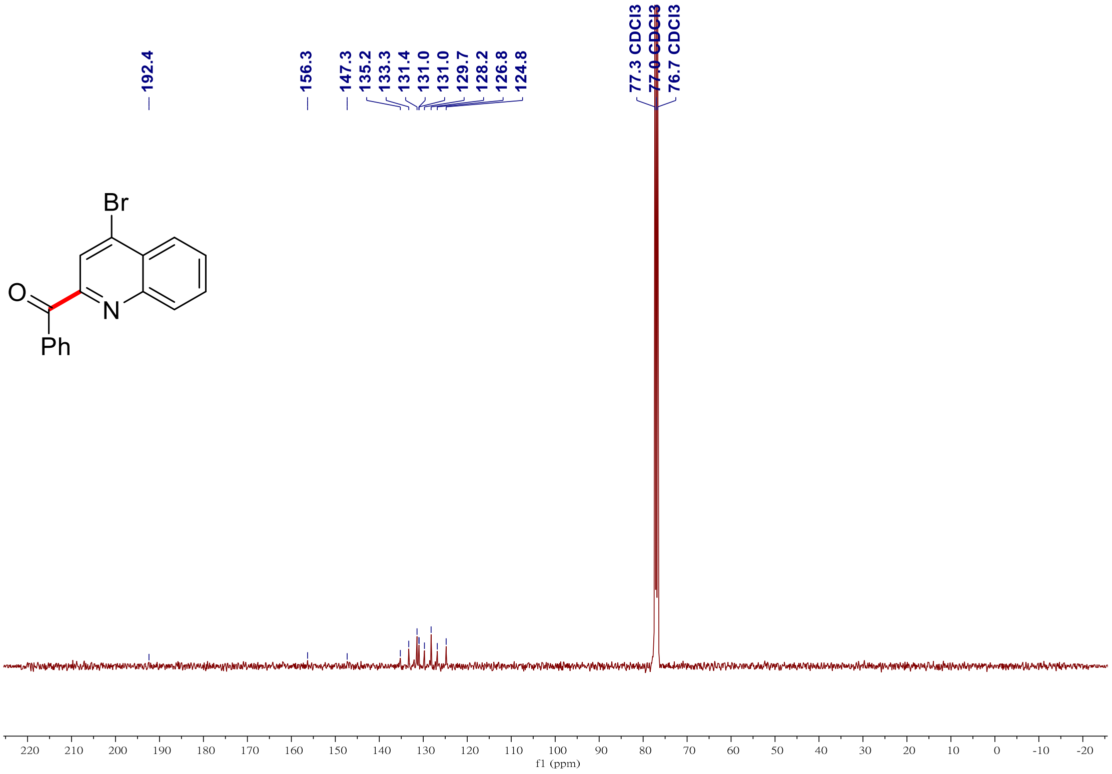

^{1}**H NMR** (400 MHz, $CDCl_3$) of **7a**, See procedure

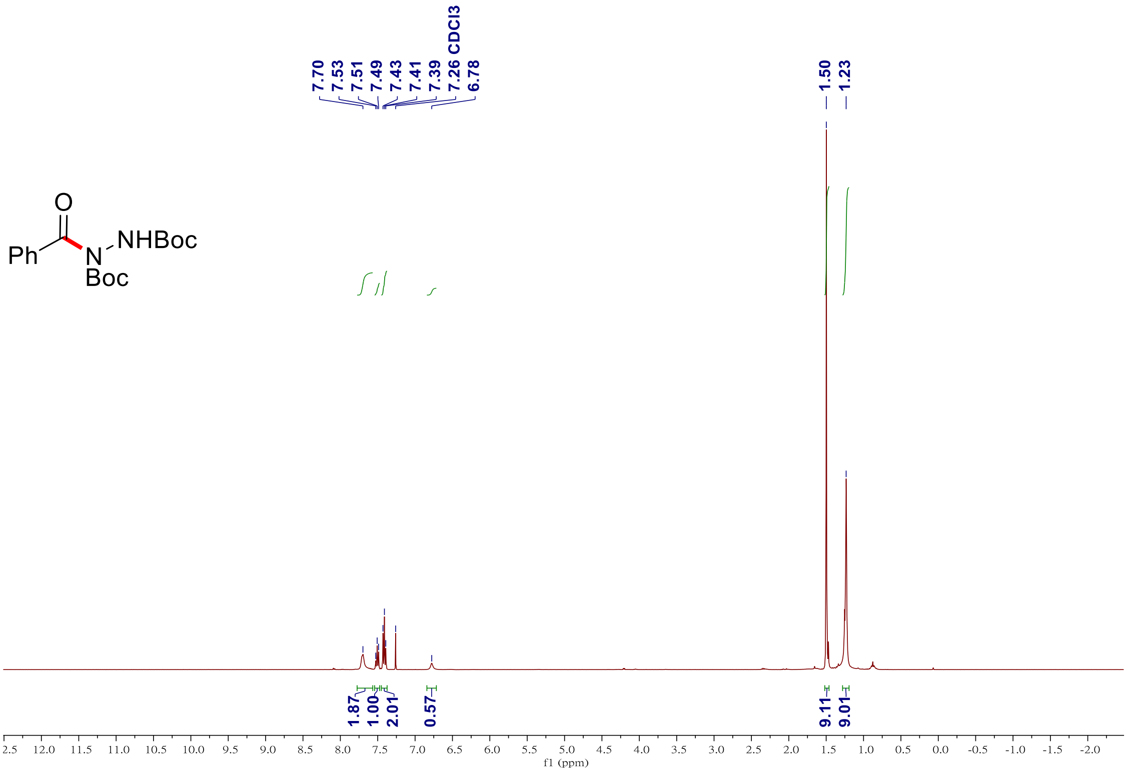

[Back to the contents](#)

^{13}C NMR (101 MHz, $CDCl_3$) of **7a**

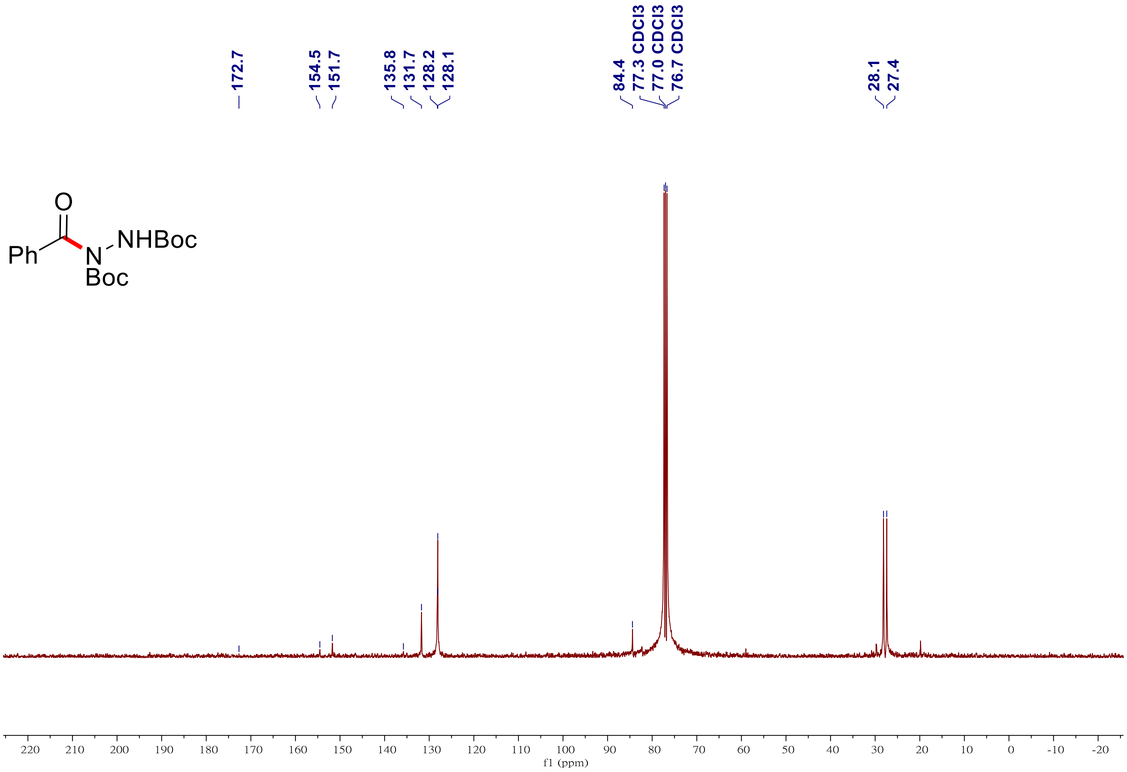

Supplement: Supplementary file 2 [file ol5c02448_si_002.pdf]
